# Distinguishing different psychiatric disorders using DDx-PRS

**DOI:** 10.1038/s41588-026-02684-x

**Published:** 2026-08-20

**Authors:** Wouter J. Peyrot, Georgia Panagiotaropoulou, Loes M. Olde Loohuis, Mark J. Adams, Ole A. Andreassen, Swapnil Awasthi, Andreas J. Forstner, Tian Ge, Andrew M. McIntosh, Brittany L. Mitchell, Niamh Mullins, Kevin S. O’Connell, Brenda W.J.H. Penninx, Danielle Posthuma, Stephan Ripke, Douglas M. Ruderfer, Emil Uffelmann, Bjarni J. Vilhjalmsson, Zhihong Zhu, Schizophrenia Working Group of the Psychiatric Genomics Consortium, Nick Bass, Aiden Corvin, Ina Giegling, Annette M. Hartmann, Kenneth S. Kendler, George K. Kirov, Sophie E. Legge, Andrew McQuillin, Michael M.C. O’Donovan, Anthony F. O’Neill, Michael J. Owen, Antonio F. Pardiñas, Brien P. Riley, Dan Rujescu, James T. Walters, Daniel R. Weinberger, Bipolar Disorder Working Group of the Psychiatric Genomics Consortium, Nick Bass, Susanne A. Bengesser, Joanna M. Biernacka, Nina Dalkner, Arianna Di Florio, Frederike T. Fellendorf, Mark A. Frye, Melanie Lenger, Susan L. McElroy, Andrew McQuillin, Eva Z. Reininghaus, Major Depressive Disorder Working Group of the Psychiatric Genomics Consortium, Mark J. Adams, Gerome Breen, Cathryn M. Lewis, Andrew M. McIntosh, Jordan W. Smoller, Alkes L. Price

**Affiliations:** 1 Department of Psychiatry, Amsterdam UMC, Vrije Universiteit, Amsterdam, The Netherlands; 2 Department of Complex Trait Genetics, Center for Neurogenomics and Cognitive Research, Amsterdam Neuroscience, Vrije Universiteit Amsterdam, Netherlands; 3 Amsterdam Public Health, Amsterdam UMC, Vrije Universiteit, Amsterdam, The Netherlands; 4 Dept. of Psychiatry and Psychotherapy, Charité - Universitätsmedizin, Berlin 10117, Germany; 5 Center for Neurobehavioral Genetics, University of California Los Angeles, Los Angeles, CA, USA; 6 Department of Genetics and Genomics, University of California Los Angeles, Los Angeles, CA, USA; 7 Department of Computational Medicine, University of California Los Angeles, Los Angeles, CA, USA; 8 Institute of Neuroscience and Cardiovascular Research, University of Edinburgh, Edinburgh, UK; 9 Division of Mental Health and Addiction, Oslo University Hospital, Oslo, NO; 10 Centre for Precision Psychiatry, University of Oslo, Oslo, NO; 11 KG Jebsen Centre for Neurodevelopmental Disorders, University of Oslo, Oslo, NO; 12 Institute of Human Genetics, University of Bonn, School of Medicine & University Hospital Bonn, Bonn, Germany; 13 Institute of Neuroscience and Medicine (INM-1), Research Center Jülich, Jülich, Germany; 14 Psychiatric and Neurodevelopmental Genetics Unit, Center for Genomic Medicine, Massachusetts General Hospital, Boston, MA, USA; 15 Center for Precision Psychiatry, Department of Psychiatry, Massachusetts General Hospital, Boston, MA, USA; 16 Mental Health and Neuroscience, QIMR Berghofer Medical Research Institute, Brisbane, QLD, Australia; 17 Department of Psychiatry, Icahn School of Medicine at Mount Sinai, New York, NY, US; 18 Charles Bronfman Institute for Personalized Medicine, Icahn School of Medicine at Mount Sinai, New York, NY, US; 19 Department of Genetics and Genomic Sciences, Icahn School of Medicine at Mount Sinai, New York, NY, US; 20 NORMENT, University of Oslo, Oslo, NO; 21 Stanley Center for Psychiatric Research, Broad Institute of Harvard and MIT, Cambridge MA 02142, USA; 22 German Center for Mental Health (DZPG), site Berlin-Potsdam; 23 Center for Digital Genomic Medicine, Department of Medicine, Vanderbilt University Medical Center, Nashville, TN, US; 24 Departments of Biomedical Informatics and Psychiatry and Behavioral Sciences, Vanderbilt University Medical Center, Nashville, TN, US; 25 Division of Genetic Medicine, Department of Medicine, Vanderbilt Genetics Institute, Vanderbilt University Medical Center, Nashville, TN, US; 26 National Centre for Register-based Research, Aarhus University, Aarhus, Denmark; 27 Bioinformatics Research Centre, Aarhus University, Aarhus, Denmark; 28 Novo Nordisk Foundation Center for Genomics Mechanisms of Disease, Broad Institute of MIT and Harvard, Cambridge, MA, USA; 29 Department of Epidemiology, Harvard T.H. Chan School of Public Health, Boston, MA, USA; 30 Department of Biostatistics, Harvard T.H. Chan School of Public Health, Boston, MA, USA; 31 Program in Medical and Population Genetics, Broad Institute of MIT and Harvard, Cambridge, MA, USA; 32 Division of Psychiatry, University College London, London, UK; 33 Department of Psychiatry, Trinity College Dublin, Dublin, Ireland; 34 Department of Psychiatry and Psychotherapy, Comprehensive Center for Clinical Neurosciences and Mental Health, Medical University of Vienna, Austria; 35 Virginia Institute for Psychiatric and Behavioral Genetics, Department of Psychiatry, Virginia Commonwealth University, Richmond VA, USA; 36 Centre for Neuropsychiatric Genetics and Genomics, Department of Psychological Medicine and Clinical Neurosciences, Cardiff University School Of Medicine, Cardiff CF24 4HQ, UK; 37 Centre For Public Health, Institute Of Clinical Sciences, Queens University Belfast, Belfast, NI; 38 Virginia Institute for Psychiatric and Behavioral Genetics, Departments of Psychiatry and Cellular, Molecular & Genetic Medicine, Virginia Commonwealth University, Richmond VA, USA; 39 Lieber Institute for Brain Development, Departments of Psychiatry, Neurology, Neuroscience and Genetic Medicine, Johns Hopkins School of Medicine, Baltimore, Md. 21205 USA; 40 Clinical Division of Psychiatry and Psychotherapeutic Medicine, Medical University of Graz, Austria; 41 Department of Quantitative Health Sciences, Mayo Clinic, Rochester, MN, USA; 42 Department of Psychiatry and Psychology, Mayo Clinic, Rochester, MN, USA; 43 Department of Psychiatry and Psychology, Mayo Clinic, Rochester, MN, USA 55905; 44 Lindner Center of HOPE, Manon, OH & University of Cincinnati College of Medicine, Cincinnati, OH, USA; 45 Social, Genetic and Developmental Psychiatry Centre, King’s College London, London, UK

## Abstract

Despite great progress on case-control polygenic prediction, there remains an unmet need for a method that genetically distinguishes clinically related disorders (e.g. schizophrenia (SCZ) vs. bipolar disorder (BIP) vs. depression (MDD) vs. control). We introduce DDx-PRS, which jointly estimates posterior probabilities of each diagnostic category (e.g. SCZ=50%, BIP=25%, MDD=15%, control=10%) by modeling variance/covariance structure across disorders, leveraging case-control polygenic risk scores and prior clinical probabilities for each diagnostic category. We applied DDx-PRS to Psychiatric Genomics Consortium SCZ/BIP/MDD/control data, including summary-level training data from 3 case-control GWAS (*N*=41,917-173,140 cases; total *N*=1,048,683) and held-out test data from different cohorts with equal numbers of each diagnostic category (total *N*=11,460). DDx-PRS was well-calibrated and well-powered (consistent with simulations), and produced comparable results to methods that require tuning data. True diagnosis probabilities in top deciles of predicted diagnosis probabilities were considerably larger than prior baseline probabilities, implying appreciable potential for clinical utility in certain settings.

## Introduction

Great progress has been made in methods for case-control polygenic prediction (e.g. schizophrenia vs. control)^1–8^. However, in clinical practice the most pressing challenge is often to distinguish related disorders from each other and from controls (e.g. schizophrenia (SCZ) vs. bipolar disorder (BIP) vs. depression (MDD) vs. control), especially at disorder onset when differential diagnosis can be challenging. A recent study proposed a method to distinguish inflammatory-arthritis causing diseases using genome-wide significant variants^9^, but this approach is unlikely to perform well for highly polygenic disorders such as psychiatric disorders^1,2,8,10–12^. We emphasize that very limited laboratory or biomarker tests are currently available to aid differential diagnosis in psychiatry^13^, providing a strong motivation for genetic testing approaches.

Here, we introduce a new method, Differential Diagnosis-Polygenic Risk Score (DDx-PRS), to distinguish related disorders from each other and from controls. DDx-PRS models the variance/covariance structure of disorder liabilities^14^ and polygenic risk scores (PRS) across disorders, leveraging case-control PRS computed using all genome-wide variants^3,5–7^. DDx-PRS uses prior disorder probabilities (which can be flexibly specified by a clinician) and applies Bayes’ Theorem to compute posterior probabilities of each diagnostic/other category (e.g. SCZ=50%, BIP=25%, MDD=15%, control=10%; note that control is defined as the absence of SCZ, BIP and MDD; we use the abbreviation “diagnostic category” in lieu of “diagnostic/other category” throughout the manuscript; we focus here on predicting SCZ/BIP/MDD, even though these diagnoses collapse and underrepresent the diagnostic categories available, because they have large training sample sizes and often represent diagnostic challenges). We apply DDx-PRS to predict SCZ vs. BIP vs. MDD vs. control in simulated data and in empirical data from the Psychiatric Genomics Consortium^8,15–17^. We also apply DDx-PRS to simulated data at larger training sample sizes to obtain projections of future clinical utility.

## Results

### Overview of methods

DDx-PRS distinguishes cases from clinically related disorders (e.g. SCZ vs. BIP vs. MDD vs. control), estimating posterior probabilities of each diagnostic category using case-control GWAS summary statistics for each disorder (as training data) and prior clinical probabilities (e.g. a unform prior of 25% for each diagnostic category). We have publicly released open-source software implementing DDx-PRS (see Code Availability). DDx-PRS has low computational cost, excluding the time required to compute case-control PRS (see Methods).

DDx-PRS consists of 4 steps (see Methods). In step 1, DDx-PRS estimates prior probabilities of each possible configuration of liabilities, relying on the fact that, for *n* disorders, there exist 2^*n*^ possible configurations of liabilities (above or below the liability threshold for each disorder^14^), e.g. 8 possible configurations for 3 disorders; the mapping from configurations of liabilities to diagnostic categories is informed by the PGC classifications used in ref. ^8,15–17^ (Table 1). In step 2, DDx-PRS computes case-control PRS for each disorder using an existing method (in this paper, we use PRS-CS^5^) transformed to their respective liability scales^18^. In step 3, DDx-PRS estimates truncated multivariate normal probability densities of liabilities and case-control PRS for each configuration of liabilities, using cross-trait LDSC^19^ and analytical derivations; DDx-PRS accounts for sample overlap across GWAS training data^19,20^ by estimating the covariances of the PRSs in an external reference panel (e.g. 1000 Genomes^21^). In step 4, DDx-PRS applies Bayes’ Theorem to estimate posterior probabilities of each configuration of liabilities for each test sample, conditional on their case-control PRS:

Eq.1 $$P(c_{j}|{\vec{PRS}}_{i})=P({\vec{PRS}}_{i}|c_{j})P(c_{j})/P({\vec{PRS}}_{i})$$

where *c*_*j*_ denotes configuration *j* and ${\vec{PRS}}_{i}$ denotes the case-control PRS for each disorder of individual *i*. The posterior probabilities of each configuration of liabilities are subsequently combined into posterior probabilities of each diagnostic category using the information in Table 1.

We compare the results of DDx-PRS to the results of a simpler method, Marginal-PRS (Table 2). Briefly, Marginal-PRS consists of 3 steps: (1) compute the marginal disorder probabilities *P*(*SCZ*), *P*(*BIP*) and *P*(*MDD*) using univariate densities for each disorder separately^18^; (2) compute the control probability as *P*(*control*) = (1 − *P*(*SCZ*))(1 − *P*(*BIP*))(1 − *P*(*MDD*)); and (3) rescale these four probabilities to add up to 1.

DDx-PRS and Marginal-PRS do not require additional tuning data to train parameters connecting case-control PRS to probabilities of each diagnostic category. Although methods that do not require tuning data are more practical, for completeness we also considered two methods that do require tuning data: DDx-PRS-tuned and Direct-tuned (Table 2 and Methods). DDx-PRS-tuned uses a tuning dataset to estimate the multivariate probability densities of $\vec{PRS}$ in step 2 above more directly (while still modelling the eight configurations *c*_*j*_). Direct-tuned applies Bayes’ Theorem as in Eq. 1 to the four diagnostic categories directly (i.e. using four diagnostic categories instead of the eight configurations in Table 1), estimating the multivariate probability densities of $\vec{PRS}$ in these four categories directly from the tuning data.

We assessed the performance of each method in classifying each diagnostic category vs. the rest (SCZ vs. rest, BIP vs. rest, MDD vs. rest, and control vs. rest). Calibration was assessed using the Integrated Calibration Index (ICI)^22^, defined as the weighted absolute difference between the predicted probability and the true probability, reflecting the average error of the predicted probabilities (ICI=0 reflects perfect calibration). Prediction accuracy was assessed using three metrics: area under the ROC curve (AUC)^23^ for each diagnostic category vs. rest, multi-class Matthews Correlation Coefficient (MCC)^24^, and Accuracy of most likely diagnosis (the proportion of the time that the most likely diagnosis matches the true diagnosis). We also estimate contingency tables.

We evaluated the performance of each method using data from the SCZ, BIP and MDD working groups of the Psychiatric Genomics Consortium (PGC; see Data Availability)^25^. The full PGC data that we analyzed consisted of the European-ancestry case-control GWAS summary statistics for SCZ^8^ (*N*=53,386 cases and 77,258 controls), BIP^15^ (*N*=41,917 cases and 371,549 controls), and MDD^16,17^ (*N*=173,140 cases and 331,433 controls) (Methods and Supplementary Table 1). The test data consisted of merged PGC case-control cohorts comprising 11,460 individuals of European ancestry (2,865 of each of SCZ, BIP, MDD, control), subdivided into 4 test cohorts within which SCZ, BIP, MDD, and control samples were matched with respect to country and genotyping platform (Methods and Supplementary Table 2). All PRS were based on independent training data (see Methods).

### Simulations

We simulated data based on the genetic architectures of SCZ^8^, BIP^15^ and MDD^16,17^, with liability-scale SNP-heritabilities^26^ of 0.24, 0.19 and 0.09, population prevalences of 1%, 2% and 16%, liability-scale case-control PRS *r*^2^ (ref.^27^) of 0.10, 0.09 and 0.04, respectively, and genetic correlations of 0.70 for SCZ-BIP, 0.35 for SCZ-MDD and 0.45 for BIP-MDD (collectively matching our empirical findings; see Methods). We simulated individual-level data for 1,000 SNPs (of which 50% were causal) in training samples whose sample size was selected to attain the specified case-control PRS *r*^2^ and 1,000 test samples for each diagnostic category (SCZ, BIP, MDD, controls). We introduced sample overlap of controls in SCZ, BIP and MDD training data to mimic empirical data, which includes substantial overlap of controls^20^. We analyzed 50 simulation replicates for each simulation.

**Figure 1:**
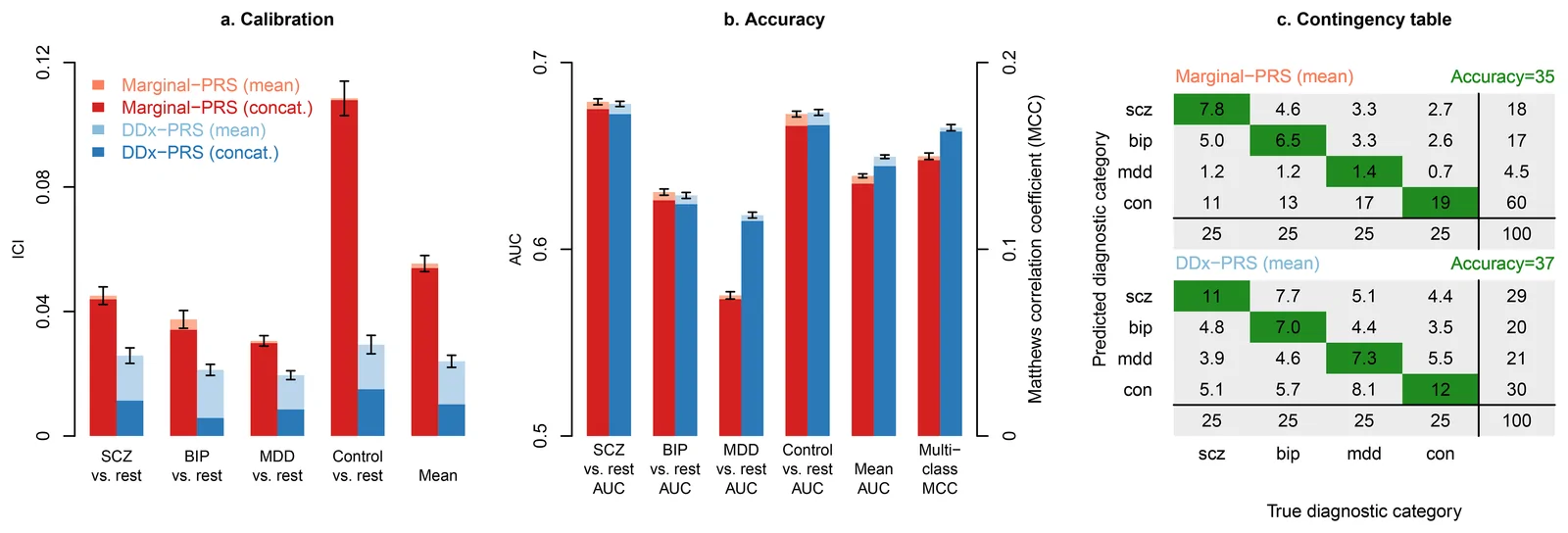
Calibration and accuracy of DDx-PRS and Marginal-PRS in simulations. We report the calibration (Integrated Calibration Index; ICI) **(Panel a)** and AUC (area under the ROC curve) **(Panel b)** for the comparisons of schizophrenia (SCZ) vs. rest, bipolar disorder (BIP) vs. rest, major depressive disorder (MDD) vs. rest, control vs. rest and the mean across these four comparisons, for two methods: Marginal-PRS (red) and DDx-PRS (blue). We also report the multi-class Matthews Correlation Coefficient (MCC) **(Panel b)**, contingency tables and Accuracy of most likely diagnosis **(Panel c)**. Results are based on 50 simulation replicates; mean values are displayed in light red and light blue, and results based on data concatenated across 50 replicates are displayed in dark red and dark blue. Error bars denote standard errors. The mean and concatenated contingency tables and Accuracy of most likely diagnosis are the same (the weighted average of proportions is equal to the proportion in concatenated data); all SE in contingency tables were <1.5%. Numerical results are reported in Supplementary Table 3.

**Figure 2:**
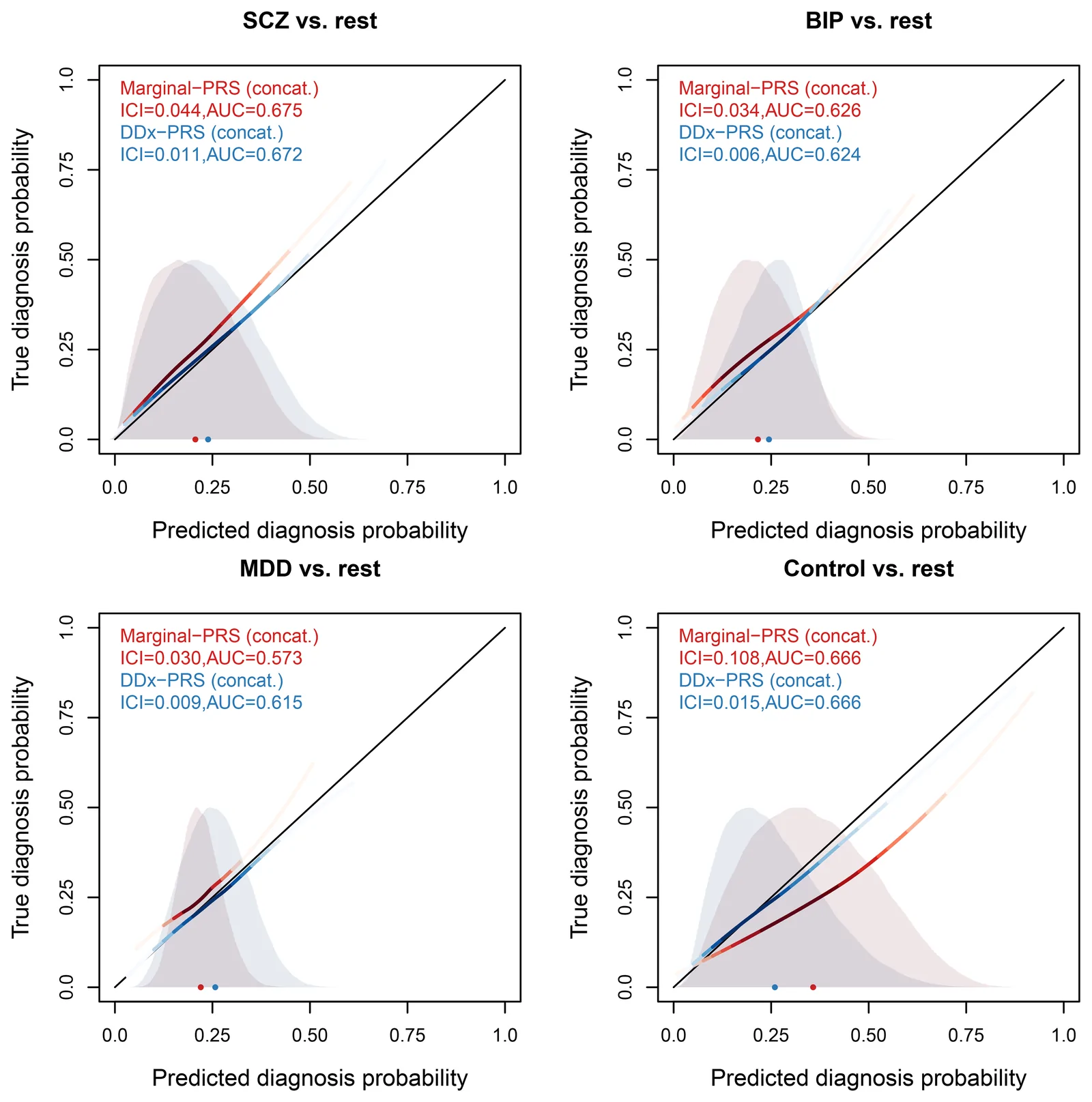
Calibration curves of DDx-PRS and Marginal-PRS in simulations. We report the true diagnosis probability vs. the predicted diagnosis probability for the comparisons of schizophrenia (SCZ) vs. rest, bipolar disorder (BIP) vs. rest, major depressive disorder (MDD) vs. rest, and control vs. rest, for two methods: Marginal-PRS (red) and DDx-PRS (blue). Results are based on concatenated data across 50 simulation replicates. The calibration curves are plotted using a locally estimated scatterplot smoothing (loess)-based smoothing function. The Integrated Calibration Index (ICI) equals the average absolute difference between the calibration curve and the line y=x (plotted in black), weighted by the density of the predicted diagnosis probabilities (displayed in the shaded histograms and in the color-intensity of the calibration curves). AUC, area under the ROC curve.

The calibration of DDx-PRS and Marginal-PRS are reported in Figure 1a, Figure 2 and Supplementary Table 3. DDx-PRS was well-calibrated with a mean ICI (±SE) of 0.024±0.002 (averaged across the four comparisons), whereas Marginal-PRS was substantially less well-calibrated with a mean ICI of 0.055±0.003. Marginal-PRS suffered particularly poor calibration in the comparison of control vs. rest (mean ICI of 0.108±0.006) because Marginal-PRS systematically underestimates the three disorder probabilities and overestimates the control probability (Figure 2). In addition to computing the mean ICI across simulation replicates, we computed concatenated ICI for simulated data that was concatenated across simulation replicates (Figure 1a and Supplementary Table 3). The concatenated ICI (averaged across the four comparisons) was much lower than the mean ICI for DDx-PRS (0.010 vs. 0.024), but only very slightly smaller for Marginal-PRS (0.054 vs. 0.055), which we interpret as follows. The ICI reflects prediction error due to both noise and method miscalibration, and the concatenated ICI reduces the noise (most important for DDx-PRS) but not the method miscalibration (most important for Marginal-PRS). The absolute difference between the mean ICI and concatenated ICI is larger for DDx-PRS than for Marginal PRS, because noise for a well-calibrated method always increases the ICI whereas noise for a miscalibrated method can either increase or decrease the ICI.

The prediction accuracy of DDx-PRS and Marginal-PRS are reported in Figure 1b and Supplementary Table 3. The AUC of DDx-PRS averaged across the four comparisons (mean AUC of 0.650±0.001) was slightly higher than the AUC of Marginal-PRS (average AUC of 0.639±0.001). The concatenated AUC (averaged across the four comparisons) were slightly smaller than the mean AUC (0.645 vs. 0.650 for DDx-PRS and 0.635 vs. 0.639 for Marginal-PRS), likely due to very slight heterogeneity across simulation replicates (see Methods). Consistent with the AUC results, the multi-class Matthews Correlation Coefficient (MCC) for DDx-PRS (0.165±0.002) was slightly higher than for Marginal-PRS (0.150±0.002), and the concatenated MCC were slightly smaller than the mean MCC (0.163 vs. 0.165 for DDx-PRS and 0.148 vs. 0.150 for Marginal-PRS). The contingency tables of DDx-PRS and Marginal-PRS are reported in Figure 1c and Supplementary Table 3. Consistent with the AUC and MCC results, the Accuracy of most likely diagnosis for DDx-PRS (0.372±0.001) was slightly higher than for Marginal-PRS (0.347±0.002). The concatenated Accuracy of most likely diagnosis results were identical to the mean results (the weighted average of proportions is equal to the proportion in concatenated data). We note that the MCC and contingency table discretises the predicted probabilities of each diagnosis to the most likely diagnosis, which loses information.

As an additional point of reference, we also compared the accuracy of DDx-PRS in case vs. rest comparisons to the accuracy of DDx-PRS in case vs. control comparisons (Supplementary Table 4). We anticipated that distinguishing case vs. rest would be a harder problem than distinguishing case vs. control, given the positive genetic correlations between disorders. Indeed, case vs. rest accuracy was lower than case vs. control accuracy for SCZ (AUC of 0.678±0.001 for SCZ vs. rest, 0.745±0.002 for SCZ vs. control) and BIP (0.629±0.002 for BIP vs. rest, 0.701±0.002 for BIP vs. control)—but slightly higher than case vs. control accuracy for MDD (0.618±0.002 for MDD vs. rest, 0.610±0.002 for MDD vs. control), because MDD cases are genetically more similar to controls than to SCZ cases and BIP cases (Figure 1 of ref.^20^). We discuss secondary simulation results in the Supplementary Note, Supplementary Tables 5-9 and Supplementary Figures 1-7.

### DDx-PRS distinguishes SCZ, BIP, MDD and controls in PGC data

We applied DDx-PRS to distinguish 4 diagnostic categories (SCZ, BIP, MDD, control) in data from the Psychiatric Genomics Consortium (PGC). We note that there is no laboratory gold standard for validating psychiatric diagnoses, and that current DSM/ICD classifications are not pathophysiological entities^28–31^; despite these limitations, the predictive value of genetic effects has consistently been confirmed in fully independent samples across different recruitment settings^8,15–17^. We also note that if the PGC classifications only partially capture true neurobiological differences, genetic predictions will still capture these neurobiological differences, albeit with reduced accuracy.

The full PGC data that we analyzed consisted of European-ancestry case-control GWAS summary statistics for SCZ^8^ (*N*=53,386 cases and 77,258 controls), BIP^15^ (*N*=41,917 cases and 371,549 controls), and MDD^16,17^ (*N*=173,140 cases and 331,433 controls) (Methods and Supplementary Table 1). The test data consisted of merged PGC case-control cohorts comprising 11,460 individuals of European ancestry (2,865 of each of SCZ, BIP, MDD, control), subdivided into 4 test cohorts within which SCZ, BIP, MDD, and control samples were matched with respect to country and genotyping platform (GER (N=3,368), UK1 (N=1,136), UK2 (N=6,080), USA (N=876); see Methods and Supplementary Table 2). We computed standard errors (SE) of our results based on variation across our four test cohorts, while noting that this does not account for variation in the training data.

**Figure 3:**
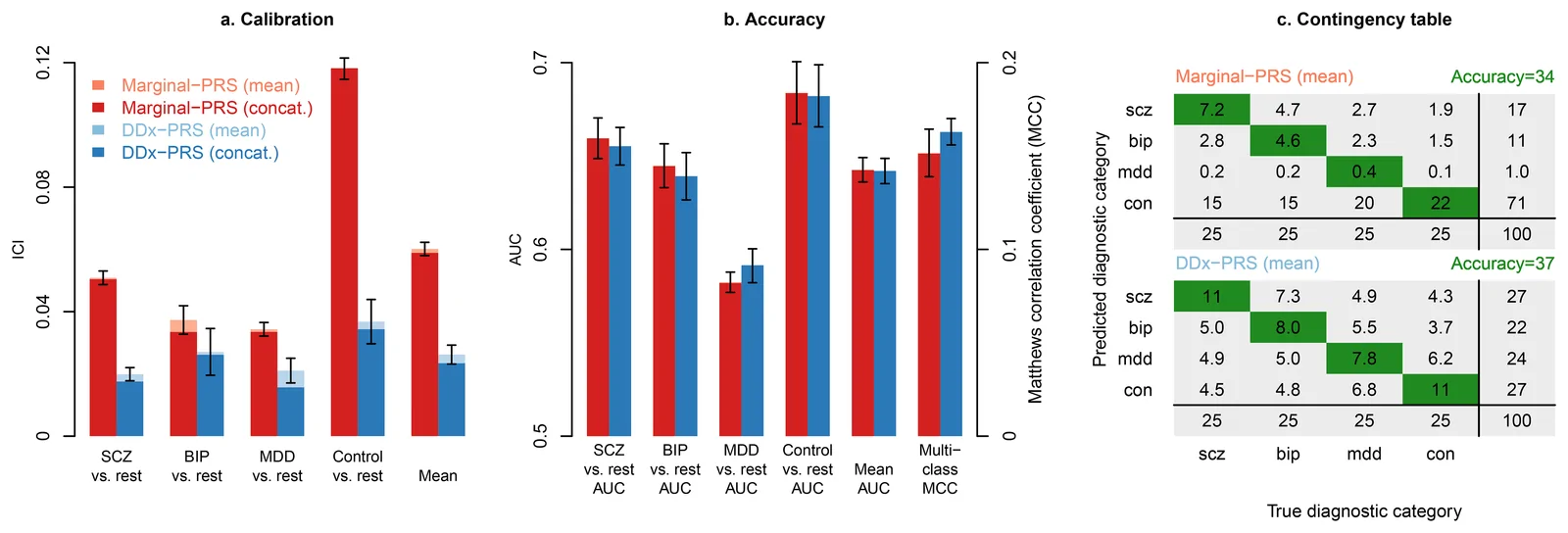
Calibration and accuracy of DDx-PRS and Marginal-PRS in PGC data. We report the calibration (Integrated Calibration Index; ICI) **(Panel a)** and AUC (area under the ROC curve) **(Panel b)** for the comparisons of schizophrenia (SCZ) vs. rest, bipolar disorder (BIP) vs. rest, major depressive disorder (MDD) vs. rest, control vs. rest and the mean across these four comparisons, for two methods: Marginal-PRS (red) and DDx-PRS (blue). We also report the multi-class Matthews Correlation Coefficient (MCC) **(Panel b)**, contingency tables and Accuracy of most likely diagnosis **(Panel c)**. The training data consisted of case-control GWAS summary statistics for SCZ, BIP and MDD; the test data consisted of independent PGC samples subdivided in four test cohorts that were matched with respect to country and genotyping platform (total N=11,460, with 2,865 for each of SCZ, BIP, MDD and control). The mean values across these four test cohorts are displayed in light red and light blue, and results based on data concatenated across these four test cohorts are displayed in dark red and dark blue. Error bars denote standard errors. The mean and concatenated contingency tables and Accuracy of most likely diagnosis are the same (the weighted average of proportions is equal to the proportion in concatenated data); all SE in contingency tables were <1.7%. Numerical results are reported in Supplementary Table 10.

**Figure 4:**
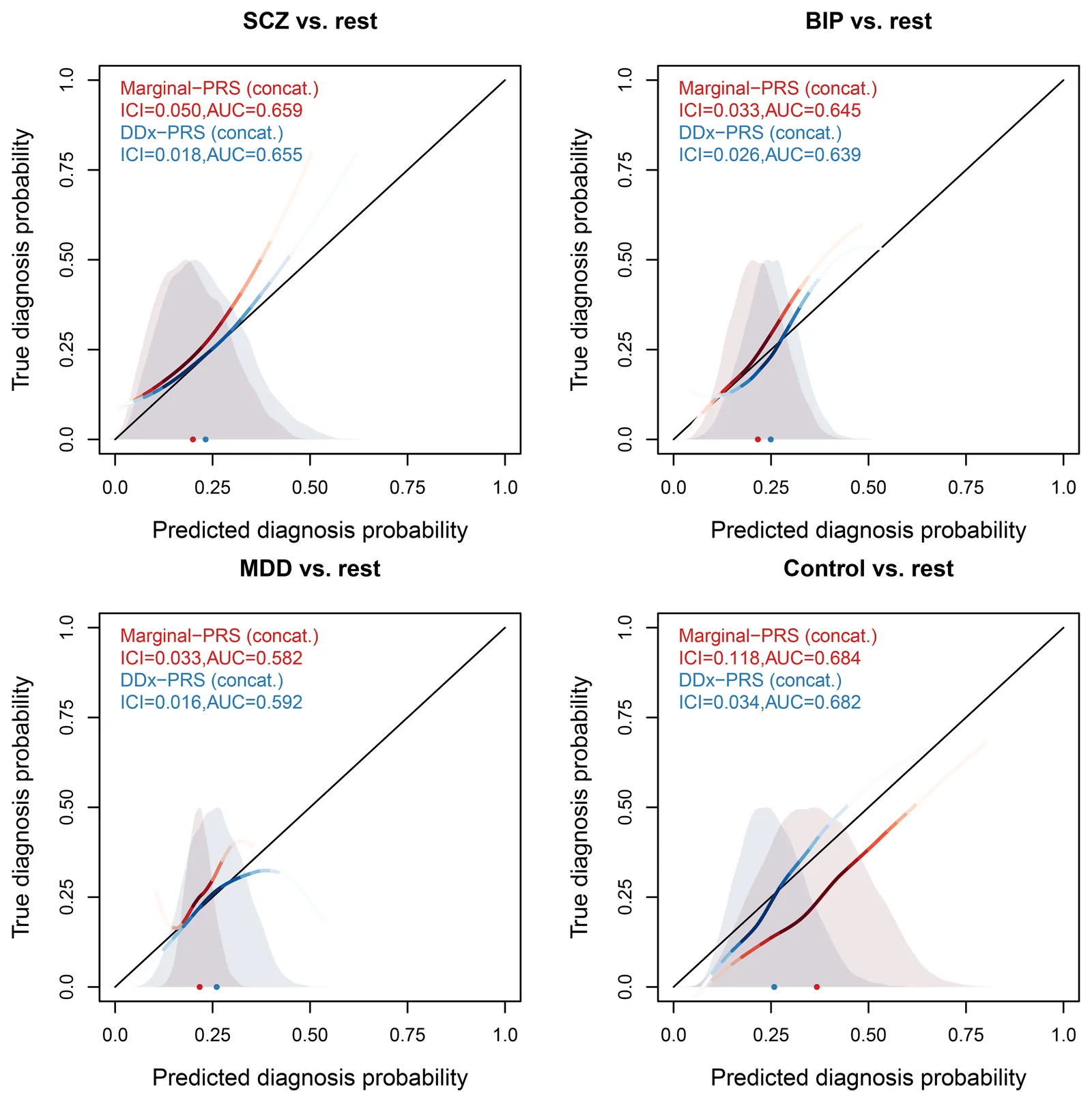
Calibration curves of DDx-PRS and Marginal-PRS in PGC data. We report the true diagnosis probability vs. the predicted diagnosis probability for the comparisons of schizophrenia (SCZ) vs. rest, bipolar disorder (BIP) vs. rest, major depressive disorder (MDD) vs. rest, and control vs. rest, for two methods: Marginal-PRS (red) and DDx-PRS (blue). The calibration curves are plotted using a locally estimated scatterplot smoothing (loess)-based smoothing function. Results are based on the concatenated data across the four PGC test cohorts (total N=11,460, with 2,865 for each of SCZ, BIP, MDD and control). The Integrated Calibration Index (ICI) equals the average absolute difference between the calibration curve and the line y=x (plotted in black), weighted by the density of the predicted diagnosis probabilities (displayed in the shaded histograms and in the color-intensity of the calibration curves). AUC, area under the ROC curve.

The calibration of DDx-PRS and Marginal-PRS are reported in Figure 3a and Supplementary Table 10. DDx-PRS was well-calibrated with a mean ICI (±SE) of 0.026±0.003 (averaged across the four comparisons), whereas Marginal-PRS was substantially less well-calibrated with a mean ICI of 0.060±0.003. Analogous to simulations (Figure 1a), Marginal-PRS suffered particularly poor calibration in the comparison of control vs. rest (ICI of 0.118±0.003), because Marginal-PRS systematically underestimates the three disorder probabilities and overestimates the control probability (Figure 4). We also computed concatenated ICI for PGC data that was concatenated across the 4 test cohorts (Figure 3a and Supplementary Table 10). The concatenated ICI (averaged across the four comparisons) was slightly smaller than the mean ICI for DDx-PRS (0.023 vs. 0.026), likely because the concatenated results were slightly less noisy due to 4 times larger test sample size. However, the concatenated ICI was only very slightly smaller than the mean ICI for Marginal-PRS (0.059 vs. 0.060), analogous to simulations.

The prediction accuracy of DDx-PRS and Marginal-PRS are reported in Figure 3b and Supplementary Table 10. DDx-PRS attained similar AUC (mean AUC of 0.642±0.007) as Marginal-PRS (mean AUC of 0.643±0.007). Results for concatenated AUC (concatenated across the 4 test cohorts) were very similar to results for mean AUC. The MCC for DDx-PRS (0.163±0.007) was slightly higher than for Marginal-PRS (0.152±0.013), and the concatenated MCC were very similar to the mean MCC. The contingency tables of DDx-PRS and Marginal-PRS are reported in Figure 3c and Supplementary Table 10. The Accuracy of most likely diagnosis for DDx-PRS (0.372±0.005) was slightly higher than for Marginal-PRS (0.338±0.005). The concatenated Accuracy of most likely diagnosis results were identical to the mean results (the weighted average of proportions is equal to the proportion in concatenated data). We also compared the accuracy of DDx-PRS in case vs. rest comparisons to the accuracy of DDx-PRS in case vs. control comparisons (Supplementary Table 11). Case vs. rest accuracy was lower than case vs. control accuracy for SCZ (AUC of 0.655±0.010 for SCZ vs. rest, 0.728±0.016 for SCZ vs. control), BIP (0.639±0.013 for BIP vs. rest, 0.720±0.013 for BIP vs. control), and MDD (0.591±0.009 for MDD vs. rest, 0.618±0.017 for MDD vs. control; non-significant difference), similar to results in simulations. We discuss secondary analyses in the Supplementary Note, Supplementary Tables 12-14 and Supplementary Figures 8-12.

### Comparison of DDx-PRS to methods that require tuning data

An advantage of DDx-PRS is that it does not require additional tuning data to train parameters connecting case-control PRS to probabilities of each diagnostic category, offering practical advantages for implementation. However, for completeness, we also compared the performance of DDx-PRS to two methods that do require tuning data: DDx-PRS-tuned and Direct-tuned (Table 2 and Methods). We primarily focused on cross-cohort tuning, in which the tuning data set is from a different cohort than test data (but we also report results for within-cohort tuning). For every possible choice of tuning cohort and test cohort in PGC data (4×3=12 analyses, as we analysed four tuning/test cohorts; Supplementary Table 2), we restricted tuning data to 200 samples from each of the four diagnostic categories (total *N*_tuning_=800) and evaluated the performance of each method in test data.

**Figure 5:**
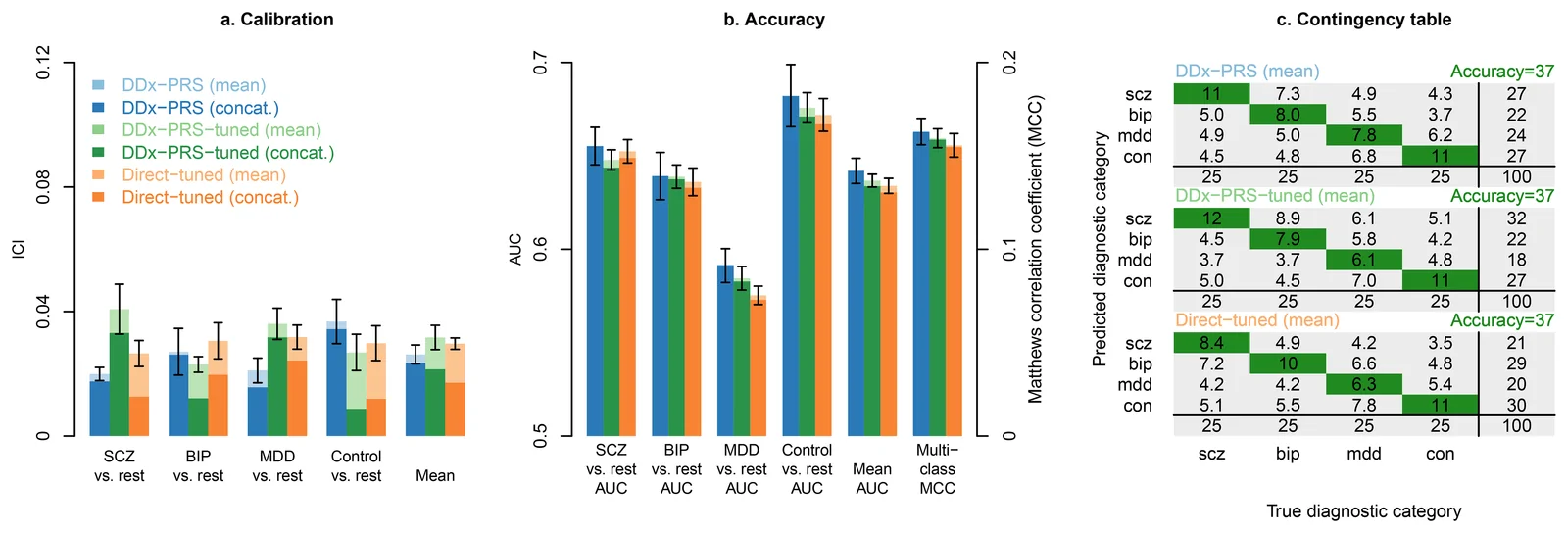
Calibration and accuracy of DDx-PRS, DDx-PRS-tuned and Direct-tuned in PGC data. We report the calibration (Integrated Calibration Index; ICI) **(Panel a)** and AUC (area under the ROC curve) **(Panel b)** for the comparisons of schizophrenia (SCZ) vs. rest, bipolar disorder (BIP) vs. rest, major depressive disorder (MDD) vs. rest, control vs. rest and the mean across these four comparisons, for three methods: DDx-PRS (blue), DDx-PRS-tuned (green) and Direct-tuned (orange). We also report the multi-class Matthews Correlation Coefficient (MCC) **(Panel b)**, contingency tables and Accuracy of most likely diagnosis **(Panel c)**. The training data consisted of case-control GWAS summary statistics for SCZ, BIP and MDD; the test data consisted of independent PGC samples subdivided in four test cohorts that were matched with respect to country and genotyping platform (total N=11,460, with 2,865 for each of SCZ, BIP, MDD and control). For tuning for DDx-PRS-tuned and Direct-tuned, 4×3=12 tuning/test cohort-pairs were analysed with tuning data restricted to 200 samples from each of SCZ, BIP, MDD and control (total *N*_tuning_=800). The mean values across these four test cohorts (DDx-PRS) resp. 12 tuning/test cohort-pairs (DDx-PRS-tuned and Direct-tuned) are displayed in light blue, light green and light orange, and results based on data concatenated across these four test cohorts (resp. 12 tuning/test cohort-pairs) are displayed in dark blue, dark green and dark orange. Error bars denote standard errors. The mean and concatenated contingency tables and Accuracy of most likely diagnosis are the same (the weighted average of proportions is equal to the proportion in concatenated data); all SE in contingency tables were <2.4%. Numerical results are reported in Supplementary Table 15.

The calibration of DDx-PRS, DDx-PRS-tuned and Direct-tuned are reported in Figure 5a and Supplementary Table 15. The three methods attained similar calibration with mean ICIs (±SE) averaged across the four comparisons of 0.026±0.002, 0.032±0.004, and 0.030±0.002 respectively (Figure 5a and Supplementary Table 15). A comparison of true vs. predicted probabilities further illustrated that the three methods had similar calibration (Extended Data Figure 1). We also computed concatenated ICI for PGC data that was concatenated across 4 test cohorts for DDx-PRS (which does not use tuning data) or 12 pairs of tuning-test cohorts for DDx-PRS-tuned and Direct-tuned. The concatenated ICI (averaged across the four comparisons) were slightly smaller than the mean ICI, analogous to Figure 3a.

The prediction accuracy of DDx-PRS, DDx-PRS-tuned and Direct-tuned are reported in Figure 5b and Supplementary Table 15. The three methods attained comparable AUC with mean AUCs (±SE) of 0.0642±0.0039, 0.0637±0.0034, and 0.0634±0.0040, respectively. Results for concatenated AUC were very similar to results for mean AUC, analogous to Figure 3b. The three methods also attained comparable MCC of 0.163±0.007, 0.159±0.005, and 0.163±0.005, respectively. The concatenated MCC were very similar to the mean MCC.

The contingency tables of DDx-PRS, DDx-PRS-tuned and Direct-tuned are reported in Figure 5c and Supplementary Table 15. DDx-PRS, DDx-PRS-tuned and Direct-tuned attained comparable Accuracy of most likely diagnosis with values of 0.372±0.003, 0.368±0.004, and 0.365±0.005, respectively. The concatenated Accuracy of most likely diagnosis results were identical to the mean results (the weighted average of proportions is equal to the proportion in concatenated data). Overall patterns in the contingency tables were very similar for DDx-PRS, DDx-PRS-tuned and Direct-tuned. We discuss secondary analyses in the Supplementary Note, Supplementary Tables 16-18 and Supplementary Figures 13-16.

We conclude that DDx-PRS produces comparable results to DDx-PRS-tuned and Direct-tuned; thus, DDx-PRS is likely to be preferred in most settings (as it does not require tuning data).

### Projections of clinical utility at larger training sample sizes

We sought to assess the clinical utility of DDx-PRS in PGC data and in projections to larger training sample sizes. For each diagnostic category (SCZ, BIP, MDD, control), we computed the true diagnosis probability in each decile of predicted diagnosis probability (similar to previous studies of clinical utility^32–35^).

**Figure 6:**
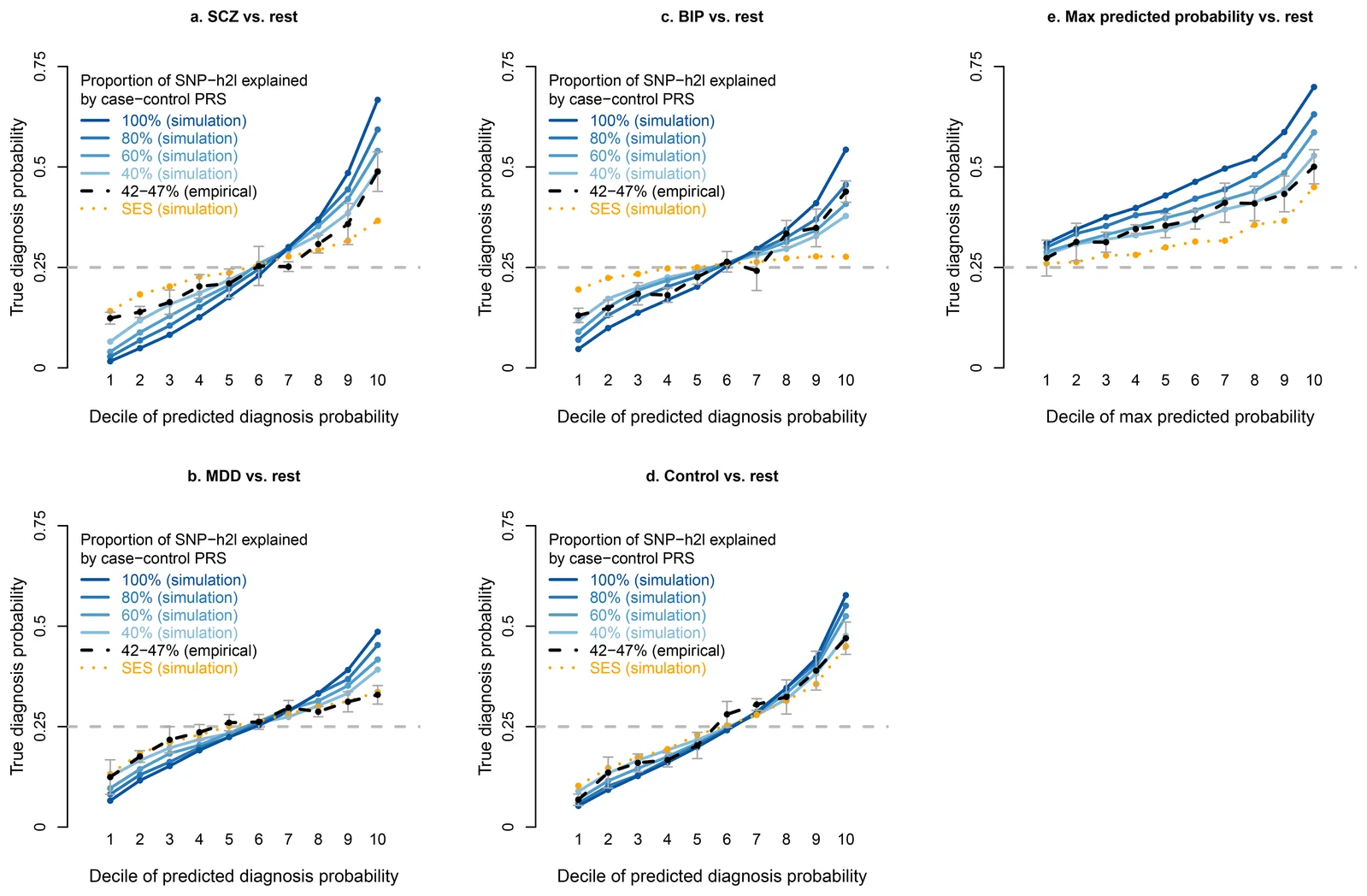
Projections of clinical utility at larger training sample sizes. We report the true diagnosis probability per decile of predicted diagnosis probability for the comparisons of schizophrenia (SCZ) vs. rest, bipolar disorder (BIP) vs. rest, major depressive disorder (MDD) vs. rest, control vs. rest, and maximum (max) predicted probability vs. rest, for DDx-PRS. Empirical results in PGC data are reported as black points (error bars in grey) connected with dashed black lines; error bars denote 1.96 times the SE around the mean across the four PGC test cohorts. Projections (simulations) at various values of SNP-heritability captured by case-control PRS (40%, 60%, 80% and 100%) are reported as blue points connected with solid blue lines, with varying colour intensity; these results represent the mean across 50 simulations runs (1.96*SE is reported in Supplementary Table 19). We also report simulated projections to compare the ability of PRS to distinguish diagnostic categories with a common non-genetic risk factor (in this example SES); these results are based on a single simulation run in a very large population of N=5,000,000. The baseline diagnosis probability (i.e. prior clinical probability) equals 0.25 for each of SCZ, BIP, MDD and control (dashed grey horizontal lines). We note that the true diagnosis probability for max predicted probability vs. rest corresponds to Accuracy of most likely diagnosis. PGC results for DDx-PRS-tuned and Direct-tuned are reported in Supplementary Figure 17 and Supplementary Table 21. Numerical results, including error bars for all data points, are reported in Supplementary Table 19. The values of the predicted diagnosis probabilities are reported in Supplementary Table 20.

Results for PGC data are reported in Figure 6 (dashed black curve) and Supplementary Table 19. The top decile of predicted diagnosis probability yielded true diagnosis probabilities of 0.49 for SCZ, 0.44 for BIP, 0.33 for MDD and 0.47 for controls (close to the predicted diagnosis probabilities; Supplementary Table 20), substantially above baseline probabilities of 0.25.

We computed projections to larger training sample sizes by directly simulating case-control PRS (instead of simulating individual-level SNP data; Figure 1 and Figure 2) at different values of % of SNP-heritability captured by case-control PRS, ranging from 40% (similar to PGC data; 42-47%) to 100% (a best-case scenario, but already approximately attained for height^36^) (Methods). Results for projections to larger training samples sizes are reported in Figure 6 (solid blue curves) and Supplementary Table 19. In simulations at SNP-heritability captured by case-control PRS equal to 100%, the top decile of predicted diagnosis probability yielded true diagnosis probabilities of 0.67 for SCZ, 0.54 for BIP, 0.48 for MDD and 0.58 for controls, implying appreciable potential for clinical utility.

We also computed the true diagnosis probability in each decile of maximum predicted probability (i.e., the Accuracy of most likely diagnosis; Figure 6e). In analyses of the PGC data, the Accuracy of the most likely diagnosis was 50% for the top decile and >41% for each of the top 3 deciles. In simulations at SNP-heritability captured by case-control PRS equal to 100%, the Accuracy of the most likely diagnosis was 70% for the top decile and >52% for each of the top 3 deciles. Thus, DDx-PRS provides appreciable potential for clinical utility at larger training sample-sizes under certain circumstances (see Discussion).

To contextualize our projections of clinical utility at larger training sample sizes, we compared the diagnostic accuracy of DDx-PRS to the projected diagnostic accuracy of socio-economic status (SES) based on simulations (no phenotypic SES data is available in our samples), by assuming a correlation of 0.21, 0.07, 0.36 between SES and the liabilities of SCZ, BIP, MDD respectively (with SES assessed via the Townsend deprivation index; see Methods)^37^. Compared to our findings in PGC data, we observed that projections of SES are expected to have lower power to distinguish SCZ vs. rest and BIP vs. rest, and similar power to distinguish MDD vs. rest and control vs rest (Figure 6 and Supplementary Table 19); the limited expected predictive power of SES to distinguish SCZ/BIP/MDD/control is consistent with the fact that SES provides a one-dimensional predictor for a multi-class outcome. We discuss secondary analyses in the Supplementary Note, Supplementary Tables 21-22 and Supplementary Figures 17-20.

## Discussion

Differential diagnosis is a common clinical challenge, specifically for disorders that are phenotypically (and genetically) similar; in particular, psychiatric disorders often present with non-specific symptoms at disorder onset. Here, we developed a new method, DDx-PRS, to distinguish related disorders from each other as well as controls. We determined that DDx-PRS is well-calibrated and well-powered based on the training sample sizes analysed, outperforming a simpler method (Marginal-PRS) in both simulated data and empirical PGC data. A strength of DDx-PRS is that is does not use additional tuning data to train parameters connecting case-control PRS to probabilities of each diagnostic category. When comparing DDx-PRS to methods that do require additional tuning data (DDx-PRS-tuned and Direct-tuned), we determined that DDx-PRS produced comparable results, confirming that DDx-PRS is an effective method that is likely to be preferred in most settings as it does not require tuning data. We also assessed the clinical utility of DDx-PRS in projections to larger training sample sizes, indicating appreciable potential for future clinical utility under certain circumstances (see below).

We are currently aware of only one previous method for distinguishing different disorders using genetic data (G-PROB)^9^. Unlike DDx-PRS, G-PROB uses only genome-wide significant variants. This may be suitable for distinguishing inflammatory arthritis-causing diseases for which G-PROB was developed, as these diseases are relatively less polygenic^10,11^; however, this approach will have limited utility for highly polygenic disorders such as psychiatric disorders^38^ with high heterogeneity of current diagnostic classification and poorly understood biological underpinnings^28–31^, for which the contribution of genome-wide significant variants is limited^1,2,8,10,11^. We note that G-PROB is similar to Marginal-PRS in that the disorder probabilities are computed separately for every diagnostic category and later combined into a multi-diagnostic outcome; thus, G-PROB lacks the advantages of DDx-PRS obtained by modeling the variance/covariance structure across disorders. Finally, G-PROB aims to only distinguish different disorders (for which DDx-PRS can also be applied; Supplementary Figure 12) but does not additionally distinguish disorder cases from controls (our primary focus; Figure 3).

The potential clinical utility of DDx-PRS is subject to several important considerations. First, refining diagnostic probability for a diagnostic category in differential diagnosis can be clinically useful; for example, for SCZ/BIP/MDD/control, increasing probability from a prior baseline risk of 25% to genetically informed risk of ~50% may guide clinicians in selecting interventions such as preventive psychotherapy, targeted follow-up, and psychoeducation pertaining to risk factors and early symptoms, which can provide important benefits^39,40^. Second, case-control PRS explain less than 8% of variance in risk of SCZ in the population^8^ (6-13% using PRS-CS^5^; Supplementary Table 23), which limits the clinical utility of using PRS alone for decision making. Indeed, the potential clinical utility is likely restricted to individuals at moderate to high levels of disorder risk (e.g. *≥*25%) (Figure 6). Third, since the PGC samples are selected for genetic research, they may not represent real-world patients, and thus extensive validation and testing are needed before DDx-PRS can be applied in clinical settings. Fourth, ethical issues should carefully be considered^41–43^, e.g., not all individuals may wish to know their risk for SCZ/BIP/MDD. We note that DDx-PRS has potential for clinical utility in other settings than psychiatry, such as distinguishing inflammatory arthritis-causing diseases^9^, latent autoimmune diabetes vs. type 2 diabetes^44^, and asthma vs. COPD vs. asthma-COPD overlap syndrome^45^. An extended discussion of the potential clinical utility of DDx-PRS is provided in the Supplementary Note.

We note several limitations of this study. First, there is no laboratory gold standard for validating psychiatric diagnoses, and current DSM/ICD classifications are not pathophysiological entities^28–31^ (in contrast to e.g. inflammatory arthritis-causing diseases^9^), which could impact external validation. However, our results validated predictive performance in fully independent test samples within the PGC (Figure 3-6). We note that even if PGC classifications only partially capture true neurobiological differences, genetic predictions will still capture these neurobiological differences, albeit with reduced accuracy. Ultimately, future clinical intervention studies will be needed to establish clinical utility. Second, we chose to focus here on SCZ/BIP/MDD, because they have the largest available training sample sizes and often represent diagnostic challenges. However, relevant diagnostic distinctions often include additional disorders beyond SCZ/BIP/MDD, e.g. schizoaffective disorder (SZA) (which we considered as a subset of SCZ^8^). Similarly, there exist clinically-relevant subtypes of BIP (e.g., with/without psychosis) and MDD (e.g., with/without psychosis), which we do not consider here as these subtypes were pooled into single case groups in ref.^15^ and ref.^16,17^. We note that DDx-PRS can flexibly include additional disorders/subtypes in its prediction framework as GWAS training sample sizes increase. Additional limitations are discussed in Supplementary Note. Despite these limitations, DDx-PRS offers a novel and useful method for leveraging genomic information to inform differential diagnosis.

## Methods

### DDx-PRS method

DDx-PRS consists of 4 steps: (1) estimate prior probabilities of each possible configuration of liabilities; (2) compute case-control PRS; (3) estimate analytical variances and covariances across disorders of liabilities and case-control PRS (overall and for each configuration of liabilities); and (4) estimate posterior probabilities for each test sample.

In step 1, DDx-PRS relies on the fact that, for *n* disorders, there exist 2^*n*^ possible configurations of liabilities (above or below the liability threshold for each disorder^14^), e.g. 8 possible configurations for 3 disorders; the mapping from configurations of liabilities to diagnostic categories is informed by the PGC classifications used in ref. ^8,15–17^ (Table 1). Given prior clinical probabilities of each diagnostic category, DDx-PRS estimates prior probabilities of each configuration of liabilities by assuming random sampling of liabilities within each diagnostic category. We note that the prior clinical probability for help-seeking individuals will typically be much larger than the population prevalence. For simplicity, we assume a clinical prior probability of 25% for each of SCZ, BIP, MDD and control for the main analyses presented in this paper; however, in application of DDx-PRS, the prior can be specified flexibly by the physician based on e.g. psychiatric examination, questionnaires and/or disorder prevalence in a specific clinical setting.

In step 2, DDx-PRS computes case-control PRS for each disorder (on the observed scale) using an existing method (in this paper, we use PRS-CS^5^) and transforms the PRS to their respective liability scales^18^. Case-control PRS are computed for both the test samples and samples from an external reference panel such as 1000 Genomes^21^; the external reference panel does not include phenotypes, and as such does not constitute a tuning data set.

In step 3, DDx-PRS estimates (i) overall variances and covariances of liabilities for each disorder using cross-trait LDSC^19^, (ii) overall variances and covariances of liability-scale case-control PRS for each disorder using the external reference panel (e.g. 1000 Genomes^21^)—in particular, this accounts for sample overlap across controls for different disorders in GWAS training data increasing correlation between the case-control PRS^19,20^, and (iii) overall covariances between liabilities and case-control PRS, based on analytical computations. DDx-PRS then estimates truncated multivariate normal probability densities of liabilities and case-control PRS for each configuration of liabilities using analytical derivations.

In step 4, DDx-PRS applies Bayes’ Theorem to estimate posterior probabilities of each configuration of liabilities for each test sample, conditional on their case-control PRS. Letting *c*_*j*_ denote configuration *j* and ${\vec{PRS}}_{i}$denote the case-control PRS for each disorder of individual *i*, these posterior probabilities can be expressed as

Eq.2 $$P\left(c_{j}|{\vec{PRS}}_{i}\right)=P\left({\vec{PRS}}_{i}|c_{j}\right)P\left(c_{j}\right)/P\left({\vec{PRS}}_{i}\right)$$

where $P\left({\vec{PRS}}_{i}|c_{j}\right)$denotes the probability density of ${\vec{PRS}}_{i}$ given configuration *c*_*j*_ (computed from the output of step 3); *P*(*c*_*j*_) denotes the prior probability of configuration *c*_*j*_ (specified in step 1); and

$P\left({\vec{PRS}}_{i}\right)$denotes the probability density of ${\vec{PRS}}_{i}$ summed across all configurations weighted by the prior probabilities, i.e. $P({\vec{PRS}}_{i})=\sum_{j^{\prime }}P({\vec{PRS}}_{i}|c_{j^{\prime }})P(c_{j^{\prime }}).$ The posterior probabilities of each configuration of liabilities are subsequently combined into posterior probabilities of each diagnostic category using the information in Table 1.

Details of DDx-PRS are described in the Supplementary Note.

### Marginal-PRS method

We compare the results of DDx-PRS to the results of a simpler method, Marginal-PRS (Table 2). Briefly, Marginal-PRS consists of 3 steps: (1) compute the marginal disorder probabilities *P*(*SCZ*), *P*(*BIP*) and *P*(*MDD*) using univariate densities for each disorder separately^18^; (2) compute the control probability as *P*(*controI*) = (1 − *P*(*SCZ*))(1 − *P*(*BIP*))(1− *P*(*MDD*)); and (3) rescale these four probabilities to add up to 1. We note two important limitations of Marginal-PRS compared to DDx-PRS. First, Marginal-PRS does not model the covariance structure between the respective case-control PRS. Second, when computing marginal disorder probabilities, Marginal-PRS does not account for the other disorders (e.g., when computing *P*(*SCZ*), Marginal-PRS incorrectly assumes that non-SCZ individuals are all controls). Details of Marginal-PRS are described in the Supplementary Note.

### Methods that require tuning data: DDx-PRS-tuned & Direct-tuned

DDx-PRS and Marginal-PRS do not require additional tuning data to train parameters connecting case-control PRS to probabilities of each diagnostic category. We also considered two methods that do require tuning data: DDx-PRS-tuned and Direct-tuned (Table 2). The tuning data consists of individuals from the four diagnostic categories (SCZ, BIP, MDD and controls) with the case-control PRS for SCZ, BIP and MDD. The tuning data is completely independent from both the training data and the test data.

DDx-PRS-tuned consists of the same 4 steps as DDx-PRS: (1) estimate prior probabilities of each possible configuration of liabilities (identical to DDx-PRS); (2) compute case-control PRS in the tuning data and the testing data; (3) estimate analytical variances and covariances across disorders of liabilities and case-control PRS based on the tuning; and (4) estimate posterior probabilities for each test sample (identical to DDx-PRS). The tuning data is used to estimate the variance explained by the PRS, contrary to DDx-PRS approximating the variance explained based 1000G data (see Supplementary Note)^18^. Thus, compared to DDx-PRS, DDx-PRS-tuned takes away the uncertainty that comes from the assumptions made to approximate the case-control variance explained based on 1000G data^18^. Details of DDx-PRS-tuned are described in the Supplementary Note.

Direct-tuned uses a simpler and more direct approach than DDx-PRS-tuned. Direct-tuned consists of 4 steps: (1) specify prior probabilities of each diagnostic category (contrary to DDx-PRS, Direct-tuned does not estimate the prior probabilities of each possible configuration of liabilities; Table 1); (2) compute case-control PRS in the tuning data and in the testing data; (3) estimate the density across the case-control PRSs for the diagnostic categories; and (4) estimate posterior probabilities for each test sample using Bayes’ Theorem. Details of Direct-tuned are described in the Supplementary Note.

### Statistics - Outcome metrics

We assessed the performance of each method in classifying each diagnostic category vs. the rest (SCZ vs. rest, BIP vs. rest, MDD vs. rest, and control vs. rest). Calibration was assessed using the Integrated Calibration Index (ICI)^22^, defined as the weighted absolute difference between the predicted probability and the true probability. Briefly, the ICI is computed by estimating the true probabilities compared to the predicted probability using a locally estimated scatterplot smoothing (loess)-based smoothing function, and subsequently assessing the average absolute difference between the predicted and true probabilities, weighted by the density of predicted probabilities; it thus reflects the average error of the predicted probabilities (ICI=0 reflects perfect calibration). Prediction accuracy was assessed using three metrics: area under the ROC curve (AUC)^23^ for each diagnostic category vs. rest, multi-class Matthews Correlation Coefficient (MCC)^24^, and Accuracy of most likely diagnosis. MCC compares the most likely diagnostic category (i.e. the diagnostic category with highest predicted probability) to the true diagnostic category, and provides a multi-class correlation between actual and predicted classifications across multiple classes. Accuracy of most likely diagnosis is the proportion of the time that the most likely diagnosis matches the true diagnosis. We also estimate contingency tables (defined as the proportion of individuals with true diagnostic category X and predicted diagnostic category Y, for each choice of X and Y).

### Main simulations with individual-level SNP data

We simulated data based on the genetic architectures of SCZ^8^, BIP^15^ and MDD^16,17^, with liability-scale SNP-heritabilities^26^ of 0.24, 0.19 and 0.09, population prevalences of 1%, 2% and 16%, liability-scale case-control PRS *r*^2^ (ref.^27^) of 0.10, 0.09 and 0.04, respectively, and genetic correlations of 0.70 for SCZ-BIP, 0.35 for SCZ-MDD and 0.45 for BIP-MDD (collectively matching our empirical findings; see Methods). We simulated individual-level data for 1,000 SNPs (of which 50% were causal) in training samples whose sample size was selected to attain the specified case-control PRS *r*^2^ and 1,000 test samples for each diagnostic category (SCZ, BIP, MDD, controls). We introduced sample overlap of controls in SCZ, BIP and MDD training data to mimic empirical data, which includes substantial overlap of controls^20^. We simulated a small number of SNPs (*M*) and training samples (*N*) to limit computational cost, while noting that PRS *r*^2^ primarily depends on *M*/*N*, so that simulations at reduced values of both *M* and *N* are appropriate^3^. Because linkage disequilibrium (LD) does not impact cross-disorder architectures conditional on the above parameter values, we simulated genotypes without LD. Case-control PRS were computed using Bpred, which analytically computes posterior mean causal effects sizes under a point-normal prior in the special case of no LD^3^. We simulated individual-level SNP data (instead of directly simulating case-control PRS values in test samples) to also assess the performance of the complexities of transforming case-control PRS values to the liability scale (see Methods). We analyzed 50 simulation replicates for each simulation. We assessed the performance of DDx-PRS and Marginal-PRS (Table 2) in classifying each diagnostic category vs. the rest (SCZ vs. rest, BIP vs. rest, MDD vs. rest, and control vs. rest). Calibration was assessed using the ICI^22^, defined as the weighted absolute difference between the predicted probability and the true probability, and prediction accuracy was assessed using the area under the ROC curve (AUC)^23^. All simulations were performed in R version 4.3.0. A more detailed description of the simulations is provided in the Supplementary Note.

### Psychiatric Genomics Consortium data

The empirical analyses were conducted using data from the Psychiatric Genomics Consortium (PGC)^25^. The full PGC data that we analyzed consisted of European-ancestry case-control GWAS data for SCZ^8^ (76 PGC case-control cohorts; *N*=53,386 cases and 77,258 controls), BIP^15^ (36 PGC case-control cohorts; *N*=41,917 cases and 371,549 controls), and MDD^16,17^ (34 PGC case-control cohorts; *N*=173,140 cases and 331,433 controls, consisting of the data from ref.^17^ excluding 23andMe) (Supplementary Table 1). We note that some of the individuals defined as SCZ cases in ref.^8^ were diagnosed with schizoaffective disorder; however, these pertained to only a small subset of some of the cases of 14 of the 90 cohorts, and thus we believe that the overall results are representative of SCZ. We also note that DDx-PRS assumes that controls are screened for the absence of SCZ/BIP/MDD, but the PGC applied different screening strategies per cohort ranging from no screening to screening for MDD, BIP, non-affective psychosis and substance use disorders (i.e., super-normal)^16,17^ (Supplementary Table 2), thus the PGC controls are likely different from the population of people presenting to a psychiatric facility who do not have SCZ/BIP/MDD; secondary analyses excluding controls suggest that the impact of the screening of controls is limited (Supplementary Figure 12).

We constructed 4 test cohorts by merging selected PGC case-control cohorts, and we constructed nonoverlapping training data sets for each disorder-specific PGC case-control cohort that was included in one of the 4 test cohorts.

#### Construction of 4 test cohorts

The 4 test cohorts were constructed by merging selected PGC case-control cohorts of SCZ^8^, BIP^15^ and MDD^16^. Control test samples were selected from the set of MDD controls, which have more stringent criteria for being labeled as a control (Table 1)^16^. SCZ cases were matched to BIP cases by merging PGC case-control cohorts that were from the same country and had at least 200,000 overlapping SNPs on their genotyping chip; BIP cases were matched to MDD cases with the same criteria. Combining the SCZ-BIP match with the BIP-MDD match also assured an accurate SCZ-MDD match (as confirmed with sensitivity analyses; see Supplementary Note). This resulted in 4 merged test cohorts: GER, UK1, UK2 and USA. From these test cohorts, subjects were randomly selected to obtain sample sizes of exactly 25% of each of SCZ, BIP, MDD and control (the main prior used throughout this study). The resulting test data consisted of 11,460 individuals (2,865 of each of SCZ, BIP, MDD, control): 3,368 in the GER test cohort, 1,136 in UK1, 6,080 in UK2, and 876 in USA. An overview of the PGC case-control cohorts included in the four test cohorts is provided in Supplementary Table 2.

When applying methods that require tuning data (DDx-PRS-tuned & Direct-tuned), we primarily focused on cross-cohort tuning, designating one test cohort as the tuning cohort and another test cohort as the test cohort. This resulted in 4×3=12 tuning/test cohort pairs used for analyses. We restricted tuning data to 200 samples from each of the four diagnostic categories (total *N*_tuning_=800) and evaluated the performance of each method in test data. In addition to cross-cohort tuning, we also conducted analyses based on within-cohort tuning in the cohorts GER and UK2 (UK1 and USA were too small to split in tuning and testing data).

#### Construction of training data sets for each disorder-specific PGC case-control test cohort

For each disorder-specific PGC case-control cohort that was included in one of the 4 test cohorts, we constructed corresponding training data sets (used to compute case-control PRS for each disorder) that excluded that PGC case-control cohort. For PGC SCZ case-control cohort X, the SCZ training data consisted of the full SCZ data excluding PGC SCZ case-control cohort X, the BIP training data consisted of the full BIP data, and the MDD training data consisted of the full MDD data (and analogously for PGC BIP case-control cohort X and PGC MDD case-control cohort X). With this strategy, we assured independence between training and test data, while also retaining maximum sample size of the training data. The precise training data thus used to compute the case-control PRS for every PGC case-control cohort is reported in Supplementary Table 1. All data handling was performed in R version 4.3.0. We provide a more detailed description of the training data, two sets of sensitivity analyses and Supplementary Table 23 in the Supplementary Note.

#### Quality control

Quality control of SNPs was based on previous work^46^. We selected SNPs with imputation INFO>0.9 and MAF>0.10 in both the training and testing data, and we took the intersection of these SNPs with the HapMap3 SNPs included in the PRS-CS LD reference panel (see below). As a secondary analyses to this strict SNP-QC, we also performed analyses retaining SNPS with INFO>0.3 and MAF>0.01 (results in Supplementary Figure 11 and Supplementary Table 14).

For quality control of subjects, we tested for sample overlap of controls between the PGC MDD case-control cohorts (used as diagnostic category ‘control’ in this study) and PGC SCZ (resp. BIP) case-control cohorts; when such overlap was found, the respective PGC SCZ (resp. BIP) case-control cohort was excluded from the SCZ (resp. BIP) training data to compute the SCZ (resp. BIP) case-control PRS in the respective PGC MDD case-control cohort. Furthermore, in secondary analyses, we applied stricter subject QC to also exclude individuals from the analyses who were outliers with respect to principal component analyses of the merged cohorts (based on visual screening), SNP missingness >5% pre-imputation, and discrepancy between reported and genotypic sex (results in Supplementary Figure 11 and Supplementary Table 14).

#### Applying PRS-CS and Plink 1.9 to compute case-control PRS

The case-control PRS in PGC and in 1000G were computed using PRS-CS (version Jun 4, 2021q)^5^. In short, sample size in the input GWAS results for PRS-CS was set at Neff^47^ resulting in posterior mean betas on the standardized observed scale with 50/50 case/control ascertainment^20^, which were subsequently transformed to the liability scale^26^. (Details of thus transforming the PRS to the liability scale are described in ref.^18^; the default PRS-CS command for PRS-CS-auto was used, i.e. “--a=1 --b=0.5 --phi=None --beta_std=False --n_iter=1000 --thin=5 --n_burnin=500 ---seed=None”). We note that the posterior mean betas could have also been computed with SBayesR^6^ or any other Bayesian polygenic scoring method that has been shown to be well-calibrated (see ref.^18^ for details).

The lability-scale posterior mean betas were used to compute case-control PRS in PGC and in 1000G with Plink 1.9 (v1.90b7; command “--score header sum center”)^48^. The allele frequencies for the “--score” command were read from 1000G with the “--read-freq” command (the default of Plink is to estimate the allele frequencies from the test data), thereby assuring (i) full independency of the test data and (ii) that DDx-PRS can also be applied on a single individual only.

#### S-LDSC and cross-trait LDSC analyses

Based on the full PGC data described above, S-LDSC with the baseline-LD (v2.0) model^49–51^ estimated the liability-scale SNP-heritabilities as 0.23 for SCZ, 0.19 for BIP and 0.09 for MDD, assuming population prevalences of 0.01, 0.02 and 0.16 respectively. Cross-trait LDSC^19^ estimated genetic correlations of 0.71 for SCZ-BIP, 0.34 for SCZ-MDD and 0.45 for BIP-MDD, and cross-trait LDSC intercept (equal to the correlations of error terms^20^) of 0.24 for SCZ-BIP, 0.07 for SCZ-MDD and 0.09 for BIP-MDD. These liability scale SNP heritabilities and genetic correlation estimates are used as input in steps 1 and 3 of DDx-PRS and steps 1 and 3 of DDx-PRS-tuned (see above), and as input parameters for simulations (see above).

### Simulations of case-control PRS directly for projections of clinical utility at larger training sample sizes

We computed projections to larger training sample sizes by directly simulating case-control PRS (instead of simulating individual-level SNP data; see Main simulations above) at different values of % of SNP-heritability captured by case-control PRS, ranging from 40% (similar to PGC data; 42-47%) to 100%. We verified that our empirical findings were similar to simulated results at the exact empirical parameters for both simulating PRS directly (as described here) and simulating individual-level SNP data (described above; see Supplementary Figure 18 and Supplementary Table 22). A detailed description of these simulations is provided in the Supplementary Note.

### Simulation of potential of socioeconomic status to distinguish SCZ, BIP and MDD

To benchmark the projections of clinical utility at larger training sample sizes, we simulated the expected potential of socioeconomic status (SES) to distinguish SCZ, BIP and MDD. To this aim we used estimates of the genetic correlation between SCZ, BIP, MDD and SES^37^, and assumed these reflect the correlation between the liability scales of SCZ, BIP, MDD and SES (which corresponds to assuming that the genetic correlation and environmental correlation are the same^52,53^). We simulated the liabilities and SES for 5,000,000 individuals and selected a subset with 25% for each of SCZ/BIP/MDD/control, ordered this subset according to SES and determined the proportion of SCZ/BIP/MDD/control in the 10 ordered deciles (reported in Figure 6).

As primary SES metric we used the Townsend deprivation index with genetic correlations of 0.21, 0.07, 0.36 with SCZ, BIP and MDD respectively^37^. In secondary analyses we considered Income with genetic correlations of -0.09, 0.11, -0.29 respectively, and Educational Attainment with genetic correlations of 0.06, 0.19, -0.17^37^.

## Extended Data

**Extended Data Figure 1:**
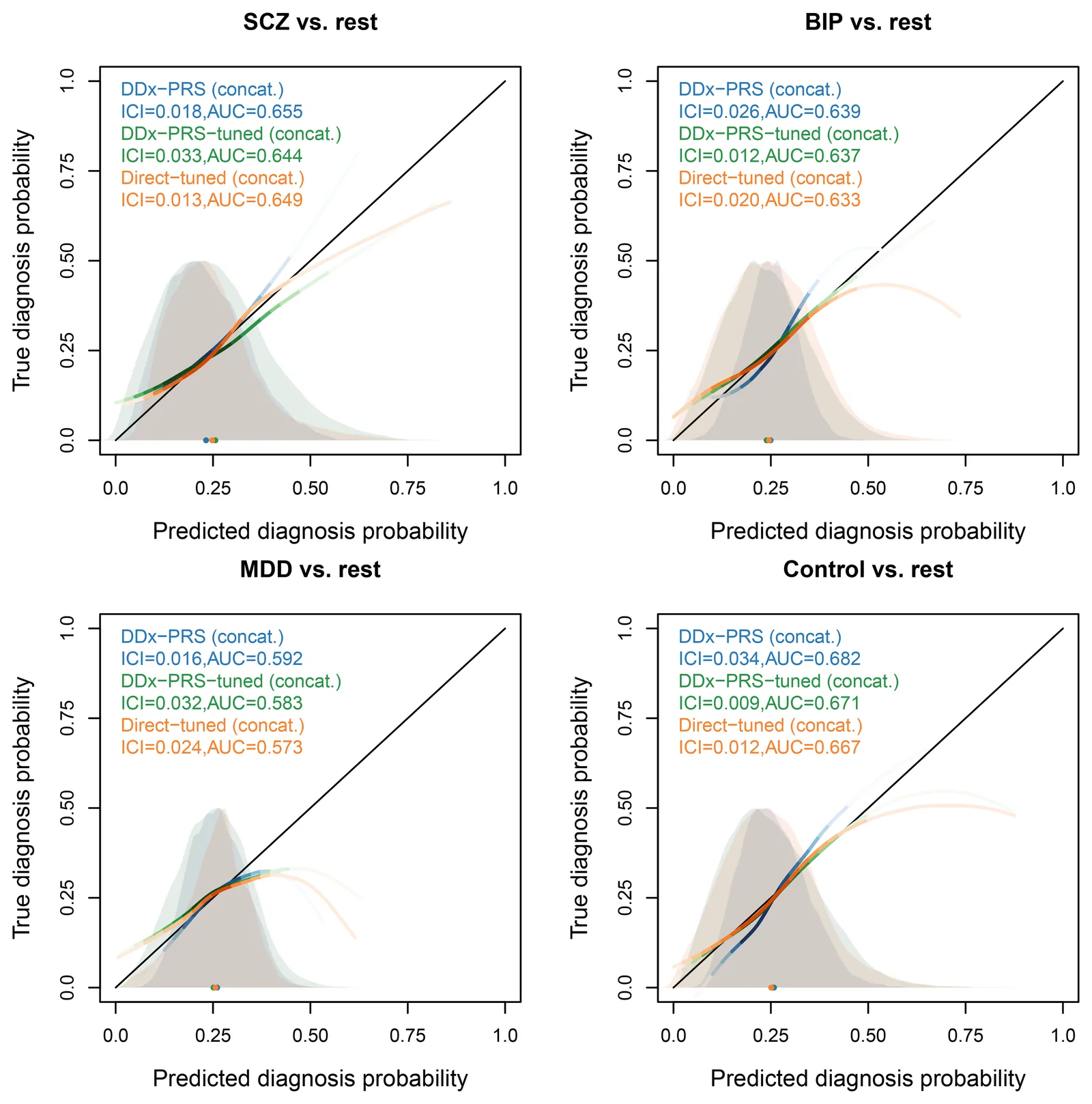
Projections of clinical utility at larger training sample sizes. We report the true diagnosis probability per decile of predicted diagnosis probability for the comparisons of schizophrenia (SCZ) vs. rest, bipolar disorder (BIP) vs. rest, major depressive disorder (MDD) vs. rest, control vs. rest, and maximum (max) predicted probability vs. rest, for DDx-PRS. Empirical results in PGC data are reported as black points (error bars in grey) connected with dashed black lines; error bars denote 1.96 times the SE across the four PGC test cohorts. Projections (simulations) at various values of SNP-heritability captured by case-control PRS (40%, 60%, 80% and 100%) are reported as blue points connected with solid blue lines, with varying colour intensity. We also report simulated projections to gauge the potential to distinguish diagnostic categories using socio-economic status (SES). The baseline diagnosis probability (i.e. prior clinical probability) equals 0.25 for each of SCZ, BIP, MDD and control (dashed grey horizontal lines). We note that the true diagnosis probability for max predicted probability vs. rest corresponds to Accuracy of most likely diagnosis. PGC results for DDx-PRS-tuned and Direct-tuned are reported in Figure S17 and Table S21. Numerical results, including error bars for all data points, are reported in Table S19. The values of the predicted diagnosis probabilities are reported in Table S20.

## Supplementary Material

Supplementary Information

Supplementary Tables

## Figures and Tables

**Table 1:** Overview of configurations of liabilities and disorder classification. For schizophrenia (SCZ), bipolar disorder (BIP) and major depressive disorder (MDD), we list the eight configurations of liabilities (i.e. liability (liab) above or below the liability threshold (thresh) for each disorder) and the corresponding diagnostic category. *These would likely be diagnosed as schizoaffective disorder following DSM-5 classification criteria (or as comorbid SCZ and MDD for configuration *c*_3_), but are labelled here as SCZ in line with ref.^8^. **Control is defined as the absence of SCZ, BIP and MDD.

Number	Configuration of liabilities			Diagnostic category
	liab_scz_ >thresh_scz_	liab_BIP_ >thresh_BIP_	liab_MDD_ >thresh_MDD_	
***c*** **_1_**	**1**	**1**	**1**	SCZ*
***c*** **_2_**	**1**	**1**	**0**	SCZ*
***c*** **_3_**	**1**	**0**	**1**	SCZ*
***c*** **_4_**	**1**	**0**	**0**	**SCZ**
***c*** **_5_**	**0**	**1**	**1**	**BIP**
***c*** **_6_**	**0**	**1**	**0**	**BIP**
***c*** **_7_**	**0**	**0**	**1**	**MDD**
***c*** **_8_**	**0**	**0**	**0**	Control**

**Table 2:** List of methods for genetically distinguishing different disorders. We list the methods for genetically distinguishing different disorders evaluated in this paper, and indicate whether they use summary statistics only (as opposed to summary statistics plus tuning data), whether they model multivariate density (across four diagnostic categories or eight configurations from Table 1), and whether they specifically model all configurations from Table 1.

Method	Uses summary statistics only	Models multivariate density	Models eight configurations
DDx-PRS	yes	yes	yes
Marginal-PRS	yes	no	no
DDx-PRS-tuned	no	yes	yes
Direct-tuned	no	yes	no

## Data Availability

DDx-PRS is a multivariate method to jointly estimate posterior probabilities, applied to predict four diagnostic categories, and the results therefore cannot be deposited in the PGS Catalog. The full case-control GWAS results for Schizophrenia, Bipolar Disorder and Major Depressive Disorder can be downloaded from the website from the Psychiatric Genomics Consortium (PGC): https://pgc.unc.edu/for-researchers/download-results/. Access to individual-level PGC data and GWAS results derived from subsets of the full case–control data requires submission of a Secondary Analysis Proposal; see https://pgc.unc.edu/for-researchers/individual-level-data-access/ for details.

## Code availability

DDx-PRS software: https://github.com/wouterpeyrot/DDxPRS & https://doi.org/10.5281/zenodo.15018879 (ref.^54^)

PRS-CS software: https://github.com/getian107/PRScs

Plink1.9 software: https://www.cog-genomics.org/plink/
