# Supplementary material for: Distinguishing different psychiatric disorders using DDx-PRS
Source: Nat Genet. Author manuscript; Available in PMC 2026 Oct 28. (PMC13559851; doi:10.1038/s41588-026-02684-x)
Supplement: Supplementary file 1 [file EMS218219-supplement-Supplementary_Information.pdf]

Table of Contents

List of PGC SCZ consortium members 2
List of PGC BIP consortium members 4
List of PGC MDD consortium members 6
Affiliations of all listed PGC consortium members 7
Supplementary Note 31
1. Supplementary results of the subsection 'Simulations' 31
2. Supplementary results of the subsection 'DDx-PRS distinguishes SCZ, BIP, MDD and controls in PGC data' 32
3. Supplementary results of the subsection 'Comparison of DDx-PRS to methods that require tuning data' 32
4. Supplementary results of the subsection 'Projections of clinical utility at larger training sample sizes' 33
5. Supplementary discussion of 'the potential clinical utility of DDx-PRS' 33
6. Supplementary discussion of limitations 34
7. Supplementary methods of the 'DDx-PRS method' subsection 36
8. Supplementary methods of the 'Marginal-PRS method' subsection 40
9. Supplementary methods of the 'Methods that require tuning data: DDx-PRS-tuned & Direct-tuned' subsection 41
10. Supplementary methods of the 'Main simulations with individual-level SNP data' subsection 44
11. Supplementary methods of the 'Psychiatric Genomics Consortium data' subsection 46
12. Supplementary methods of the 'Simulations of case-control PRS directly for projections of clinical utility at larger training sample sizes' subsection 48
Supplementary Table captions 50
Supplementary Figures 54
References 98

List of PGC SCZ consortium members

Vassily Trubetskoy 4, Antonio F Pardiñas 46, Georgia Panagiotaropoulou 4, Swapnil Awasthi 4, Tim B Bigdeli 47,48,49, Charlotte A Dennison 46, Lynsey S Hall 46, Max Lam 50,51,52, Oleksandr Frei 53,54,55, Alexander L Richards 46, Jakob Grove 56,57,58, Zhiqiang Li 59,60, Mark Adams 61, Ingrid Agartz 53,62,63, Elizabeth G Atkinson 64,65, Esben Agerbo 66,56, Mariam Al Eissa 67, Margot Albus 68, Madeline Alexander 69, Behrooz Z Alizadeha 70,71, Köksal Alptekin 72,73, Thomas D Als 56,57,58, Farooq Amin 74, Volker Arolt 75, Manuel Arrojo 76, Lavinia Athanasiu 53,54, Maria Helena Azevedo 77, Silviu A Bacanu 78, Nicholas J Bass 67, Martin Begemann 79, Richard A Belliveau 80, Judit Bene 81, Beben Benyamin 82,83,84, Sarah E Bergen 85, Giuseppe Blasi 86, Julio Bobes 87,88,89, Stefano Bonassi 90, Alice Braun 4, Rodrigo Affonseca Bressan 91,92, Evelyn J Bromet 93, Richard Bruggeman 70,94, Peter F Buckley 95, Randy L Buckner 96, Jonas Bybjerg-Grauholm 97,98, Wiepke Cahn 99,100, Murray J Cairns 101,102,103, Monica E Calkins 104, Vaughan J Carr 105,106,107, David Castle 108,109, Stanley V Catts 110,111, Kimberley D Chambert 112, Raymond CK Chan 113,114, Boris Chaumette 115,116, Wei Cheng 117, Eric FC Cheung 118, Siow Ann Chong 119,120, David Cohen 121,122,123, Angèle Consoli 121,124, Quirino Cordeiro 125, Javier Costas 126, Charles Curtis 127,128, Michael Davidson 129, Kenneth L Davis 130, Lieuwe de Haan 131,132, Franziska Degenhardt 133, Lynn E DeLisi 134,135, Ditte Demontis 56,57,58, Faith Dickerson 136, Dimitris Dikeos 137, Timothy Dinan 138,139, Srdjan Djurovic 140,141, Jubao Duan 142,143, Giuseppe Ducci 144, Johan G Eriksson 145,146,147, Lourdes Fañanás 148,149, Stephen V Faraone 150, Alessia Fiorentino 67, Andreas Forstner 133,151, Josef Frank 152, Nelson B Freimer 153,154, Menachem Fromer 155, Alessandra Frustaci 156, Ary Gadelha 91,92, Giulio Genovese 80, Elliot S Gershon 157, Marianna Giannitelli 158,122, Ina Giegling 159, Paola Giusti-Rodríguez 160, Stephanie Godard 161, Jacqueline I Goldstein 162, Javier González Peñas 163,164, Ana González-Pinto 165,164, Srihari Gopal 166, Jacob Gratten 167,168, Michael F Green 169,170, Tiffany A Greenwood 171, Olivier Guillin 172,173,174, Sinan Gülöksüz 175,176, Raquel E Gur 104, Ruben C Gur 104, Blanca Gutiérrez 177, Eric Hahn 178, Hakon Hakonarson 179, Vahram Haroutunian 130,180,181, Annette M Hartmann 159, Carol Harvey 108,182, Caroline Hayward 183, Frans A Henskens 184, Stefan Herms 185, Per Hoffmann 185, Daniel P Howrigan 162,186, Masashi Ikeda 187, Conrad Iyegbe 188, Inge Joa 189, Antonio Julià 190, Anna K Kähler 85, Tony Kam-Thong 191, Yoichiro Kamatani 192,193, Sena Karachanak-Yankova 194,195, Oussama Kebir 115, Matthew C Keller 196, Brian J Kelly 184, Andrey Khrunin 197, Sung-Wan Kim 198, Janis Klovins 199, Nikolay Kondratiev 200, Bettina Konte 159, Julia Kraft 4,201, Michiaki Kubo 202, Vaidutis Kučinskas 203, Zita Ausrele Kučinskiene 203, Agung Kusumawardhani 204, Hana Kuzelova-Ptackova 205, Stefano Landi 206, Laura C Lazzeroni 207,208, Phil H Lee 209,80, Sophie E Legge 46, Douglas S Lehrer 210, Rebecca Lencer 75, Bernard Lerer 211, Miaoxin Li 212, Jeffrey Lieberman 213, Gregory A Light 214,171, Svetlana Limborska 197, Chih-Min Liu 215,216, Jouko Lönnqvist 217,218, Carmel M Loughland 219, Jan Lubinski 220, Jurjen J Luykx 221,222,223, Amy Lynham 46, Milan Macek Jr 224, Andrew Mackinnon 225,226, Patrik KE Magnusson 85, Brion S Maher 227, Wolfgang Maier 228, Dolores Malaspina 130,229, Jacques Mallet 230, Stephen R Marder 231, Sara Marsal 190, Alicia R Martin 162,232,233, Lourdes Martorell 234, Manuel Mattheisen 235,236,237, Robert W McCarley 238,239, Colm McDonald 240, John J McGrath 66,241,242, Helena Medeiros 243,244, Sandra Meier 245,246, Bela Melegh 247, Ingrid Melle 53,54, Raquelle I Mesholam-Gately 248,249, Andres Metspalu 250, Patricia T Michie 251, Lili Milani 252, Vihra Milanova 253, Marina Mitjans 79, Espen Molden 254,255, Esther Molina 256, María Dolores Molto 257,164,258, Valeria Mondelli 259,260, Carmen Moreno 163,164, Christopher P Morley 261, Gerard Muntané 234,262, Kieran C Murphy 263, Inez Myin-Germeys 264, Igor Nenadić 265,266, Gerald Nestadt 267, Liene Nikitina-Zake 199, Cristiano Noto 91,92, Keith H

Nuechterlein 169, Niamh Louise O'Brien 67, F Anthony O'Neill 268, Sang-Yun Oh 269,270, Ann Olincy 271, Vanessa Kiyomi Ota 272,92, Christos Pantelis 273,274,182, George N Papadimitriou 137, Mara Parellada 163,164, Tiina Paunio 275,276, Renata Pellegrino 179, Sathish Periyasamy 277,278, Diana O Perkins 279, Bruno Pfuhlmann 280, Olli Pietiläinen 281,282,283, Jonathan Pimm 284, David Porteous 285, John Powell 286, Diego Quattrone 127,128,287, Digby Quested 288,289, Allen D Radant 290,291, Antonio Rampino 86, Mark H Rapaport 292, Anna Rautanen 191, Abraham Reichenberg 130, Cheryl Roe 293, Joshua L Roffman 294, Julian Roth 295, Matthias Rothermundt 75, Bart PF Rutten 296, Safaa Saker-Delye 297, Veikko Salomaa 298, Julio Sanjuan 299,164,258, Marcos Leite Santoro 272,92, Adam Savitz 166, Ulrich Schall 300,301, Rodney J Scott 101,302,303, Larry J Seidman 304,305, Sally Isabel Sharp 67, Jianxin Shi 306, Larry J Siever 130,307, Kang Sim 308,309,310, Nora Skarabis 4, Petr Slominsky 197, Hon-Cheong So 311,312, Janet L Sobell 243, Erik Söderman 313, Helen J Stain 314,315, Nils Eiel Steen 316,54, Agnes A. Steixner-Kumar 79, Elisabeth Stögmann 317, William S Stone 318,319, Richard E Straub 320, Fabian Streit 321, Eric Strengman 322, T Scott Stroup 213, Mythily Subramaniam 119,323, Catherine A Sugar 324,169, Jaana Suvisaari 298, Dragan M Svrakic 325, Neal R Swerdlow 171, Jin P Szatkiewicz 160, Thi Minh Tam Ta 326,327, Atsushi Takahashi 296,328, Chikashi Terao 328, Florence Thibaut 329,330, Draga Toncheva 194,331, Paul A Tooney 101,102,103, Silvia Torretta 86, Sarah Tosato 332, Gian Battista Tura 333, Bruce I Turetsky 104, Alp Üçok 334, Arne Vaaler 335,336, Therese van Amelsvoort 296,260, Ruud van Winkel 337,296, Juha Veijola 338,339, John Waddington 340, Henrik Walter 341, Anna Waterreus 342,343, Bradley T Webb 78, Mark Weiser 344, Nigel M Williams 46, Stephanie H Witt 152, Brandon K Wormley 78, Jing Qin Wu 345, Zhida Xu 346, Robert Yolken 347, Clement C Zai 348,349, Wei Zhou 350, Feng Zhu 351,352, Fritz Zimprich 353, Eşref Cem Atbaşoğlu 354,229, Muhammad Ayub 355, Alessandro Bertolino 86, Donald W Black 356, Nicholas J Bray 46, Gerome Breen 127, Nancy G Buccola 357, William F Byerley 358, Wei J Chen 359,360, C Robert Cloninger 325, Benedicto Crespo-Facorro 361,362, Gary Donohoe 363, Robert Freedman 271, Cherrie Galletly 364,365,366, Massimo Gennarelli 367,368, David M Hougaard 97,98, Hai-Gwo Hwu 216,369, Assen V Jablensky 370, Steven A McCarroll 80, Jennifer L Moran 112,371, Ole Mors 56,372, Preben B Mortensen 66,56, Bertram Müller-Myhsok 373,374,375, Amanda L Neil 376, Merete Nordentoft 97,377, Michele T Pato 378,379, Tracey L Petryshen 209, Ann E Pulver 267, Thomas G Schulze 380,381,382, Jeremy M Silverman 130,307, Jordan W Smoller 209,383, Eli A Stahl 155,384,385, Debby W Tsuang 386,387, Elisabet Vilella 234, Shi-Heng Wang 388, Shuhua Xu 389,390,391, Rolf Adolfsson 392, Celso Arango 163,164, Bernhard T Baune 75,393,274, Sintia Iole Belangero 272,92, Anders D Børglum 56,57,58, David Braff 171,394, Elvira Bramon 395, Joseph D Buxbaum 130, Dominique Campion 172,173, Jorge A Cervilla 396, Sven Cichon 397,398,399, David A Collier 400, Aiden Corvin 401, Marta Di Forti 127,260,287, Enrico Domenici 402, Hannelore Ehrenreich 79, Valentina Escott-Price 403,404, Tõnu Esko 405,406, Ayman H Fanous 407,408,409, Anna Gareeva 410,411, Micha Gawlik 412, Pablo V Gejman 142,143, Michael Gill 401, Stephen J Glatt 413, Vera Golimbet 200, Kyung Sue Hong 414, Christina M Hultman 85, Steven E Hyman 112,415, Nakao Iwata 187, Erik G Jönsson 313,416, René S Kahn 417,99, James L Kennedy 348,349, Elza Khusnutdinova 418,419, George Kirov 46, James A Knowles 420,421, Marie-Odile Krebs 115, Claudine Laurent-Levinson 121,422, Jimmy Lee 423,424, Todd Lencz 425,426,427, Douglas F Levinson 428, Qingqin S Li 166, Jianjun Liu 429,430, Anil K Malhotra 425,426,427, Dheeraj Malhotra 431, Andrew McIntosh 61, Andrew McQuillin 284, Paulo R Menezes 432, Vera A Morgan 433,434, Derek W Morris 240, Bryan J Mowry 277,278, Robin M Murray 260,435, Vishwajit Nimgaonkar 436, Markus M Nöthen 133, Roel A Ophoff 437,153,438, Sara A Paciga 439, Aarno Palotie 440,441,442, Carlos N Pato 378,379, Shengying Qin 350,443, Marcella Rietschel 152, Brien P Riley 78, Margarita Rivera 444,445, Dan Rujescu 159, Meram C Saka 446, Alan

R Sanders 142,447, Sibylle G Schwab 448,449, Alessandro Serretti 450, Pak C Sham 451,452,453, Yongyong Shi 454,455, David St Clair 456, Ming T Tsuang 457,458, Jim van Os 459,460, Marquis P Vawter 461, Daniel R Weinberger 320, Thomas Werge 462,463,464, Dieter B Wildenauer 465, Xin Yu 466,467, Weihua Yue 466,467,468, Peter A Holmans 46, Panos Roussos 469,470, Evangelos Vassos 127,260,471, Danielle Posthuma 472, Ole A Andreassen 53,54, Kenneth S Kendler 78, Michael J Owen 46, Naomi R Wray 473,277, Mark J Daly 162,474,475, Hailiang Huang 162,232,233, Benjamin M Neale 162,232, Patrick F Sullivan 476,160,477, Stephan Ripke 4,478,479, James TR Walters 46, Michael C O'Donovan 46

List of PGC BIP consortium members

Niamh Mullins 480,481, Andreas J. Forstner 482,483,484, Kevin S. O'Connell 485,486, Brandon Coombes 487, Jonathan R. I. Coleman 488,489, Zhen Qiao 490, Thomas D. Als 491,492,493, Tim B. Bigdeli 494,495, Sigrid Børte 496,497,498, Julien Bryois 476, Alexander W. Charney 481, Ole Kristian Drange 499,500, Michael J. Gandal 501, Saskia P. Hagenaars 488,489, Masashi Ikeda 502, Nolan Kamitaki 503,504, Minsoo Kim 501, Kristi Krebs 505, Georgia Panagiotaropoulou 4, Brian M. Schilder 480,506,507, Laura G. Sloofman 480, Stacy Steinberg 508, Vassily Trubetskoy 509, Bendik S. Winsvold 498,510, Hong-Hee Won 511, Liliya Abramova 512, Kristina Adorjan 380,513, Esben Agerbo 493,514,515, Mariam Al Eissa 32, Diego Albani 516, Ney Alliey-Rodriguez 517,518, Adebayo Anjorin 519, Verneri Antilla 520, Anastasia Antoniou 521, Swapnil Awasthi 509, Ji Hyun Baek 522, Marie Bækvad-Hansen 493,98, Nicholas Bass 32, Michael Bauer 523, Eva C. Beins 482, Sarah E. Bergen 476, Armin Birner 524, Carsten Bøcker Pedersen 493,514,515, Erlend Bøen 525, Marco P. Boks 526, Rosa Bosch 527,528,529, Murielle Brum 530, Ben M. Brumpton 498, Nathalie Brunkhorst-Kanaan 530, Monika Budde 380, Jonas Bybjerg-Grauholm 493,98, William Byerley 531, Murray Cairns 532, Miquel Casas 527,528,529, Pablo Cervantes 533, Toni-Kim Clarke 534, Cristiana Cruceanu 533,535, Alfredo Cuellar-Barboza 536,42, Julie Cunningham 537, David Curtis 538,539, Piotr M. Czerski 540, Anders M. Dale 541, Nina Dalkner 524, Friederike S. David 482, Franziska Degenhardt 482,542, Srdjan Djurovic 543,544, Amanda L. Dobbyn 480,481, Athanassios Douzenis 521, Torbjørn Elvsåshagen 497,545,546, Valentina Escott-Price 547, I. Nicol Ferrier 548, Alessia Fiorentino 32, Tatiana M. Foroud 549, Liz Forty 547, Josef Frank 550, Oleksandr Frei 485,497, Nelson B. Freimer 501,551, Louise Frisén 552, Katrin Gade 380,382, Julie Garnham 553, Joel Gelernter 176,554,555, Marianne Giørtz Pedersen 493,514,515, Ian R. Gizer 556, Scott D. Gordon 557, Katherine Gordon-Smith 558, Tiffany A. Greenwood 559, Jakob Grove 491,492,493, José Guzman-Parra 560, Kyooseob Ha 561, Magnus Haraldsson 562, Martin Hautzinger 563, Urs Heilbronner 380, Dennis Hellgren 476, Stefan Herms 482,398,564, Per Hoffmann 482,398,564, Peter A. Holmans 547, Laura Huckins 480,481, Stéphane Jamain 565,566, Jessica S. Johnson 480,481, Janos L. Kalman 380,513,567, Yoichiro Kamatani 568,569, James L. Kennedy 570,571,572, Sarah Kittel-Schneider 530,573, James A. Knowles 574,575, Manolis Kogevinas 576, Maria Koromina 577, Thorsten M. Kranz 530, Henry R. Kranzler 578,579, Michiaki Kubo 580, Ralph Kupka 581,582,583, Steven A. Kushner 584, Catharina Lavebratt 585,586, Jacob Lawrence 587, Markus Leber 588, Heon-Jeong Lee 589, Phil H. Lee 590, Shawn E. Levy 591, Catrin Lewis 547, Calwing Liao 592,593, Susanne Lucae 535, Martin Lundberg 585,586, Donald J. MacIntyre 594, Sigurdur H. Magnusson 508, Wolfgang Maier 595, Adam Maihofer 559, Dolores Malaspina 480,481, Eirini Maratou 596, Lina Martinsson 552, Manuel Mattheisen 491,492,493, Steven A. McCarroll 503,504, Nathaniel W. McGregor 597, Peter McGuffin 488, James D. McKay 598, Helena Medeiros 575, Sarah E. Medland 557, Vincent Millischer 585,586, Grant W. Montgomery 490, Jennifer L. Moran 503,599, Derek W. Morris 600, Thomas W. Mühleisen 483,398, Niamh O'Brien 32, Claire O'Donovan

553, Loes M. Olde Loohuis 501,551, Lilijana Oruc 601, Sergi Papiol 380,513, Antonio F. Pardiñas 547, Amy Perry 558, Andrea Pfennig 523, Evgenia Porichi 521, James B. Potash 602, Digby Quested 288,603, Towfique Raj 480,506,507, Mark H. Rapaport 604, J. Raymond DePaulo 602, Eline J. Regeer 605, John P. Rice 606, Fabio Rivas 560, Margarita Rivera 444,445, Julian Roth 573, Panos Roussos 480,481,506, Douglas M. Ruderfer 607, Cristina Sánchez-Mora 527,528,608, Eva C. Schulte 380,513, Fanny Senner 380,513, Sally Sharp 32, Paul D. Shilling 559, Engilbert Sigurdsson 562,609, Lea Sirignano 550, Claire Slaney 553, Olav B. Smeland 485,486, Daniel J. Smith 610, Janet L. Sobell 611, Christine Søholm Hansen 493,98, Maria Soler Artigas 527,528,608, Anne T. Spijker 612, Dan J. Stein 613, John S. Strauss 570, Beata Świątkowska 614, Chikashi Terao 569, Thorgeir E. Thorgeirsson 508, Claudio Toma 615,616,617, Paul Tooney 532, Evangelia-Eirini Tsermpini 577, Marquis P. Vawter 618, Helmut Vedder 619, James T. R. Walters 547, Stephanie H. Witt 550, Simon Xi 620, Wei Xu 621, Jessica Mei Kay Yang 547, Allan H. Young 622,623, Hannah Young 480, Peter P. Zandi 602, Hang Zhou 176,554, Lea Zillich 550, Rolf Adolfsson 624, Ingrid Agartz 525,625,626, Martin Alda 553,627, Lars Alfredsson 628, Gulja Babadjanova 629, Lena Backlund 585,586, Bernhard T. Baune 75,630,631, Frank Bellivier 632,633, Susanne Bengesser 524, Wade H. Berrettini 634, Douglas H. R. Blackwood 534, Michael Boehnke 635, Anders D. Børglum 493,636,637, Gerome Breen 488,489, Vaughan J. Carr 638, Stanley Catts 639, Aiden Corvin 640, Nicholas Craddock 547, Udo Dannlowski 75, Dimitris Dikeos 641, Tõnu Esko 504,505,642, Bruno Etain 632,633, Panagiotis Ferentinos 488,521, Mark Frye 42, Janice M. Fullerton 615,616, Micha Gawlik 573, Elliot S. Gershon 517,643, Fernando S. Goes 602, Melissa J. Green 615,638, Maria Grigoroiu-Serbanescu 644, Joanna Hauser 540, Frans Henskens 532, Jan Hillert 552, Kyung Sue Hong 522, David M. Hougaard 493,98, Christina M. Hultman 476, Kristian Hveem 498,645, Nakao Iwata 502, Assen V. Jablensky 646, Ian Jones 547, Lisa A. Jones 558, René S. Kahn 481,526, John R. Kelsoe 559, George Kirov 547, Mikael Landén 476,647, Marion Leboyer 565,566,648, Cathryn M. Lewis 488,489,649, Qingqin S. Li 650, Jolanta Lissowska 651, Christine Lochner 652, Carmel Loughland 532, Nicholas G. Martin 557,653, Carol A. Mathews 654, Fermin Mayoral 560, Susan L. McElroy 655, Andrew M. McIntosh 594,656, Francis J. McMahon 657, Ingrid Melle 485,658, Patricia Michie 532, Lili Milani 505, Philip B. Mitchell 638, Gunnar Morken 499,659, Ole Mors 493,660, Preben Bo Mortensen 491,493,514, Bryan Mowry 639, Bertram Müller-Myhsok 535,661,662, Richard M. Myers 591, Benjamin M. Neale 503,520,642, Caroline M. Nievergelt 559,663, Merete Nordentoft 493,377, Markus M. Nöthen 482, Michael C. O'Donovan 547, Ketil J. Oedegaard 664,665, Tomas Olsson 666, Michael J. Owen 547, Sara A. Paciga 667, Chris Pantelis 668, Carlos Pato 575, Michele T. Pato 575, George P. Patrinos 577,669,670, Roy H. Perlis 671,672, Danielle Posthuma 673,674, Josep Antoni Ramos-Quiroga 527,528,529, Andreas Reif 530, Eva Z. Reininghaus 524, Marta Ribasés 527,528,608, Marcella Rietschel 550, Stephan Ripke 503,509,520, Guy A. Rouleau 593,675, Takeo Saito 502, Ulrich Schall 532, Martin Schalling 585,586, Peter R. Schofield 615,616, Thomas G. Schulze 380,550,382, Laura J. Scott 635, Rodney J. Scott 532, Alessandro Serretti 676, Cynthia Shannon Weickert 615,638,677, Jordan W. Smoller 503,599,678, Hreinn Stefansson 508, Kari Stefansson 508,679, Eystein Stordal 680,681, Fabian Streit 550, Patrick F. Sullivan 476,682,683, Gustavo Turecki 684, Arne E. Vaaler 685, Eduard Vieta 686, John B. Vincent 570, Irwin D. Waldman 687, Thomas W. Weickert 615,638,677, Thomas Werge 493,462,463, Naomi R. Wray 490,688, John-Anker Zwart 497,498,510, Joanna M. Biernacka 487,42, John I. Nurnberger 689, Sven Cichon 482,483,398, Howard J. Edenberg 549,690, Eli A. Stahl 480,481,642, Andrew McQuillin 32, Arianna Di Florio 547,683, Roel A. Ophoff 501,551,584, Ole A. Andreassen 485,486

List of PGC MDD consortium members

Mark J Adams 534, Fabian Streit 691, Swapnil Awasthi 692, Brett N Adey 45, Karmel W Choi 693,694, V Kartik Chundru 695, Jonathan RI Coleman 45,696, Jerome C Foo 691, Olga Giannakopoulou 32, Alisha S M Hall 691,697, Jens Hjerling-Leffler 698, David M Howard 45, Christopher Hübel 45,699,700, Alex S F Kwong 534,701, Bochao Danae Lin 702, Xiangrui Meng 32, Guiyan Ni 703, Oliver Pain 704, Gita A Pathak 705,706, Eva C Schulte 707,708,709, Jackson G Thorp 710, Alicia Walker 703, Shuyang Yao 711, Jian Zeng 703, Johan Zvrskovec 45,696, Dag Aarsland 712, Ky'Era V Actkins 713, Mazda Adli 692,714, Esben Agerbo 699,715,716, Mareike Aichholzer 717, Tracy M Air 718, Allison Aiello 719, Thomas D Als 716,720,721, Evelyn Andersson 722, Till F M Andlauer 723,724, Volker Arolt 725, Helga Ask 726,727, Sunita Badola 728, Clive Ballard 729, Karina Banasik 730, Nicholas J Bass 32, Aartjan T F Beekman 731, Sintia Belangero 732, Elisabeth B Binder 724,733, Ottar Bjerkeset 734,735, Gyda Bjornsdottir 736, Julia Boberg 722, Sigrid Børte 737,738,739, Emma Bränn 740, Alice Braun 741, Thorsten Brodersen 742, Søren Brunak 730, Mie T Bruun 743, Pichit Buspavanich 744,745, Jonas Bybjerg-Grauholm 746,747, Enda M Byrne 748, Archie Campbell 749,750, Megan L. Campbell 751, Enrique Castelao 752, Jorge Cervilla 753,754, Boris Chaumette 755, Chia-Yen Chen 756, Zhengming Chen 757,758, Sven Cichon 759,760,761, Lucía Colodro-Conde 710, Anne Corbett 729, Elizabeth C Corfield 726,762, Baptiste Couvy-Duchesne 763, Nick Craddock 764,765, Udo Dannlowski 725, Gail Davies 766, EJC de Geus 767, Ian J Deary 766, Franziska Degenhardt 768,769, Abbas Dehghan 770,771, J Raymond DePaulo 772, Michael Deuschle 773, Maria Didriksen 774, Khoa Manh Dinh 775, Nese Direk 776, Srdjan Djurovic 777,778, Anna R Docherty 779,780,781, Katharina Domschke 782, Joseph Dowsett 774, Ole Kristian Drange 735,783,784, Erin C Dunn 694,785, Gudmundur Einarsson 736, Thalia C Eley 45, Samar S M Elsheikh 786, Jan Engelmann 787, Michael E Benros 746,788,789, Christian Erikstrup 775, Valentina Escott-Price 765, Chiara Fabbri 45,790, Yu Fang 791, Sarah Finer 792, Josef Frank 691, Robert C Free 793, He Gao 794, Michael Gill 795, Maria Gilles 773, Fernando S Goes 772, Scott Douglas Gordon 710, Jakob Grove 716,720,721, Daniel F Gudbjartsson 736,796, Blanca Gutierrez 753,754, Tim Hahn 725, Lynsey S Hall 765, Thomas F Hansen 730,746,797, Magnus Haraldsson 798, Catherina A Hartman 799, Alexandra Havdahl 726, Caroline Hayward 800, Stefanie Heilmann-Heimbach 768, Stefan Herms 760,768, Ian B Hickie 801, Henrik Hjalgrim 802, Per Hoffmann 760,768, Georg Homuth 803, Carsten Horn 804, Jouke-Jan Hottenga 767, David M Hougaard 746,747, Iiris Hovatta 805, Qin Qin Huang 695, Floris Huider 767, Karen A Hunt 806, Marcus Ising 807, Erkki Isometsä 808, Rick Jansen 731, Yunxuan Jiang 809, Ian Jones 765, Lisa A Jones 810, Lina Jonsson 811, Robert Karlsson 711, Siegfried Kasper 812, Kenneth S Kendler 813, Ronald C Kessler 814, Stefan Kloiber 786,807,815, James A Knowles 816, Nastassja Koen 751, Julia Kraft 741, Henry R Kranzler 817,818, Kristi Krebs 819, Theodora Kunovac Kallak 820, Zoltán Kutalik 821,822,823, Elisa Lahtela 824, Margit Hørup Larsen 774, Eric J Lenze 825, Daniel F Levey 826,827, Melissa Lewins 534, Glyn Lewis 32, Liming Li 828,829, Kuang Lin 757, Penelope A Lind 710, Donald J MacIntyre 534,830,831, Dean F MacKinnon 772, Hermine HM Maes 832,833, Wolfgang Maier 834, Victoria S Marshe 786,835, Hamdi Mbarek 767, Peter McGuffin 45, Sarah E Medland 710, Susanne Meinert 725,836, Susan Mikkelsen 775, Christina Mikkelsen 774,837, Yuri Milaneschi 731, Iona Y Millwood 757,758, Brittany L Mitchell 710, Esther Molina 753,838, Francis M Mondimore 772, Preben Bo Mortensen 699,715,716, Benoit H Mulsant 786,815, Joonas Naamanka 805, Jake M Najman 839, Matthias Nauck 840,841, Igor Nenadić 842, Kasper R Nielsen 843, Ilja M Nolte 844, Merete Nordentoft 746,788,789, Markus M Nöthen 768, Mette Nyegaard 716,845,846, Michael C O'Donovan 765, Asmundur Oddsson 736, Catherine M Olsen 847,848, Hogni Oskarsson 849, Sisse Rye Ostrowski 774,850, Vanessa K Ota 732, Michael J Owen 765, Richard Packer 851, Teemu Palviainen 824, Pedro M Pan 852, Carlos N Pato 853, Michele T Pato 853, Nancy L Pedersen 711, Ole

Birger Pedersen 854, Roseann E Peterson 813,855, Wouter J Peyrot 731, James B Potash 772, Martin Preisig 752, Jorge A Quiroz 856, Charles F Reynolds III 857, John P Rice 825, Giovanni A Salum 858, Robert A Schoevers 859,860, Andrew Schork 716,861,862, Thomas G Schulze 691,708,772, Tabea S Send 773, Jianxin Shi 863, Engilbert Sigurdsson 798, Kritika Singh 713, Grant C B Sinnamon 864, Lea Sirignano 691, Olav B Smeland 865,866, Daniel J Smith 610, Erik Sørensen 774, Sundararajan Srinivasan 867, Hreinn Stefansson 736, Kari Stefansson 736,868, Dan J. Stein 869, Frederike Stein 870, André Tadic 787,871, Henning Teismann 872, Alexander Teumer 873, Anita Thapar 765,874, Pippa A Thomson 750, Lise Wegner Thørner 774, Apostolia Topaloudi 875, Ioanna Tzoulaki 770,771,876, Monica Uddin 877, André G Uitterlinden 878, Henrik Ullum 774,879,880, Daniel Umbricht 881, Robert J Ursano 882, Sandra Van der Auwera 883, David A van Heel 806, Albert M van Hemert 884, Abirami Veluchamy 867, Alexander Viktorin 711, Henry Völzke 873, Agaz Wani 877, G Bragi Walters 736, Robin G Walters 757,758, Sylvia Wassertheil-Smoller 885, Myrna M Weissman 886,887, Jürgen Wellmann 872, David C Whiteman 847, Derek Wildman 877, Gonneke Willemsen 767, Alexander T Williams 851, Bendik S Winsvold 737,738,888, Stephanie H Witt 691, Ying Xiong 711, Lea Zillich 691, John-Anker Zwart 737,738,739, 23andMe Research Team 809, Estonian Biobank Research Team 819, HUNT All-In Psychiatry 889, China Kadoorie Biobank Collaborative Group 890, Genes & Health Research Team 891, Ole A Andreassen 865,866,892, Bernhard T Baune 893,894,895, Klaus Berger 872, Dorret I Boomsma 767, Anders D Børglum 716,720,721, Gerome Breen 45,696, Na Cai 896,897,898, Hilary Coon 780, William E Copeland 899, Byron Creese 729, Lea K Davis 713, Eske M Derks 710, Enrico Domenici 900, Paul Elliott 770,771,876, Andreas J Forstner 759,768, Micha Gawlik 901, Joel Gelernter 706,826,902, Hans J Grabe 883, Steven P Hamilton 903, Kristian Hveem 904,905,906, Catherine John 851,907, Jaakko Kaprio 824, Tilo Kircher 842, Marie-Odile Krebs 908, Karoline Kuchenbaecker 32,757, Mikael Landén 711,811, Kelli Lehto 819, Douglas F Levinson 909, Qingqin S Li 910, Klaus Lieb 787, Yi Lu 711, Susanne Lucae 807, Jurjen J Luykx 702,911, Patrik K Magnusson 711, Nicholas G Martin 710, Hilary C Martin 695, Andrew McQuillin 32, Christel M Middeldorp 748,912, Lili Milani 819, Ole Mors 716,913, Daniel J Müller 786,815,914, Bertram Müller-Myhsok 724,915,662, Albertine J Oldehinkel 799, Sara A Paciga 916, Colin Palmer 867, Peristera Paschou 875, Brenda WJH Penninx 731, Roy H Perlis 693,694,917, Giorgio Pistis 752, Renato Polimanti 705,706, David J Porteous 750, Danielle Posthuma 918,919, Ted Reichborn-Kjennerud 726, Andreas Reif 717, Frances Rice 765,920, Roland Ricken 692, Marcella Rietschel 691, Margarita Rivera 753,921, Christian Rück 922, Catherine Schaefer 923, Srijan Sen 791,924, Alessandro Serretti 790, Alkistis Skalkidou 820, Jordan W Smoller 693,925,926, Frederike Stein 870, Murray B Stein 927,928,711, Patrick F Sullivan 711,929, Martin Tesli 726, Thorgeir E Thorgeirsson 736, Henning Tiemeier 930,931, Nicholas J Timpson 701, Rudolf Uher 932, Jens R Wendland 728, Thomas Werge 746,861,880, Naomi R Wray 703,933, Stephan Ripke 692,925, Cathryn M Lewis 45,934, Andrew M McIntosh 534,935

Affiliations of all listed PGC consortium members

1. Department of Psychiatry, Amsterdam UMC, Vrije Universiteit, Amsterdam, The Netherlands; 2. Department of Complex Trait Genetics, Center for Neurogenomics and Cognitive Research, Amsterdam Neuroscience, Vrije Universiteit Amsterdam, Netherlands; 3. Amsterdam Public Health, Amsterdam UMC, Vrije Universiteit, Amsterdam, The Netherlands; 4. Dept. of Psychiatry and Psychotherapy, Charité - Universitätsmedizin, Berlin 10117, Germany; 5. Center for Neurobehavioral Genetics, University of California Los Angeles, Los Angeles, CA, USA; 6. Department of Genetics and Genomics, University of California Los Angeles, Los Angeles, CA, USA; 7. Department of Computational Medicine, University of California Los Angeles, Los Angeles, CA, USA; 8. Institute of Neuroscience and

Cardiovascular Research, University of Edinburgh, Edinburgh, UK; 9. Division of Mental Health and Addiction, Oslo University Hospital, Oslo, NO; 10. Centre for Precision Psychiatry, University of Oslo, Oslo, NO; 11. KG Jebsen Centre for Neurodevelopmental Disorders, University of Oslo, Oslo, NO; 12. Institute of Human Genetics, University of Bonn, School of Medicine & University Hospital Bonn, Bonn, Germany; 13. Institute of Neuroscience and Medicine (INM-1), Research Center Jülich, Jülich, Germany; 14. Psychiatric and Neurodevelopmental Genetics Unit, Center for Genomic Medicine, Massachusetts General Hospital, Boston, MA, USA; 15. Center for Precision Psychiatry, Department of Psychiatry, Massachusetts General Hospital, Boston, MA, USA; 16. Mental Health and Neuroscience, QIMR Berghofer Medical Research Institute, Brisbane, QLD, Australia; 17. Department of Psychiatry, Icahn School of Medicine at Mount Sinai, New York, NY, US; 18. Charles Bronfman Institute for Personalized Medicine, Icahn School of Medicine at Mount Sinai, New York, NY, US; 19. Department of Genetics and Genomic Sciences, Icahn School of Medicine at Mount Sinai, New York, NY, US; 20. NORMENT, University of Oslo, Oslo, NO; 21. Stanley Center for Psychiatric Research, Broad Institute of Harvard and MIT, Cambridge MA 02142, USA; 22. German Center for Mental Health (DZPG), site Berlin-Potsdam; 23. Center for Digital Genomic Medicine, Department of Medicine, Vanderbilt University Medical Center, Nashville, TN, US; 24. Departments of Biomedical Informatics and Psychiatry and Behavioral Sciences, Vanderbilt University Medical Center, Nashville, TN, US; 25. Division of Genetic Medicine, Department of Medicine, Vanderbilt Genetics Institute, Vanderbilt University Medical Center, Nashville, TN, US; 26. National Centre for Register-based Research, Aarhus University, Aarhus, Denmark; 27. Bioinformatics Research Centre, Aarhus University, Aarhus, Denmark; 28. Novo Nordisk Foundation Center for Genomics Mechanisms of Disease, Broad Institute of MIT and Harvard, Cambridge, MA, USA; 29. Department of Epidemiology, Harvard T.H. Chan School of Public Health, Boston, MA, USA; 30. Department of Biostatistics, Harvard T.H. Chan School of Public Health, Boston, MA, USA; 31. Program in Medical and Population Genetics, Broad Institute of MIT and Harvard, Cambridge, MA, USA; 32. Division of Psychiatry, University College London, London, UK; 33. Department of Psychiatry, Trinity College Dublin, Dublin, Ireland; 34. Department of Psychiatry and Psychotherapy, Comprehensive Center for Clinical Neurosciences and Mental Health, Medical University of Vienna, Austria; 35. Virginia Institute for Psychiatric and Behavioral Genetics, Department of Psychiatry, Virginia Commonwealth University, Richmond VA, USA; 36. Centre for Neuropsychiatric Genetics and Genomics, Department of Psychological Medicine and Clinical Neurosciences, Cardiff University School Of Medicine, Cardiff CF24 4HQ, UK; 37. Centre For Public Health, Institute Of Clinical Sciences, Queens University Belfast, Belfast, NI; 38. Virginia Institute for Psychiatric and Behavioral Genetics, Departments of Psychiatry and Cellular, Molecular & Genetic Medicine, Virginia Commonwealth University, Richmond VA, USA; 39. Lieber Institute for Brain Development, Departments of Psychiatry, Neurology, Neuroscience and Genetic Medicine, Johns Hopkins School of Medicine, Baltimore, Md. 21205 USA; 40. Clinical Division of Psychiatry and Psychotherapeutic Medicine, Medical University of Graz, Austria; 41. Department of Quantitative Health Sciences, Mayo Clinic, Rochester, MN, USA; 42. Department of Psychiatry and Psychology, Mayo Clinic, Rochester, MN, USA; 43. Department of Psychiatry and Psychology, Mayo Clinic, Rochester MN, USA 55905; 44. Lindner Center of HOPE, Manon, OH &University of Cincinnati College of Medicine, Cincinnati, OH, USA; 45. Social, Genetic and Developmental Psychiatry Centre, King's College London, London, UK; 46. MRC Centre for Neuropsychiatric Genetics and Genomics, Division of Psychiatry and Clinical Neurosciences, Cardiff University, Hadyn Ellis Building, Maindy Road, Cardiff, CF24 4HQ; 47. Department of Psychiatry and the Behavioral Sciences, State University of New York, Downstate Medical Center, 450 Clarkson Ave, Brooklyn, NY, 11203; 48. Institute for Genomic Health,

SUNY Downstate Medical Center, Brooklyn, NY, USA; 49. Department of Psychiatry, Veterans Affairs New York Harbor Healthcare System, Brooklyn, NY, USA; 50. Stanley Center for Psychiatric Research, Broad Institute of MIT and Harvard, Cambridge, MA 02142; 51. Research Division, Institute of Mental Health, Singapore, Republic of Singapore; 52. Division of Psychiatry Research, Zucker Hillside Hospital, Glen Oaks NY, USA; 53. NORMENT Centre, Division of Mental Health and Addiction, University of Oslo, 0424 Oslo, Norway; 54. Division of Mental Health and Addiction, Oslo University Hospital, 0424 Oslo, Norway; 55. Center for Bioinformatics, Department of Informatics, University of Oslo, PO box 1080, Blindern, 0316 Oslo, Norway; 56. The Lundbeck Foundation Initiative for Integrative Psychiatric Research (iPSYCH), Aarhus, Denmark; 57. Department of Biomedicine and Centre for Integrative Sequencing (iSEQ), Aarhus University, Aarhus, Denmark.; 58. Center for Genomics and Personalized Medicine, Aarhus, Denmark; 59. Affiliated Hospital of Qingdao University & Biomedical Sciences Institute of Qingdao University, Qingdao University; 60. Bio-X Institutes, Key Laboratory for the Genetics of Developmental and Neuropsychiatric Disorders (Ministry of Education), Collaborative Innovation Centre for Brain Science, Shanghai Jiao Tong University; 61. Division of Psychiatry, Centre for Clinical Brain Sciences, University of Edinburgh, Royal Edinburgh Hospital, Edinburgh, EH10 5HF, UK; 62. Department of Psychiatric Research, Diakonhjemmet Hospital, Oslo, Norway.; 63. Centre for Psychiatry Research, Department of Clinical Neuroscience, Karolinska Institutet & Stockholm Health Care Services, Stockholm County Council, Stockholm, Sweden.; 64. Analytic and Translational Genetics Unit, Massachusetts General Hospital, Boston, MA; 65. Stanley Center for Psychiatric Research, Broad Institute of MIT and Harvard, Cambridge, MA; 66. National Centre for Register-based Research, Aarhus University, DK-8210 Aarhus, Denmark; 67. Molecular Psychiatry Laboratory, Division of Psychiatry, University College London, London, WC1E 6BT; 68. Comedicum Lindwurmhof, Lindwurmstr. 88, 80337, Munich, Germany; 69. Center for Depression, Anxiety and Stress Research, McLean Hospital, Belmont, MA, USA; 70. University Medical Center Groningen, University Center for Psychiatry, Rob Giel Research Center, Unviersity of Groningen, Groningen, The Netherlands; 71. Department of Epidemiology, University Medical Center Groningen, Unviersity of Groningen, Groningen, The Netherlands; 72. Department of Psychiatry, Dokuz Eylül University School of Medicine, Izmir, Turkey; 73. Department of Neuroscience, Dokuz Eylül Univ Graduate School of Health Sciences; 74. Department of Psychiatry and Behavioral Sciences, Emory University, Atlanta, Georgia 30322, USA; 75. Department of Psychiatry, University of Münster, Münster, Germany; 76. Servizo de Psiquiatría, Complexo Hospitalario Universitario de Santiago de Compostela, Servizo Galego de Saúde (SERGAS), Santiago de Compostela, Galicia, Spain; 77. Institute of Medical Psychology, Faculty of Medicine, University of Coimbra, Coimbra, PT, Portugal.; 78. Virginia Institute for Psychiatric and Behavioral Genetics, Department of Psychiatry, Virginia Commonwealth University, Richmond, Virginia 23298, USA; 79. Clinical Neuroscience, Max Planck Institute of Experimental Medicine, Göttingen 37075, Germany; 80. Stanley Center for Psychiatric Research, Broad Institute of MIT and Harvard, Cambridge, Massachusetts 02142, USA; 81. Department of Medical Genetics, Medical School, University of Pécs, Pécs, Hungary; 82. Australian Centre for Precision Health, University of South Australia Cancer Research Institute, University of South Australia, Adelaide, Australia; 83. UniSA Allied Health & Human Performance, University of South Australia, Adelaide, Australia; 84. South Australian Health and Medical Research Institute, Adelaide, South Australia, Australia; 85. Department of Medical Epidemiology and Biostatistics, Karolinska Institutet, Stockholm SE-17177, Sweden; 86. Department of Basic Medical Science, Neuroscience and Sense Organs, University of Bari 'Aldo Moro', Bari, Italy; 87. Área de Psiquiatría-Universidad de Oviedo, Hospital Universitario Central de Asturias (HUCA), Asturias, Spain; 88. Instituto de Investigación Sanitaria del Principado de Asturias (ISPA), Asturias,

Spain; 89. Centro de Investigación Biomédica en Red de Salud Mental, Oviedo, Asturias, Spain; 90. Unit of Clinical and Molecular Epidemiology, IRCCS San Raffaele Pisana, and San Raffaele University, Rome, Italy; 91. Department of Psychiatry, Universidade Federal de Sao Paulo, Rua Major Maragliano, 241-CEP 04017-030, Sao Paulo, SP, Brazil; 92. Laboratory of Integrative Neuroscience, Universidade Federal de Sao Paulo, Rua Pedro de Toledo, 669 - 3° andar fundos, CEP 04039-032, Sao Paulo, SP, Brazil; 93. Department of Psychiatry and Behavioural Health, Stony Brook University, HSC, Level T-10, Stony Brook, NY; 94. University of Groningen, Department of Clinical and Developmental Neuropsychology, Groningen, The Netherlands; 95. School of Medicine, Virginia Commonwealth University,, Richmond VA, USA; 96. Department of Psychology, Harvard University, Cambridge, Massachusetts 02138, USA; 97. The Lundbeck Foundation Initiative for Integrative Psychiatric Research (iPSYCH), Denmark; 98. Center for Neonatal Screening, Department for Congenital Disorders, Statens Serum Institut, Copenhagen, Denmark; 99. University Medical Center Utrecht, Department of Psychiatry, Rudolf Magnus Institute of Neuroscience, 3584 Utrecht, The Netherlands; 100. Altrecht, General Menthal Health Care, Utrecht, The Netherlands; 101. School of Biomedical Sciences and Pharmacy, University of Newcastle, Callaghan NSW 2308, Australia; 102. Hunter Medical Research Institute, Newcastle, New South Wales, Australia; 103. Centre for Brain & Mental Health Research, The University of Newcastle, Callaghan, NSW, Australia; 104. Department of Psychiatry, University of Pennsylvania, 3400 Spruce Street, Philadelphia, PA 19104; 105. School of Psychiatry, University of New South Wales, Sydney, Australia; 106. Department of Psychiatry, Monash University, Melbourne, Australia; 107. Neuroscience Research Australia, Sydney, Australia; 108. Department of Psychiatry, The University of Melbourne, Parkville, Victoria 3010, Australia; 109. St Vincent's Hospital, 41 Victoria Parade, Fitzroy, Victoria3065, Australia.; 110. Brain and Mind Centre, The University of Sydney, Sydney, NSW, Australia; 111. School of Medicine, University of Queensland, Herston, QLD Australia; 112. Stanley Center for Psychitric Research, Broad Institute of MIT and Harvard, Cambridge, MA 02142; 113. Institute of Psychology, Chinese Academy of Science, Beijing 100101, China; 114. Department of Psychology, University of Chinese Academy of Sciences, Beijing, China; 115. INSERM U1266, Institute of Psychiatry and Neuroscience of Paris, Université de Paris, GHU Paris Psychiatrie & Neurosciences, 108 rue de la Santé, 75014 Paris, France; 116. Department of Psychiatry, McGill University, Montreal, Canada; 117. Department of Computer Science, University of North Carolina, Chapel Hill, North Carolina 27514, USA; 118. Castle Peak Hospital, Hong Kong, China; 119. Research Division, Institute of Mental Health, Singapore; 120. Saw Swee Hock School of Public Health, University of Singapore, Singapore; 121. Faculté de Médecine Sorbonne Université, Groupe de Recherche Clinique n°15 - Troubles Psychiatriques et Développement (PSYDEV), Department of Child and Adolescent Psychiatry, Hôpital Universitaire de la Pitié-Salpêtrière, 47-83 Boulevard de l’Hôpital, 75651 Paris Cedex 13, France.; 122. Centre de Référence des Maladies Rares à Expression Psychiatrique, Department of Child and Adolescent Psychiatry, AP-HP Sorbonne Université, Hôpital Universitaire de la Pitié-Salpêtrière, 47 - 83 Boulevard de l’Hôpital, 75651 Paris Cedex 13, France; 123. Institut des Systèmes Intelligents et de Robotique (ISIR), CNRS UMR7222, Sorbonne Université, Campus Pierre et Marie Curie, Faculté des Sciences et Ingénierie, Pyramide, Tour 55, Boîte courrier 173, 4 Place Jussieu, 75252 Paris Cedex 05, France; 124. Centre de Référence des Maladies Rares à Expression Psychiatrique, Department of Child and Adolescent Psychiatry, AP-HP Sorbonne Université, Hôpital Universitaire de la Pitié-Salpêtrière, 47 - 83 Boulevard de l’Hôpital, 75651 Paris Cedex 13, France;; 125. Department of Psychiatry, Irmandade da Santa Casa de Misericórdia de São Paulo, Rua Dona Veridiana, 55 - São Paulo - SP - CEP 01238-010, São Paulo, SP, Brazil; 126. Instituto de Investigación Sanitaria (IDIS) de Santiago de Compostela, Complexo Hospitalario Universitario de

Santiago de Compostela (CHUS), Servizo Galego de Saúde (SERGAS), Santiago de Compostela, Galicia, Spain.; 127. Social, Genetic and Developmental Psychiatry Centre, Institute of Psychiatry, Psychology and Neuroscience, King’s College London, London SE5 8AF, UK; 128. National Institute for Health Research (NIHR) Maudsley Biomedical Research Centre at South London and Maudsley NHS Foundation Trust and King's College London, UK; 129. University of Nicosia Medical School, Nicosia, Cyprus; 130. Department of Psychiatry, Icahn School of Medicine at Mount Sinai, New York, New York 10029, USA; 131. Department of Psychiatry, Academic Medical Centre, University of Amsterdam, Amsterdam, The Netherlands; 132. Arkin, Institute for Mental Health, Amsterdam, The Netherlands; 133. Institute of Human Genetics, University of Bonn, D-53127 Bonn, Germany; 134. Cambridge Health Alliance, Cambridge, MA 02139, USA; 135. Department of Psychiatry, Harvard Medical School, Boston, Massachusetts 02115, USA; 136. Sheppard Pratt Health System, Baltimore, MD, USA; 137. First Department of Psychiatry, Medical School, National and Kapodistrian University of Athens, Eginition Hospital, Athens 11528, Greece; 138. Department of Psychiatry and Neurobehavioural Sciences, University College Cork, Cork, Ireland; 139. APC Microbiome Ireland, University College Cork, Cork, Ireland; 140. NORMENT Centre, Department of Clinical Science, University of Bergen, Bergen, Norway; 141. Department of Medical Genetics, Oslo University Hospital, 0424 Oslo, Norway; 142. Center for Psychiatric Genetics, NorthShore University HealthSystem, Evanston, IL 60201, USA.; 143. Department of Psychiatry and Behavioral Neurosciences, The University of Chicago, Chicago, IL 60637, USA.; 144. Department of Mental Health, ASL Rome 1, 00135 Rome, Italy; 145. Department of General Practice and Primary Health Care, University of Helsinki and Helsinki University Hospital, Helsinki, Finland; 146. Folkhälsan Research Center, Helsinki, Finland; 147. Department of Obstetrics & Gynecology, Yong Loo Lin School of Medicine, National University of Singapore; 148. Department of Evolutionary Biology, Ecology and Environmental Sciences, Faculty of Biology, University of Barcelona, Spain; 149. Centro de Investigación Biomédica en Red en Salud Mental (CIBERSAM), Madrid, Spain; 150. Departments of Psychiatry and Neuroscience and Physiology, SUNY Upstate Medical University, Syracuse, New York, USA; 151. Centre for Human Genetics, University of Marburg, D-35033 Marburg, Germany; 152. Department of Genetic Epidemiology in Psychiatry, Central Institute of Mental Health, Medical Faculty Mannheim, University of Heidelberg, Heidelberg, D-68159 Mannheim, Germany; 153. Department of Human Genetics, David Geffen School of Medicine, University of California, Los Angeles, California 90095, USA; 154. Department of Psychiatry and Biobehavioral Sciences, University of California, Los Angeles, Los Angeles, CA, USA; 155. Division of Psychiatric Genomics, Department of Psychiatry, Icahn School of Medicine at Mount Sinai, New York, New York 10029, USA; 156. Barnet, Enfield and Haringey Mental Health NHS Trust, St.Ann’s Hospital, St.Ann’s Road, London, N15 3 TH, UK; 157. Departments of Psychiatry and Human Genetics, University of Chicago, Chicago, Illinois 60637, USA; 158. Faculté de Médecine Sorbonne Université, Groupe de Recherche Clinique n°15 - Troubles Psychiatriques et Développement (PSYDEV), Department of Child and Adolescent Psychiatry,Hôpital Universitaire de la Pitié-Salpêtrière, 47-83 Boulevard de l’Hôpital, 75651 Paris Cedex 13, France.; 159. Department of Psychiatry and Psychotherapy, Medical University of Vienna, Vienna, Austria; 160. Department of Genetics, University of North Carolina, Chapel Hill, North Carolina 27599-7264, USA; 161. Departments of Psychiatry and Human and Molecular Genetics, INSERM, Institut de Myologie, Hôpital de la Pitiè-Salpêtrière, Paris, 75013, France; 162. Analytic and Translational Genetics Unit, Massachusetts General Hospital, Boston, Massachusetts 02114, USA; 163. Department of Child and Adolescent Psychiatry, Hospital General Universitario Gregorio Marañón, School of Medicine, Universidad Complutense, Investigación Sanitaria del Hospital Gregorio Marañón, Madrid, Spain; 164. Centro de Investigación Biomédica en Red de Salud Mental, Spain (CIBERSAM); 165. BIOARABA Health

Research Institute. OSI Araba. University Hospital, University of the Basque Country, Vitoria, Spain; 166. Neuroscience Therapeutic Area, Janssen Research and Development, Titusville, New Jersey 08560, USA; 167. Mater Research Institute, University of Queensland, Brisbane, Queensland 4102, Australia; 168. Institute for Molecular Biology, University of Queendland, Brisbane, Queensland 4072, Australia; 169. Department of Psychiatry and Biobehavioral Sciences, Geffen School of Medicine, University of California Los Angeles, 10833 Le Conte Ave, Los Angeles, CA 90095; 170. VA Greater Los Angeles Healthcare System, 11301 Wilshire Boulevard, Los Angeles, CA90073; 171. Department of Psychiatry, University of California San Diego, 9500 Gilman Drive, La Jolla, CA 92093-0804; 172. INSERM U1245, 76000 Rouen, Normandie, France; 173. Centre Hospitalier du Rouvray, Rouen 76000 France; 174. UFR santé, Université de Rouen Normandie, Rouen, France; 175. Department of Psychiatry and Neuropsychology, School for Mental Health and Neuroscience, Maastricht University Medical Centre, Maastricht, the Netherlands; 176. Department of Psychiatry, Yale School of Medicine, New Haven, CT, USA; 177. Department of Psychiatry, Faculty of Medicine and Biomedical Research Centre (CIBM), University of Granada, Granada, Spain.; 178. Department of Psychiatry, Charité - Universitätsmedizin, Berlin, Campus Benjamin Franklin, Germany; 179. Children's Hospital of Philadelphia, Leonard Madlyn Abramson Research Center,3615 Civic Center Boulevard, Suite 1216, Philadelphia, PA , USA 19104-4318; 180. Department of Neuroscience, Icahn School of Medicine at Mount Sinai, New York, New York 10029, USA; 181. Mental Illness Research Clinical and Education Center (MIRECC), JJ Peters VA Medical Center, Bronx, NY 10468, USA; 182. NorthWestern Mental Health, Melbourne, Vic, Australia; 183. MRC Human Genetics Unit, University of Edinburgh, Institute of Genetics and Molecular Medicine, Western General Hospital, Edinburgh, UK; 184. School of Medicine and Public Health, University of Newcastle, Newcastle NSW 2308, Australia; 185. Division of Medical Genetics, Department of Biomedicine, University of Basel, Basel, CH-4058, Switzerland; 186. Broad Institute of MIT and Harvard, Cambridge, Massachusetts, USA; 187. Department of Psychiatry, Fujita Health University School of Medicine, Toyoake, Aichi, 470-1192, Japan; 188. Department of Psychosis Studies, Institute of Psychiatry, Psychology and Neuroscience, King's College London, London SE5 8AF, UK; 189. Regional Centre for Clinical Research in Psychosis, Department of Psychiatry, Stavanger University Hospital, 4011 Stavanger, Norway; 190. Rheumatology Research Group, Vall d'Hebron Research Institute, Barcelona, 08035, Spain; 191. Roche Pharma Research and Early Development, Pharmaceutical Sciences, Roche Innovation Center Basel, F. Hoffman-La Roche Ltd, Grenzacherstrasse 124, 4070 Basel, Switzerland; 192. Laboratory of Complex Trait Genomics, Department of Computational Biology and Medical Sciences, Graduate School of Frontier Sciences, The University of Tokyo, Tokyo,108-8639, Japan.; 193. Laboratory for Statistical Analysis, RIKEN Center for Integrative Medical Sciences, Yokohama, Kanagawa, 230-0045, Japan; 194. Department of Medical Genetics, Medical University, Sofia 1431, Bulgaria; 195. Department of Genetics, Faculty of Biology, Sofia University \"St. Kliment Ohridski\", Sofia 1164, Bulgaria; 196. Institute for Behavioural Genetics, University of Colorado Boulder, Boulder, Colorado 80309, USA; 197. Institute of Molecular Genetics of National Research Centre "Kurchatov Institute", Moscow, Russia; 198. Department of Psychiatry, Chonnam National University Medical School, Gwangju, Korea; 199. Latvian Biomedical Research and Study Centre, Riga, LV-1067, Latvia; 200. Mental Health Research Center, Moscow, Russian Federation; 201. Berlin School of Mind and Brain, Humboldt-Universität zu Berlin, Berlin, Germany; 202. RIKEN Center for Integrative Medical Sciences, Yokohama, Kanagawa, 230-0045, Japan; 203. Faculty of Medicine, Vilnius University, LT-01513 Vilnius, Lithuania; 204. Psychiatry Department, University of Indonesia - Cipto Mangunkusumo National General Hospital, Jakarta Pusat, DKI Jakarta, 10430, Indonesia; 205. Department of Psychiatry, 1st Faculty of Medicine and General University

Hospital, Prague, Czech Republic; 206. Dipartimento di Biologia, Universita' di Pisa, Pisa, Italy; 207. Departments of Psychiatry and Behavioral Sciences, Stanford University, 450 Serra Mall, Stanford, CA 94305; 208. Department of Biomedical Data Science, Stanford University, CA; 209. Psychiatric and Neurodevelopmental Genetics Unit, Department of Psychiatry and Center for Genomic Medicine, Massachusetts General Hospital, Harvard Medical School, Boston, MA, USA; 210. Department of Psychiatry, Wright State University, 3640 Colonel Gleen Hwy, Dayton, OH; 211. Department of Psychiatry, Hadassah-Hebrew University Medical Center, Jerusalem 91120, Israel; 212. Zhongshan School of Medicine and Key Laboratory of Tropical Diseases Control (SYSU), Sun Yat-sen University, Guangzhou 510080, China; 213. Department of Psychiatry, Columbia University, New York, New York 10032, USA; 214. VISN 22, Mental Illness Research, Education & Clinical Center (MIRECC), VA San Diego Healthcare System, 3350 La Jolla Village Drive, San Diego, CA 92161; 215. Department of Psychiatry, National Taiwan University Hospital, Taipei, Taiwan; 216. Neurobiology and Cognitive Science Center, National Taiwan University, Taipei, Taiwan; 217. Mental Health Unit, Department of Public Health Solutions, National Institute for Health and Welfare, PO Box 30, FI-00271, Helsinki, Finland; 218. Department of Psychiatry, University of Helsinki, PO Box 22, FI-00014, Helsinki, Finland; 219. Hunter New England Health & University of Newcastle, Newcastle NSW 2308, Australia; 220. Department of Genetics and Pathology, International Hereditary Cancer Center, Pomeranian Medical University in Szczecin, 70-453 Szczecin, Poland; 221. Department of Psychiatry, UMC Utrecht Brain Center, University Medical Centre Utrecht, Utrecht University, Utrecht, The Netherlands; 222. Department of Translational Neuroscience, UMC Utrecht Brain Center, University Medical Center Utrecht, Utrecht University, Utrecht, The Netherlands.; 223. Second opinion outpatient clinic, GGNet mental health, Warnsveld, The Netherlands; 224. Department of Biology and Medical Genetics, 2nd Faculty of Medicine and University Hospital Motol, 150 06 Prague, Czech Republic; 225. Black Dog Institute, University of new South Wales, NSW, Australia; 226. Melbourne School of Population and Global health, University of Melbourne, Victoria, Australia; 227. Department of Mental Health, Bloomberg School of Public Health, Johns Hopkins University, Baltimore, Maryland 21205, USA; 228. Department for Neurodegenerative Diseases and Geriatric Psychiatry, University Hospital Bonn, Bonn, Germany; 229. Department of Genetics & Genomics, Icahn School of Medicine at Mount Sinai, Mount Sinai, NY, USA; 230. Asfalia Biologics, iPEPS-ICM, Hôpital Universitaire de la Pitié-Salpêtrière, AP-HP, Paris 75013, France; 231. Semel Institute for Neurosciene, University of California, Los Angeles, 11301 Wilshire Blvd, Los Angeles, CA; 232. Stanley Center for Psychiatric Research, the Broad Institute of MIT and Harvard, Cambridge, MA, USA; 233. Department of Medicine, Harvard Medical School, Boston, MA, USA; 234. Hospital Universitari Institut Pere Mata, IISPV, Universitat Rovira i Virgili, CIBERSAM, Reus, Spain; 235. Department of Psychiatry, Dalhousie University, Halifax NS, Canada; 236. Department of Community Health and Epidemiology, Dalhousie University, Halifax Nova Scotia, Canada; 237. Department of Biomedicine and Centre for Integrative Sequencing (iSEQ), Aarhus University, Aarhus, Denmark; 238. VA Boston Health Care System, Brockton, Massachusetts 02301, USA; 239. Deceased; 240. Centre for Neuroimaging, Cognition and Genomics (NICOG), National University of Ireland Galway, Galway, Ireland.; 241. Queensland Brain Institute, University of Queensland, St Lucia, Queensland, Australia; 242. Queensland Centre for Mental Health Research, The Park Centre for Mental Health, Queensland, Australia; 243. Department of Psychiatry and the Behavioral Sciences, Keck School of Medicine, University of Southern California, Los Angeles, CA, USA; 244. College of Medicine, SUNY Downstate Health Sciences University, Brooklyn, NY, USA; 245. Department of Psychiatry, Dalhousie University, 5850/5980 University Avenue | PO Box 9700, Halifax, Nova Scotia | B3K 6R8; 246. Department of Biomedicine, Aarhus University, DK-8000 Aarhus C, Denmark; 247.

Department of Medical Genetics, University of Pécs, Pécs H-7624, Hungary; 248. Massachusetts Mental Health Center Public Psychiatry Division of the Beth Israel Deaconess Medical Center, Boston, Massachusetts 02114, USA; 249. Department of Psychiatry, Harvard Medical School, Boston MA, USA; 250. Estonian Genome Center, Institute of Genomics, University of Tartu, Tartu 50090, Estonia; 251. School of Psychology, University of Newcastle, Newcastle NSW 2308, Australia; 252. Estonian Genome Center, Institute of Genomics, University of Tartu, Tartu 51010, Estonia; 253. First Psychiatric Clinic, Medical University, Sofia 1431, Bulgaria; 254. Department of Pharmacy, University of Oslo, Oslo, Norway; 255. Center for Psychopharmacology, Diakonhjemmet Hospital, Oslo, Norway; 256. Department of Nursing, Faculty of Health Sciences and Biomedical Research Centre (CIBM), University of Granada, Granada, Spain.; 257. Department of Genetics, Faculty of Biological Sciences, Campus of Burjassot, Universidad de Valencia, Valencia, Spain; 258. Biomedical Research Institute INCLIVA, Valencia, Spain; 259. Department of Psychological Medicine, Institute of Psychiatry, Psychology, and Neuroscience, King's College London, London, SE5 8AF, UK; 260. National Institute for Health Research (NIHR) Mental Health Biomedical Research Centre at South London and Maudsley NHS Foundation Trust and King's College London, UK; 261. Departments of Public Health and Preventive Medicine, Family Medicine, and Psychiatry and Behavioral Sciences, State University of New York, Upstate Medical University, Syracuse, NY; 262. Institut de Biologia Evolutiva (UPF-CSIC), Departament de Ciències Experimentals i de la Salut, Universitat Pompeu Fabra, PRBB, Barcelona, Spain; 263. Department of Psychiatry, Royal College of Surgeons in Ireland, Dublin, Ireland; 264. Department for Neurosciences, Center for Contextual Psychiatry, KU Leuven, Leuven, Belgium; 265. Cognitive Neuropsychiatry Lab, Department of Psychiatry and Psychotherapy, Philipps Universität Marburg, Marburg, Germany; 266. Department of Psychiatry and Psychotherapy, Jena University Hospital, 07743 Jena, Germany; 267. Department of Psychiatry and Behavioral Sciences, Johns Hopkins University School of Medicine, Baltimore, Maryland 21205, USA; 268. Centre for Public Health, Institute of Clinical Sciences, Queen's University Belfast, Belfast BT12 6AB, UK; 269. Department of Statistics and Applied Probability, University of California at Santa Barbara, Santa Barbara, California, USA.; 270. Computational Research Division, Lawrence Berkeley National Laboratory, Berkeley, California, USA; 271. Department of Psychiatry, University of Colorado Denver, Aurora, Colorado 80045, USA; 272. Department of Morphology and Genetics, Universidade Federal de Sao Paulo, Rua Botucatu, 740, Laboratorio de Genetica, CEP 04023-900, Sao Paulo, SP, Brazil; 273. Melbourne Neuropsychiatry Centre, University of Melbourne & Melbourne Health, Melbourne VIC 3053, Australia; 274. The Florey Institute of Neuroscience and Mental Health, The University of Melbourne, Parkville, VIC, Australia; 275. Department of Public Health Solutions, Genomics and Biomarkers Unit, National Institute for Health and Welfare, PO Box 30, FI-00271, Helsinki; 276. Department of Psychiatry and SleepWell Research Program, Faculty of medicine, University of Helsinki and Helsinki University Central Hospital; 277. Queensland Brain Institute, The University of Queensland, Brisbane, QLD 4072, Australia; 278. Queensland Centre for Mental Health Research, The University of Queensland, Brisbane, QLD, Australia; 279. Department of Psychiatry, University of North Carolina, Chapel Hill, North Carolina 27599-7160, USA; 280. Clinic of Psychiatry and Psychotherapy, Weißer Hirsch, Dresden, Germany; 281. Department of Stem Cell and Regenerative Biology, Harvard University, Cambridge, MA 02138, USA; 282. The Stanley Center for Psychiatric Research, The Broad Institute, Cambridge, MA 02142, USA; 283. Institute for Molecular Medicine Finland, FIMM, University of Helsinki, P.O. BOX 20 FI-00014, Helsinki, Finland; 284. Molecular Psychiatry Laboratory, Division of Psychiatry, University College London, London WC1E 6JJ, UK; 285. Centre for Genomic and Experimental Medicine, Institute of Genetics and Molecular Medicine, University of Edinburgh,

Western General Hospital, Crewe Road, Edinburgh, EH4 2XU, UK; 286. Department of Basic and Clinical Neuroscience, Institute of Psychiatry, Psychology and Neuroscience, King's College London, London SE5 8AF, UK; 287. South London and Maudsley NHS Mental Health Foundation Trust; 288. Oxford Health NHS Foundation Trust, Warneford Hospital, Oxford, UK; 289. Department of Psychiatry, University of Oxford, Oxford, OX3 7JX, UK; 290. Department of Psychiatry and Behavioral Sciences, University of Washington, 2815 Eastlake Ave E #200, Seattle, WA 98102; 291. VA Puget Sound Health Care System, 1660 S. Columbian Way, Seattle, WA 98108; 292. Huntsman Mental Health Institute, Department of Psychiatry, University of Utah School of Medicine, Salt Lake City, UT, USA; 293. SUNY Upstate Medical University, Syracuse, New York, USA; 294. Department of Psychiatry, Massachusetts General Hospital, Boston, Massachusetts 02114, USA; 295. Department of Psychiatry, Psychosomatics and Psychotherapy, Julius-Maximilians-Universität Würzburg, Wuerzburg, Germany; 296. Maastricht University Medical Center, Department of Psychiatry and Neuropsychology, School for Mental Health and Neuroscience, Maastricht, The Netherlands; 297. Généthon, 1 bis, Rue de l'Internationale, 91000 Evry, France.; 298. THL-Finnish Institute for Health and Welfare, P.O BOX 30, Mannerheimintie 166, FI-00271 Helsinki, Finland; 299. Department of Psychiatry, School of Medicine, University of Valencia, Hospital Clínico Universitario de Valencia, Spain; 300. Priority Centre for Brain & Mental Health Research, The University of Newcastle, Mater Hospital, McAuley Centre, Waratah, New South Wales 2298, Australia; 301. Hunter Medical Research Institute, New Lambton Heights, New South Wales 2305, Australia; 302. Division of Molecular Medicine, NSW Health Pathology North, Newcastle, NSW 2305, Australia; 303. Hunter Medical Research Institute, Newcastle NSW, Australia; 304. Department of Psychiatry, Harvard Medical School, 25 Shattuck St, Boston, MA 02115; 305. Massachusetts Mental Health Center Public Psychiatry Division of the Beth Israel Deaconess Medical Center, 330 Brookline Ave, Boston, MA 02215; 306. Division of Cancer Epidemiology and Genetics, National Cancer Institute, Bethesda, Maryland 20892, USA; 307. James J. Peters VA Medical Center, 130 W Kingsbridge Road, Bronx, NY 10468; 308. West Region, Institute of Mental Health, Singapore; 309. Yoo Loo Lin School of Medicine, National University of Singapore; 310. Lee Kong Chian School of Medicine, Nanyang Technological University, Singapore; 311. School of Biomedical Sciences, The Chinese University of Hong Kong, Hong Kong, China; 312. Department of Psychiatry, The Chinese University of Hong Kong, Hong Kong, China; 313. Centre for Psychiatry Research, Department of Clinical Neuroscience, Karolinska Institutet & Stockholm Health Care Services, Stockholm Region, SE-171 77 Stockholm, Sweden; 314. School of Social and Health Sciences, Leeds Trinity University, Leeds; 315. TIPS - Network for Clinical Research in Psychosis; Stavanger University Hospital, Stavanger, Norway; 316. NORMENT Centre, Institute of Clinical Medicine, University of Oslo, 0424 Oslo, Norway; 317. Department of Clinical Neurology, Medical University of Vienna, 1090 Wien, Austria; 318. Harvard Medical School Department of Psychiatry at Beth Israel Deaconess Medical Center, 330 Brookline Ave, Boston MA 02215; 319. Massachusetts Mental Health Center, 75 Fenwood Road, MA 02115; 320. Lieber Institute for Brain Development, Baltimore, Maryland 21205, USA; 321. Department of Genetic Epidemiology in Psychiatry, Central Institute of Mental Health, Medical Faculty Mannheim, University of Heidelberg, Heidelberg,D-68159 Mannheim, Germany; 322. Department of Medical Genetics, University Medical Centre Utrecht, Universiteitsweg 100, 3584 CG, Utrecht, The Netherlands; 323. Saw Swee Hock School of Public Health, National University of Singapore; 324. Department of Biostatistics, Fielding School of Public Health, University of California Los Angeles, 650 Charles E. Young Dr. South, Los Angeles, CA 90095; 325. Department of Psychiatry, Washington University, St. Louis, Missouri 63110, USA; 326. Department of Psychiatry, Campus Benjamin Franklin, Charité – Universitätsmedizin Berlin; 327. Berlin Institute of Health (BIH), 10178 Berlin, Germany; 328. Laboratory for Statistical and Translational

Genetics, RIKEN Center for Integrative Medical Sciences, Yokohama, Kanagawa, 230-0045, Japan; 329. Université de Paris, Faculté de médecine, Hôpital Cochin-Tarnier, Paris 75006, France; 330. INSERM U1266, Institut de psychiatrie et de neurosciences, Paris, France; 331. Bulgarian Academy of Science; 332. Department of Neuroscience, Biomedicine and Movement Sciences, Section of Psychiatry, University of Verona, 37134 Verona, Italy; 333. Psychiatry Unit, IRCCS Istituto Centro San Giovanni di Dio Fatebenefratelli, Brescia, Italy; 334. Department of Psychiatry, Faculty of Medicine, Istanbul University, Istanbul, Turkey; 335. Division of Mental Health, St. Olav's Hospital, Trondheim University Hospital, Trondheim, Norway; 336. Department of Mental Health, Norwegian University of Science and Technology, Trondheim, Norway; 337. KU Leuven, Department of Neurosciences, Center for Clinical Psychiatry, Leuven, Belgium; 338. Department of Psychiatry, Research Unit of Clinical Neuroscience, University of Oulu, Oulu, Finland; 339. Medical Research Center Oulu, Oulu University Hospital and University of Oulu, Oulu, Finland; 340. Molecular and Cellular Therapeutics, Royal College of Surgeons in Ireland, Dublin 2, Ireland; 341. Department for Psychiatry and Psychotherapy, CCM Charité Universitätsmedizin Berlin, corporate member of Freie Universität Berlin, Humboldt-Universität zu Berlin, and Berlin Institute of Health, Berlin, Germany; 342. Neuropsychiatric Epidemiology Research Unit, School of Population and Global Health, University of Western Australia, Perth, Australia; 343. Centre for Clinical Research in Neuropsychiatry, Division of Psychiatry, Medical School, University of Western Austral; 344. Sheba Medical Center, Tel Hashomer 52621, Israel; 345. School of Life and Environmental Sciences, University of Sydney, Sydney, NSW 2006, Australia; 346. Department of Psychiatry, GGz Centraal, The Netherlands; 347. Stanley Neurovirology Laboratory, Johns Hopkins University School of Medicine, Baltimore, MD, 21287, USA.; 348. Campbell Family Mental Health Research Institute, Centre for Addiction and Mental Health, Toronto, Ontario, M5T 1R8, Canada; 349. Department of Psychiatry, University of Toronto, Toronto, ON, Canada; 350. Bio-X Institutes, Key Laboratory for the Genetics of Developmental and Neuropsychiatric Disorders, Ministry of Education, Shanghai Jiao Tong University, Shanghai 200030, PR China; 351. Department of Psychiatry, The First Affiliated Hospital of Xi'an Jiaotong University, 277 Yanta West Road, Xi'an 710061, China.; 352. Center for Translational Medicine, The First Affiliated Hospital of Xi'an Jiaotong University, 277 Yanta West Road, Xi'an 710061, China.; 353. Department of Neurology, Medical University of Vienna, Austria; 354. Department of Psychiatry, Ankara University Faculty of Medicine, Ankara, Turkey; 355. Department of Psychiatry, Queens University Kingston, 191 Portsmouth Avenue, Kingston ON Canada K7M 8A6; 356. Department of Psychiatry, University of Iowa Carver College of Medicine, Iowa City, Iowa 52242, USA; 357. School of Nursing, Louisiana State University Health Sciences Center, New Orleans, Louisiana 70112, USA; 358. Department of Psychiatry, University of California San Francisco, San Francisco, California, 94143 USA; 359. Center for Neuropsychiatric Research, National Health Research Institutes, Zhunan Town, Miaoli County, Taiwan; 360. Institute of Epidemiology and Preventive Medicine, College of Public Health, National Taiwan University, Taipei, Taiwan; 361. University of Sevilla, CIBERSAM, Sevilla, Spain; 362. Hospital Universitario Virgen del Rocio, Department of Psychiatry, Universidad del Sevilla, Sevilla, Spain; 363. Centre for Neuroimaging, Cognition and Genomics (NICOG), National University of Ireland Galway, Galway, Ireland; 364. Discipline of Psychiatry, Adelaide Medical School, University of Adelaide, Adelaide, SA, Australia.; 365. Ramsay Health Care (SA) Mental Health; 366. Northern Adelaide Local Health Network; 367. Department of Molecular and Translational Medicine, University of Brescia, Brescia, Italy; 368. Genetic Unit, IRCCS Istituto Centro San Giovanni di Dio Fatebenefratelli, Brescia, Italy; 369. Department of Psychiatry, College of Medicine and National Taiwan University Hospital, National Taiwan University, Taipei, Taiwan; 370. Centre for Clinical Research in Neuropsychiatry, The University of Western

Australia, Perth, WA, Australia; 371. Department of Psychiatry, Massachusetts General Hospital, Boston, MA 02114, USA; 372. Psychosis Research Unit, Aarhus University Hospital, Aarhus, Denmark; 373. Max Planck Institute of Psychiatry, 80804 Munich, Germany; 374. Munich Cluster for Systems Neurology, Munich, Germany; 375. Department of Health Data Science, University of Liverpool, Liverpool, UK; 376. Menzies Institute for Medical Research, University of Tasmania; 377. Mental Health Services in the Capital Region of Denmark, Mental Health Center Copenhagen, University of Copenhagen, Copenhagen, Denmark; 378. Rutgers University, Robert Wood Johnson Medical School, New Brunswick NJ, USA; 379. Rutgers University, New Jersey Medical School, Newark NJ, USA; 380. Institute of Psychiatric Phenomics and Genomics (IPPG), University Hospital, LMU Munich, Munich, Germany; 381. Department of Psychiatry and Behavioral Sciences, SUNY Upstate Medical University, Syracuse, NY, USA; 382. Department of Psychiatry and Psychotherapy, University Medical Center Göttingen, Göttingen, Germany; 383. Stanley Center for Psychiatric Research, Broad Institute of MIT and Harvard, Cambridge, MA 02138, USA; 384. Medical and Population Genetics, The Broad Institute of MIT and Harvard, Cambridge, MA, USA; 385. Regeneron Genetics Center; 386. VA Puget Sound Health Care System, 1660 S. Columbian Way, Seattle, WA 98108D WA 98102; 387. Department of Psychiatry and Behavioral Sciences, University of Washington, Seattle,; 388. College of Public Health, China Medical University, Taichung, Taiwan; 389. State Key Laboratory of Genetic Engineering and Ministry of Education (MOE) Key Laboratory of Contemporary Anthropology, Collaborative Innovation Center of Genetics and Development, Human Phenome Institute, School of Life Sciences, Fudan University, Shanghai, China; 390. School of Life Science and Technology, ShanghaiTech University, Shanghai, China; 391. Center for Excellence in Animal Evolution and Genetics, Chinese Academy of Sciences, Kunming, China; 392. Department of Clinical Sciences, Psychiatry, Umeå University, SE-901 87 Umeå, Sweden; 393. Department of Psychiatry, Melbourne Medical School, The University of Melbourne, Parkville, VIC, Australia; 394. VISN 22, Mental Illness Research, Education & Clinical Center (MIRECC) , VA San Diego Healthcare System, 3350 La Jolla Village Drive, San Diego, CA 92161; 395. Division of Psychiatry, Department of Mental Health Neuroscience, University College London, London WC1E 6BT, UK; 396. Department of Psychiatry, San Cecilio University Hospital, University of Granada, Granada, Spain.; 397. Institute of Medical Genetics and Pathology, University Hospital Basel, Schönbeinstrasse 40, CH-4031 Basel, Switzerland; 398. Department of Biomedicine, University of Basel, Basel, Switzerland; 399. Institute of Neuroscience and Medicine (INM-1), Research Center Juelich, Juelich, Germany; 400. Eli Lilly and Company, Erl Wood Manor, Sunninghill Road, Windlesham, Surrey, GU20 6PH, UK; 401. Neuropsychiatric Genetics Research Group, Department of Psychiatry, Trinity College Dublin, Dublin, Ireland; 402. Department of Cellular, Computational and Integrative Biology, University of Trento, Trento, Italy; 403. Dementia Research Institute, Cardiff University, Hadyn Ellis Building, Maindy Road, Cardiff, CF24 4HQ; 404. MRC Centre for Neuropsychiatric Genetics and Genomics, Cardiff University, Hadyn Ellis Building, Cardiff, CF24 4HQ; 405. Program in Medical and Population Genetics, The Broad Institute of MIT and Harvard, Cambridge, MA, USA; 406. Estonian Genome Center, Institute of Genomics, University of Tartu, Tartu, 51010, Estonia; 407. Department of Psychiatry, Phoenix VA Healthcare System, Phoenix AZ, USA; 408. Banner-University Medical Center, Phoenix AZ, USA; 409. Department of Psychiatry, Veterans Affairs New York Harbor Healthcare System, Brooklyn, NY, USA.; 410. Department of Human Molecular Genetics of the Institute of Biochemistry and Genetics of the Ufa Federal Research Center of the Russian Academy of Sciences. (IBG UFRC RAS) Ufa, 450054, Prospekt Octyabrya, 71; 411. Federal State Educational Institution of Highest Education Bashkir State Medical University of Public Health Ministry of Russian Federation (BSMU). Lenina, 3. 450008 Ufa, Russia; 412. Department of Psychiatry, Psychosomatics and

Psychotherapy, University of Würzburg, Margarete-Höppel-Platz 1, 97080, Würzburg, Germany.; 413. Psychiatric Genetic Epidemiology and Neurobiology Laboratory (PsychGENe lab), Department of Psychiatry and Behavioral Sciences, SUNY Upstate Medical University, Syracuse, NY, USA; 414. Department of Psychiatry, Sungkyunkwan University School of Medicine, Samsung Medical Center, Seoul, Korea; 415. Department of Stem Cell and Regnerative Biology, Harvard University; 416. NORMENT, Institute of Clinical Medicine, University of Oslo, 0424 Oslo, Norway; 417. Department of Psychiatry, Icahn School of Medicine at Mount Sinai, New York NY, 10029, USA; 418. Institute of Biochemistry and Genetics of the Ufa Federal Research Center of the Russian Academy of Sciences (IBG UFRC RAS), Ufa, Russia; 419. Federal State Educational Institution of Highest Education Bashkir State Medical University of Public Health Ministry of Russian Federation (BSMU), Ufa, Russia; 420. Department of Psychiatry and Zilkha Neurogenetics Institute, Keck School of Medicine at University of Southern California, Los Angeles, California 90089, USA; 421. Department of Cell Biology, State University of New York, Downstate Medical Center, 450 Clarkson Ave, Brooklyn NY; 422. Centre de Référence des Maladies Rares à Expression Psychiatrique, Department of Child and Adolescent Psychiatry,AP-HP Sorbonne Université, Hôpital Universitaire de la Pitié-Salpêtrière, 47 - 83 Boulevard de l'Hôpital, 75651 Paris Cedex 13, France.; 423. Department of Psychosis, Institute of Mental Health, Singapore; 424. Neuroscience and Mental Health, Lee Kong Chian School of Medicine, Nanyang Technological University, Singapore; 425. Division of Psychiatry Research, Zucker Hillside Hospital, 75-59 263rd Street, Glen Oaks, NY, 11004, USA; 426. Institute of Behavioral Science, Feinstein Institutes for Medical Research, 350 Community Drive, Manhasset, NY, 11030, USA; 427. Department of Psychiatry, Zucker School of Medicine at Hofstra/Northwell, 500 Hofstra University, Hempstead, NY, 11549, USA; 428. Department of Psychiatry, Stanford University, 401 Quarry Rd., Stanford, CA 94305-5797, USA.; 429. Human Genetics, Genome Institute of Singapore, A*STAR, Singapore 138672, Singapore; 430. Department of Medicine, Yong Loo Lin School of Medicine, National University of Singapore, Singapore; 431. Roche Pharma Research and Early Development, Roche Innovation Center Basel, F. Hoffman-La Roche Ltd, Basel, Switzerland; 432. Department of Preventative Medicine, Faculdade de Medicina FMUSP, University of Sao Paulo, Brazil;; 433. Neuropsychiatric Epidemiology Research Unit, School of Population and Global Health M431, University of Western Australia, Perth, Australia 6009; 434. Centre for Clinical Research in Neuropsychiatry, Division of Psychiatry, Medical School, University of Western Australia, Perth, Australia 6009; 435. Department of Psychosis Studies, Institute of Psychiatry, King's College London, De Crespigny Park, Denmark Hill, London SE5 8AF, UK; 436. Department of Psychiatry, University of Pittsburgh, 3811 O'Hara St., Pittsburgh PA 15213; 437. Center for Neurobehavioral Genetics, Semel Institute for Neuroscience and Human Behavior, University of California, Los Angeles, California 90095, USA; 438. Department of Psychiatry, Erasmus University Medical Center, Rotterdam, The Netherlands; 439. Early Clinical Development, Pfizer Worldwide Research and Development, Groton, Connecticut 06340, USA; 440. Institute for Molecular Medicine Finland (FIMM), University of Helsinki, Helsinki, Finland; 441. Analytic and Translational Genetics Unit, Department of Medicine, Department of Neurology and Department of Psychiatry Massachusetts General Hospital, Boston, MA, USA.; 442. The Stanley Center for Psychiatric Research and Program in Medical and Population Genetics, The Broad Institute of MIT and Harvard, Cambridge, MA, USA.; 443. Shanghai Key Laboratory of Psychotic Disorders, Shanghai Mental Health Center, Shanghai Jiao Tong University School of Medicine, Shanghai, China.; 444. Department of Biochemistry and Molecular Biology II, Faculty of Pharmacy, University of Granada, Granada, Spain; 445. Institute of Neurosciences, Biomedical Research Center (CIBM), University of Granada, Granada, Spain; 446. Department of Psychiatry, School of Medicine, Ankara University, Ankara, Turkey; 447. Department

of Psychiatry and Behavioral Neurosciences, The University of Chicago, Chicago IL, USA; 448. Faculty of Science, Medicine and Health, School of Chemistry and Molecular Bioscience, University of Wollongong, Wollongong NSW 2522, Australia; 449. Illawarra Health and Medical Research Institute, Wollongong NSW 2522, Australia; 450. Department of Biomedical and Neuromotor Sciences, University of Bologna, Italy.; 451. Centre for PanorOmic Sciences, LKS Faculty of Medicine, The University of Hong Kong; 452. State Key Laboratory of Brain and Cognitive Sciences, LKS Faculty of Medicine, The University of Hong Kong; 453. Department of Psychiatry, LKS Faculty of Medicine, The University of Hong Kong; 454. Bio-X Institutes, Key Laboratory for the Genetics of Developmental and Neuropsychiatric Disorders (Ministry of Education), Collaborative Innovation Center for Brain Science, Shanghai Jiao Tong University, Shanghai, China.; 455. Affiliated Hospital of Qingdao University and Biomedical Sciences Institute of Qingdao University (Qingdao Branch of SJTU Bio-X Institutes), Qingdao University, Qingdao, China.; 456. Institute of Medical Sciences, University of Aberdeen, Aberdeen, AB25 2ZD, UK; 457. Center for Behavioral Genomics; Department of Psychiatry; University of California, San Diego; La Jolla, CA 92039; U.S.A.; 458. Institute of Genomic Medicine, University of California, San Diego; La Jolla, CA 92039; U.S.A; 459. University Medical Center Utrecht, Department of Psychiatry, PO BOX 85500, 3584 GA Utrecht, The Netherlands; 460. King's College London, King's Health Partners, Department of Psychosis Studies, Institute of Psychiatry, London, United Kingdom; 461. Department of Psychiatry & Human Behavior, School of Medicine, University of California, Irvine CA 92697-4260, USA; 462. Institute of Biological Psychiatry, Mental Health Services, Copenhagen University Hospital, Copenhagen, Denmark; 463. Department of Clinical Medicine, University of Copenhagen, Copenhagen, Denmark; 464. Center for GeoGenetics, GLOBE Institute, University of Copenhagen, Copenhagen, Denmark; 465. School of Psychiatry and Clinical Neurosciences, The University of Western Australia, Perth WA 6009, Australia; 466. Peking University Sixth Hospital, Peking University Institute of Mental Health, Beijing, 100191, China;; 467. National Clinical Research Center for Mental Disorders & NHC Key Laboratory of Mental Health (Peking University) & Chinese Academy of Medical Sciences Research Unit (No.2018RU006), Beijing, 100191, China.; 468. PKU-IDG/McGovern Institute for Brain Research, Peking University, Beijing, 100871, China; 469. Department of Psychiatry, Pamela Sklar Division of Psychiatric Genomics, Friedman Brain Institute, Department of Genetics and Genomic Science and Institute for Data Science and Genomic Technology, Icahn School of Medicine at Mount Sinai, New York, NY, 10029, USA; 470. Mental Illness Research, Education, and Clinical Center (VISN 2 South), James J. Peters VA Medical Center, Bronx, NY, USA; 471. Oxford Health NHS Foundation Trust; 472. Department of Functional Genomics, Center for Neurogenomics and Cognitive Research, Neuroscience Campus Amsterdam, VU University, Amsterdam 1081, The Netherlands; 473. Institute for Molecular Bioscience, The University of Queensland, Brisbane, QLD 4067, Australia; 474. Broad Institute of MIT and Harvard, Cambridge, Massachusetts, USA.; 475. Institute for Molecular Medicine Finland (FIMM), Helsinki, Finland; 476. Department of Medical Epidemiology and Biostatistics, Karolinska Institutet, Stockholm, Sweden; 477. Department of Psychiatry, University of North Carolina, Chapel Hill, North Carolina 27599-7264, USA; 478. Analytic and Translational Genetics Unit, Massachusetts General Hospital, Boston MA 02114, USA; 479. Stanley Center for Psychiatric Research, Broad Institute of MIT and Harvard, Cambridge MA 02142, USA; 480. Department of Genetics and Genomic Sciences, Icahn School of Medicine at Mount Sinai, New York, NY, USA; 481. Department of Psychiatry, Icahn School of Medicine at Mount Sinai, New York, NY, USA; 482. Institute of Human Genetics, University of Bonn, School of Medicine and University Hospital Bonn, Bonn, Germany; 483. Institute of Neuroscience and Medicine (INM-1), Research Centre Jülich, Jülich, Germany; 484. Centre for Human Genetics, University of Marburg,

Marburg, Germany; 485. Division of Mental Health and Addiction, Oslo University Hospital, Oslo, Norway; 486. NORMENT, University of Oslo, Oslo, Norway; 487. Department of Health Sciences Research, Mayo Clinic, Rochester, MN, USA; 488. Social, Genetic and Developmental Psychiatry Centre, King's College London, London, UK; 489. NIHR Maudsley BRC, King's College London, London, UK; 490. Institute for Molecular Bioscience, The University of Queensland, Brisbane, Queensland, Australia; 491. iSEQ, Center for Integrative Sequencing, Aarhus University, Aarhus, Denmark; 492. Department of Biomedicine – Human Genetics, Aarhus University, Aarhus, Denmark; 493. iPSYCH, The Lundbeck Foundation Initiative for Integrative Psychiatric Research, Aarhus, Denmark; 494. Department of Psychiatry and Behavioral Sciences, SUNY Downstate Health Sciences University, Brooklyn, NY, USA; 495. VA NY Harbor Healthcare System, Brooklyn, NY, USA; 496. Research and Communication Unit for Musculoskeletal Health, Division of Clinical Neuroscience, Oslo University Hospital, Oslo, Norway; 497. Institute of Clinical Medicine, University of Oslo, Oslo, Norway; 498. K. G. Jebsen Center for Genetic Epidemiology, Department of Public Health and Nursing, Faculty of Medicine and Health Sciences, Norwegian University of Science and Technology, Trondheim, Norway; 499. Department of Mental Health, Faculty of Medicine and Health Sciences, Norwegian University of Science and Technology (NTNU), Trondheim, Norway; 500. Department of Østmarka, Division of Mental Health Care, St Olavs Hospital, Trondheim University Hospital, Trondheim, Norway; 501. Department of Psychiatry and Biobehavioral Science, Semel Institute, David Geffen School of Medicine, University of California, Los Angeles, Los Angeles, CA, USA; 502. Department of Psychiatry, School of Medicine, Fujita Health University, Toyoake, Japan; 503. Stanley Center for Psychiatric Research, Broad Institute, Cambridge, MA, USA; 504. Department of Genetics, Harvard Medical School, Boston, MA, USA; 505. Estonian Genome Center, Institute of Genomics, University of Tartu, Tartu, Estonia; 506. Department of Neuroscience, Icahn School of Medicine at Mount Sinai, New York, NY, USA; 507. Ronald M. Loeb Center for Alzheimer's Disease, Icahn School of Medicine at Mount Sinai, New York, NY, USA; 508. deCODE Genetics/Amgen, Reykjavik, Iceland; 509. Department of Psychiatry and Psychotherapy, Charité - Universitätsmedizin, Berlin, Germany; 510. Department of Research, Innovation and Education, Division of Clinical Neuroscience, Oslo University Hospital, Oslo, Norway; 511. Samsung Advanced Institute for Health Sciences and Technology (SAIHST), Samsung Medical Center, Sungkyunkwan University, Seoul, South Korea; 512. Russian Academy of Medical Sciences, Mental Health Research Center, Moscow, Russian Federation; 513. Department of Psychiatry and Psychotherapy, University Hospital, LMU Munich, Munich, Germany; 514. National Centre for Register-Based Research, Aarhus University, Aarhus, Denmark; 515. Centre for Integrated Register-Based Research, Aarhus University, Aarhus, Denmark; 516. Department of Neuroscience, Istituto Di Ricerche Farmacologiche Mario Negri IRCCS, Milan, Italy; 517. Department of Psychiatry and Behavioral Neuroscience, University of Chicago, Chicago, IL, USA; 518. Northwestern University, Chicago, IL, USA; 519. Psychiatry, Berkshire Healthcare NHS Foundation Trust, Bracknell, UK; 520. Analytic and Translational Genetics Unit, Massachusetts General Hospital, Boston, MA, USA; 521. 2nd Department of Psychiatry, Attikon General Hospital, National and Kapodistrian University of Athens, Athens, Greece; 522. Department of Psychiatry, Samsung Medical Center, School of Medicine, Sungkyunkwan University, Seoul, South Korea; 523. Department of Psychiatry and Psychotherapy, University Hospital Carl Gustav Carus, Technische Universität Dresden, Dresden, Germany; 524. Department of Psychiatry and Psychotherapeutic Medicine, Medical University of Graz, Graz, Austria; 525. Department of Psychiatric Research, Diakonhjemmet Hospital, Oslo, Norway; 526. Psychiatry, Brain Center UMC Utrecht, Utrecht, the Netherlands; 527. Instituto de Salud Carlos III, Biomedical Network Research Centre on Mental Health (CIBERSAM), Madrid, Spain; 528. Department of

Psychiatry, Hospital Universitari Vall d´Hebron, Barcelona, Spain; 529. Department of Psychiatry and Forensic Medicine, Universitat Autònoma de Barcelona, Barcelona, Spain; 530. Department of Psychiatry, Psychosomatic Medicine and Psychotherapy, University Hospital Frankfurt, Frankfurt am Main, Germany; 531. Psychiatry, University of California San Francisco, San Francisco, CA, USA; 532. University of Newcastle, Newcastle, New South Wales, Australia; 533. Mood Disorders Program, Department of Psychiatry, McGill University Health Center, Montreal, Quebec, Canada; 534. Division of Psychiatry, University of Edinburgh, Edinburgh, UK; 535. Department of Translational Research in Psychiatry, Max Planck Institute of Psychiatry, Munich, Germany; 536. Department of Psychiatry, Universidad Autonoma de Nuevo Leon, Monterrey, Mexico; 537. Department of Laboratory Medicine and Pathology, Mayo Clinic, Rochester, MN, USA; 538. Centre for Psychiatry, Queen Mary University of London, London, UK; 539. UCL Genetics Institute, University College London, London, UK; 540. Department of Psychiatry, Laboratory of Psychiatric Genetics, Poznan University of Medical Sciences, Poznan, Poland; 541. Center for Multimodal Imaging and Genetics, Departments of Neurosciences, Radiology, and Psychiatry, University of California, San Diego, CA, USA; 542. Department of Child and Adolescent Psychiatry, Psychosomatics and Psychotherapy, University Hospital Essen, University of Duisburg-Essen, Duisburg, Germany; 543. Department of Medical Genetics, Oslo University Hospital, Oslo, Norway; 544. NORMENT, Department of Clinical Science, University of Bergen, Bergen, Norway; 545. Department of Neurology, Oslo University Hospital, Oslo, Norway; 546. NORMENT, KG Jebsen Centre for Psychosis Research, Oslo University Hospital, Oslo, Norway; 547. Medical Research Council Centre for Neuropsychiatric Genetics and Genomics, Division of Psychological Medicine and Clinical Neurosciences, Cardiff University, Cardiff, UK; 548. Academic Psychiatry, Newcastle University, Newcastle upon Tyne, UK; 549. Department of Medical and Molecular Genetics, Indiana University, Indianapolis, IN, USA; 550. Department of Genetic Epidemiology in Psychiatry, Central Institute of Mental Health, Medical Faculty Mannheim, Heidelberg University, Mannheim, Germany; 551. Center for Neurobehavioral Genetics, Semel Institute for Neuroscience and Human Behavior, Los Angeles, CA, USA; 552. Department of Clinical Neuroscience, Karolinska Institutet, Stockholm, Sweden; 553. Department of Psychiatry, Dalhousie University, Halifax, Nova Scotia, Canada; 554. Veterans Affairs Connecticut Healthcare System, West Haven, CT, USA; 555. Departments of Genetics and Neuroscience, Yale University School of Medicine, New Haven, CT, USA; 556. Department of Psychological Sciences, University of Missouri, Columbia, MO, USA; 557. Genetics and Computational Biology, QIMR Berghofer Medical Research Institute, Brisbane, Queensland, Australia; 558. Psychological Medicine, University of Worcester, Worcester, UK; 559. Department of Psychiatry, University of California San Diego, La Jolla, CA, USA; 560. Mental Health Department, University Regional Hospital, Biomedicine Institute (IBIMA), Málaga, Spain; 561. Department of Psychiatry, Seoul National University College of Medicine, Seoul, South Korea; 562. Landspitali University Hospital, Reykjavik, Iceland; 563. Department of Psychology, Eberhard Karls Universität Tübingen, Tübingen, Germany; 564. Institute of Medical Genetics and Pathology, University Hospital Basel, Basel, Switzerland; 565. Neuropsychiatrie Translationnelle, Inserm U955, Créteil, France; 566. Faculté de Santé, Université Paris Est, Créteil, France; 567. International Max Planck Research School for Translational Psychiatry (IMPRS-TP), Munich, Germany; 568. Laboratory of Complex Trait Genomics, Department of Computational Biology and Medical Sciences, Graduate School of Frontier Sciences, The University of Tokyo, Tokyo, Japan; 569. Laboratory for Statistical and Translational Genetics, RIKEN Center for Integrative Medical Sciences, Yokohama, Japan; 570. Campbell Family Mental Health Research Institute, Centre for Addiction and Mental Health, Toronto, Ontario, Canada; 571. Neurogenetics Section, Centre for Addiction and Mental Health, Toronto, Ontario, Canada; 572.

Department of Psychiatry, University of Toronto, Toronto, Ontario, Canada; 573. Department of Psychiatry, Psychosomatics and Psychotherapy, Center of Mental Health, University Hospital Würzburg, Würzburg, Germany; 574. Cell Biology, SUNY Downstate Medical Center College of Medicine, Brooklyn, NY, USA; 575. Institute for Genomic Health, SUNY Downstate Medical Center College of Medicine, Brooklyn, NY, USA; 576. ISGlobal, Barcelona, Spain; 577. Laboratory of Pharmacogenomics and Individualized Therapy, Department of Pharmacy, School of Health Sciences, University of Patras, Patras, Greece; 578. Mental Illness Research, Education and Clinical Center, Crescenz VAMC, Philadelphia, PA, USA; 579. Center for Studies of Addiction, University of Pennsylvania Perelman School of Medicine, Philadelphia, PA, USA; 580. RIKEN Center for Integrative Medical Sciences, Yokohama, Japan; 581. Psychiatry, Altrecht, Utrecht, the Netherlands; 582. Psychiatry, GGZ inGeest, Amsterdam, the Netherlands; 583. Psychiatry, VU Medisch Centrum, Amsterdam, the Netherlands; 584. Department of Psychiatry, Erasmus MC, University Medical Center Rotterdam, Rotterdam, the Netherlands; 585. Department of Molecular Medicine and Surgery, Karolinska Institutet, Stockholm, Sweden; 586. Center for Molecular Medicine, Karolinska University Hospital, Stockholm, Sweden; 587. Psychiatry, North East London NHS Foundation Trust, Ilford, UK; 588. Clinic for Psychiatry and Psychotherapy, University Hospital Cologne, Cologne, Germany; 589. Department of Psychiatry, Korea University College of Medicine, Seoul, South Korea; 590. Psychiatric and Neurodevelopmental Genetics Unit, Center for Genomic Medicine, Massachusetts General Hospital and Harvard Medical School, Boston, MA, USA; 591. HudsonAlpha Institute for Biotechnology, Huntsville, AL, USA; 592. Department of Human Genetics, McGill University, Montréal, Quebec, Canada; 593. Montreal Neurological Institute and Hospital, McGill University, Montréal, Quebec, Canada; 594. Division of Psychiatry, Centre for Clinical Brain Sciences, The University of Edinburgh, Edinburgh, UK; 595. Department of Psychiatry and Psychotherapy, University of Bonn, Bonn, Germany; 596. Clinical Biochemistry Laboratory, Attikon General Hospital, Medical School, National and Kapodistrian University of Athens, Athens, Greece; 597. Systems Genetics Working Group, Department of Genetics, Stellenbosch University, Stellenbosch, South Africa; 598. Genetic Cancer Susceptibility Group, International Agency for Research on Cancer, Lyon, France; 599. Department of Psychiatry, Massachusetts General Hospital, Boston, MA, USA; 600. Centre for Neuroimaging and Cognitive Genomics (NICOG), National University of Ireland Galway, Galway, Ireland; 601. Medical Faculty, School of Science and Technology, University Sarajevo, Sarajevo, Bosnia and Herzegovina; 602. Department of Psychiatry and Behavioral Sciences, Johns Hopkins University School of Medicine, Baltimore, MD, USA; 603. Department of Psychiatry, University of Oxford, Warneford Hospital, Oxford, UK; 604. Department of Psychiatry and Behavioral Sciences, Emory University School of Medicine, Atlanta, GA, USA; 605. Outpatient Clinic for Bipolar Disorder, Altrecht, Utrecht, the Netherlands; 606. Department of Psychiatry, Washington University in Saint Louis, Saint Louis, MO, USA; 607. Medicine, Psychiatry, Biomedical Informatics, Vanderbilt University Medical Center, Nashville, TN, USA; 608. Psychiatric Genetics Unit, Group of Psychiatry Mental Health and Addictions, Vall d´Hebron Research Institut (VHIR), Universitat Autònoma de Barcelona, Barcelona, Spain; 609. Faculty of Medicine, Department of Psychiatry, School of Health Sciences, University of Iceland, Reykjavik, Iceland; 610. Institute of Health and Wellbeing, University of Glasgow, Glasgow, UK; 611. Psychiatry and the Behavioral Sciences, University of Southern California, Los Angeles, CA, USA; 612. Mood Disorders, PsyQ, Rotterdam, the Netherlands; 613. SAMRC Unit on Risk and Resilience in Mental Disorders, Department of Psychiatry and Neuroscience Institute, University of Cape Town, Cape Town, South Africa; 614. Department of Environmental Epidemiology, Nofer Institute of Occupational Medicine, Lodz, Poland; 615. Neuroscience Research Australia, Sydney, New South Wales, Australia; 616. School

of Medical Sciences, University of New South Wales, Sydney, New South Wales, Australia; 617. Centro de Biología Molecular Severo Ochoa, Universidad Autónoma de Madrid and CSIC, Madrid, Spain; 618. Department of Psychiatry and Human Behavior, School of Medicine, University of California, Irvine, Irvine, CA, USA; 619. Psychiatry, Psychiatrisches Zentrum Nordbaden, Wiesloch, Germany; 620. Computational Sciences Center of Emphasis, Pfizer Global Research and Development, Cambridge, MA, USA; 621. Dalla Lana School of Public Health, University of Toronto, Toronto, Ontario, Canada; 622. Department of Psychological Medicine, Institute of Psychiatry, Psychology and Neuroscience, King's College London, London, UK; 623. South London and Maudsley NHS Foundation Trust, Bethlem Royal Hospital, Beckenham, UK; 624. Department of Clinical Sciences, Psychiatry, Umeå University Medical Faculty, Umeå, Sweden; 625. Department of Clinical Neuroscience, Centre for Psychiatry Research, Karolinska Institutet, Stockholm, Sweden; 626. NORMENT, KG Jebsen Centre for Psychosis Research, Division of Mental Health and Addiction, Institute of Clinical Medicine and Diakonhjemmet Hospital, University of Oslo, Oslo, Norway; 627. National Institute of Mental Health, Klecany, Czech Republic; 628. Institute of Environmental Medicine, Karolinska Institutet, Stockholm, Sweden; 629. Institute of Pulmonology, Russian State Medical University, Moscow, Russian Federation; 630. Department of Psychiatry, Melbourne Medical School, The University of Melbourne, Melbourne, Victoria, Australia; 631. The Florey Institute of Neuroscience and Mental Health, The University of Melbourne, Parkville, Victoria, Australia; 632. Université de Paris, INSERM, Optimisation Thérapeutique en Neuropsychopharmacologie, UMRS 1144, Paris, France; 633. APHP Nord, DMU Neurosciences, Département de Psychiatrie et de Médecine Addictologique, GHU Saint Louis-Lariboisière-Fernand Widal, Paris, France; 634. Psychiatry, University of Pennsylvania, Philadelphia, PA, USA; 635. Center for Statistical Genetics and Department of Biostatistics, University of Michigan, Ann Arbor, MI, USA; 636. Department of Biomedicine and the iSEQ Center, Aarhus University, Aarhus, Denmark; 637. Center for Genomics and Personalized Medicine, CGPM, Aarhus, Denmark; 638. School of Psychiatry, University of New South Wales, Sydney, New South Wales, Australia; 639. University of Queensland, Brisbane, Queensland, Australia; 640. Neuropsychiatric Genetics Research Group, Department of Psychiatry and Trinity Translational Medicine Institute, Trinity College Dublin, Dublin, Ireland; 641. 1st Department of Psychiatry, Eginition Hospital, National and Kapodistrian University of Athens, Athens, Greece; 642. Medical and Population Genetics, Broad Institute, Cambridge, MA, USA; 643. Department of Human Genetics, University of Chicago, Chicago, IL, USA; 644. Biometric Psychiatric Genetics Research Unit, Alexandru Obregia Clinical Psychiatric Hospital, Bucharest, Romania; 645. HUNT Research Center, Department of Public Health and Nursing, Faculty of Medicine and Health Sciences, Norwegian University of Science and Technology, Trondheim, Norway; 646. University of Western Australia, Nedlands, Western Australia, Australia; 647. Institute of Neuroscience and Physiology, University of Gothenburg, Gothenburg, Sweden; 648. Department of Psychiatry and Addiction Medicine, Assistance Publique - Hôpitaux de Paris, Paris, France; 649. Department of Medical and Molecular Genetics, King's College London, London, UK; 650. Neuroscience Therapeutic Area, Janssen Research and Development, LLC, Titusville, NJ, USA; 651. Cancer Epidemiology and Prevention, M. Sklodowska-Curie National Research Institute of Oncology, Warsaw, Poland; 652. SA MRC Unit on Risk and Resilience in Mental Disorders, Department of Psychiatry, Stellenbosch University, Stellenbosch, South Africa; 653. School of Psychology, The University of Queensland, Brisbane, Queensland, Australia; 654. Department of Psychiatry and Genetics Institute, University of Florida, Gainesville, FL, USA; 655. Research Institute, Lindner Center of HOPE, Mason, OH, USA; 656. Centre for Cognitive Ageing and Cognitive Epidemiology, University of Edinburgh, Edinburgh, UK; 657. Human Genetics Branch, Intramural Research Program, National Institute of Mental Health, Bethesda,

MD, USA; 658. Division of Mental Health and Addiction, University of Oslo, Institute of Clinical Medicine, Oslo, Norway; 659. Psychiatry, St Olavs University Hospital, Trondheim, Norway; 660. Psychosis Research Unit, Aarhus University Hospital - Psychiatry, Risskov, Denmark; 661. Munich Cluster for Systems Neurology (SyNergy), Munich, Germany; 662. University of Liverpool, Liverpool, UK; 663. Research/Psychiatry, Veterans Affairs San Diego Healthcare System, San Diego, CA, USA; 664. Division of Psychiatry, Haukeland Universitetssjukehus, Bergen, Norway; 665. Faculty of Medicine and Dentistry, University of Bergen, Bergen, Norway; 666. Department of Clinical Neuroscience and Center for Molecular Medicine, Karolinska Institutet at Karolinska University Hospital, Solna, Sweden; 667. Human Genetics and Computational Biomedicine, Pfizer Global Research and Development, Groton, CT, USA; 668. University of Melbourne, Melbourne, Victoria, Australia; 669. Department of Pathology, College of Medicine and Health Sciences, United Arab Emirates University, Al-Ain, United Arab Emirates; 670. Zayed Center of Health Sciences, United Arab Emirates University, Al-Ain, United Arab Emirates; 671. Psychiatry, Harvard Medical School, Boston, MA, USA; 672. Division of Clinical Research, Massachusetts General Hospital, Boston, MA, USA; 673. Department of Complex Trait Genetics, Center for Neurogenomics and Cognitive Research, Amsterdam Neuroscience, Vrije Universiteit Amsterdam, Amsterdam, the Netherlands; 674. Department of Clinical Genetics, Amsterdam Neuroscience, Vrije Universiteit Medical Center, Amsterdam, the Netherlands; 675. Department of Neurology and Neurosurgery, Faculty of Medicine, McGill University, Montreal, Quebec, Canada; 676. Department of Biomedical and NeuroMotor Sciences, University of Bologna, Bologna, Italy; 677. Department of Neuroscience, SUNY Upstate Medical University, Syracuse, NY, USA; 678. Psychiatric and Neurodevelopmental Genetics Unit (PNGU), Massachusetts General Hospital, Boston, MA, USA; 679. Faculty of Medicine, University of Iceland, Reykjavik, Iceland; 680. Department of Psychiatry, Hospital Namsos, Namsos, Norway; 681. Department of Neuroscience, Norges Teknisk Naturvitenskapelige Universitet Fakultet for Naturvitenskap og Teknologi, Trondheim, Norway; 682. Department of Genetics, University of North Carolina at Chapel Hill, Chapel Hill, NC, USA; 683. Department of Psychiatry, University of North Carolina at Chapel Hill, Chapel Hill, NC, USA; 684. Department of Psychiatry, McGill University, Montreal, Quebec, Canada; 685. Department of Psychiatry, Sankt Olavs Hospital Universitetssykehuset i Trondheim, Trondheim, Norway; 686. Clinical Institute of Neuroscience, Hospital Clinic, University of Barcelona, IDIBAPS, CIBERSAM, Barcelona, Spain; 687. Department of Psychology, Emory University, Atlanta, GA, USA; 688. Queensland Brain Institute, The University of Queensland, Brisbane, Queensland, Australia; 689. Psychiatry, Indiana University School of Medicine, Indianapolis, IN, USA; 690. Biochemistry and Molecular Biology, Indiana University School of Medicine, Indianapolis, IN, USA; 691. Department of Genetic Epidemiology in Psychiatry, Central Institute of Mental Health, Medical Faculty Mannheim, Heidelberg University, Mannheim, BW, DE; 692. Department of Psychiatry and Psychotherapy, Charité – Universitätsmedizin Berlin, Berlin, BE, DE; 693. Department of Psychiatry, Massachusetts General Hospital, Boston, MA, US; 694. Department of Psychiatry, Harvard Medical School, Boston, MA, US; 695. Human Genetics, Wellcome Sanger Institute, Hinxton, UK; 696. NIHR Maudsley Biomedical Research Centre, King's College London, London, UK; 697. Department of Clinical Medicine, Aarhus University, Aarhus, DK; 698. Department of Medical Biochemistry and Biophysics, Karolinska Institutet, Stockholm, SE; 699. National Centre for Register-based Research, Aarhus University, Aarhus, DK; 700. Department of Pediatric Neurology, Charité – Universitätsmedizin Berlin, Berlin, BE, DE; 701. MRC Integrative Epidemiology Unit, University of Bristol, Bristol, UK; 702. Department of Psychiatry and Neuropsychology, School for Mental Health and Neuroscience, Maastricht University Medical Centre, Maastricht, NL; 703. Institute for Molecular Bioscience, University of Queensland, Brisbane, QLD, AU;

704. Maurice Wohl Clinical Neuroscience Institute, Department of Basic and Clinical Neuroscience, King's College London, London, UK; 705. Veterans Affairs Connecticut Healthcare System, West Haven, CT, US; 706. Department of Psychiatry, Yale University School of Medicine, New Haven, CT, US; 707. Department of Psychiatry, University of Munich, Munich, BY, DE; 708. Institute of Psychiatric Phenomics and Genomics, University of Munich, Munich, BY, DE; 709. Department of Psychiatry and Psychotherapy, University Hospital Bonn, Medical Faculty, University of Bonn, Bonn, DE; 710. Mental Health and Neuroscience, QIMR Berghofer Medical Research Institute, Brisbane, QLD, AU; 711. Department of Medical Epidemiology and Biostatistics, Karolinska Institutet, Stockholm, SE; 712. Old Age Psychiatry, King's College London, London, UK; 713. Department of Medicine, Division of Genetic Medicine, Vanderbilt University Medical Center, Nashville, TN, US; 714. Department of Psychiatry and Psychotherapy, Fliedner Klinik Berlin, Berlin, BE, DE; 715. Centre for Integrated Register-based Research, Aarhus University, Aarhus, DK; 716. iPSYCH, The Lundbeck Foundation Initiative for Integrative Psychiatric Research, Aarhus, DK; 717. Department of Psychiatry, Psychosomatic Medicine and Psychotherapy, Goethe University Frankfurt - University Hospital, Frankfurt am Main, DE; 718. Discipline of Psychiatry, University of Adelaide, Adelaide, SA, AU; 719. Department of Epidemiology, Columbia University Mailman School of Public Health, New York, NY, US; 720. Department of Biomedicine and Centre for Integrative Sequencing, iSEQ, Aarhus University, Aarhus, DK; 721. Center for Genomics and Personalized Medicine, Aarhus University, Aarhus, DK; 722. Department of Clinical Neuroscience, Karolinska Institutet,, SE; 723. Department of Neurology, Klinikum rechts der Isar, Technical University of Munich, Munich, BY, DE; 724. Department of Translational Research in Psychiatry, Max Planck Institute of Psychiatry, Munich, BY, DE; 725. Institute for Translational Psychiatry, University of Münster, Münster, NRW, DE; 726. Department of Mental Disorders, Norwegian Institute of Public Health, Oslo, NO; 727. PROMENTA Research Center, Department of Psychology, University of Oslo, Oslo, NO; 728. Research and Development, Takeda Pharmaceutical Company Limited, Cambridge, MA, US; 729. Faculty of Health and Life Sciences, University of Exeter, Exeter, UK; 730. Novo Nordisk Center for Protein Research, Department of Health Sciences, University of Copenhagen, Copenhagen, DK; 731. Department of Psychiatry, Amsterdam Public Health and Amsterdam Neuroscience, Amsterdam UMC, Vrije Universiteit Amsterdam, Amsterdam, NL; 732. Morphology and Genetics, Universidade Federal de Sao Paulo, Sao Paulo, SP, BR; 733. Department of Psychiatry and Behavioral Sciences, Emory University School of Medicine, Atlanta, GA, US; 734. Faculty of Nursing and Health Sciences, NORD University, Levanger, NO; 735. Department of Mental Health, Faculty of Medicine and Health Sciences, Norwegian University of Science and Technology (NTNU), Trondheim, TRD, NO; 736. deCODE Genetics / Amgen, Reykjavik, IS; 737. K. G. Jebsen Center for Genetic Epidemiology, Department of Public Health and Nursing, Faculty of Medicine and Health Sciences, Norwegian University of Science and Technology (NTNU), Trondheim, TRD, NO; 738. Department of Research and Innovation, Division of Clinical Neuroscience, Oslo University Hospital, Oslo, NO; 739. Institute of Clinical Medicine, Faculty of Medicine, University of Oslo, Oslo, NO; 740. Institute of Environmental Medicine, Unit of Integrative Epidemiology, Karolinska Institutet, Stockholm, SE; 741. Department of Psychiatry and Psychotherapy, Charité – Universitätsmedizin Berlin, Berlin, DE; 742. Department of Clinical Immunology, Roskilde University/Næstved Hospital, Roskilde, DK; 743. Department of Clinical Immunology, Odense University Hospital, Odense, DK; 744. Department of Psychiatry, Psychotherapy and Psychosomatics, Brandenburg Medical School Theodor Fontane, Neuruppin, BB, DE; 745. Department of Psychiatry and Psychotherapy, Gender Research in Medicine, Institute of Sexology and Sexual Medicine, Charité – Universitätsmedizin Berlin, Berlin, BE, DE; 746. iPSYCH, The Lundbeck Foundation Initiative for Integrative Psychiatric Research,

Copenhagen, DK; 747. Center for Neonatal Screening, Department for Congenital Disorders, Statens Serum Institut, Copenhagen, DK; 748. Child Health Research Centre, University of Queensland, Brisbane, QLD, AU; 749. Centre for Medical Informatics, Usher Institute, University of Edinburgh, Edinburgh, UK; 750. Centre for Genomic & Experimental Medicine, Institute for Genetics and Cancer, University of Edinburgh, Edinburgh, UK; 751. Department of Psychiatry and Mental Health, University of Cape Town, Cape Town, SA; 752. Department of Psychiatry, Lausanne University Hospital and University of Lausanne, Prilly, VD, CH; 753. Instituto de Investigación Biosanitaria ibs.GRANADA, Granada, ES; 754. Department of Psychiatry, Faculty of Medicine and Institute of Neurosciences, Biomedical Research Centre (CIBM), University of Granada, Granada, ES; 755. Université de Paris Cité, INSERM U1266, Institute of Psychiatry and Neuroscience of Paris, GHU Paris Psychiatry and Neuroscience, Paris, FR; 756. Translational Biology, Biogen, Cambridge, MA, US; 757. Nuffield Department of Population Health, University of Oxford, Oxford, UK; 758. MRC Population Health Research Unit, University of Oxford, Oxford, UK; 759. Institute of Neuroscience and Medicine (INM-1), Research Center Juelich, Juelich, DE; 760. Human Genomics Research Group, Department of Biomedicine, University of Basel, Basel, CH; 761. Institute of Medical Genetics and Pathology, University Hospital Basel, University of Basel, Basel, CH; 762. Nic Waals Institute, Lovisenberg Diakonale Hospital, Oslo, NO; 763. Centre for Advanced Imaging, University of Queensland, Saint Lucia, QLD, AU; 764. Psychological Medicine, Cardiff University, Cardiff, WLS, UK; 765. Centre for Neuropsychiatric Genetics and Genomics, Cardiff University, Cardiff, WLS, UK; 766. The Lothian Birth Cohorts, University of Edinburgh, Edinburgh, UK; 767. Department of Biological Psychology & Amsterdam Public Health Research Institute, Vrije Universiteit Amsterdam, Amsterdam, NL; 768. Institute of Human Genetics, University of Bonn, School of Medicine & University Hospital Bonn, Bonn, DE; 769. Department of Child and Adolescent Psychiatry, Psychosomatics and Psychotherapy, University Hospital Essen, Unversity of Duisburg-Essen, Duisburg, DE; 770. MRC Centre for Environment and Health, School of Public Health, Imperial College London, London, UK; 771. Imperial College Dementia Research Institute, Imperial College London, London, UK; 772. Department of Psychiatry and Behavioral Sciences, Johns Hopkins University School of Medicine, Baltimore, MD, US; 773. Department of Psychiatry and Psychotherapy, Research Group Stress Related Disorders, Central Institute of Mental Health, Medical Faculty Mannheim, Heidelberg University, Mannheim, BW, DE; 774. Department of Clinical Immunology, Copenhagen University Hospital, Rigshospitalet, Copenhagen, CPH, DK; 775. Department of Clinical Immunology, Aarhus University Hospital, Aarhus, DK; 776. Department of Psychiatry, Istanbul University, Istanbul, TR; 777. Department of Medical Genetics, Oslo University Hospital, Oslo, OSL, NO; 778. NORMENT, Department of Clinical Science, University of Bergen, Bergen, NO; 779. Virginia Institute for Psychiatric & Behavioral Genetics, Virginia Commonwealth University, Richmond, VA, US; 780. Psychiatry Department / Huntsman Mental Health Institute, University of Utah School of Medicine, Salt Lake City, UT, US; 781. Center for Genomic Research, University of Utah School of Medicine, Salt Lake City, UT, US; 782. Department of Psychiatry and Psychotherapy, Medical Center, University of Freiburg, Faculty of Medicine, University of Freiburg, Freiburg, DE; 783. Division of Mental Health Care, St. Olavs Hospital, Trondheim University Hospital, Trondheim, TRD, NO; 784. Department of Psychiatry, Sørlandet Hospital, Kristiansand, AG, NO; 785. Center for Genomic Medicine, Massachusetts General Hospital, Boston, MA, US; 786. Centre for Addiction and Mental Health, Toronto, ON, CA; 787. Department of Psychiatry and Psychotherapy, University Medical Center of the Johannes Gutenberg University Mainz, Mainz, DE; 788. Mental Health Center Copenhagen, Mental Health Services Capital Region of Denmark, Copenhagen, DK; 789. Faculty of Health Science, Department of Clinical Medicine, University of Copenhagen, Copenhagen, DK; 790.

Department of Biomedical and Neuromotor Sciences, University of Bologna, Bologna, IT; 791. Michigan Neuroscience Institute, University of Michigan, Ann Arbor, MI, US; 792. Wolfson Institute of Population Health, Queen Mary University of London, London, UK; 793. School of Computing and Mathematical Sciences, University of Leicester, Leicester, UK; 794. Department of Epidemiology and Biostatistics, Imperial College London, London, UK; 795. Discipline of Psychiatry, School of Medicine, Trinity College Dublin, Dublin, IE; 796. School of Engineering, University of Iceland, Reykjavik, IS; 797. Danish Headache Centre, Department of Neurology, Rigshospitalet, Glostrup, DK; 798. Faculty of Medicine, Department of Psychiatry, University of Iceland, Reykjavik, IS; 799. Department of Psychiatry, University of Groningen, University Medical Center Groningen, Groningen, NL; 800. MRC Human Genetics Unit, Institute for Genetics and Cancer, University of Edinburgh, Edinburgh, UK; 801. Brain and Mind Centre, University of Sydney, Sydney, NSW, AU; 802. Department of Epidemiology Research, Statens Serum Institut, Copenhagen, DK; 803. Interfaculty Institute for Genetics and Functional Genomics, Department of Functional Genomics, University Medicine Greifswald, Greifswald, MV, DE; 804. Roche Pharmaceutical Research and Early Development, Pharmaceutical Sciences, Roche Innovation Center Basel, F. Hoffmann-La Roche Ltd, Basel, CH; 805. SleepWell Research Program and Department of Psychology and Logopedics, University of Helsinki, Helsinki, FI; 806. Blizard Institute, Barts and the London School of Medicine and Dentistry, Queen Mary University of London, London, UK; 807. Max Planck Institute of Psychiatry, Munich, BY, DE; 808. Department of Psychiatry, University of Helsinki, Helsinki, FI; 809. 23andMe Research Team, 23andMe, Inc., Sunnyvale, CA, US; 810. Department of Psychological Medicine, University of Worcester, Worcester, UK; 811. Institution of Neuroscience and Physiology, University of Gothenburg, Gothenburg, SE; 812. Department of Psychiatry and Psychotherapy, Medical University of Vienna, Vienna, AT; 813. Department of Psychiatry, Virginia Commonwealth University, Richmond, VA, US; 814. Health Care Policy, Harvard Medical School, Boston, MA, US; 815. Department of Psychiatry, University of Toronto, Toronto, ON, CA; 816. Department of Genetics, Rutgers University, Piscataway, NJ, US; 817. Department of Psychiatry, Perelman School of Medicine, University of Pennsylvania, Philadelphia, PA, US; 818. Mental Illness Research, Education and Clinical Center, Crescenz VA Medical Center, Philadelphia, PA, US; 819. Estonian Genome Centre, Institute of Genomics, University of Tartu, Tartu, EE; 820. Department of Women's and Children's Health, Uppsala University, Uppsala, SE; 821. Department of Epidemiology and Health Systems, Center for Primary Care and Public Health, Lausanne, VD, CH; 822. Swiss Institute of Bioinformatics, Lausanne, VD, CH; 823. Department of Computational Biology, University of Lausanne, Lausanne, VD, CH; 824. Institute for Molecular Medicine Finland - FIMM, University of Helsinki, Helsinki, FI; 825. Department of Psychiatry, Washington University School of Medicine in St. Louis, St. Louis, MO, US; 826. Psychiatry, Veterans Affairs Connecticut Healthcare System, West Haven, CT, US; 827. Department of Psychiatry, Yale University, New Haven, CT, US; 828. Department of Epidemiology and Biostatistics, School of Public Health, Peking University, Beijing, CN; 829. Peking University Center for Public Health and Epidemic Preparedness & Response, Peking University, Beijing, CN; 830. Mental Health, NHS 24, Glasgow, UK; 831. Royal Edinburgh Hospital, NHS Lothian, Edinburgh, UK; 832. Department of Human and Molecular Genetics, Virginia Commonwealth University, Richmond, VA, USA; 833. Virginia Institute for Psychiatric and Behavioral Genetics, Virginia Commonwealth University, Richmond, VA, USA; 834. Department of Psychiatry and Psychotherapy, University of Bonn, Bonn, DE; 835. Center for Translational and Computational Neuroimmunology, Columbia University Medical Center, New York, NY, US; 836. Institute for Translational Neuroscience, University of Münster, Münster, NRW, DE; 837. Novo Nordisk Foundation Center for Basic Metabolic Research, Faculty of Health Science, Copenhagen

University, Copenhagen, DK; 838. Department of Nursing, Faculty of Health Sciences and Institute of Neurosciences, Biomedical Research Centre (CIBM), University of Granada, Granada, ES; 839. School of Public Health, University of Queensland, Brisbane, QLD, AU; 840. DZHK (German Centre for Cardiovascular Research), Partner Site Greifswald, Greifswald, MV, DE; 841. Institute of Clinical Chemistry and Laboratory Medicine, University Medicine Greifswald, Greifswald, MV, DE; 842. Department of Psychiatry, University of Marburg, Marburg, DE; 843. Department of Clinical Immunology, Aalborg University Hospital, Aalborg, DK; 844. Department of Epidemiology, University of Groningen, University Medical Center Groningen, Groningen, NL; 845. Department of Health, Science and Technology, Aalborg University, Aalborg, DK; 846. Centre for Integrative Sequencing, iSEQ, Aarhus University, Aarhus, DK; 847. Population Health, QIMR Berghofer Medical Research Institute, Brisbane, QLD, AU; 848. The Fraser Institute, Faculty of Medicine, University of Queensland, Brisbane, QLD, AU; 849. Humus, Reykjavik, IS; 850. Department of Clinical Medicine, University of Copenhagen, Copenhagen, CPH, DK; 851. Department of Population Health Sciences, University of Leicester, Leicester, UK; 852. Department of Psychiatry, Universidade Federal de Sao Paulo, Sao Paulo, SP, BR; 853. Department of Psychiatry, Rutgers University, Piscataway, NJ, US; 854. Department of Clinical Immunology, Zealand University Hospital, Køge, DK; 855. Department of Psychiatry and Behavioral Sciences, SUNY Downstate Health Sciences University, Brooklyn, NY, US; 856. NMD Pharma, Lexington, MA, US; 857. Psychiatry, University of Pittsburgh Medical Centre, Pittsburgh, PA, US; 858. Psychiatry, Universidade Federal do Rio Grande do Sul, Porto Alegre, BR; 859. Department of Psychiatry, University Medical Center Groningen, Groningen, NL; 860. Research School of Behavioural and Cognitive Neurosciences (BCN), University of Groningen, Groningen, NL; 861. Institute of Biological Psychiatry, Mental Health Center Sct. Hans, Mental Health Services Capital Region of Denmark, Copenhagen, DK; 862. Neurogenomics Division, The Translational Genomics Research Institute (TGEN), Phoenix, AZ, US; 863. Division of Cancer Epidemiology and Genetics, National Cancer Institute, Bethesda, MD, US; 864. School of Medicine and Dentistry, James Cook University, Townsville, QLD, AU; 865. Division of Mental Health and Addiction, Oslo University Hospital, Oslo, OSL, NO; 866. NORMENT, Institute of Clinical Medicine, University of Oslo, Oslo, OSL, NO; 867. Division of Population Health and Genomics, Ninewells Hospital and School of Medicine, University of Dundee, Dundee, UK; 868. Faculty of Medicine, University of Iceland, Reykjavik, IS; 869. SAMRC Unit on Risk & Resilience in Mental Disorders, Department of Psychiatry and Mental Health, University of Cape Town, Cape Town, SA; 870. Department of Psychiatry and Psychotherapy, University of Marburg, Marburg, HE, DE; 871. Department of Psychiatry, Psychotherapy and Psychosomatics, Dr. Fontheim Mentale Gesundheit, Liebenburg, DE; 872. Institute of Epidemiology and Social Medicine, University of Münster, Münster, NRW, DE; 873. Institute for Community Medicine, University Medicine Greifswald, Greifswald, MV, DE; 874. Wolfson Centre for Young People's Mental Health, Division of Psychological Medicine and Clinical Neurosciences, Cardiff University, Cardiff, WLS, UK; 875. Department of Biological Sciences, Purdue University, West Lafayette, IN, US; 876. Imperial College BHF Centre for Research Excellence, Imperial College London, London, UK; 877. Genomics Program, University of South Florida College of Public Health, Tampa, FL, US; 878. Department of Internal Medicine, Erasmus University Medical Center Rotterdam, Rotterdam, NL; 879. Management Section, Statens Serum Institut, Copenhagen, DK; 880. Department of Clinical Medicine, University of Copenhagen, Copenhagen, DK; 881. Xperimed LLC, Basel, CH; 882. Psychiatry, USUHS, Bethesda, US; 883. Department of Psychiatry and Psychotherapy, University Medicine Greifswald, Greifswald, MV, DE; 884. Department of Psychiatry, Leiden University Medical Center, Leiden, NL; 885. Department of Epidemiology and Population Health, Albert Einstein College of Medicine, Bronx, NY, US; 886. Department of Psychiatry, Columbia

University College of Physicians and Surgeons, New York, NY, US; 887. Division of Epidemiology, New York State Psychiatric Institute, New York, NY, US; 888. Department of Neurology, Oslo University Hospital, Oslo, NO; 889. HUNT All-In Psychiatry; 890. China Kadoorie Biobank Collaborative Group; 891. Genes & Health Research Team; 892. KG Jebsen Centre for Neurodevelopmental Research, University of Oslo, Oslo, OSL, NO; 893. Department of Psychiatry, University of Melbourne, Melbourne, VIC, AU; 894. Florey Institute of Neuroscience and Mental Health, University of Melbourne, Melbourne, VIC, AU; 895. Department of Psychiatry, University of Münster, Münster, NRW, DE; 896. Computational Health Centre, Helmholtz Zentrum München, Neuherberg, DE; 897. School of Medicine, Technical University of Munich, Munich, BY, DE; 898. Helmholtz Pioneer Campus, Helmholtz Zentrum München, Neuherberg, DE; 899. Department of Psychiatry, University of Vermont, Burlington, VT, US; 900. Department of Cellular, Computational and Integrative Biology, Università degli Studi di Trento, Trento, IT; 901. Department of Psychiatry, Psychosomatics and Psychotherapy, Julius-Maximilians-Universität Würzburg, Würzburg, DE; 902. Department of Genetics, Department of Neuroscience, Yale University School of Medicine, New Haven, CT, US; 903. Psychiatry, Kaiser Permanente Northern California, San Francisco, CA, US; 904. K. G. Jebsen Center for Genetic Epidemiology, Department of Public Health and Nursing, Faculty of Medicine and Health Sciences, Norwegian University of Science and Technology (NTNU), Trondheim, NO; 905. HUNT Research Center, Department of Public Health and Nursing, Faculty of Medicine and Health Sciences, Norwegian University of Science and Technology (NTNU), Trondheim, NO; 906. Department of Research, Innovation and Education, St. Olavs Hospital, Trondheim University Hospital, Trondheim, NO; 907. NIHR Leicester Biomedical Research Centre, Glenfield Hospital, Leicester, UK; 908. Pathophysiology of Psychiatric Diseases, INSERM, Univ Paris Cité, GHU Paris, Paris, FR; 909. Department of Psychiatry & Behavioral Sciences, Stanford University, Stanford, CA, US; 910. Neuroscience Therapeutic Area, Janssen Research and Development, LLC, Titusville, NJ, US; 911. Second Opinion Outpatient Clinic, GGNet Mental Health, Warnsveld, NL; 912. Child and Youth Mental Health Service, Children's Health Queensland Hospital and Health Service, Brisbane, QLD, AU; 913. Psychosis Research Unit, Aarhus University Hospital-Psychiatry, Aarhus, DK; 914. Department of Pharmacology & Toxicology, University of Toronto, Toronto, ON, CA; 915. Munich Cluster for Systems Neurology (SyNergy), Munich, BY, DE; 916. Human Genetics and Computational Biomedicine, Pfizer Global Research and Development, Groton, CT, US; 917. Centre for Quantitative Health, Massachusetts General Hospital, Boston, MA, US; 918. Child and Adolescent Psychiatry, Amsterdam UMC, Vrije Universiteit Amsterdam, Amsterdam, NL; 919. Complex Trait Genetics, Vrije Universiteit Amsterdam, Amsterdam, NL; 920. Wolfson Centre for Young People's Mental Health, Division of Psychological Medicine and Clinical Neurosciences, Cardiff University, Cardiff, UK; 921. Department of Biochemistry and Molecular Biology II, Faculty of Pharmacy and Institute of Neurosciences, Biomedical Research Centre (CIBM), University of Granada, Granada, ES; 922. Department of Clinical Neuroscience, Karolinska Institutet, Stockholm, SE; 923. Division of Research, Kaiser Permanente Northern California, Oakland, CA, US; 924. Department of Psychiatry, University of Michigan, Ann Arbor, MI, US; 925. Stanley Center for Psychiatric Research, Broad Institute of MIT and Harvard, Cambridge, MA, US; 926. Psychiatric and Neurodevelopmental Genetics Unit, Massachusetts General Hospital, Boston, MA, US; 927. Psychiatry, UCSD School of Medicine, La Jolla, CA, US; 928. Public Health, UCSD School of Public Health, La Jolla, CA, US; 929. Departments of Genetics and Psychiatry, University of North Carolina at Chapel Hill, Chapel Hill, NC, US; 930. Child and Adolescent Psychiatry, Erasmus University Medical Center Rotterdam, Rotterdam, NL; 931. Social and Behavioral Science, Harvard T.H. Chan School of Public Health, Boston, MA, US; 932. Psychiatry, Dalhousie University, Halifax, NS, CA; 933. Queensland Brain

Institute, University of Queensland, Brisbane, QLD, AU; 934. Department of Medical & Molecular Genetics, King's College London, London, UK; 935. Institute for Genomics and Cancer, University of Edinburgh, Edinburgh, UK

Supplementary Note

1. Supplementary results of the subsection 'Simulations'

We performed six secondary analyses. First, we compared DDx-PRS to Marginal-PRS across all 6 pairs of diagnostic categories (SCZ vs. BIP, SCZ vs. MDD, BIP vs. MDD, SCZ vs. control, BIP vs. control, MDD vs. control), instead of 4 pairs of one diagnostic category vs. rest. Analogous to Figure 1, DDx-PRS attained substantially better calibration and slightly higher accuracy than Marginal-PRS (Supplementary Figure 1 and Supplementary Table 5). We also note that, despite the large genetic correlation between SCZ and BIP (0.70), there is significant potential to distinguish SCZ vs. BIP (AUC 0.632±0.002). Second, we assessed the calibration of DDx-PRS and Marginal-PRS using calibration slope[1], instead of ICI. Analogous to Figure 1a, DDx-PRS attained substantially better calibration than Marginal-PRS (e.g. 0.073±0.004 vs. 0.151±0.006 for mean absolute difference of slope from 1; Supplementary Figure 2 and Supplementary Table 6). Third, we repeated our simulations while doubling the GWAS training sample size. Once again, DDx-PRS attained substantially better calibration than Marginal-PRS (with ICI values similar to Figure 1a) and slightly higher accuracy than Marginal-PRS (with AUC values larger than Figure 1b for both methods, as expected) (Supplementary Figure 3 and Supplementary Table 7). Fourth, we repeated our simulations while decreasing the genetic correlation for SCZ-BIP from 0.7 to 0.3. Once again, we observed that DDx-PRS attained better calibration and slightly higher accuracy than Marginal-PRS, with both methods attaining higher accuracy than in our main simulations due to better discrimination between SCZ and BIP (Supplementary Figure 4 and Supplementary Table 8). Fifth, we repeated our simulations without sample overlap among controls of SCZ, BIP and MDD. We determined that our results were largely unchanged (Supplementary Figure 5 and Supplementary Table 9). Sixth, we repeated our simulations with unbalanced test sample sizes for the four diagnostic categories (using correctly specified prior probabilities). We determined that DDx-PRS performed well with similar conclusions for several test sample size ratios, including for case-only analyses with ratios of 33.3% for SCZ/BIP/MDD (and 0% for controls; Supplementary Figure 6a-k and Supplementary Figure 7). Seventh, we repeated our simulations with unbalanced test sample sizes using misspecified prior probabilities. We determined that DD-PRS and Marginal-PRS results were miscalibrated in the expected direction (Supplementary Figure 6l-s and Supplementary Figure 7a), and that the multi-class MCC was impacted more than the mean AUC (mean of four binary AUCs; Supplementary Figure 7b).

2. Supplementary results of the subsection 'DDx-PRS distinguishes SCZ, BIP, MDD and controls in PGC data'

We performed five secondary analyses. First, we repeated our analyses while analysing the four test cohorts separately. We observed similar results for all four test cohorts individually as for the mean results in Figure 3 (Supplementary Figure 8). Second, we compared DDx-PRS to Marginal-PRS across all 6 pairs of diagnostic categories (SCZ vs. BIP, SCZ vs. MDD, BIP vs. MDD, SCZ vs. control, BIP vs. control, MDD vs. control), instead of 4 pairs of each diagnostic category vs. rest. Analogous to Figure 3, DDx-PRS attained substantially better calibration and similar accuracy as Marginal-PRS (Supplementary Figure 9 and Supplementary Table 12). We also observed significant potential to distinguish SCZ vs. BIP (AUC 0.633±0.003; Supplementary Table 12), despite the large genetic correlation between SCZ and BIP (0.70). Third, we assessed the calibration of DDx-PRS and Marginal-PRS using calibration slope[1], instead of ICI. Analogous to Figure 3, DDx-PRS attained better calibration than Marginal-PRS (Supplementary Figure 10 and Supplementary Table 13). Fourth, we repeated our analyses at different QC thresholds (less strict QC for SNPs and/or stricter QC for individuals; see Methods). Results were similar to our primary results reported in Figure 3 (Supplementary Figure 11 and Supplementary Table 14). Fifth, we repeated our analyses with unbalanced test sample sizes for the four diagnostic categories (using correctly specified prior probabilities). We determined that DDx-PRS remained well-calibrated, Marginal-PRS remained poorly calibrated, and mean AUC and MCC remained comparable for DDx-PRS and Marginal (Supplementary Figure 12). Specifically, when considering case-only analyses with test sample size ratios of 33.3% for SCZ/BIP/MDD (and 0% for controls), results remained qualitatively similar with only a slightly smaller mean AUC (0.65 for SCZ vs. BIP/MDD, 0.61 for BIP vs. SCZ/MDD and 0.64 for MDD vs. SCZ/BIP), compared to test sample size ratios of 25% for SCZ/BIP/MDD/control.

3. Supplementary results of the subsection 'Comparison of DDx-PRS to methods that require tuning data'

We performed four secondary analyses. First, we compared DDx-PRS to DDx-PRS-tuned and Direct-tuned in simulations (Methods). We determined that DDx-PRS, DDx-PRS-tuned and Direct-tuned yielded comparable results (Supplementary Figure 13 and Supplementary Table 16). Second, we repeated our PGC analyses with smaller tuning data sets (N_{tuning}=4x100 and N_{tuning}=4x50 respectively). We determined that the results were minimally affected by smaller tuning sample size (Supplementary Figure 14 and Supplementary Table 17). Third, we compared DDx-PRS to DDx-PRS-tuned and Direct-tuned using within-cohort tuning (N_{tuning}=4x200), in which the tuning data set is from the same cohort as test data (although this type of tuning data is much less likely to be available in

clinical settings). We observed minimal performance improvement of DDx-PRS-tuned and Direct-tuned using within-cohort tuning, and that results for DDx-PRS, DDx-PRS-tuned and Direct-tuned remained comparable (Supplementary Figure 15 and Supplementary Table 18). Fourth, we repeated our analyses with unbalanced test sample sizes for the four diagnostic categories (using correctly specified prior probabilities). We determined that the performance of DDx-PRS, DDx-PRS-tuned and Direct-tuned remained very similar for all test sample size ratios considered, including for case-only analyses with test sample size ratios of 33.3% for SCZ/BIP/MDD (and 0% for controls) (Supplementary Figure 16).

4. Supplementary results of the subsection 'Projections of clinical utility at larger training sample sizes'

We performed four secondary analyses. First, we repeated the above analyses for DDx-PRS-tuned and Direct-tuned. We determined that results were similar to DDx-PRS (Supplementary Figure 17 and Supplementary Table 21). (We did not perform these analyses for Marginal-PRS, as this method is not well-calibrated; Figure 1a and Figure 3a.) Second, we performed simulations at the exact empirical values of % of SNP-heritability captured by case-control PRS, yielding results that were close to the empirical results (Supplementary Figure 18 and Supplementary Table 22). Third, we repeated the above analyses for case-only analyses with test sample size ratios of 33.3% for SCZ/BIP/MDD (and 0% for controls; using correctly specified prior probabilities). We observed that the results were qualitatively similar (Supplementary Figure 19). Fourth, we repeated our SES analyses with SES assessed via income or educational attainment, two SES metrics with different genetic correlations with SCZ/BIP/MDD37. We observed similar results compared to the Townsend deprivation index (Supplementary Figure 20).

5. Supplementary discussion of 'the potential clinical utility of DDx-PRS'

(This section repeats content from the *Discussion* section, to ensure that it is self-contained.) The potential clinical utility of DDx-PRS is subject to several important considerations. First, refining diagnostic probability for a diagnostic category in differential diagnosis can be clinically useful; for example, for SCZ/BIP/MDD/control, increasing probability from a prior baseline risk of 25% to genetically informed risk of ~50% may guide clinicians in selecting interventions such as preventive psychotherapy, targeted follow-up, and psychoeducation pertaining to risk factors and early symptoms, which can provide important benefits[2,3]. (In particular, simulations suggest that DDx-PRS has greater potential to distinguish SCZ/BIP/MDD/control than socioeconomic status; Figure 6.) However, we caution that a diagnostic probability of ~50% for psychiatric disorders would generally not justify starting psychiatric medication due to significant potential side effects[4,5]. Second, case-

control PRS explain less than 8% of variance in risk of SCZ in the population[6] (6-13% using PRS-CS[7]; Supplementary Table 23), which limits the clinical utility of using PRS alone for decision making. Indeed, the potential clinical utility is likely restricted to individuals at moderate to high levels of disorder risk (e.g. ≥25%) (Figure 6); increasing diagnostic category probability from the prior baseline population risk of 1% to genetically informed risk of 3% would not be clinically meaningful[8]. On the other hand, while several challenges still need to be overcome, DDx-PRS might potentially have utility in the future for patients at ultra-high risk (UHR)[9,10] for psychosis (as occurring in SCZ), who are already systematically identified in mental healthcare[11] and at increased risk not only for psychosis (long-term risk 15-38%)[12–16] but also for other disorders[10] (one of the challenges to overcome will be the varying[12–16] and declining risk that has been observed in subsequent studies over the years[13]). Third, other examples of potential clinical utility could be (i) to distinguish between schizophrenia, bipolar disorder, and major depressive disorder with psychotic features in patients who present with a psychosis[17] by specifying a lower prior control probability, or (ii) to distinguish between bipolar disorder and major depressive disorder in patients who present with a depressive episode by specifying lower SCZ and control prior probabilities (DDx-PRS performs well across different test sample size ratios; Figures S7,S12,S16). Fourth, ethical issues should carefully be considered[18–20]. Specifically, not all individuals may wish to know their risk for SCZ/BIP/MDD: informed consent should be thoroughly provided by trained professionals prior to testing, and opt-out of genetic testing should be made very easy (if not the default). Fifth, we emphasize that the potential for clinical utility is contingent on external validation of our results, which is challenging given that there is no laboratory gold standard for validating psychiatric diagnoses, and that current DSM/ICD classifications are not pathophysiological entities[21–24]. Sixth, since the PGC samples are selected for genetic research, they may not represent real-world patients, and thus extensive validation and testing are needed before DDx-PRS can be applied in clinical settings. Although our results validated predictive performance in fully independent test samples within the PGC, we emphasize that future clinical intervention studies will be needed to ultimately determine clinical utility. Sixth, we note that, although we have emphasized psychiatric disorders in this study, DDx-PRS has potential for clinical utility in other settings; for example, previous studies have emphasized the importance of distinguishing inflammatory arthritis-causing diseases[25], latent autoimmune diabetes vs. type 2 diabetes[26], and asthma vs. COPD vs. asthma-COPD overlap syndrome[27] (leveraging the advantages of case-only analyses; Supplementary Figure 12).

6. Supplementary discussion of limitations

(This section repeats content from the *Discussion* section, to ensure that it is self-contained.) We note several limitations of this study. First, there is no laboratory gold standard for validating psychiatric

diagnoses, and current DSM/ICD classifications are not pathophysiological entities[21–24] (in contrast to e.g. inflammatory arthritis-causing diseases[25]), which could impact external validation. However, our results validated predictive performance in fully independent test samples within the PGC (Figure 3-6). We note that even if PGC classifications only partially capture true neurobiological differences, genetic predictions will still capture these neurobiological differences, albeit with reduced accuracy. Ultimately, future clinical intervention studies will be needed to establish clinical utility. Second, we chose to focus here on SCZ/BIP/MDD, because they have the largest available training sample sizes and often represent diagnostic challenges. However, relevant diagnostic distinctions often include additional disorders beyond SCZ/BIP/MDD, e.g. schizoaffective disorder (SZA) (which we considered as a subset of SCZ[6]). Similarly, there exist clinically-relevant subtypes of BIP (e.g., with/without psychosis) and MDD (e.g., with/without psychosis), which we do not consider here as these subtypes were pooled into single case groups in ref.[28] and ref.[29,30]. We note that DDx-PRS can flexibly include additional disorders/subtypes in its prediction framework as GWAS training sample sizes increase. Third, we acknowledge that for many clinical scientists the most pressing need may be to eliminate DSM/ICD classifications untethered from neurobiology[21–24] as they may be perceived as not benefitting many patients. Another pressing need may be separating primary defining characteristics of, for instance, SCZ (psychosis/reality distortions) from differential diagnosis, as these are not disorder-specific (psychosis/reality distortions can also present in the context of MDD and BIP). However, at the same time, we believe the rich genetic data on N>1,000,000 also presents opportunities on which we ought to capitalize. Fourth, the framework of DDx-PRS assumes that controls are screened for the absence of SCZ/BIP/MDD, whereas screening strategies in the PGC cohorts varied (ranging from no screening to screening for MDD, BIP, non-affective psychosis and substance use disorders)[29,30]. Thus, the population of PGC controls is expected to differ from the population of people without SCZ/BIP/MDD who present to clinical settings, which may inflate the reported accuracy relative to the accuracy that could be attained in settings involving clinical controls. However, results for case-only analyses excluding controls (i.e., analyzing test sample size ratios of 33.3% for SCZ/BIP/MDD) attained similar predictive performance (Supplementary Figure 12), suggesting that the impact of control screening is likely to be limited. Fifth, DDx-PRS has been developed for within-ancestry prediction, and extending DDx-PRS to cross-ancestry prediction[31–33] is an important direction for future research to ensure equitable application of genetic prediction. Important progress to improve cross-ancestry prediction accuracy has already been made[34–40]; we emphasize the importance of prioritizing genetic studies of diverse ancestries, and additionally highlight the importance of improving the calibration of cross-ancestry prediction[41]. Sixth, DDx-PRS provides predictions based on polygenic effects of common variants with small effects[7]; extending

DDx-PRS to also include rare variants with large effects (such as 22qdel in SCZ[42,43]) is a direction for future research. Even though rare variants may contribute little to prediction accuracy at the group level, rare variants with large effects have high clinical value for individuals who are carriers. Seventh, in the methods that require tuning data, we did not consider complex multiclass predictors such as XGBoost, which have proven useful in applications involving many predictive features[44]. However, given that we consider only three predictive features (the case-control PRS for SCZ, BIP and MDD) for four diagnostic categories, we anticipate that such predictors would provide little benefit. Eighth, the predicted posterior disorder probabilities computed by DDx-PRS depend strongly on the prior probabilities, which may be misspecified. However, the relative change between the prior and posterior diagnostic category probability is robust to misspecification of the prior (Figures S6 and S7), and thus still informative. Nevertheless, further developing strategies to specify accurate prior disorder probabilities (such as screening for UHR[11]) is an important direction for future research. Ninth, recent studies have addressed the impact of assortative mating, participation bias and socioeconomic status on polygenic risk[45,46]. However, case-control polygenic prediction has consistently been confirmed in fully independent samples across different recruitment settings[6,28–30], and in this study we observed that DDx-PRS predicts consistently across four different independent testing sets (Supplementary Figure 8). Tenth, individual differences and environmental features not considered in this paper could be of high importance for disorder risk. Indeed, our projections at larger training sample sizes shows a clear upper bound to the precision of predictions even at sample-sizes resulting in case-control PRS explaining 100% of the SNP-heritability (Figure 6). Thus, integrating DDx-PRS with non-genetic predictions will be an important step to enhance the potential for clinical utility. Specifically, integrating non-genetic risk factors (i.e. risk factors not derived from genetic data) that are strongly correlated with the case-control PRS (e.g. family history) requires further investigation, and model adjustments may be required[47–49]; on the other hand, non-genetic risk factors that are not correlated with the case-control PRS can be incorporated by adjusting the prior probabilities.

7. Supplementary methods of the 'DDx-PRS method' subsection

(This subsection repeats content from the Methods section, to ensure that it is self-contained.) DDx-PRS consists of 4 steps: (1) estimate prior probabilities of each possible configuration of liabilities; (2) compute case-control PRS; (3) estimate analytical variances and covariances across disorders of liabilities and case-control PRS (overall and for each configuration of liabilities); and (4) estimate posterior probabilities for each test sample. We describe each step in detail below.

Step 1. Estimate prior probabilities of each possible configuration of liabilities.

DDx-PRS relies on the fact that, for *n* disorders, there exist 2^n possible configurations, c, of liabilities (above or below the liability threshold for each disorder[50]), e.g. 8 possible configurations for 3 disorders; the mapping from configurations of liabilities to diagnostic categories is informed by the PGC classifications used in ref. [6,28–30] (Table 1). Given prior clinical probabilities of each diagnostic category, DDx-PRS estimates prior probabilities of each configuration of liabilities by assuming random sampling of liabilities within each diagnostic category. We note that the prior clinical probability will typically be much larger than the population prevalence for help-seeking individuals. For simplicity, we assume a clinical prior probability of 25% for each of SCZ, BIP, MDD and control for the main analyses presented in this paper; however, in application of DDx-PRS, the prior can be specified flexibly by the physician based on e.g. psychiatric examination, questionnaires and/or disorder prevalence in a specific clinical setting.

Specifically, the pairwise genetic correlations SCZ-BIP, SCZ-MDD and BIP-MDD are estimated with cross-trait LDSC[51]. (We note that cross-trait LDSC is robust to sample overlap in controls in training data.[51]) We assume that the correlation of environmental effects equals the genetic correlation[52,53]; thus, the variance-covariance matrices of environmental and genetic effects are specified in the full population, which are added to obtain the variance covariance matrix of liabilities in the full population. Based on the liability thresholds corresponding to the respective disease prevalences ($K_{SCZ} = 0.01$, $K_{BIP} = 0.02$, $K_{MDD} = 0.16$)[6,28,54], normal theory gives the expected prevalence of the eight configurations in Table 1 in the full population (e.g. 0.0024 for configuration c_1, 0.0017 for configuration c_2, 0.0022 for configuration c_3 and 0.0037 for configuration 4, comprising the diagnostic category SCZ in Table 1). These are now proportionally scaled to add up to the specified prior probability (i.e. 25% for the main analyses in this paper) of diagnostic category (e.g. $P(c_1) = 0.0591$ for configuration c_1, $P(c_2) = 0.04215$ for c_2, $P(c_3) = 0.0554$ for c_3 and $P(c_4) = 0.0934$ for c_4, add up to 0.25 for the diagnostic category SCZ in Table 1). To aid interpretation of the results, we specified the prior probabilities of diagnostic categories throughout this paper at 25% for each of SCZ, BIP, MDD and controls. However, we note that the prior probabilities can be specified flexibly, based on e.g. questionnaires, incidence of psychiatric diagnoses in a specific clinical setting, and/or psychiatric examination by a psychiatrist.

Step 1 of DDx-PRS is computationally fast, with a running time of <10 seconds (excluding the running time of cross-trait LDSC).

Step 2. Compute case-control PRS.

DDx-PRS computes case-control PRSs by applying PRS-CS[7] in two datasets: 1000G[55] and the PGC test data (see below). The case-control PRSs are scaled to their respective liability scales[50] by (i) setting N_{eff} as input in PRS-CS, and (ii) multiplying the posterior mean betas outputted by PRS-CS by $\{K_D^2(1-K_D)^2\}/\{z_D^2 * 0.5(1-0.5)\}$ where K_D is the prevalence of the respective disorder (i.e. SCZ, BIP or MDD) and z_D the height of the normal distribution at the liability threshold corresponding to K_D [56] (more details on applying PRS-CS are provided below; all analyses in this paper are restricted to European ancestry).

In brief, the intuition of this transformation is that by specifying $Neff$ as input in PRS-CS, the posterior mean beta's will be on the standardized observed scale with 50-50 case-control ascertainment[57], which are then transformed to the liability scale with Eq 23 of ref.[56]. We note that case-control PRS on the liability scale have the property that the slope of regressing the liability on this PRS has an expected value of 1. A detailed description and evaluation of transforming case-control PRS to the liability scale is provided in ref.[58]. We note that the posterior mean betas could have also been computed with SBayesR[59] (or any other Bayesian polygenic scoring method that has been shown to be well-calibrated[58]).

We use the individual-level 1000G data to compute the variances/covariances of the case-control PRS (see step 3). However, we note these variances/covariances could have also been estimated using summary-level LD data from 1000G in combination with the posterior mean betas, which would make DDx-PRS a method fully based on summary statistics. Nevertheless, in our analyses, we used the individual-level 1000G data, because 1000G is publicly available and there is no significant computational advantage of used summary-level LD data from 1000G instead of individual-level 1000G data. Instead of 1000G, any other population reference sample could have been used as well for this purpose. However, we specifically did not use the test data to estimate the variances/covariances of the case-control PRS, to ensure full independency of the test data from the data used to train DDx-PRS.

The computational cost of step 2 of DDx-PRS depends on the PRS method being applied, e.g. PRS-CS[7].

Step 3. Estimate analytical variances and covariances across disorders of liabilities and case-control PRS (overall and for each configuration of liabilities).

For all configurations c of liabilities (i.e. the eight rows in Table 1), we compute the means and covariances of case-control PRSs and liabilities as follows.

First, we consider the general population (i.e. not conditioned on case/control status) represented in the 1000G data. In the 1000G data, we estimate the variances and covariances of the case-control PRSs. We note that the variances of the case-control PRSs in the full population equal the variances explained by the case-control PRSs on their respective liability scales, i.e. $var(PRS) = r^2_{liab\sim PRS}$, because the case-control PRSs are well-calibrated on their respective liability scales and because the variance of the liabilities is modelled to be 1 (more details are described in ref.[58]). (We note that estimating and accounting for correlations between case-control PRS across disorders accounts for sample overlap in controls in training data.) We define the following variance/covariance matrix Σ:

$$\begin{pmatrix} \sigma^2_{PRS_{SCZ}} & \sigma_{PRS_{SCZ},PRS_{BIP}} & \sigma_{PRS_{SCZ},PRS_{MDD}} & \sigma_{PRS_{SCZ},liab_{SCZ}} & \sigma_{PRS_{SCZ},liab_{BIP}} & \sigma_{PRS_{SCZ},liab_{MDD}} \\ \sigma_{PRS_{SCZ},PRS_{BIP}} & \sigma^2_{PRS_{BIP}} & \sigma_{PRS_{BIP},PRS_{MDD}} & \sigma_{PRS_{BIP},liab_{SCZ}} & \sigma_{PRS_{BIP},liab_{BIP}} & \sigma_{PRS_{BIP},liab_{MDD}} \\ \sigma_{PRS_{SCZ},PRS_{MDD}} & \sigma_{PRS_{BIP},PRS_{MDD}} & \sigma^2_{PRS_{MDD}} & \sigma_{PRS_{MDD},liab_{SCZ}} & \sigma_{PRS_{MDD},liab_{BIP}} & \sigma_{PRS_{MDD},liab_{MDD}} \\ \sigma_{PRS_{SCZ},liab_{SCZ}} & \sigma_{PRS_{BIP},liab_{SCZ}} & \sigma_{PRS_{MDD},liab_{SCZ}} & \sigma^2_{liab_{SCZ}} & \sigma_{liab_{SCZ},liab_{BIP}} & \sigma_{liab_{SCZ},liab_{MDD}} \\ \sigma_{PRS_{SCZ},liab_{BIP}} & \sigma_{PRS_{BIP},liab_{BIP}} & \sigma_{PRS_{MDD},liab_{BIP}} & \sigma_{liab_{SCZ},liab_{BIP}} & \sigma^2_{liab_{BIP}} & \sigma_{liab_{BIP},liab_{MDD}} \\ \sigma_{PRS_{SCZ},liab_{MDD}} & \sigma_{PRS_{BIP},liab_{MDD}} & \sigma_{PRS_{MDD},liab_{MDD}} & \sigma_{liab_{SCZ},liab_{MDD}} & \sigma_{liab_{BIP},liab_{MDD}} & \sigma^2_{liab_{MDD}} \end{pmatrix}$$

The upper-left 3x3 submatrix follows directly from the described estimates in the 1000G data. The covariances $\sigma_{PRS_A,liab_B}$ are computed by $r^2_A * r_{g_{AB}} * \sqrt{h^2_{l_B}/h^2_{l_A}}$, where $r^2_A = \sigma^2_{PRS_A}$ represents the variance explained in $liab_A$ by case-control PRS_A; $h^2_{l_A}$ (resp. $h^2_{l_B}$) the liability scale SNP-heritability of A (resp. B) estimated with LD score regression (based on the baseline LD v2.0 model[60–62]); and $r_{g_{AB}}$ the genetic correlation between disorder A and B estimated with cross-trait LD score regression[51] (we note that cross-trait LDSC is robust to sample overlap in controls in training data.). The variances $\sigma^2_{liab_A}$ equal 1 per model assumptions of the liability scale, and the covariances $\sigma_{liab_A,liab_B}$ are estimated by assuming the correlation of environmental effects are equal to the genetic correlation (see also step 1). The means equal $(0,0,0,0,0,0)$ in the general population; this fully defines this six-dimensional normal distribution in the general population. In our application, the (cross-trait) LD score regression is based on the training data (i.e. summary statistics) of the PGC data (see below). The values of Σ inferred in this study are reported in Supplementary Table 24.

The variances/covariances and means in all 8 configurations c of liabilities (i.e. the eight rows in Table 1) follow by iteratively applying theory of the truncated normal distribution, as described previously[63,64] where truncation is based on the respective disease prevalences ($K_{SCZ} = 0.01$, $K_{BIP} = 0.02$, $K_{MDD} = 0.16$) and their corresponding liability thresholds ($thresh_{SCZ} = 2.326$, $thresh_{BIP} =$

2.054, $thresh_{MDD} = 0.994$). The variances/covariances and means in all 8 configurations c of liabilities are reported in Supplementary Table 24.

For every configuration c_j, this provides the means and variances/covariances of the distribution of the three case-control PRS for SCZ, BIP and MDD, which are approximately normally distributed (even though the liabilities are no longer normally distributed after truncating, the case-control PRS are still approximately normally distributed due to the limited proportion (<<1) of variance explained in liabilities by the PRS). Thus, for every value of $\overrightarrow{PRS_i} = (PRS_{SCZ,i}, PRS_{BIP,i}, PRS_{MDD,i})$, the density $P(\overrightarrow{PRS_i}|c_j)$ in configuration c_j is defined by the properties of the multivariate normal distribution.

The running time of step 3 of DDx-PRS is <10 seconds.

Step 4. Estimate posterior probabilities for each test sample.

In step 4, DDx-PRS applies Bayes' Theorem to estimate posterior probabilities of each configuration of liabilities for each test sample, conditional on their case-control PRS. Letting c_j denote configuration j and $\overrightarrow{PRS_i}$ denote the case-control PRS for each disorder of individual i, these posterior probabilities can be expressed as

$$P\left(c_j\middle|\overrightarrow{PRS_i}\right) = P(\overrightarrow{PRS_i}|c_j)P(c_j)/P(\overrightarrow{PRS_i}) \qquad \text{Eq. 1}$$

where $P(\overrightarrow{PRS_i}|c_j)$ denotes the probability density of $\overrightarrow{PRS_i}$ given configuration c_j (computed from the output of step 3); $P(c_j)$ denotes the prior probability of configuration c_j (computed in step 1); and $P(\overrightarrow{PRS_i})$ denotes the probability density of $\overrightarrow{PRS_i}$ summed across all eight configurations weighted by the prior probabilities, i.e. $P(\overrightarrow{PRS_i}) = \sum_{j'} P(\overrightarrow{PRS_i}|c_{j'})P(c_{j'})$. The posterior probabilities of each configuration of liabilities are subsequently combined into posterior probabilities of each diagnostic category using the information in Table 1 (e.g. $\sum_{j'=1}^{4} P\left(c_{j'}\middle|\overrightarrow{PRS_i}\right)$ provides the posterior probability of the diagnostic category SCZ).

Step 4 of DDx-PRS is computationally fast, with a running time < 1 minute in analysis of the concatenated PGC data (N=11,460; see below).

8. Supplementary methods of the 'Marginal-PRS method' subsection

(This subsection repeats content from the Methods section, to ensure that it is self-contained.) We compare the results of DDx-PRS to the results of a simpler method, Marginal-PRS (Table 2). Marginal-

PRS works in three steps: (1) estimate marginal disorder probabilities; (2) estimate probability to be a control; (3) rescale probabilities to add up to 1.

Step 1. Estimate marginal disorder probabilities.

Marginal-PRS computes the marginal disorder probabilities, $P(SCZ|PRS_{SCZ,i})$, $P(BIP|PRS_{BIP,i})$ and $P(MDD|PRS_{MDD,i})$ one disorder at the time using the Bayesian polygenic score Probability Conversion (BPC) approach[58]. Briefly, for every disorder A, the BPC approach transforms the case-control PRS to its liability scale and models the mean and variance of the PRS based on 1000G data (analogue to step 2 of DDx-PRS). Subsequently, Bayes' Theorem is applied to estimate the posterior probability of being a case for disorder A as $P(D_A = 1|PRS_A) = P(PRS_A|D_A = 1)P(D_A = 1)/P(PRS_A)$. The prior probability $P(D_A = 1)$ directly follows from the prior probability of the diagnostic categories in Table 1. Marginal-PRS assumes that those with $D_A = 0$ are random controls (which is not correct as these contain an over representation of the two other diagnostic categories).

Step 2. Estimate probability to be a control.

Marginal-PRS computes the probability to be a control as $P(control) = (1 - P(SCZ))(1 - P(BIP))(1 - P(MDD))$.

Step 3. Rescale probabilities to add up to 1.

Marginal-PRS rescales these four probabilities to add up to 1. We note two important limitations of Marginal-PRS compared to DDx-PRS. First, Marginal-PRS does not model the covariance structure between the respective case-control PRS. Second, when computing marginal disorder probabilities, Marginal-PRS does not account for the other disorders (e.g., when computing $P(SCZ)$, Marginal-PRS incorrectly assumes that non-SCZ individuals are all controls).

9. Supplementary methods of the 'Methods that require tuning data: DDx-PRS-tuned & Direct-tuned' subsection

(This subsection repeats content from the Methods section, to ensure that it is self-contained.) DDx-PRS and Marginal-PRS do not require additional tuning data to train parameters connecting case-control PRS to probabilities of each diagnostic category. We also considered two methods that do require tuning data: DDx-PRS-tuned and Direct-tuned (Table 2). The tuning data consists of individuals from the four diagnostic categories (SCZ, BIP, MDD and controls) with the case-control PRS for SCZ,

BIP and MDD. The tuning data is completely independent from both the training data and the test data.

DDx-PRS-tuned

DDx-PRS-tuned consists of the same 4 steps as DDx-PRS: (1) estimate prior probabilities of each possible configuration of liabilities; (2) compute case-control PRS; (3) estimate analytical variances and covariances across disorders of liabilities and case-control PRS (overall and for each configuration of liabilities); and (4) estimate posterior probabilities for each test sample. We describe the difference between DDx-PRS-tuned and DDx-PRS in detail below.

Step 1. Estimate prior probabilities of each possible configuration of liabilities.

Step 1 of DDx-PRS-tuned is the same as step 1 of DDx-PRS (see above).

Step 2. Compute case-control PRS.

DDx-PRS-tuned computes case-control PRSs by applying PRS-CS[7] in two datasets: the PGC tuning data and the PGC test data (see below). The tuning data is used to estimate the variance explained by PRS_A (r_A^2) based on case vs. control analyses for disorder A as is commonly done, i.e. by regressing case/control status on the PRS with subsequently transforming the variance explained to the liability scale[65] (note that DDx-PRS approximates r_A^2 based on 1000G data; see steps 2 and 3 of DDx-PRS). Subsequently, DDx-PRS-tuned uses the tuning data to scale the PRS_A such that $var(PRS_A) = r_A^2$ based on data of controls (as an approximation of data of the general population; this replaces step 2 in DDx-PRS). The scaling parameters of PRS_A are based on the tuning data and then also applied on every test sample.

Step 3. Estimate analytical variances and covariances across disorders of liabilities and case-control PRS (overall and for each configuration of liabilities).

Step 3 of DDx-PRS-tuned is almost the same as step 3 of DDx-PRS (see above). The only difference is that in DDx-PRS-tuned, the variance/covariance of the case-control PRS (i.e. the upper-left quadrant of Σ above) is based on the case-control PRS in the PGC tuning data (see step 2 of DDx-PRS-tuned).

Step 4. Estimate posterior probabilities for each test sample.

Step 4 of DDx-PRS-tuned is the same as step 4 of DDx-PRS (see above).

Other than these two adjustments in steps 2 and 3 of DDx-PRS, DDx-PRS-tuned follows the rest of step 3 and the full steps 1 and 4 of DDx-PRS precisely (specifically, also still modelling the eight configurations c_j). Thus, compared to DDx-PRS, DDx-PRS-tuned takes away the uncertainty that comes from the assumptions made to assess r_A^2 based on 1000G data[58].

Direct -tuned

Direct-tuned uses a simpler and more direct approach than DDx-PRS-tuned. Direct-tuned consists of 4 steps: (1) specify prior probabilities of each diagnostic category; (2) compute case-control PRS; (3) estimate variances and covariances across case-control PRS (for the diagnostic categories); and (4) estimate posterior probabilities for each test sample. We describe each step in detail below.

Step 1. Specify prior probabilities of each diagnostic category.

The prior probabilities of each diagnostic category need to be specified as input for Direct-tuned. (Contrary to DDx-PRS, Direct-tuned does not estimate the prior probabilities of each possible configuration of liabilities; Table 1)

Step 2. Compute case-control PRS.

DDx-PRS-tuned computes case-control PRSs by applying PRS-CS[7] in two datasets: the PGC tuning data and the PGC test data (see below).

Step 3. Estimate variances and covariances case-control PRS (for the diagnostic categories).

For each diagnostic category dc_j (i.e., SCZ/BIP/MDD/control), Direct-tuned estimated the variance/covariances of the case-control PRS in the PGC tuning data (see below). Thus, for every value of $\overrightarrow{PRS}_i = (PRS_{SCZ,i}, PRS_{BIP,i}, PRS_{MDD,i})$, the density $P(\overrightarrow{PRS}_i|dc_j)$ in diagnostic category do_j is estimated directly from the tuning data.

Step 4. Estimate posterior probabilities for each test sample.

For each test sample, the posterior probability of diagnostic category do_j follows from Bayes' Theorem

$$P\left(dc_j\middle|\overrightarrow{PRS}_i\right) = P(\overrightarrow{PRS}_i|dc_j)P(dc_j)/P(\overrightarrow{PRS}_i) \qquad \text{Eq. 2}$$

where $P(\overrightarrow{PRS}_i|dc_j)$ denotes the probability density of $\overrightarrow{PRS}_i$ given diagnostic category do_j (computed directly from the tuning data); $P(dc_j)$ denotes the prior probability of diagnostic category do_j; and

$P(\overrightarrow{PRS_i})$ denotes the probability density of $\overrightarrow{PRS_i}$ summed across all four diagnostic categories weighted by the prior probabilities, i.e. $P(\overrightarrow{PRS_i}) = \sum_{j'} P(\overrightarrow{PRS_i}|dc_{j'})P(dc_{j'})$.

10. Supplementary methods of the 'Main simulations with individual-level SNP data' subsection

(This subsection repeats content from the Methods section, to ensure that it is self-contained.) We simulated data based on the genetic architectures of SCZ[6], BIP[28] and MDD[29,30], with liability-scale SNP-heritabilities[56] h_l^2 of 0.24, 0.19 and 0.09, population prevalences of 1%, 2% and 16%, and liability-scale case-control PRS variance explained[65] r^2 of 0.10, 0.09 and 0.04, respectively, and genetic correlations of 0.70 for SCZ-BIP, 0.35 for SCZ-MDD and 0.45 for BIP-MDD (in line with our empirical findings; see below).

We simulated individual-level data for $M = 1{,}000$ SNPs, of which 50% were causal for all three disorders (i.e. number of causal SNPs $M_c = 500$) and 50% were non-causal for all three disorders. In line with previous work[64,66], liability-scale SNP effect sizes ($\beta_{i,lSCZ}$, $\beta_{i,lBIP}$ and $\beta_{i,lMDD}$) were drawn from a three dimensional normal distribution with variances h_{lSCZ}^2/M_c, h_{lBIP}^2/M_c and h_{lMDD}^2/M_c; and covariances $\frac{1}{M_c} r_{g|SCZ-BIP}\sqrt{(h_{lSCZ}^2\, h_{lBIP}^2)}$, $\frac{1}{M_c} r_{g|SCZ-MDD}\sqrt{(h_{lSCZ}^2\, h_{lMDD}^2)}$, and $\frac{1}{M_c} r_{g|BIP-MDD}\sqrt{(h_{lBIP}^2\, h_{lMDD}^2)}$. Effective allele frequencies (EAF) of $M = 1{,}000$ SNPs were drawn from a uniform distribution [0.01,0.5]. Individuals were simulated one-by-one by

1. Randomly assigning $M = 1{,}000$ genotypes G_i (i.e. 0, 1 or 2 effective alleles) with the probabilities given by the $EAFs$ while assuming Hardy-Weinberg equilibrium
2. Defining genetic liabilities as $g_{lD} = \sum \beta_{i,lD}(G_i - 2EAF_i)/\sqrt{2EAF_i(1 - EAF_i)}$, for D in SCZ, BIP and MDD
3. Defining liabilities as $l_D = g_{lD} + e_{lD}$, for D in SCZ, BIP and MDD. The environmental effects e_{lD} are drawn from a three-dimensional normal distribution of e_{lSCZ}, e_{lBIP} and e_{lMDD}, with variances $1 - h_{lSCZ}^2$, $1 - h_{lBIP}^2$ and $1 - h_{lMDD}^2$; and covariances $r_{g|SCZ-BIP}\sqrt{\left((1 - h_{lSCZ}^2)\,(1 - h_{lBIP}^2)\right)}$, $r_{g|SCZ-MDD}\sqrt{\left((1 - h_{lSCZ}^2)\,(1 - h_{lMDD}^2)\right)}$, and $r_{g|BIP-MDD}\sqrt{\left((1 - h_{lBIP}^2)\,(1 - h_{lMDD}^2)\right)}$ (i.e. assuming the correlation of environmental effects is equal to the correlation of genetic effects).
4. Defining disorder status as $D = 1$ when $l_D > thresh_D$ with $thresh_D$ (resp. T_B) corresponding to a population prevalence of K_D, for D in SCZ, BIP and MDD

5. Subsequently, every individual was assigning to one of the diagnostic categories (SCZ, BIP, MDD or control) based on the link between the configurations of liabilities and the PGC classification in Table 1.

Individuals were simulated until the required number of nonoverlapping cases and controls were obtained: in training samples whose sample size was selected to attain the specified case-control PRS r^2 (690/690 cases/controls for SCZ, 1,411/1,411 for BIP, and 4,542/4,542 for MDD)[67] and 1,000 test samples for each diagnostic category (SCZ, BIP, MDD, controls). We also simulated 500 random individuals (i.e. not conditioned on case/control status) to serve as analogue of the population reference sample in applying DDx-PRS (see step 2 and 3 above).

We introduced sample overlap of controls in SCZ, BIP and MDD training data to mimic empirical data, which includes substantial overlap of controls[57]. Specifically, the correlation of error-terms was set at 0.2 for SCZ-BIP, 0.1 for SCZ-MDD and 0.1 for BIP-MDD roughly following the empirical findings of the intercept of cross-trait LDSC[51,57]. From these correlation of error terms, the sample overlap is given by the following formula[57]

$$N_{overlap,A0B0} = correlation\ error\ term * \ 4N_{A0}N_{B0}\sqrt{\left(\frac{1}{4N_{A1}} + \frac{1}{4N_{A0}}\right)\left(\frac{1}{4N_{B1}} + \frac{1}{4N_{B0}}\right)} \text{ Eq. 3}$$

resulting in a sample overlap of controls of 395 for SCZ-BIP, 354 for SCZ-MDD and 506 for BIP-MDD.

After running the three case-control GWAS on the training samples, case-control PRS were computed using Bpred, which analytically computes posterior mean causal effects sizes under a point-normal prior in the special case of no LD[1]. We simulated individual-level SNP data (instead of directly simulating case-control PRS values in test samples) to also assess the performance of the complexities of transforming case-control PRS values to the liability scale (see Methods). We analyzed 50 simulation replicates for each simulation.

We simulated a small number of SNPs (*M*) and training samples (*N*) to limit computational cost, while noting that PRS r^2 primarily depends on *M*/*N*, so that simulations at reduced values of both *M* and *N* are appropriate[1]. Because linkage disequilibrium (LD) does not impact cross-disorder architectures conditional on the above parameter values, we simulated genotypes without LD.

In the results displayed in Figure 1, we note that the concatenated AUC (averaged across the four comparisons) were slightly smaller than the mean AUC (0.645 vs. 0.650 for DDx-PRS and 0.635 vs. 0.639 for Marginal-PRS), which can be explained as follows. The 50 simulation replicates are fully independent, resulting in different causal SNP effect sizes and slightly different heritabilities and genetic correlations across simulation replicates. Thus, all simulation replicates are essentially from

different populations that are very similar but not 100% identical, and concatenating data will introduce some very slight heterogeneity to the data causing the decrease of the AUC (we note estimates of the AUC are in principle sample-size independent). The slight discrepancy across simulation replicates is expected to also impact the ICI, but is obscured for the ICI because the ICI is not sample-size independent.

11. Supplementary methods of the 'Psychiatric Genomics Consortium data' subsection

(This subsection repeats content from the Methods section, to ensure that it is self-contained.) The empirical analyses were conducted using data from the Psychiatric Genomics Consortium (PGC)[68]. The full PGC data that we analyzed consisted of European-ancestry case-control GWAS data for SCZ[6] (76 PGC case-control cohorts; *N*=53,386 cases and 77,258 controls), BIP[28] (36 PGC case-control cohorts; *N*=41,917 cases and 371,549 controls), and MDD[29,30] (34 PGC case-control cohorts; *N*=173,140 cases and 331,433 controls, consisting of the data from ref.[30] excluding 23andMe) (Supplementary Table 1). We note that some of the individuals defined as SCZ cases in ref.[6] were diagnosed with schizoaffective disorder; however, these pertained to only a small subset of some of the cases of 14 of the 90 cohorts, and thus we believe that the overall results are representative of SCZ. We also note that DDx-PRS assumes that controls are screened for the absence of SCZ/BIP/MDD, but the PGC applied different screening strategies per cohort ranging from no screening to screening for MDD, BIP, non-affective psychosis and substance use disorders (i.e., super-normal)[29,30] (Supplementary Table 2), thus the PGC controls are likely different from the population of people presenting to a psychiatric facility who do not have SCZ/BIP/MDD; secondary analyses excluding controls suggest that the impact of the screening of controls is limited (Supplementary Figure 12).

We constructed 4 test cohorts by merging selected PGC case-control cohorts; constructed nonoverlapping training data sets for each disorder-specific PGC case-control cohort that was included in one of the 4 test cohorts; applied QC; and computed case-control PRS using PRS-CS and Plink 1.9. We ran S-LDSC and cross-trait LDSC using the full PGC data. Each of these steps is detailed below.

Construction of 4 test cohorts.

The 4 test cohorts were constructed by merging selected PGC case-control cohorts of SCZ[6], BIP[28] and MDD[29]. Control test samples were selected from the set of MDD controls, which have more stringent criteria for being labeled as a control (Table 1)[29]. SCZ cases were matched to BIP cases by merging PGC case-control cohorts that were from the same country and had at least 200,000 overlapping SNPs on their genotyping chip; BIP cases were matched to MDD cases with the same criteria. Combining the

SCZ-BIP match with the BIP-MDD match also assured an accurate SCZ-MDD match (as confirmed with sensitivity analyses; see below). This resulted in 4 merged test cohorts: GER, UK1, UK2 and USA. From these test cohorts, subjects were randomly selected to obtain sample sizes of exactly 25% of each of SCZ, BIP, MDD and control (the main prior used throughout this study). The resulting test data consisted of 11,460 individuals (2,865 of each of SCZ, BIP, MDD, control): 3,368 in the GER test cohort, 1,136 in UK1, 6,080 in UK2, and 876 in USA. An overview of the PGC case-control cohorts included in the four test cohorts is provided in Supplementary Table 2. The type of screening applied in the test cohort controls is reported in Supplementary Table 2.[29,30] We note that only 2 of the 21 SCZ case-control cohorts included in our test cohorts included some SZA cases (also see above).

When applying methods that require tuning data (DDx-PRS-tuned & Direct-tuned), we primarily focused on cross-cohort tuning, designating one test cohort as the tuning cohort and another test cohort as the test cohort. This resulted in 4x3=12 tuning/test cohort pairs used for analyses. We restricted tuning data to 200 samples from each of the four diagnostic categories (total N_{tuning}=800) and evaluated the performance of each method in test data. In addition to cross-cohort tuning, we also conducted analyses based on within-cohort tuning in the cohorts GER and UK2 (for UK1 and USA, the sample sizes were too small for assessing model performance after excluding 800 samples for tuning).

Construction of training data sets for each disorder-specific PGC case-control test cohort

For each disorder-specific PGC case-control cohort that was included in one of the 4 test cohorts, we constructed corresponding training data sets (used to compute case-control PRS for each disorder) that excluded that PGC case-control cohort. (We note that the 4 test cohorts included only case samples from SCZ and BIP PGC case-control cohorts, and both case and control samples from MDD PGC case-control cohorts; see above). For PGC SCZ case-control cohort X, the SCZ training data consisted of the full SCZ data excluding PGC SCZ case-control cohort X, the BIP training data consisted of the full BIP data, and the MDD training data consisted of the full MDD data (and analogously for PGC BIP case-control cohort X and PGC MDD case-control cohort X). We tested for sample overlap of controls from each PGC MDD case-control cohort X (used as controls in the 4 test cohorts) and PGC SCZ (resp. BIP) case-control cohorts; when such overlap was identified, the overlapping PGC SCZ (resp. BIP) case-control cohort was excluded from the SCZ (resp. BIP) training data for PGC MDD case-control cohort X. With this strategy, we assured independence between training and test data, while also retaining maximum sample size of the training data. We note that the training data was slightly different across PGC case-control cohorts as different PGC training case-control cohorts were held out, but given the held-out samples are <<10% of the full training samples this had a negligible impact

on our results (as confirmed with sensitivity analyses; see below). The precise training data thus used to compute the case-control PRS for every PGC case-control cohort is reported in Supplementary Table 1.

We performed two sets of sensitivity analyses. First, we verified in the 1000G data that the case-control PRS obtained based on these slightly different training data sets had very high correlations (>0.94 for SCZ, >0.93 for BIP, >0.95 for MDD; see Supplementary Table 25). We note that despite these very large correlations among case-control PRS, it is still crucial to exclude the test data from the training data, as the inflation in r^2 can be profound even when the test data is only a small fraction of the training data[69]. We also note that the slight heterogeneity thus introduced in the test data could result in slight conservative bias of our findings. Second, we computed the r^2 contrasting the SCZ cases (resp. BIP cases) to their matched SCZ controls (resp. BIP controls) based on the original PGC case-control cohorts, and verified that this r^2 was highly similar when contrasting the SCZ cases (resp. BIP cases) to the matched MDD controls in the merged test cohorts used in this study (see Supplementary Table 23).

When applying methods that require tuning data (DDx-PRS-tuned and Direct-tuned), instead of just test data and training data we have 4 sample sets: (i) test data; (ii) tuning data; (iii) training data used to compute PRS in tuning samples (training_for_tuning); and (iv) training data used to compute PRS in test data (training_for_test). As described above, of the 4 available test cohorts, we designate one as the test cohort and one as the tuning cohort, for a total of 4x3=12 tuning/test cohort pairs. The training_for_test and training_for_tuning data are specified as described above (see Supplementary Table 1). We note that this has the consequence that there is slight overlap between the training_for_tuning data and the test data. We consider this overlap to be of minor importance, for two reasons. First, there is still no overlap between the training_for_test data and the test data. Second, even if the overlap between the training_for_tuning data and the test data improved prediction in test samples, it would do so only for DDx-PRS-tuned and Direct-tuned (and not DDx-PRS, which does not use tuning data). Given that DDx-PRS achieves comparable (or slightly better) performance as DDx-PRS-tuned and Direct-tuned (see Figure 5), any such impact must be minor, and would lead to conservative reporting of relative results for DDx-PRS (our recommended method) vs. DDx-PRS-tuned and Direct-tuned.

12. Supplementary methods of the 'Simulations of case-control PRS directly for projections of clinical utility at larger training sample sizes' subsection

(This subsection repeats content from the Methods section, to ensure that it is self-contained.) We computed projections to larger training sample sizes by directly simulating case-control PRS (instead

of simulating individual-level SNP data; see Main simulations above) at different values of % of SNP-heritability captured by case-control PRS, ranging from 40% (similar to PGC data; 42-47%) to 100%. Again, data were simulated with liability-scale SNP-heritabilities[56] h_l^2 of 0.24, 0.19 and 0.09, population prevalences of 1%, 2% and 16%, and genetic correlations of 0.70 for SCZ-BIP, 0.35 for SCZ-MDD and 0.45 for BIP-MDD (in line with our empirical findings; see above).

In this simulation, we first simulated the liability scale genetic component as a three-dimensional normal distribution with variances h_{lSCZ}^2, h_{lBIP}^2 and h_{lMDD}^2; covariances $r_{g|SCZ-BIP}\sqrt{(h_{lSCZ}^2\, h_{lBIP}^2)}$, $r_{g|SCZ-MDD}\sqrt{(h_{lSCZ}^2\, h_{lMDD}^2)}$, and $r_{g|BIP-MDD}\sqrt{(h_{lBIP}^2\, h_{lMDD}^2)}$; and means 0,0,0. We assumed to environmental correlation equal to the genetic correlation, and simulated environmental effects as a three-dimensional normal distribution with variances $1 - h_{lSCZ}^2$, $1 - h_{lBIP}^2$ and $1 - h_{lMDD}^2$; covariances $r_{g|SCZ-BIP}\sqrt{\left((1 - h_{lSCZ}^2)\,(1 - h_{lBIP}^2)\right)}$, $r_{g|SCZ-MDD}\sqrt{\left((1 - h_{lSCZ}^2)\,(1 - h_{lMDD}^2)\right)}$, and $r_{g|BIP-MDD}\sqrt{\left((1 - h_{lBIP}^2)\,(1 - h_{lMDD}^2)\right)}$; and means 0,0,0. Liabilities were computed as $l_D = g_{lD} + e_{lD}$, and disease status as $D = 1$ when $l_D > thresh_D$, for D in SCZ, BIP and MDD. Every individual was assigning to one of the diagnostic categories (SCZ, BIP, MDD or control) based on the link between the configurations of liabilities and the diagnostic categories in Table 1.

We then added an error term to the genetic components such that (i) the resulting case-control PRS explained the specified amount of variance r^2 in liability (e.g. for the parametrization of case-control PRS explaining 60% in SCZ, r^2 was specified at $0.6 * 0.24 = 0.144$), and (ii) the correlation of the case-control PRS was equal to the empirically observed correlation of the case-control PRS in 1000G (0.5 for SCZ-BIP, 0.3 for SCZ-MDD and 0.3 for BIP-MDD; for larger of % of SNP-heritability captured by case-control PRS, the correlation between PRS was set to converged to the r_g). Subsequently, the case-control PRS were scaled such that $var(PRS_D) = r_D^2$, for D in SCZ, BIP and MDD, thereby assuring the PRS were well-calibrated on the liability scale.

DDx-PRS was applied to this simulated data. We verified that our empirical findings were similar to simulated results at the exact empirical parameters for both simulating PRS directly (as described here) and simulating individual-level SNP data (described above; see Supplementary Figure 18 and Supplementary Table 22).

Supplementary Table captions

Supplementary Table 1. Overview of PGC training data. The GWAS training data consisted of case-control GWAS summary statistics for SCZ (*N*=53,386 cases and 77,258 controls), BIP (*N*=41,917 cases and 371,549 controls), and MDD (*N*=173,140 cases and 331,433 controls. Panel a: We provide the sample sizes (Neff) of the PGC training data and the number of SNPs included to compute the case-control PRS for main QC (INFO>0.9 and MAF>0.10) and for looser QC (INFO>0.3 and MAF>0.01) of which the results are presented in Supplementary Figure X and Supplementary Table X. Panel b: We show the case-control cohorts excluded from the training data. Specifically, independency between the training data and test data was attained one case-control cohort at the time. Specifically, for SCZ case-control cohort X, the SCZ case-control PRS were based on the SCZ training data excluding cohort X, the BIP case-control PRS were based on the full BIP training data, and the MDD case-control PRS were based on the full MDD training data (and analogue for the BIP case-control cohorts and MDD case-control cohorts). We tested for sample overlap of controls between the MDD controls (used as diagnostic category 'control' in this study) and BIP and SCZ controls; when such overlap was found, the respective SCZ/BIP cohort was excluded from the SCZ/BIP training data to compute the SCZ/BIP PRS in the respective MDD cases and controls.

Supplementary Table 2. Overview of PGC test data. The test data consisted of 11,460 individuals of European ancestry (2,865 of each of SCZ, BIP, MDD, control), subdivided into four cohorts that were matched with respect to country and genotyping platform (GER (N=3,368), UK1 (N=1,136), UK2 (N=6,080), USA (N=876). This Supplementary Table hows the original case-control cohorts from the PGC working groups forming these matches (sample sizes between brackets). Panel a shows the data as used in the main analyses described in this paper; Panel b shows the data based on additional subject QC (see Methods); Panel c shows the full data available after the match with respect to country and genotyping platform. Note that the data in Panel a and Panel b are a random subset of Panel c such data are restricted to 25% of each of SCZ, BIP, MDD and control. Panel d reports the sample-sizes per PGC test cohort for different priors. Note that mdd0 yields the diagnostic category 'controls' in this paper (bip0 and scz0 are not used as controls as these may contain MDD cases).

Supplementary Table 3. Numerical values of Figure 1: Calibration and accuracy of DDx-PRS and Marginal-PRS in simulations. A detailed description is provided in the caption of Figure 1.

Supplementary Table 4. Accuracy of case vs. control (resp. rest) comparisons of DDx-PRS, Marginal-PRS and standard case-control PRS in simulations. We report the accuracy (AUC) of case vs. rest and case vs. control, for schizophrenia (SCZ), bipolar disorder (BIP) and major depressive disorder (MDD) for three methods: DDx-PRS, Marginal-PRS and standard case-control PRS (i.e., as outputted by PRS-CS). These secondary analyses show that the power to distinguish SCZ vs. control (resp. BIP vs. control and MDD vs. control) was comparable for DDx-PRS, Marginal-PRS and the standard case-control PRS. As expected, case vs. rest accuracy was lower than case vs. control accuracy for SCZ (AUC of 0.678±0.001 for SCZ vs. rest, 0.745±0.002 for SCZ vs. control for DDx-PRS) and BIP (0.629±0.002 for BIP vs. rest, 0.701±0.002 for BIP vs. control)—but slightly higher than case vs. control accuracy for MDD (0.618±0.002 for MDD vs. rest, 0.610±0.002 for MDD vs. control), because MDD cases are genetically more similar to controls than to SCZ cases and BIP cases.

Supplementary Table 5. Numerical values of Supplementary Figure 1: Calibration and accuracy of DDx-PRS and Marginal-PRS for comparisons across all 6 pairs of diagnostic categories in simulations. A detailed description is provided in the caption of Supplementary Figure 1.

Supplementary Table 6. Numerical values of Supplementary Figure 2: Calibration slopes of DDx-PRS and Marginal-PRS in simulations. A detailed description is provided in the caption of Supplementary Figure 2.

Supplementary Table 7. Numerical values of Supplementary Figure 3: Calibration and accuracy of DDx-PRS and Marginal-PRS at double training sample size in simulations. A detailed description is provided in the caption of Supplementary Figure 3.

Supplementary Table 8. Numerical values of Supplementary Figure 4: Calibration and accuracy of DDx-PRS and Marginal-PRS with smaller genetic correlation SCZ-BIP in simulations. A detailed description is provided in the caption of Supplementary Figure 4.

Supplementary Table 9. Numerical values of Supplementary Figure 5: Calibration and accuracy of DDx-PRS and Marginal-PRS with no overlap of controls in training data in simulations. A detailed description is provided in the caption of Supplementary Figure 5.

Supplementary Table 10. Numerical values of Figure 3: Calibration and accuracy of DDx-PRS and Marginal-PRS in PGC data. A detailed description is provided in the caption of Figure 3.

Supplementary Table 11. Accuracy of case vs. control (resp. rest) comparisons of DDx-PRS, Marginal-PRS and standard case-control PRS in PGC data. We report the accuracy (AUC) of case vs. rest and case vs. control, for schizophrenia (SCZ), bipolar disorder (BIP) and major depressive disorder (MDD) for three methods: DDx-PRS, Marginal-PRS and standard case-control PRS (i.e., as outputted by PRS-CS). These secondary analyses show that the power to distinguish SCZ vs. control (resp. BIP vs. control and MDD vs. control) was comparable for DDx-PRS, Marginal-PRS and the standard case-control PRS. As expected, case vs. rest accuracy was lower than case vs. control accuracy for SCZ (AUC of 0.655±0.010 for SCZ vs. rest, 0.728±0.016 for SCZ vs. control for DDx-PRS), BIP (0.639±0.013 for BIP vs. rest, 0.720±0.013 for BIP vs. control), and MDD (0.591±0.009 for MDD vs. rest, 0.618±0.017 for MDD vs. control.

Supplementary Table 12. Numerical values of Supplementary Figure 9: Calibration and accuracy of DDx-PRS and Marginal-PRS for comparisons across all 6 pairs of diagnostic categories in PGC data. A detailed description is provided in the caption of Supplementary Figure 9.

Supplementary Table 13. Numerical values of Supplementary Figure 10: Calibration slopes of DDx-PRS and Marginal-PRS in PGC data. A detailed description is provided in the caption of Supplementary Figure 10.

Supplementary Table 14. Numerical values of Supplementary Figure 11: Calibration and accuracy of DDx-PRS and Marginal-PRS for different QC in PGC data. A detailed description is provided in the caption of Supplementary Figure 11.

Supplementary Table 15. Numerical values of Figure 5: Calibration and accuracy of DDx-PRS, DDx-PRS-tuned and Direct-tuned in PGC data. A detailed description is provided in the caption of Figure 5.

Supplementary Table 16. Numerical values of Supplementary Figure 13: Calibration and accuracy of DDx-PRS, DDx-PRS-tuned and Direct-tuned in simulations. A detailed description is provided in the caption of Supplementary Figure 13.

Supplementary Table 17. Numerical values of Supplementary Figure 14: Calibration and accuracy of DDx-PRS-tuned and Direct-tuned at smaller tuning sample sizes in PGC data. A detailed description is provided in the caption of Supplementary Figure 14.

Supplementary Table 18. Numerical values of Supplementary Figure 15: Calibration and accuracy of DDx-PRS-tuned and Direct-tuned using within-cohort tuning data in PGC data. A detailed description is provided in the caption of Supplementary Figure 15.

Supplementary Table 19. Numerical values of Figure 6: Projections of clinical utility at larger training sample sizes. Panel a: A detailed description is provided in the caption of Figure 6. Panel b reports the proportion of diagnostic categories among maximum predicted probability. Panel c reports the ICI, MCC and AUC at larger simulated training sample sizes.

Supplementary Table 20. True vs. predicted diagnosis probabilities in deciles of predicted diagnosis probability of DDx-PRS in PGC data. This Table accompanies main Figure 6 and reports the true vs. the predicted diagnosis probabilities in deciles of predicted diagnosis probability. This secondary Supplementary Table hows that the true and predicted diagnosis probabilities are close. Note that the absolute difference between the true and predicted diagnosis probabilities are very close to the ICIs reported in main Figure 4, as expected as the ICI measure the average absolute difference between the predicted and true probabilities, weighted by the density of predicted probabilities (thus reflects the average error of the predicted probabilities).

Supplementary Table 21. Numerical values of Supplementary Figure 17: True diagnosis probability per decile of predicted diagnosis probability of DDx-PRS, DDx-PRS-tuned and Direct-tuned in PGC data. A detailed description is provided in the caption of Supplementary Figure 17.

Supplementary Table 22. Numerical values of Supplementary Figure 18: True diagnosis probability per decile of predicted diagnosis probability of DDx-PRS in PGC data and in simulations at the exact empirical values. A detailed description is provided in the caption of Supplementary Figure 18.

Supplementary Table 23. R2 of case-control PRS in SCZ (resp. BIP) case vs. matched SCZ (resp. BIP) controls & SCZ (resp. BIP) case vs. MDD controls. We report the r^2 the case-control PRS in contrasting the SCZ cases (resp. BIP cases) to their matched SCZ controls (resp. BIP controls) based on the original PGC studies, and the r^2 of contrasting the SCZ cases (res. BIP cases) to the matched MDD controls (as applied as diagnostic category 'controls' in this study). These r^2 are close thereby confirming that a valid match was made for the merged data used in this paper.

Supplementary Table 24. Variances, covariances and means across disorders of liabilities and case-control PRS (overall and for each configuration of liabilities). The variances/covariances and means are displayed for the case-control PRS and liabilities for SCZ/BIP/MDD, for the full population and for every configuration of liabilities (see main Table 1). The approximations for the full population are based GWAS results from the PGC case-control training data, S-LDSC, cross-trait LDSC, and case-control PRS computed in 1000G; the results for every configuration of liabilities are based on analytical computations (see the description of step 3 of DDx-PRS in the Method section for details).

Supplementary Table 25. Correlation of case-control PRS in 1000G data based on training data with different cohorts held-out. Of the training data, the test data was left out one cohort at a time (Supplementary Table 1), resulting in slightly different training data for the test cohorts. Here we show the correlation of the case-control PRS based on these slightly different training data sets in 1000G. The average correlation of the PRS is 0.96 for SCZ, 0.95 for BIP and 0.96 for MDD; the minimum correlation of the PRS is 0.94 for SCZ, 0.93 for BIP and 0.95 for MDD. The high correlation between these case-control PRS validates the approach to exclude one test cohort at the time to obtain independency between training data and test data. (We note the slight heterogeneity thus introduced in the test data could results in slight conservative bias.)

Supplementary Figures

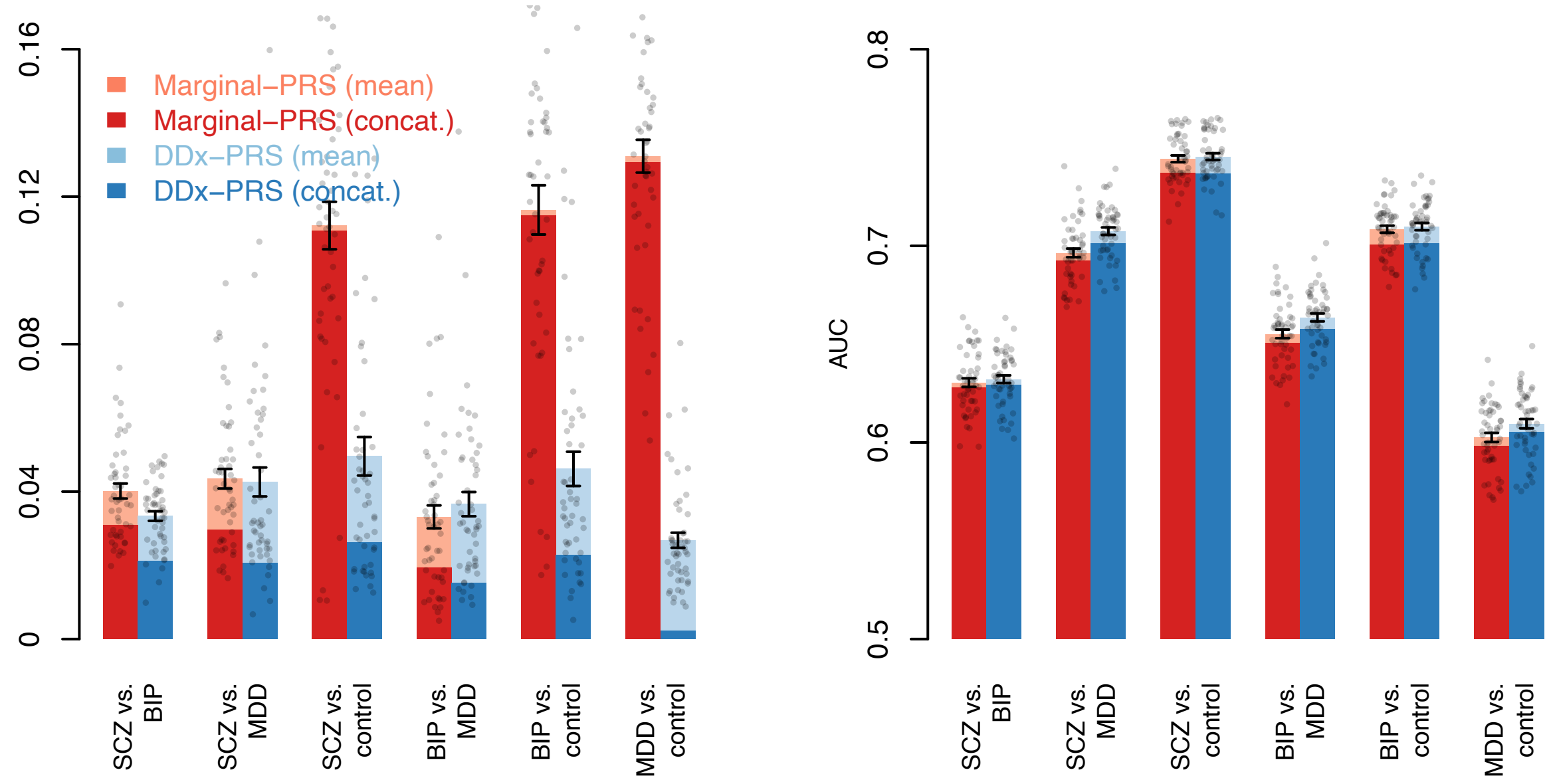

Supplementary Figure 1. Calibration and accuracy of DDx-PRS and Marginal-PRS for comparisons across all 6 pairs of diagnostic categories in simulations. This Figure is analogue to main Figure 1 and reports the calibration (Integrated Calibration Index; ICI) and accuracy (area under the ROC curve; AUC) for the comparisons of schizophrenia (SCZ) vs. bipolar disorder (BIP), SCZ vs. major depressive disorder (MDD), SCZ vs. control, BIP vs. MDD, BIP vs. control and MDD vs. control, for two methods: Marginal-PRS (red) and DDx-PRS (blue). Results are based on 50 simulation replicates; mean values are displayed in light red and light blue. Error bars denote standard errors. Numerical results are reported in Supplementary Table 5. These secondary analyses show that DDx-PRS attained substantially better calibration and slightly higher accuracy than Marginal-PRS for these 6 comparisons; this is in line with the findings of the four comparisons of one diagnostic category vs. rest (main Figure 1).

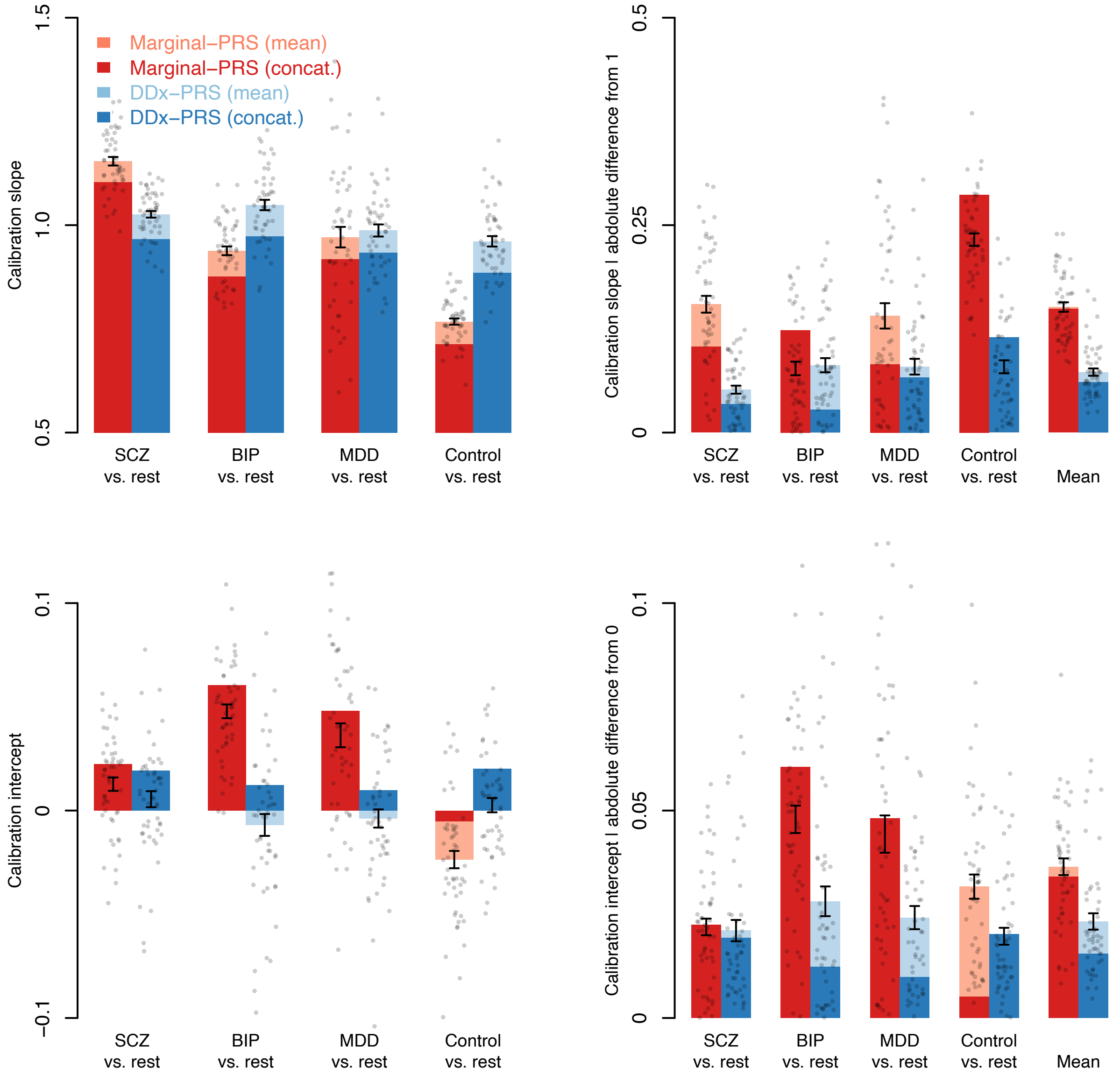

Supplementary Figure 2. Calibration slopes of DDx-PRS and Marginal-PRS in simulations. This Figure accompanies main Figure 1 and reports the calibration slopes and intercepts for the comparisons of schizophrenia (SCZ) vs. rest, bipolar disorder (BIP) vs. rest, major depressive disorder (MDD) vs. rest, and control vs. rest, for two methods: Marginal-PRS (red) and DDx-PRS (blue). Results are based on 50 simulation replicates; mean values are displayed in light red and light blue. Error bars denote standard errors. Perfect calibration would attain a slope of 1 and intercept of 0. Numerical results are reported in Supplementary Table 6. These secondary analyses show that DDx-PRS attained substantially better calibration than Marginal-PRS with a calibration slope closer to 1 and calibration intercept closer to 0; this is in line with the finding from the ICI (main Figure 1).

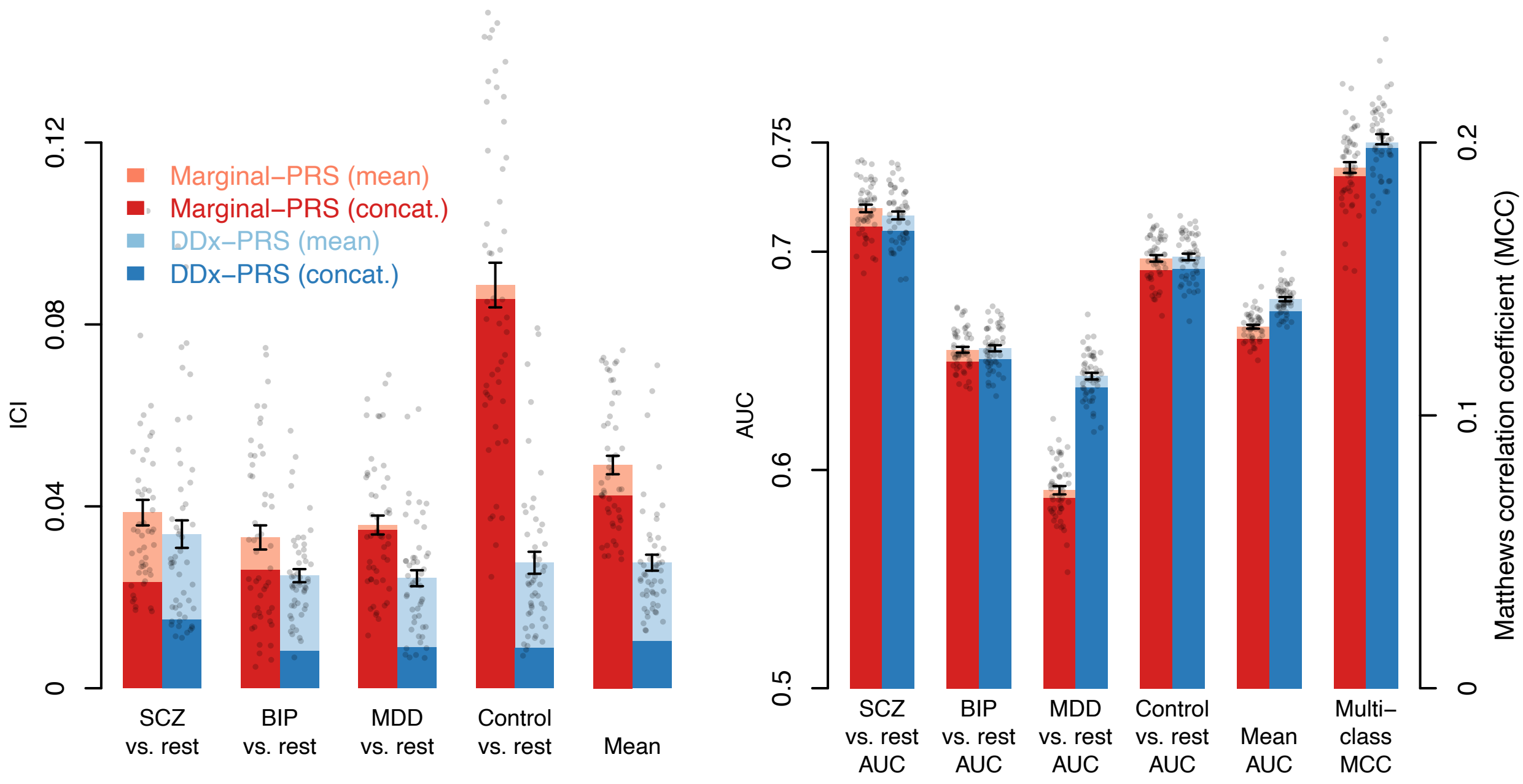

Supplementary Figure 3. Calibration and accuracy of DDx-PRS and Marginal-PRS at double training sample size in simulations. This Figure is analogue to main Figure 1 and reports results at double training sample size in simulations. We report the calibration (Integrated Calibration Index; ICI) and accuracy (area under the ROC curve; AUC) for the comparisons of schizophrenia (SCZ) vs. rest, bipolar disorder (BIP) vs. rest, major depressive disorder (MDD) vs. rest, control vs. rest and the mean across these four comparisons, for two methods: Marginal-PRS (red) and DDx-PRS (blue). We also report the multi-class Matthews Correlation Coefficient (MCC). Results are based on 50 simulation replicates; mean values are displayed in light red and light blue. Error bars denote standard errors. Numerical results are reported in Supplementary Table 7. These secondary analyses show that DDx-PRS attained substantially better calibration than Marginal-PRS (with ICI values similar to main Figure 1a) and slightly higher accuracy than Marginal-PRS (with AUC values larger than main Figure 1b for both methods, as expected).

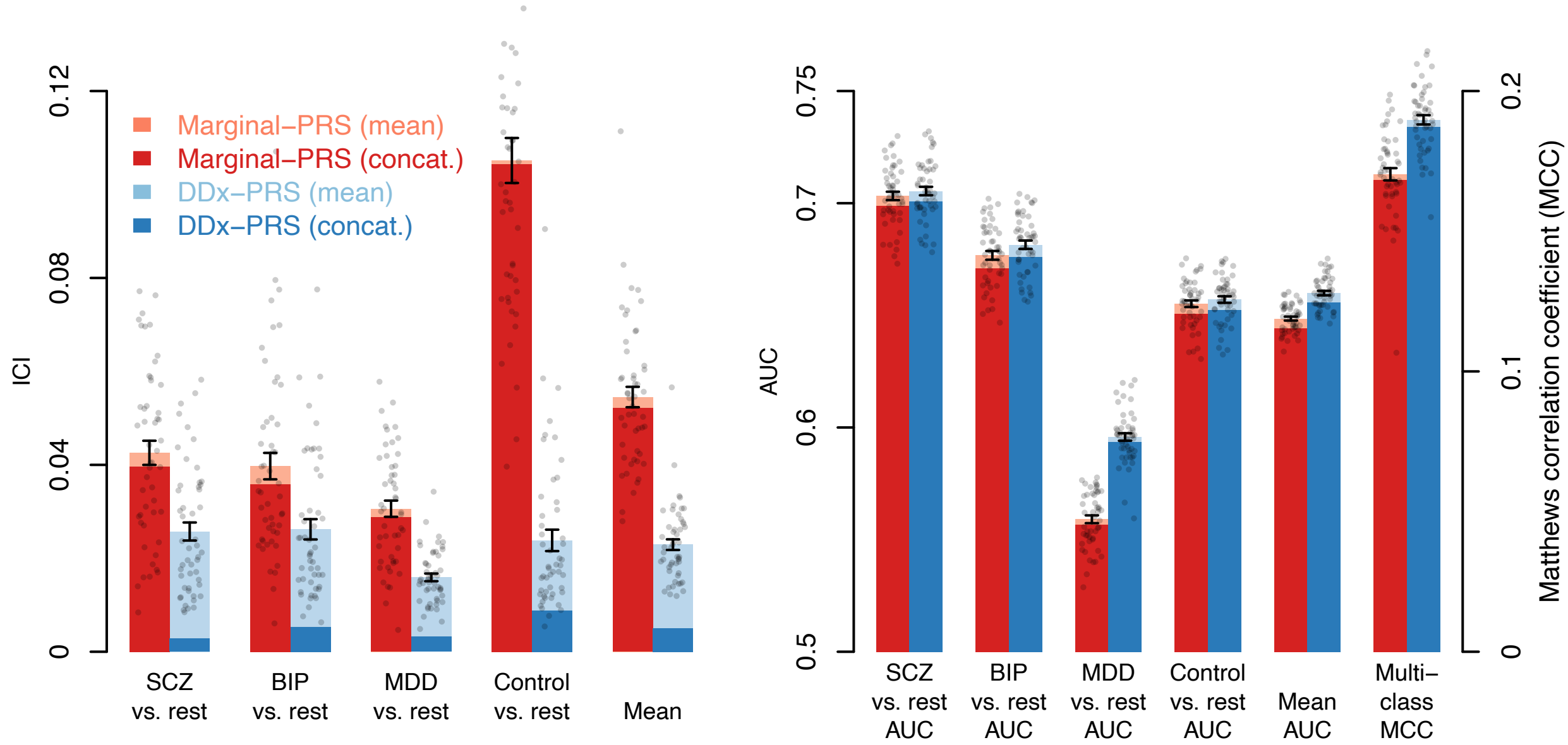

Supplementary Figure 4. Calibration and accuracy of DDx-PRS and Marginal-PRS with smaller genetic correlation SCZ-BIP in simulations. This Figure is analogue to main Figure 1 and reports results at a smaller genetic correlation SCZ-BIP of 0.3 (instead of 0.7 in main Figure 1) in simulations. We report the calibration (Integrated Calibration Index; ICI) and accuracy (area under the ROC curve; AUC) for the comparisons of schizophrenia (SCZ) vs. rest, bipolar disorder (BIP) vs. rest, major depressive disorder (MDD) vs. rest, control vs. rest and the mean across these four comparisons, for two methods: Marginal-PRS (red) and DDx-PRS (blue). We also report the multi-class Matthews Correlation Coefficient (MCC). Results are based on 50 simulation replicates; mean values are displayed in light red and light blue. Error bars denote standard errors. Numerical results are reported in Supplementary Table 8. These secondary analyses show that DDx-PRS attained better calibration and slightly higher accuracy than Marginal-PRS, with both methods attaining higher accuracy than in our main simulations due to better discrimination between SCZ and BIP (main Figure 1).

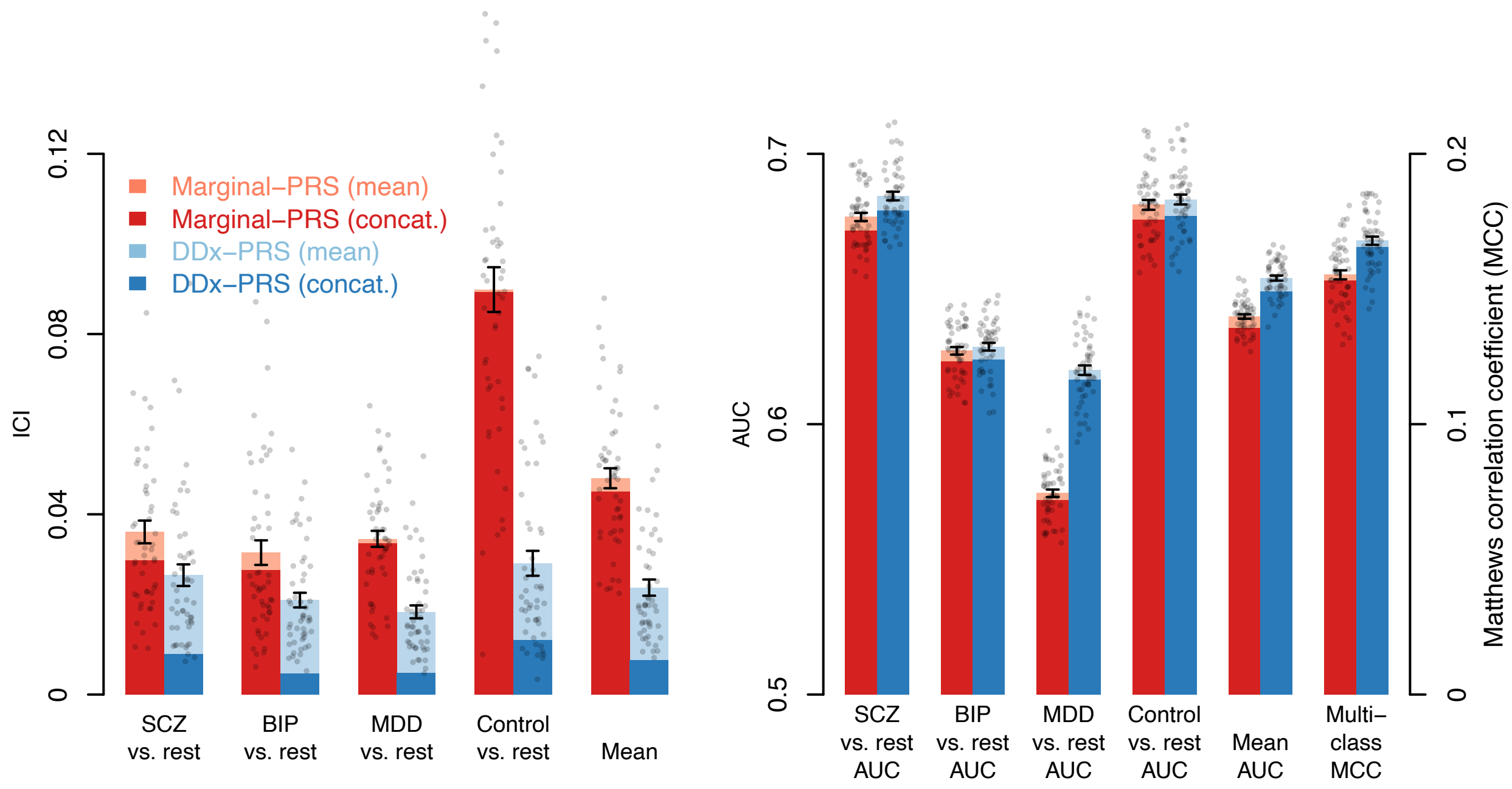

Supplementary Figure 5. Calibration and accuracy of DDx-PRS and Marginal-PRS with no overlap of controls in training data in simulations. This Figure is analogue to main Figure 1 and reports results with no overlap of controls in training data in simulations. We report the calibration (Integrated Calibration Index; ICI) and accuracy (area under the ROC curve; AUC) for the comparisons of schizophrenia (SCZ) vs. rest, bipolar disorder (BIP) vs. rest, major depressive disorder (MDD) vs. rest, control vs. rest and the mean across these four comparisons, for two methods: Marginal-PRS (red) and DDx-PRS (blue). We also report the multi-class Matthews Correlation Coefficient (MCC). Results are based on 50 simulation replicates; mean values are displayed in light red and light blue. Error bars denote standard errors. Numerical results are reported in Supplementary Table 9. These secondary analyses show that whether there is overlap among controls in training data (main Figure 1) or no overlap (this Figure) has almost no impact on the results.

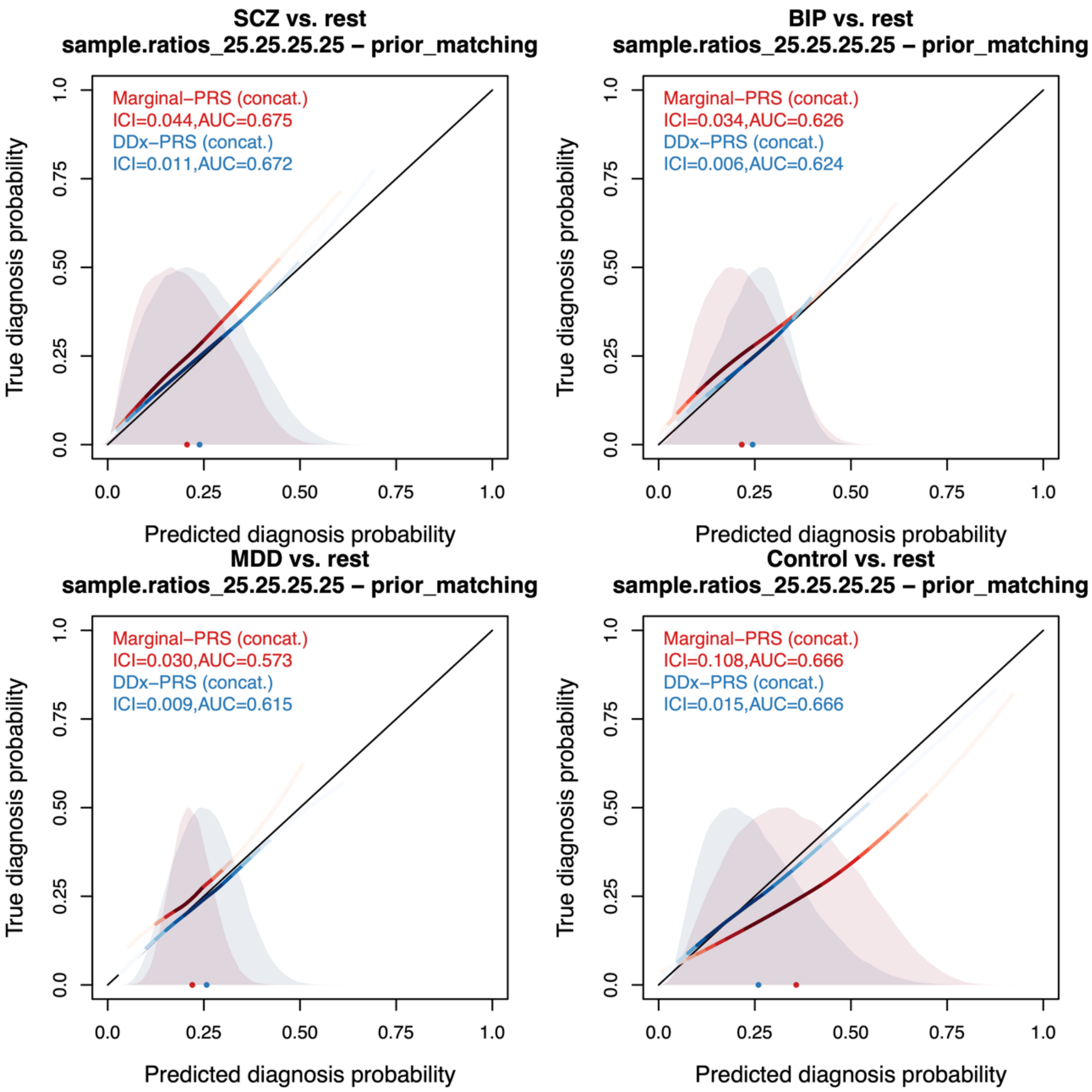

Supplementary Figure 6a. Calibration curves of DDx-PRS and Marginal-PRS for different sample size ratios in simulations. Panel a. We report the true diagnosis probability vs. the predicted diagnosis probability for the comparisons of schizophrenia (SCZ) vs. rest, bipolar disorder (BIP) vs. rest, major depressive disorder (MDD) vs. rest, and control vs. rest, for two methods: Marginal-PRS (red) and DDx-PRS (blue). Panels a-k report results based on correctly specified prior probabilities (i.e., the prior matches the sample size ratios); Panels l-s report results based on misspecified priors. An overview of the results of this Figure is provided in Supplementary Figure 7.

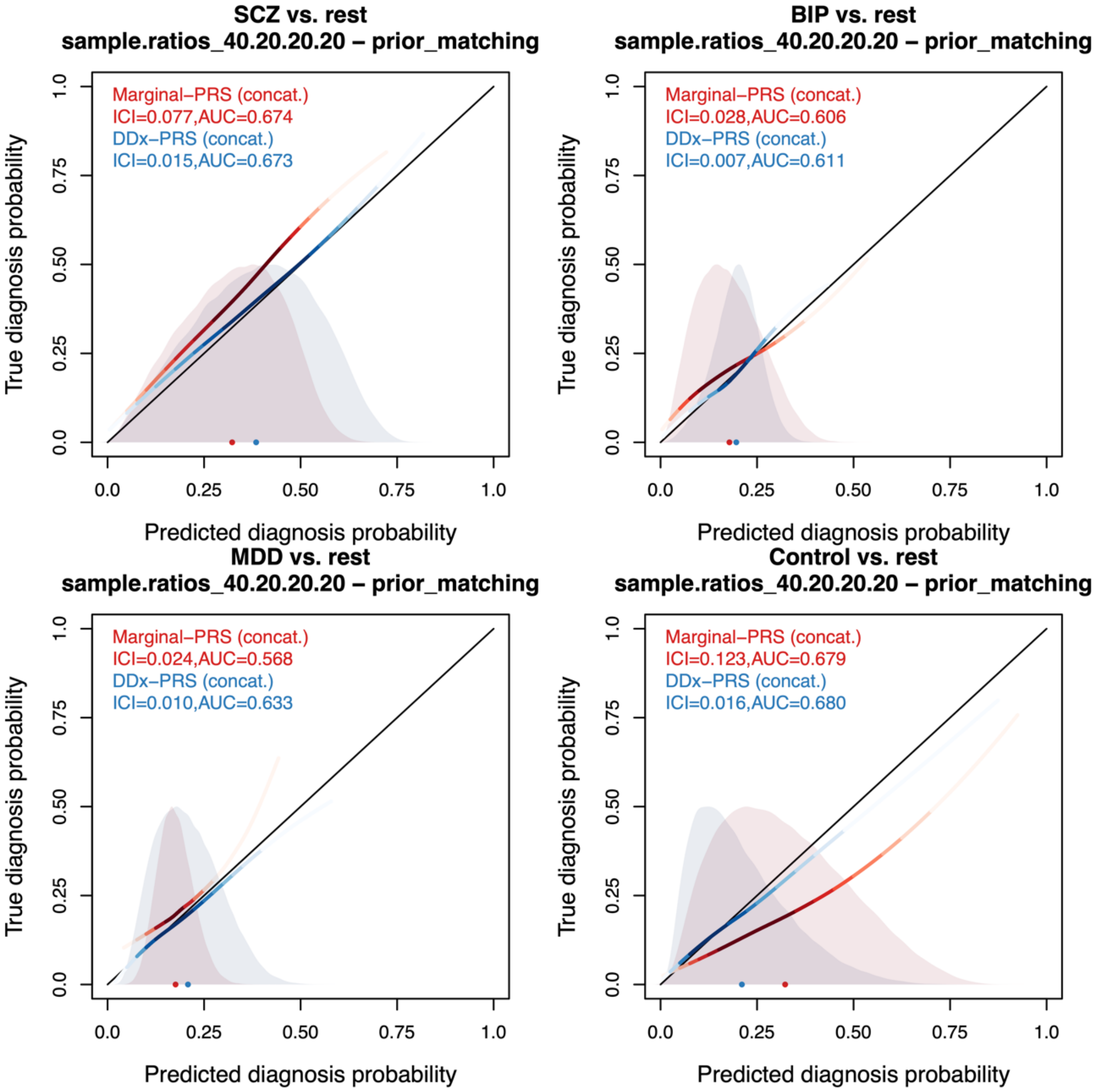

Supplementary Figure 6b. Calibration curves of DDx-PRS and Marginal-PRS for different sample size ratios in simulations. Panel b. We report the true diagnosis probability vs. the predicted diagnosis probability for the comparisons of schizophrenia (SCZ) vs. rest, bipolar disorder (BIP) vs. rest, major depressive disorder (MDD) vs. rest, and control vs. rest, for two methods: Marginal-PRS (red) and DDx-PRS (blue). Panels a-k report results based on correctly specified prior probabilities (i.e., the prior matches the sample size ratios); Panels l-s report results based on misspecified priors. An overview of the results of this Figure is provided in Supplementary Figure 7.

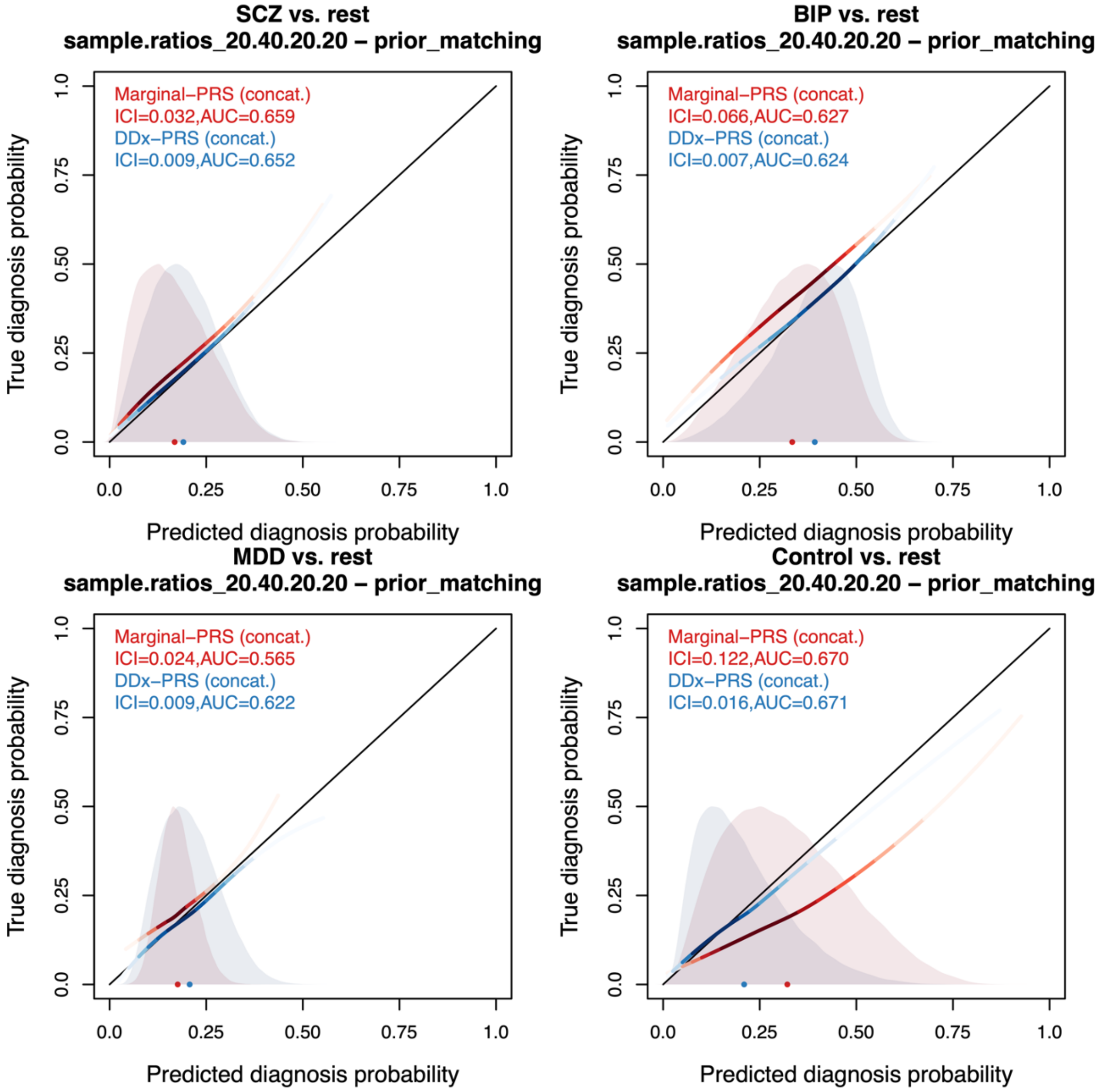

Supplementary Figure 6c. Calibration curves of DDx-PRS and Marginal-PRS for different sample size ratios in simulations. Panel c. We report the true diagnosis probability vs. the predicted diagnosis probability for the comparisons of schizophrenia (SCZ) vs. rest, bipolar disorder (BIP) vs. rest, major depressive disorder (MDD) vs. rest, and control vs. rest, for two methods: Marginal-PRS (red) and DDx-PRS (blue). Panels a-k report results based on correctly specified prior probabilities (i.e., the prior matches the sample size ratios); Panels l-s report results based on misspecified priors. An overview of the results of this Figure is provided in Supplementary Figure 7.

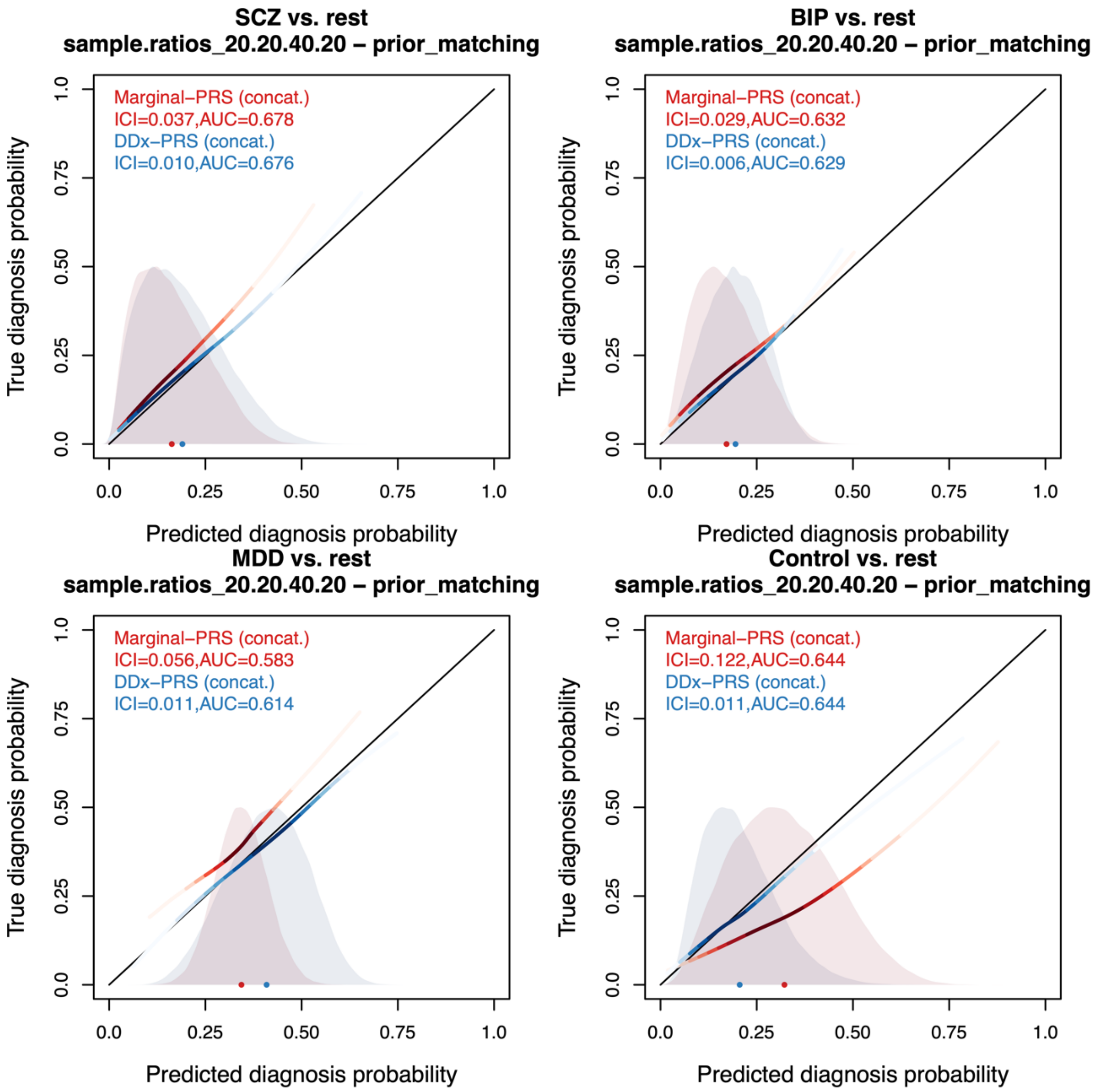

Supplementary Figure 6d. Calibration curves of DDx-PRS and Marginal-PRS for different sample size ratios in simulations. Panel d. We report the true diagnosis probability vs. the predicted diagnosis probability for the comparisons of schizophrenia (SCZ) vs. rest, bipolar disorder (BIP) vs. rest, major depressive disorder (MDD) vs. rest, and control vs. rest, for two methods: Marginal-PRS (red) and DDx-PRS (blue). Panels a-k report results based on correctly specified prior probabilities (i.e., the prior matches the sample size ratios); Panels l-s report results based on misspecified priors. An overview of the results of this Figure is provided in Supplementary Figure 7.

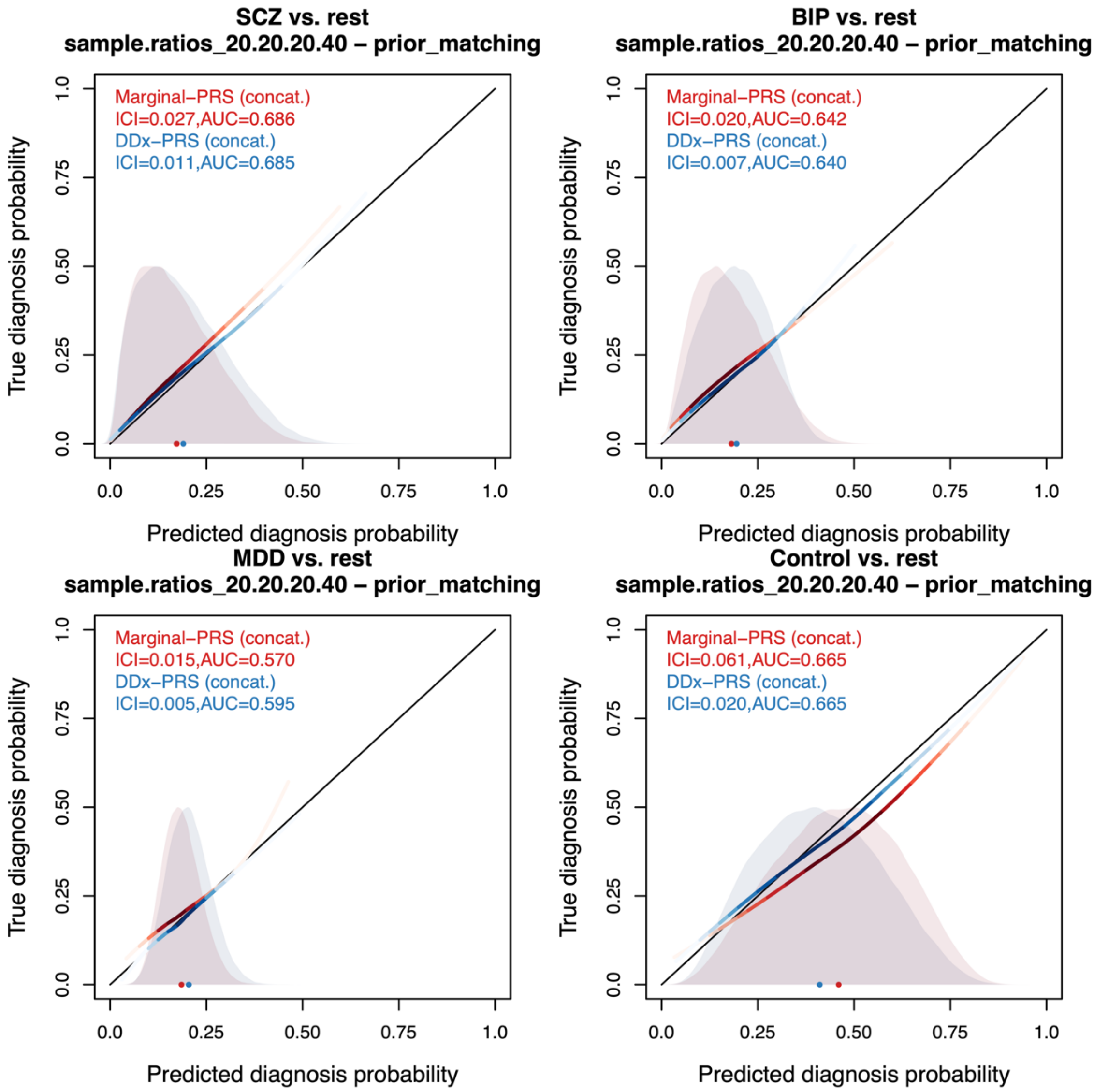

Supplementary Figure 6e. Calibration curves of DDx-PRS and Marginal-PRS for different sample size ratios in simulations. Panel e. We report the true diagnosis probability vs. the predicted diagnosis probability for the comparisons of schizophrenia (SCZ) vs. rest, bipolar disorder (BIP) vs. rest, major depressive disorder (MDD) vs. rest, and control vs. rest, for two methods: Marginal-PRS (red) and DDx-PRS (blue). Panels a-k report results based on correctly specified prior probabilities (i.e., the prior matches the sample size ratios); Panels l-s report results based on misspecified priors. An overview of the results of this Figure is provided in Supplementary Figure 7.

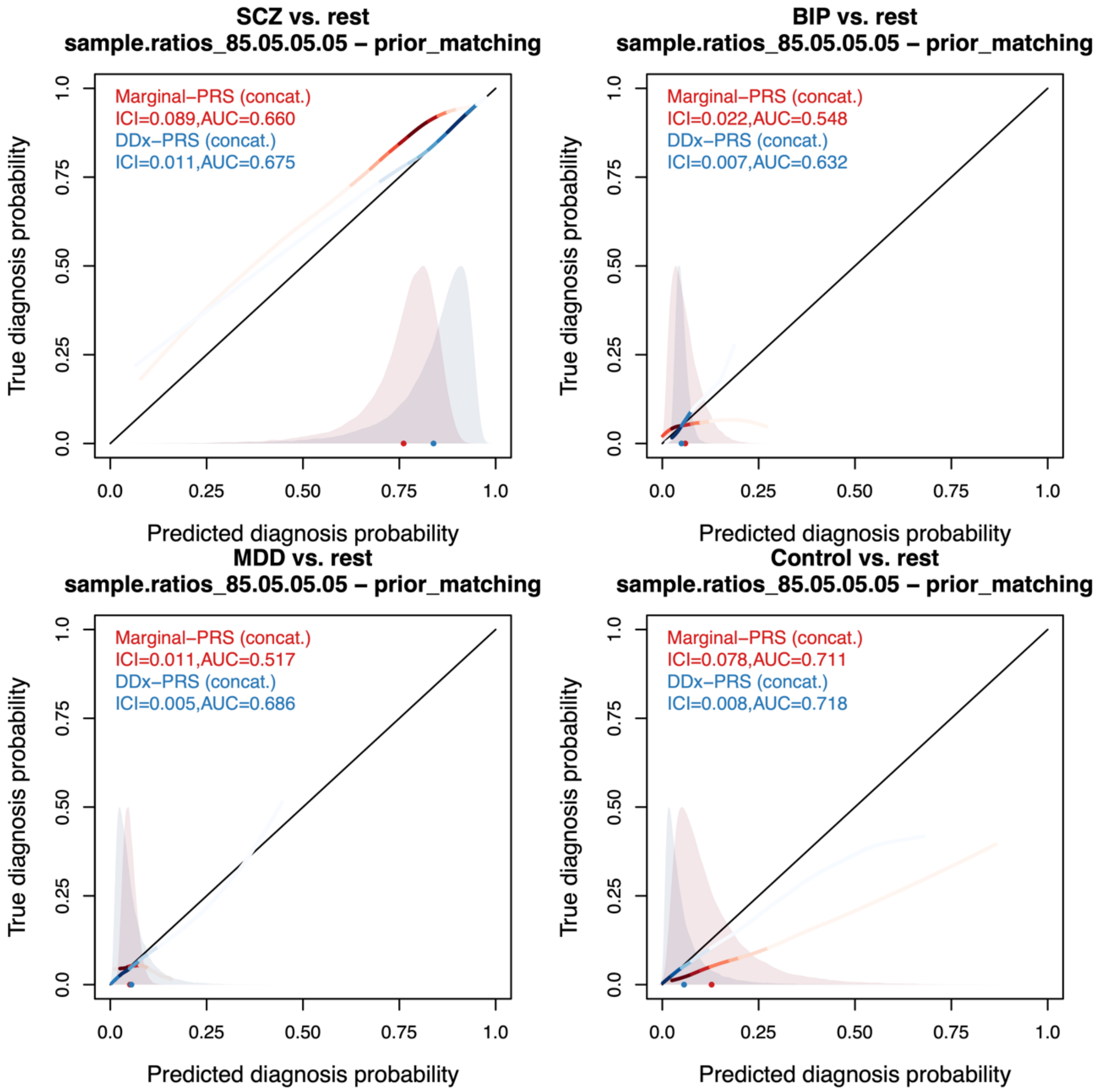

Supplementary Figure 6f. Calibration curves of DDx-PRS and Marginal-PRS for different sample size ratios in simulations. Panel f. We report the true diagnosis probability vs. the predicted diagnosis probability for the comparisons of schizophrenia (SCZ) vs. rest, bipolar disorder (BIP) vs. rest, major depressive disorder (MDD) vs. rest, and control vs. rest, for two methods: Marginal-PRS (red) and DDx-PRS (blue). Panels a-k report results based on correctly specified prior probabilities (i.e., the prior matches the sample size ratios); Panels l-s report results based on misspecified priors. An overview of the results of this Figure is provided in Supplementary Figure 7.

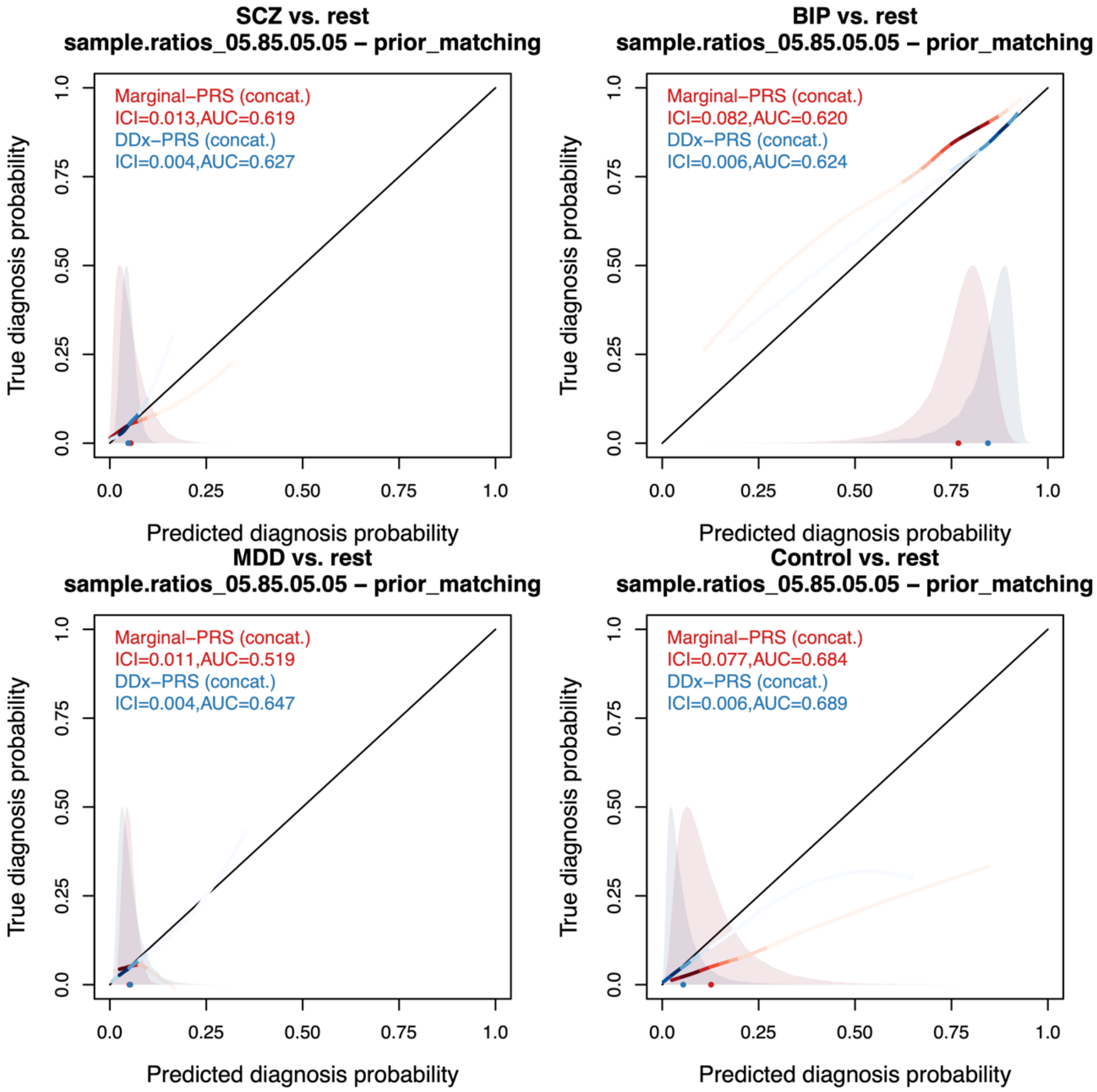

Supplementary Figure 6g. Calibration curves of DDx-PRS and Marginal-PRS for different sample size ratios in simulations. Panel g. We report the true diagnosis probability vs. the predicted diagnosis probability for the comparisons of schizophrenia (SCZ) vs. rest, bipolar disorder (BIP) vs. rest, major depressive disorder (MDD) vs. rest, and control vs. rest, for two methods: Marginal-PRS (red) and DDx-PRS (blue). Panels a-k report results based on correctly specified prior probabilities (i.e., the prior matches the sample size ratios); Panels l-s report results based on misspecified priors. An overview of the results of this Figure is provided in Supplementary Figure 7.

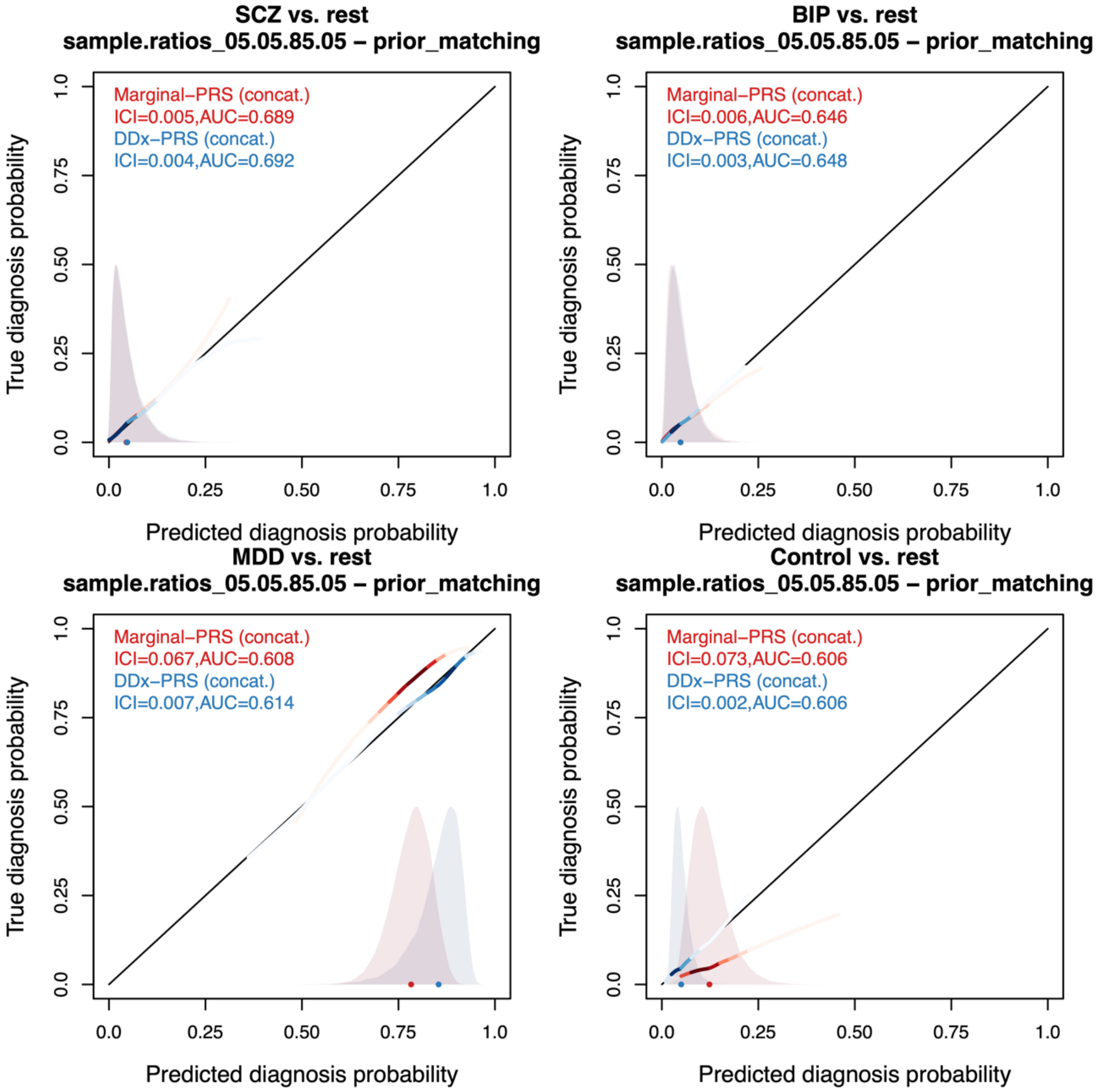

Supplementary Figure 6h. Calibration curves of DDx-PRS and Marginal-PRS for different sample size ratios in simulations. Panel h. We report the true diagnosis probability vs. the predicted diagnosis probability for the comparisons of schizophrenia (SCZ) vs. rest, bipolar disorder (BIP) vs. rest, major depressive disorder (MDD) vs. rest, and control vs. rest, for two methods: Marginal-PRS (red) and DDx-PRS (blue). Panels a-k report results based on correctly specified prior probabilities (i.e., the prior matches the sample size ratios); Panels l-s report results based on misspecified priors. An overview of the results of this Figure is provided in Supplementary Figure 7.

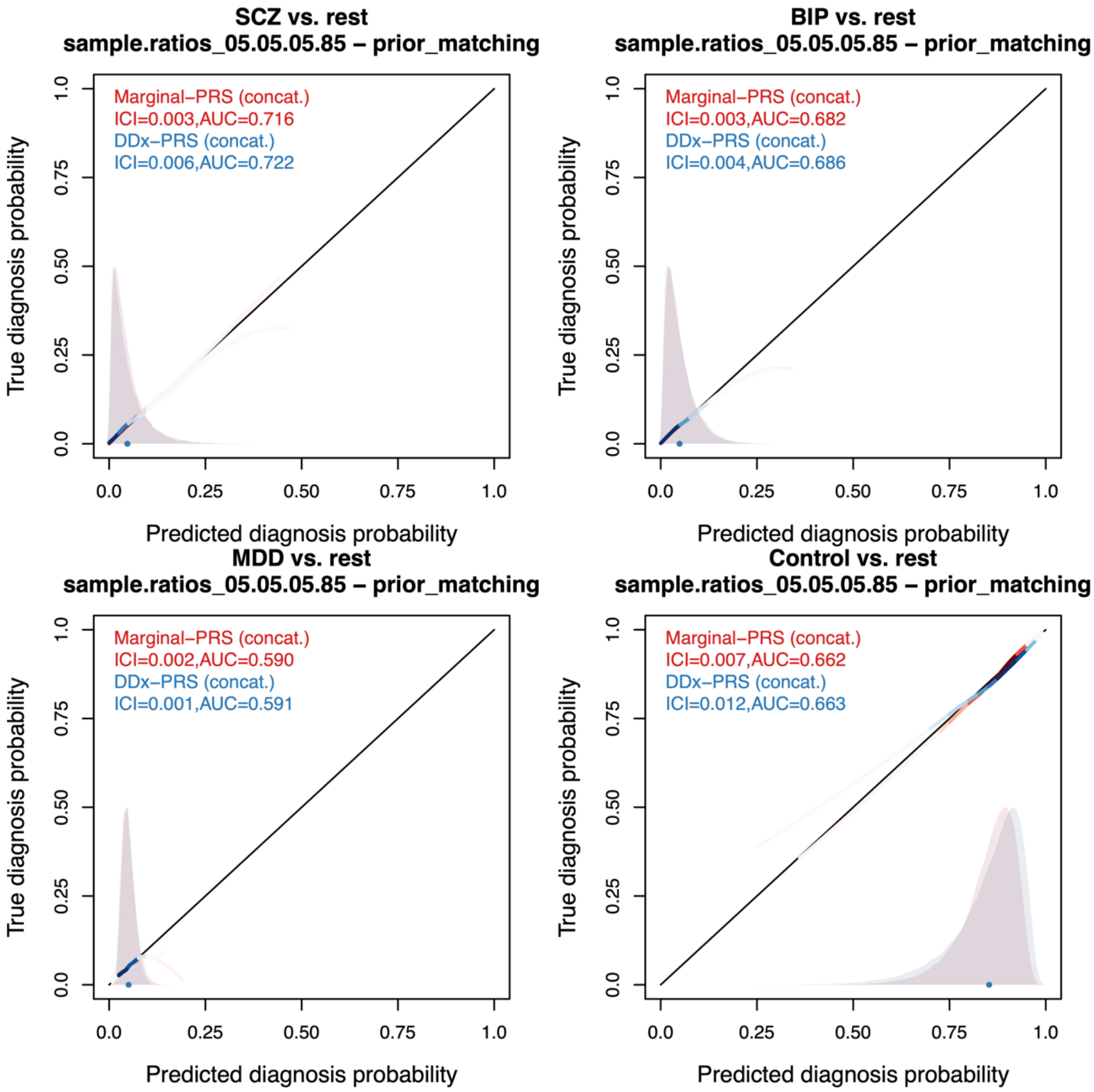

Supplementary Figure 6i. Calibration curves of DDx-PRS and Marginal-PRS for different sample size ratios in simulations. Panel i. We report the true diagnosis probability vs. the predicted diagnosis probability for the comparisons of schizophrenia (SCZ) vs. rest, bipolar disorder (BIP) vs. rest, major depressive disorder (MDD) vs. rest, and control vs. rest, for two methods: Marginal-PRS (red) and DDx-PRS (blue). Panels a-k report results based on correctly specified prior probabilities (i.e., the prior matches the sample size ratios); Panels l-s report results based on misspecified priors. An overview of the results of this Figure is provided in Supplementary Figure 7.

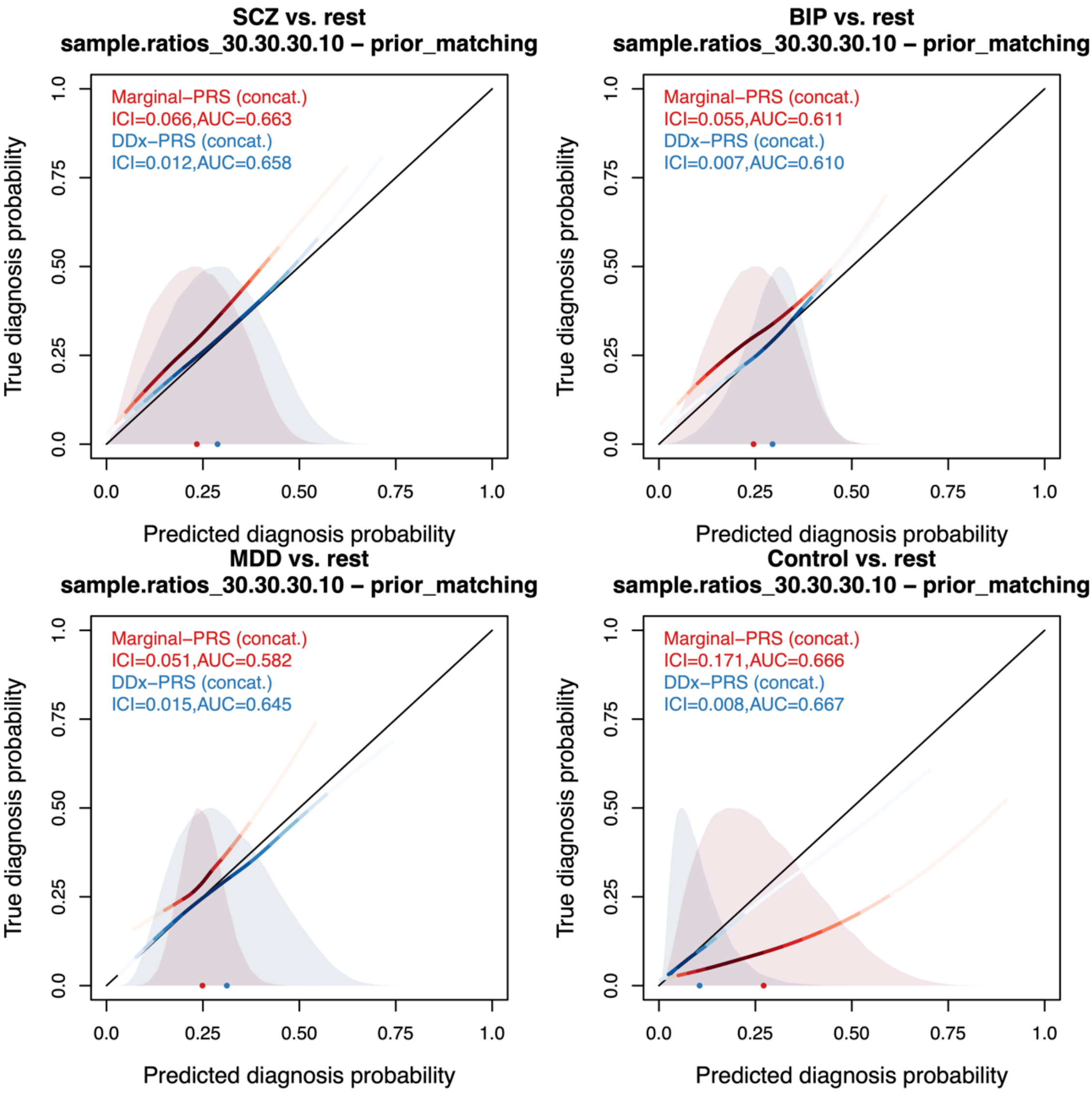

Supplementary Figure 6j. Calibration curves of DDx-PRS and Marginal-PRS for different sample size ratios in simulations. Panel j. We report the true diagnosis probability vs. the predicted diagnosis probability for the comparisons of schizophrenia (SCZ) vs. rest, bipolar disorder (BIP) vs. rest, major depressive disorder (MDD) vs. rest, and control vs. rest, for two methods: Marginal-PRS (red) and DDx-PRS (blue). Panels a-k report results based on correctly specified prior probabilities (i.e., the prior matches the sample size ratios); Panels l-s report results based on misspecified priors. An overview of the results of this Figure is provided in Supplementary Figure 7.

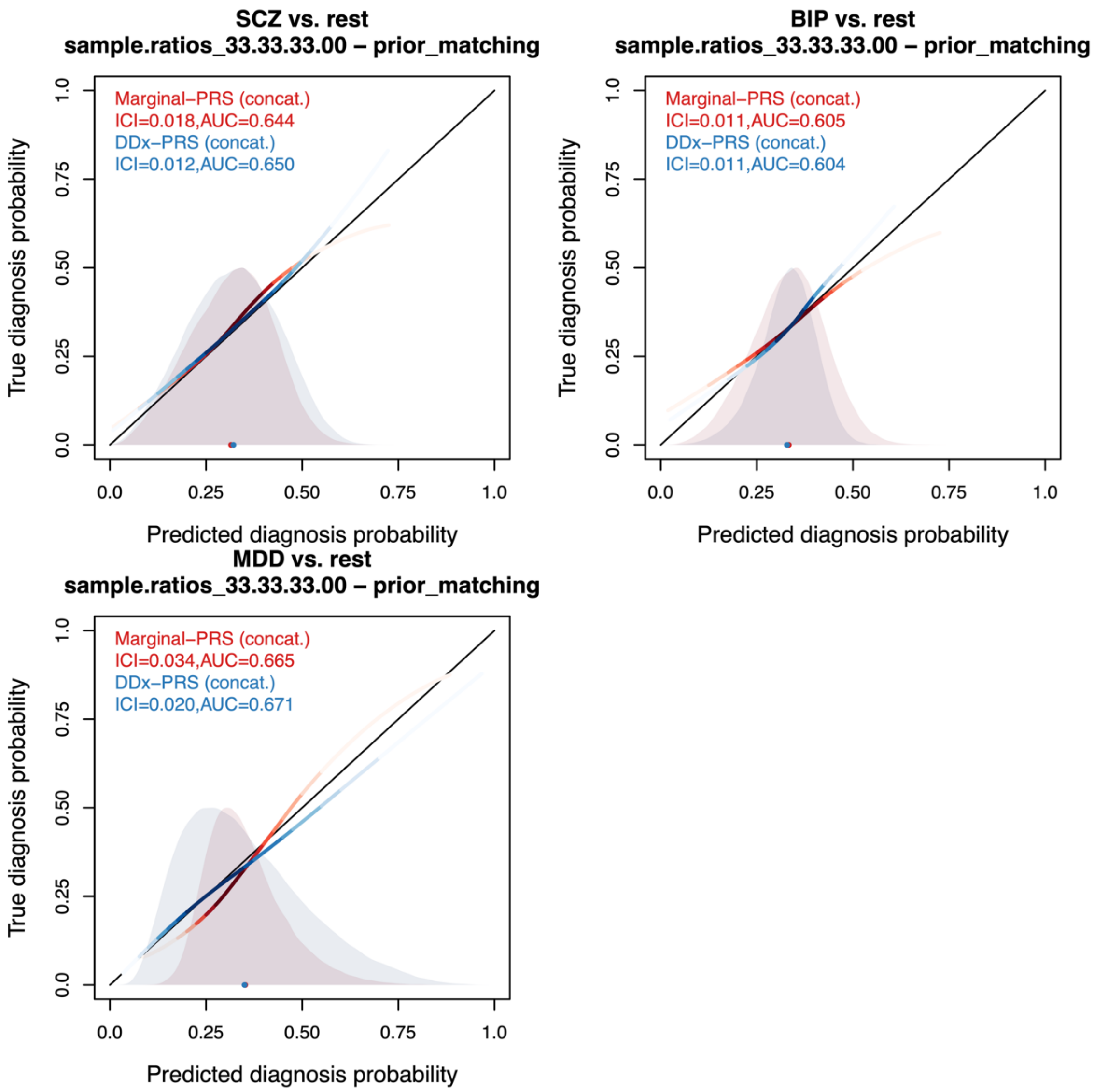

Supplementary Figure 6k. Calibration curves of DDx-PRS and Marginal-PRS for different sample size ratios in simulations. Panel k. We report the true diagnosis probability vs. the predicted diagnosis probability for the comparisons of schizophrenia (SCZ) vs. rest, bipolar disorder (BIP) vs. rest, major depressive disorder (MDD) vs. rest, and control vs. rest, for two methods: Marginal-PRS (red) and DDx-PRS (blue). Panels a-k report results based on correctly specified prior probabilities (i.e., the prior matches the sample size ratios); Panels l-s report results based on misspecified priors. An overview of the results of this Figure is provided in Supplementary Figure 7.

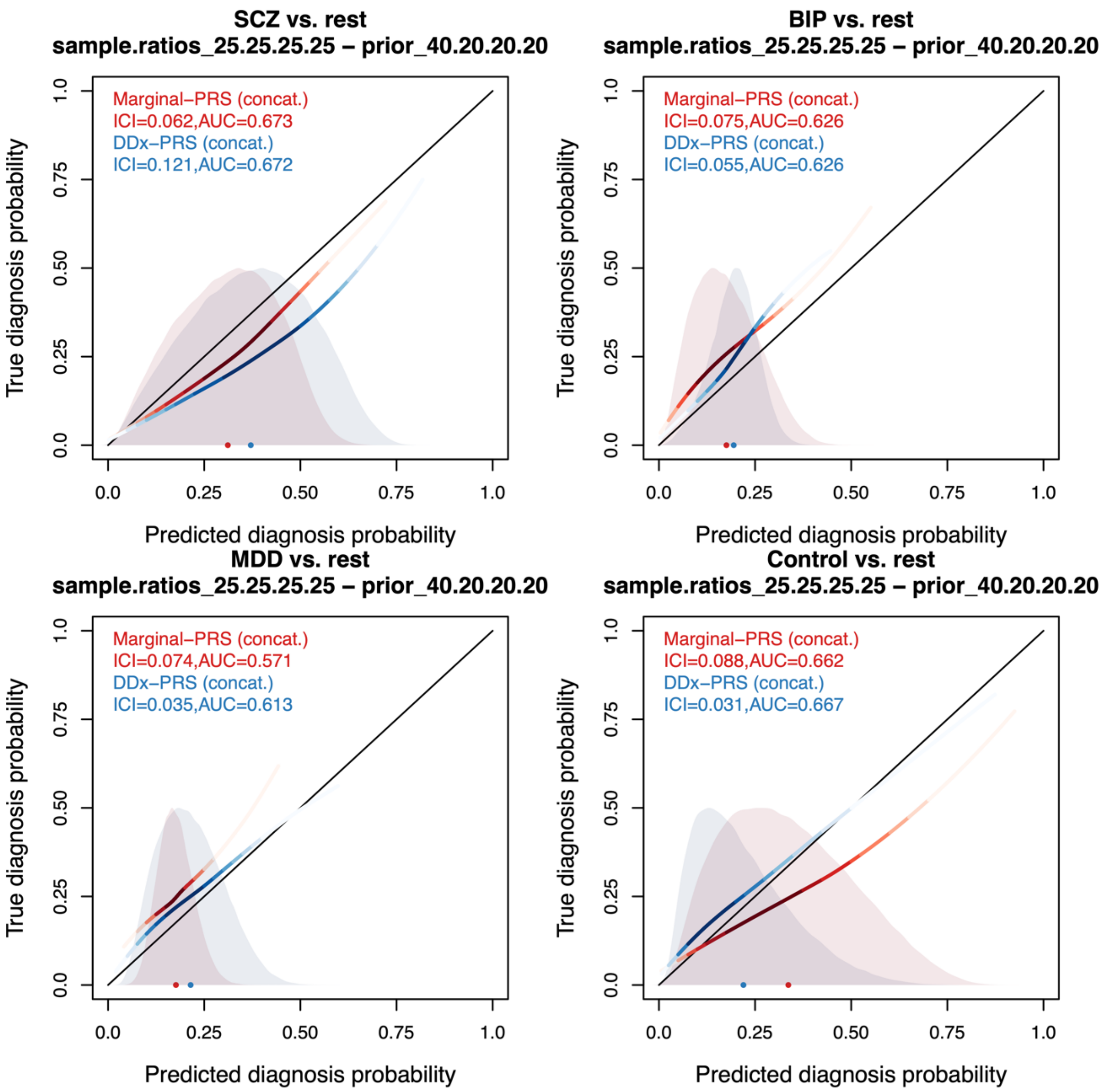

Supplementary Figure 6l. Calibration curves of DDx-PRS and Marginal-PRS for different sample size ratios in simulations. Panel l. We report the true diagnosis probability vs. the predicted diagnosis probability for the comparisons of schizophrenia (SCZ) vs. rest, bipolar disorder (BIP) vs. rest, major depressive disorder (MDD) vs. rest, and control vs. rest, for two methods: Marginal-PRS (red) and DDx-PRS (blue). Panels a-k report results based on correctly specified prior probabilities (i.e., the prior matches the sample size ratios); Panels l-s report results based on misspecified priors. An overview of the results of this Figure is provided in Supplementary Figure 7.

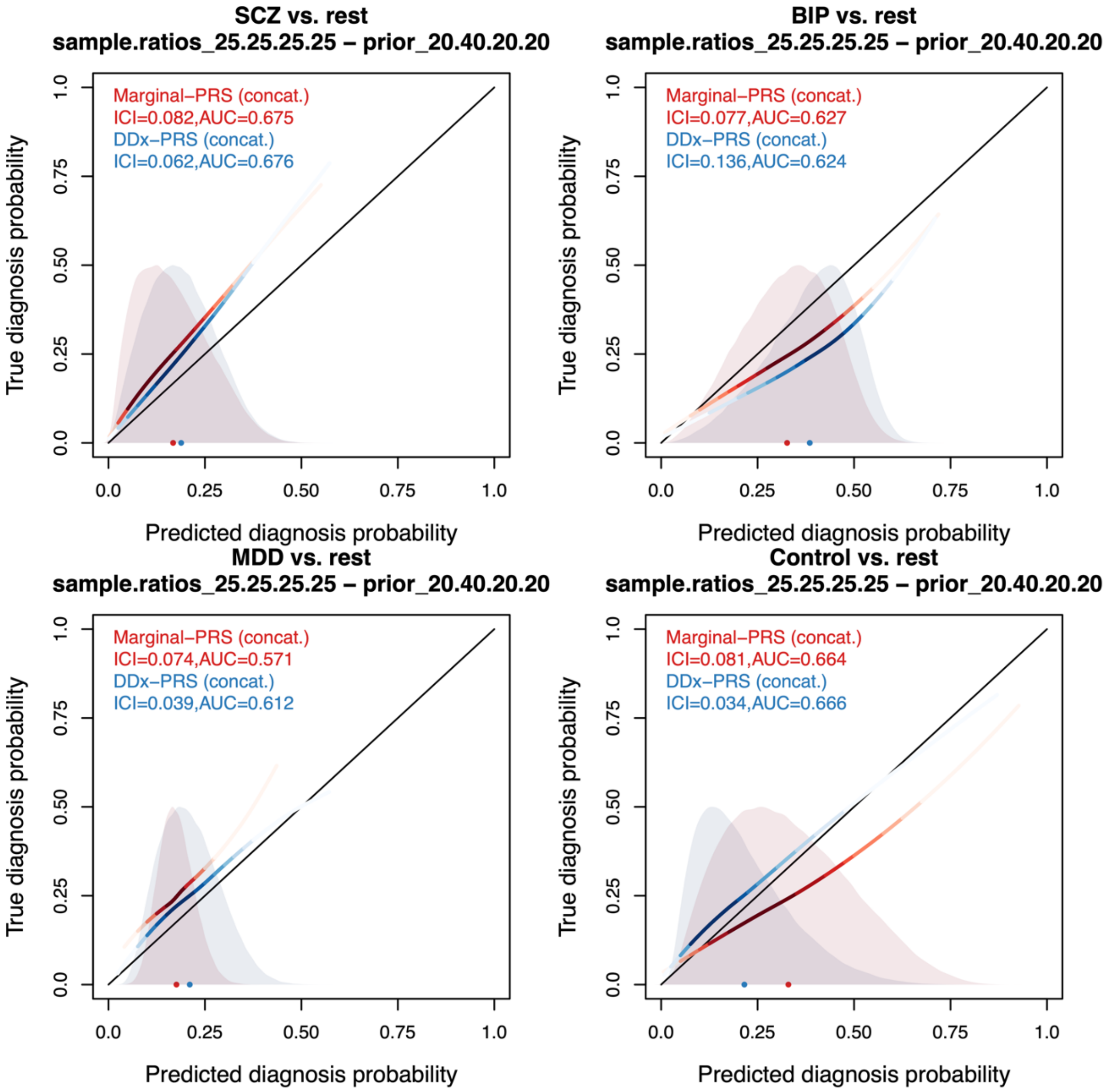

Supplementary Figure 6m. Calibration curves of DDx-PRS and Marginal-PRS for different sample size ratios in simulations. Panel m. We report the true diagnosis probability vs. the predicted diagnosis probability for the comparisons of schizophrenia (SCZ) vs. rest, bipolar disorder (BIP) vs. rest, major depressive disorder (MDD) vs. rest, and control vs. rest, for two methods: Marginal-PRS (red) and DDx-PRS (blue). Panels a-k report results based on correctly specified prior probabilities (i.e., the prior matches the sample size ratios); Panels l-s report results based on misspecified priors. An overview of the results of this Figure is provided in Supplementary Figure 7.

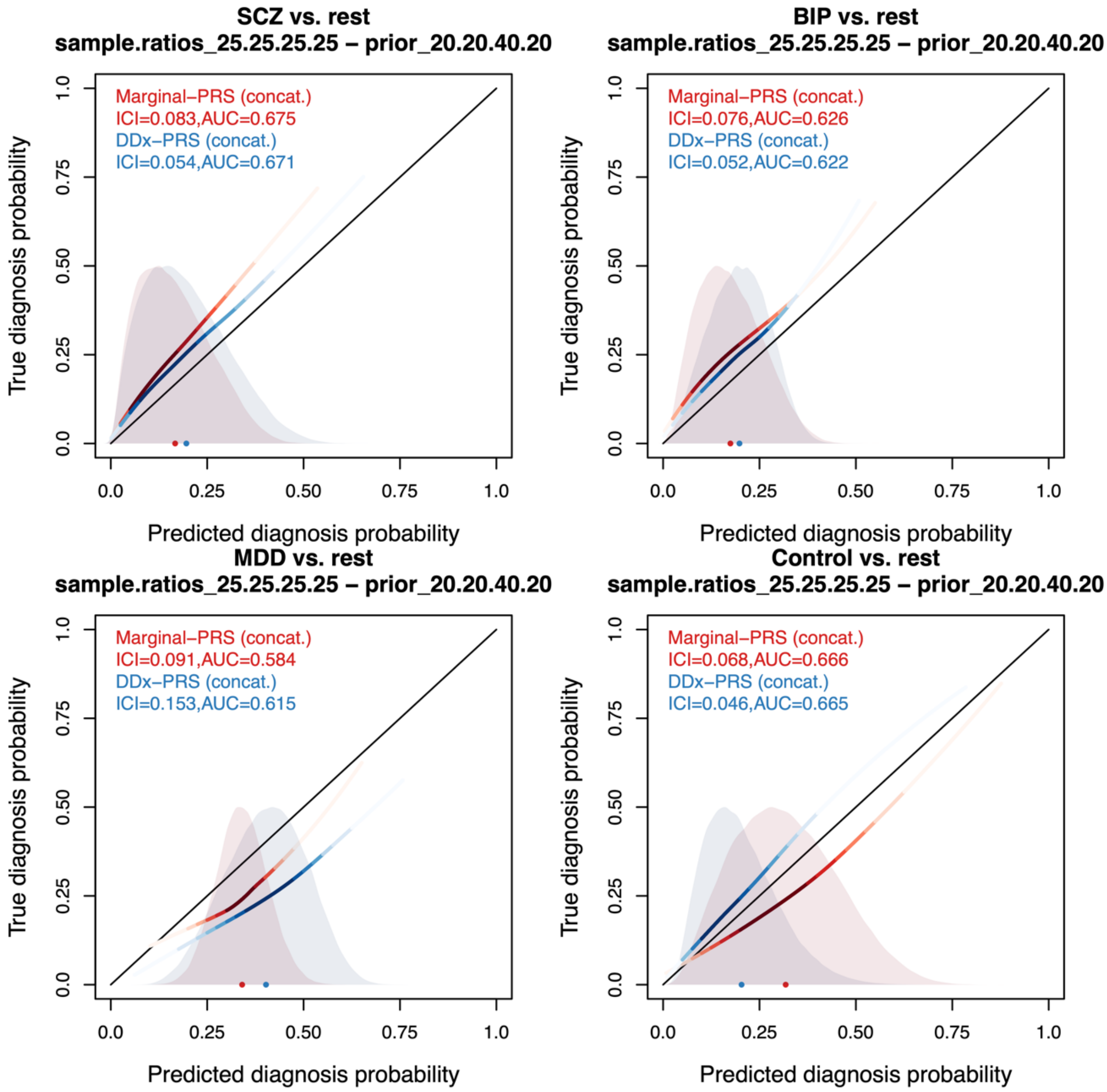

Supplementary Figure 6n. Calibration curves of DDx-PRS and Marginal-PRS for different sample size ratios in simulations. Panel n. We report the true diagnosis probability vs. the predicted diagnosis probability for the comparisons of schizophrenia (SCZ) vs. rest, bipolar disorder (BIP) vs. rest, major depressive disorder (MDD) vs. rest, and control vs. rest, for two methods: Marginal-PRS (red) and DDx-PRS (blue). Panels a-k report results based on correctly specified prior probabilities (i.e., the prior matches the sample size ratios); Panels l-s report results based on misspecified priors. An overview of the results of this Figure is provided in Supplementary Figure 7.

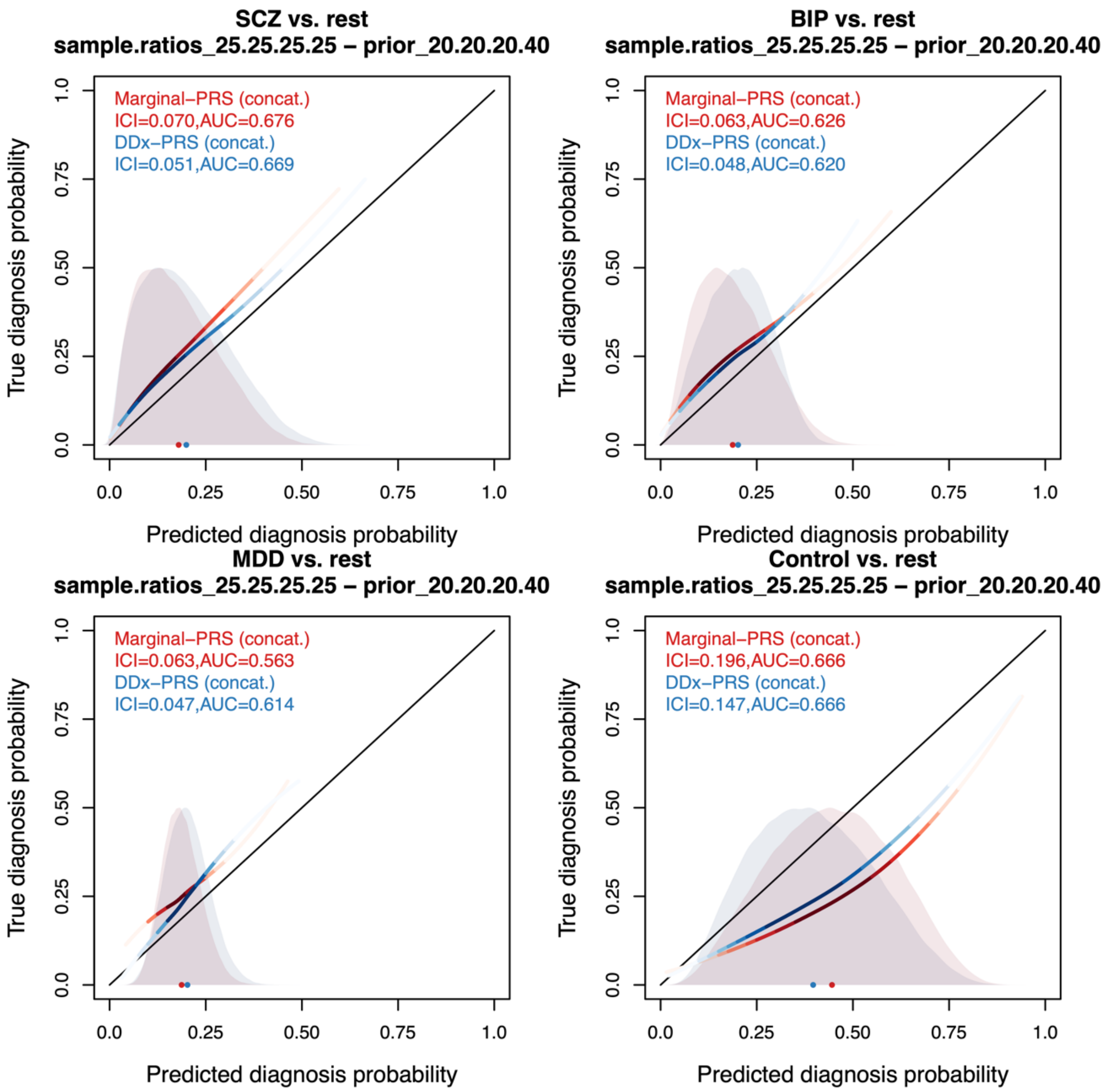

Supplementary Figure 6o. Calibration curves of DDx-PRS and Marginal-PRS for different sample size ratios in simulations. Panel o. We report the true diagnosis probability vs. the predicted diagnosis probability for the comparisons of schizophrenia (SCZ) vs. rest, bipolar disorder (BIP) vs. rest, major depressive disorder (MDD) vs. rest, and control vs. rest, for two methods: Marginal-PRS (red) and DDx-PRS (blue). Panels a-k report results based on correctly specified prior probabilities (i.e., the prior matches the sample size ratios); Panels l-s report results based on misspecified priors. An overview of the results of this Figure is provided in Supplementary Figure 7.

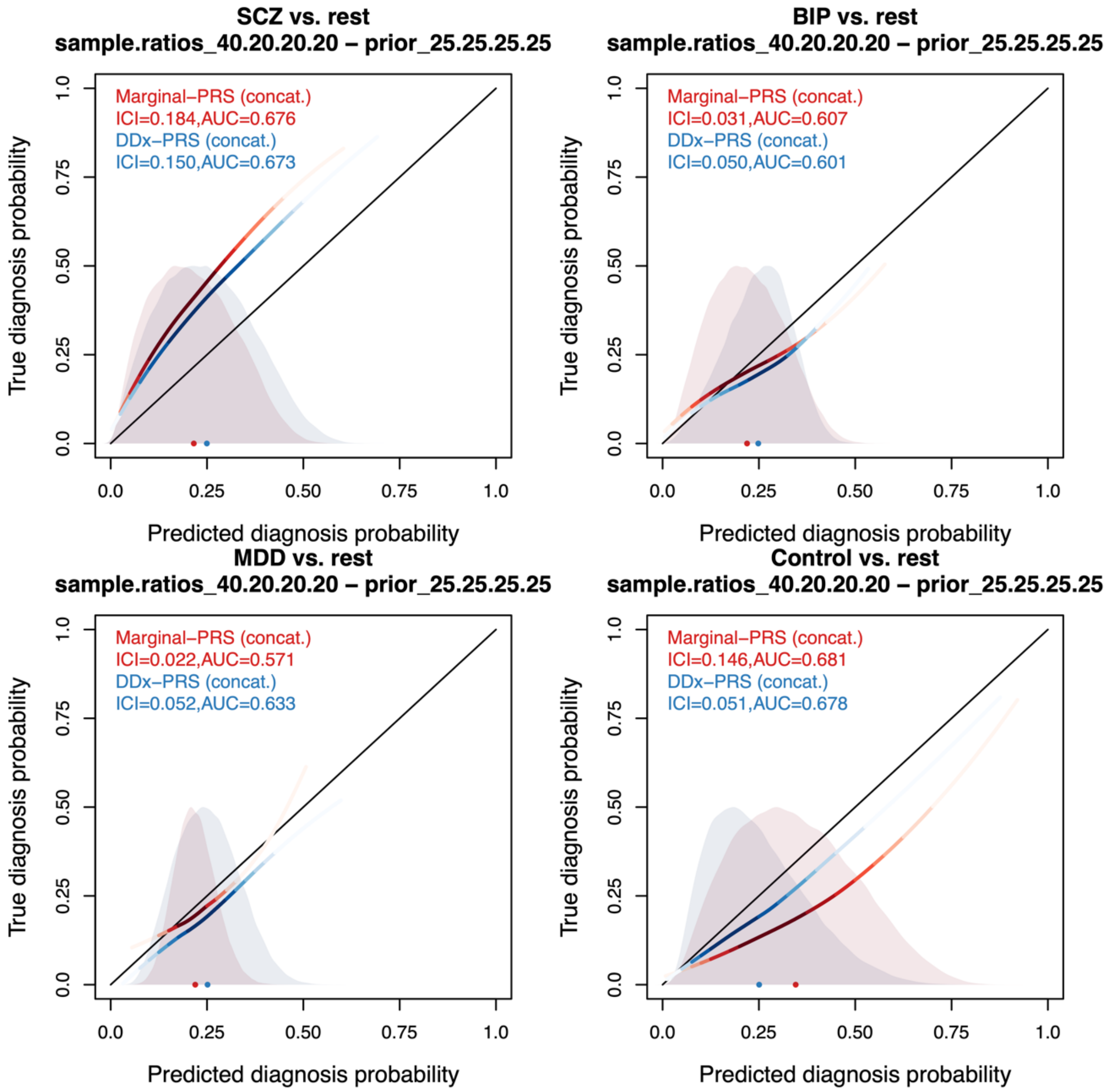

Supplementary Figure 6p. Calibration curves of DDx-PRS and Marginal-PRS for different sample size ratios in simulations. Panel p. We report the true diagnosis probability vs. the predicted diagnosis probability for the comparisons of schizophrenia (SCZ) vs. rest, bipolar disorder (BIP) vs. rest, major depressive disorder (MDD) vs. rest, and control vs. rest, for two methods: Marginal-PRS (red) and DDx-PRS (blue). Panels a-k report results based on correctly specified prior probabilities (i.e., the prior matches the sample size ratios); Panels l-s report results based on misspecified priors. An overview of the results of this Figure is provided in Supplementary Figure 7.

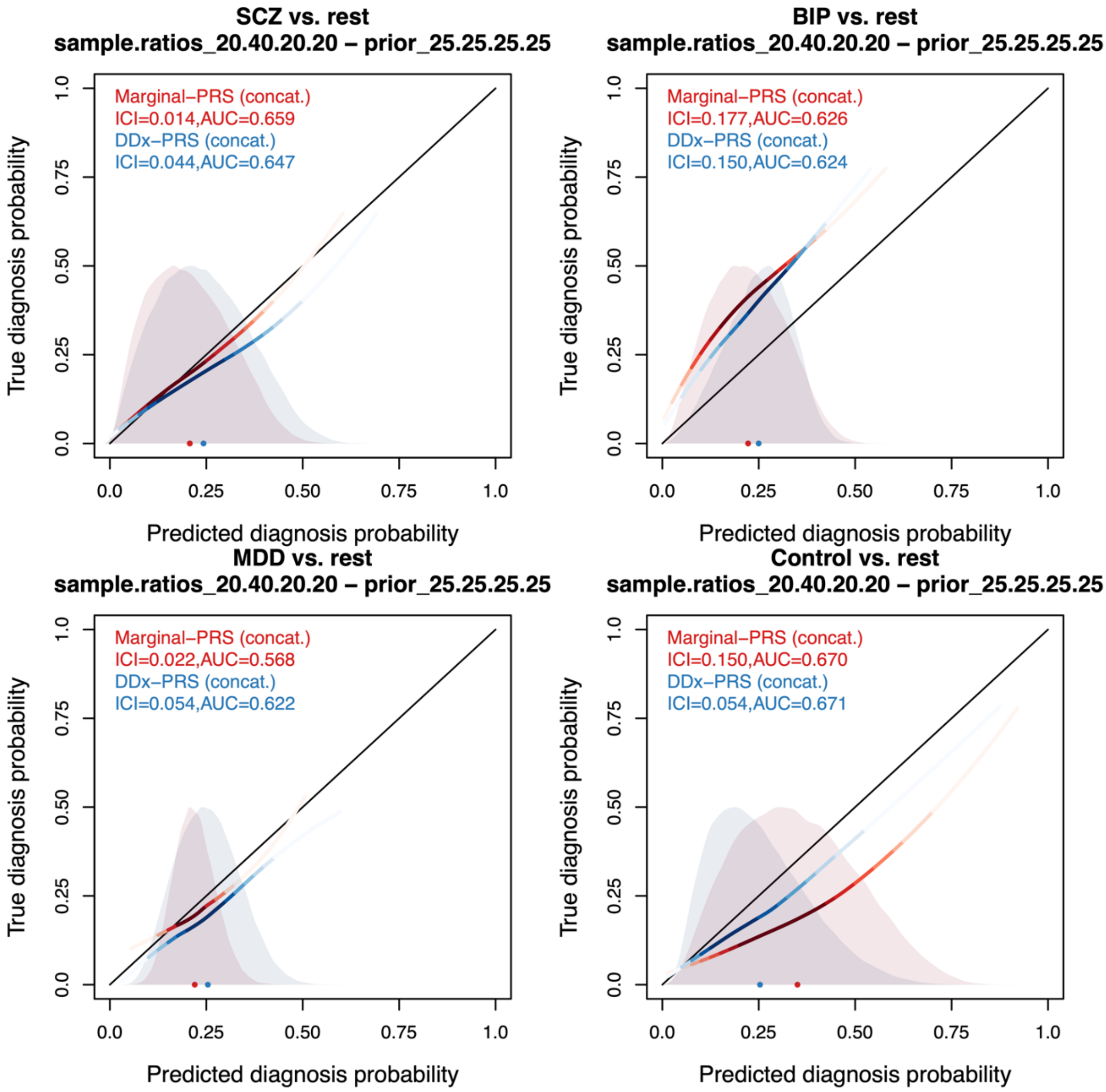

Supplementary Figure 6q. Calibration curves of DDx-PRS and Marginal-PRS for different sample size ratios in simulations. Panel q. We report the true diagnosis probability vs. the predicted diagnosis probability for the comparisons of schizophrenia (SCZ) vs. rest, bipolar disorder (BIP) vs. rest, major depressive disorder (MDD) vs. rest, and control vs. rest, for two methods: Marginal-PRS (red) and DDx-PRS (blue). Panels a-k report results based on correctly specified prior probabilities (i.e., the prior matches the sample size ratios); Panels l-s report results based on misspecified priors. An overview of the results of this Figure is provided in Supplementary Figure 7.

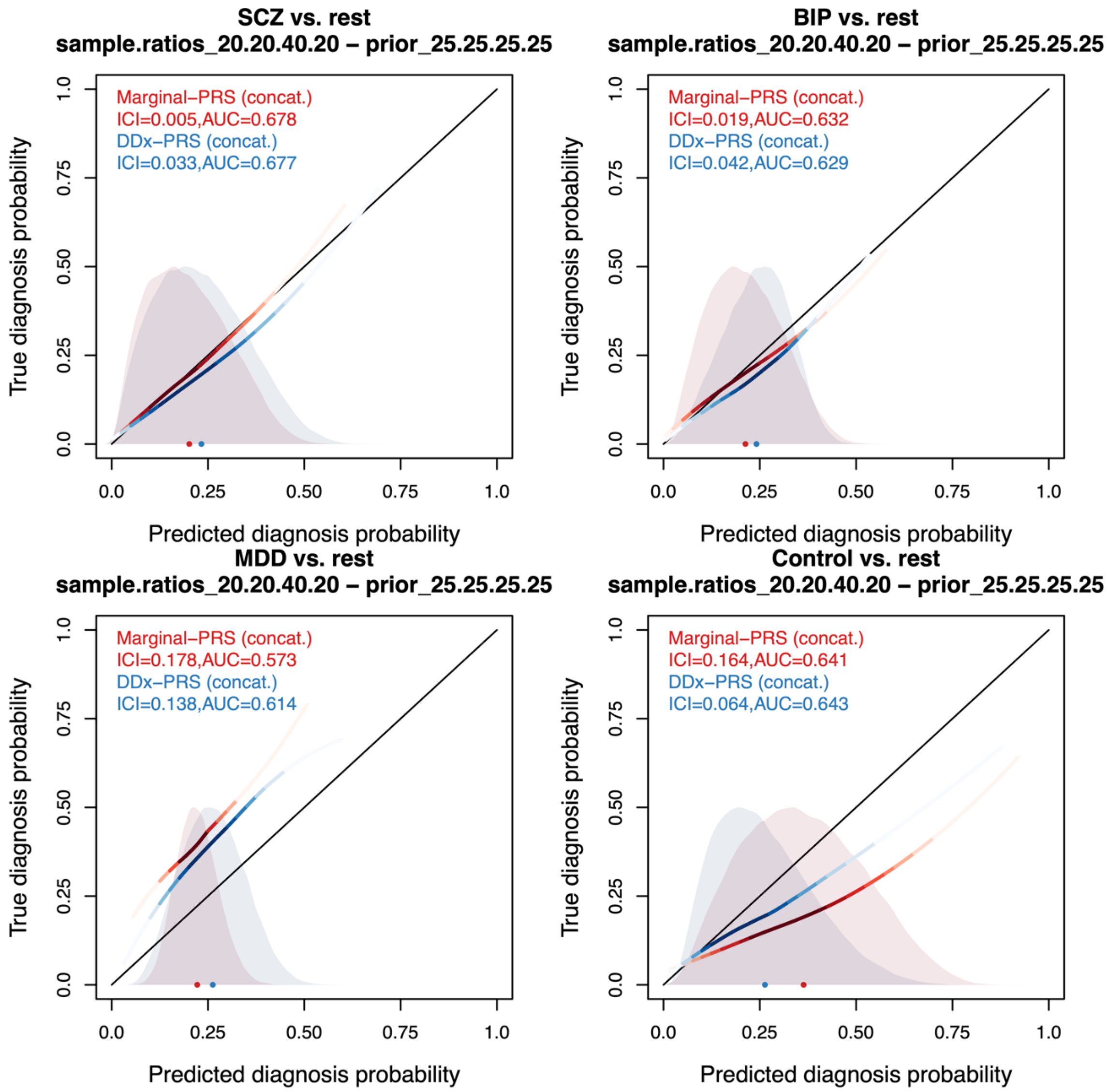

Supplementary Figure 6r. Calibration curves of DDx-PRS and Marginal-PRS for different sample size ratios in simulations. Panel r. We report the true diagnosis probability vs. the predicted diagnosis probability for the comparisons of schizophrenia (SCZ) vs. rest, bipolar disorder (BIP) vs. rest, major depressive disorder (MDD) vs. rest, and control vs. rest, for two methods: Marginal-PRS (red) and DDx-PRS (blue). Panels a-k report results based on correctly specified prior probabilities (i.e., the prior matches the sample size ratios); Panels l-s report results based on misspecified priors. An overview of the results of this Figure is provided in Supplementary Figure 7.

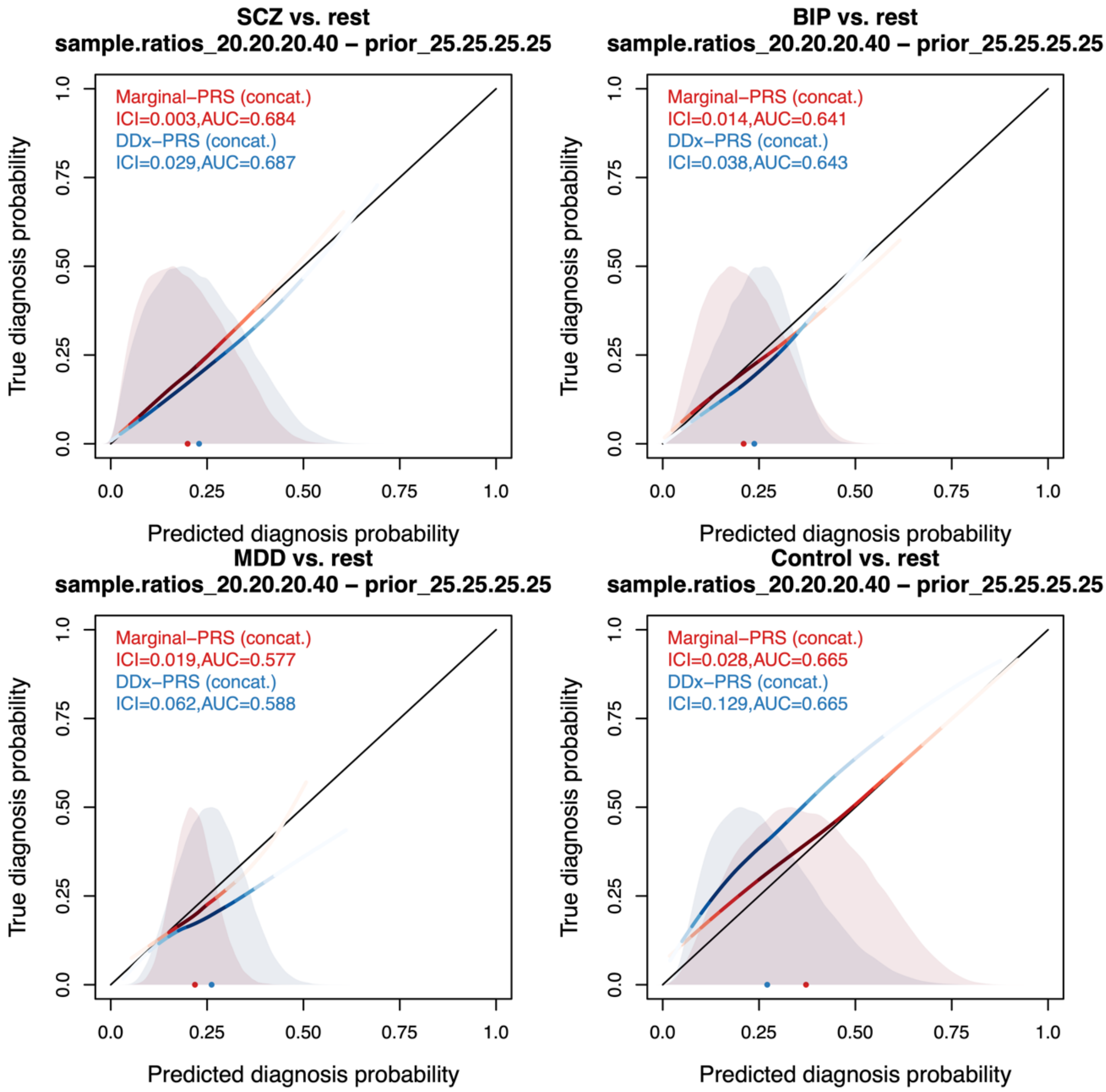

Supplementary Figure 6s. Calibration curves of DDx-PRS and Marginal-PRS for different sample size ratios in simulations. Panel s. We report the true diagnosis probability vs. the predicted diagnosis probability for the comparisons of schizophrenia (SCZ) vs. rest, bipolar disorder (BIP) vs. rest, major depressive disorder (MDD) vs. rest, and control vs. rest, for two methods: Marginal-PRS (red) and DDx-PRS (blue). Panels a-k report results based on correctly specified prior probabilities (i.e., the prior matches the sample size ratios); Panels l-s report results based on misspecified priors. An overview of the results of this Figure is provided in Supplementary Figure 7.

A. Heatmap of ICI in simulations

Sample size ratios % (SCZ_BIP_MDD_Contr)	Prior % (SCZ_BIP_MDD_Contr)	ICI: DDx-PRS (concat.) SCZ vs.rest	BIP vs.rest	MDD vs.rest	Contr. vs.rest	ICI: Marginal-PRS (concat.) SCZ vs.rest	BIP vs.rest	MDD vs.rest	Contr. vs.rest	signed ICI: DDx-PRS (concat.) SCZ vs.rest	BIP vs.rest	MDD vs.rest	Contr. vs.rest	signed ICI: Marginal-PRS (concat.) SCZ vs.rest	BIP vs.rest	MDD vs.rest	Contr. vs.rest	Figure Panel
25_25_25_25	matching	0.011	0.006	0.009	0.015	0.044	0.034	0.030	0.108	0.011	0.005	-0.008	-0.010	0.044	0.034	0.030	-0.108	a
40_20_20_20	matching	0.015	0.007	0.010	0.016	0.077	0.029	0.024	0.123	0.015	0.003	-0.008	-0.011	0.077	0.022	0.024	-0.123	b
20_40_20_20	matching	0.010	0.007	0.009	0.016	0.032	0.066	0.024	0.122	0.010	0.007	-0.007	-0.010	0.032	0.066	0.024	-0.121	c
20_20_40_20	matching	0.010	0.006	0.011	0.011	0.038	0.029	0.056	0.122	0.010	0.005	-0.009	-0.006	0.038	0.029	0.056	-0.122	d
20_20_20_40	matching	0.011	0.007	0.005	0.020	0.027	0.020	0.015	0.061	0.010	0.005	-0.005	-0.011	0.027	0.019	0.015	-0.060	e
85_5_5_5	matching	0.011	0.007	0.005	0.008	0.089	0.022	0.011	0.078	0.011	0.000	-0.005	-0.006	0.089	-0.010	-0.001	-0.078	f
5_85_5_5	matching	0.004	0.006	0.004	0.006	0.013	0.082	0.011	0.077	0.003	0.005	-0.003	-0.004	-0.004	0.082	-0.001	-0.077	g
5_5_85_5	matching	0.004	0.003	0.007	0.002	0.005	0.006	0.067	0.073	0.003	0.002	-0.005	0.000	0.005	0.002	0.067	-0.073	h
5_5_5_85	matching	0.006	0.004	0.001	0.012	0.003	0.003	0.002	0.007	0.003	0.001	-0.001	-0.003	0.003	0.001	-0.001	-0.004	i
30_30_30_10	matching	0.012	0.007	0.015	0.008	0.066	0.055	0.051	0.171	0.012	0.005	-0.012	-0.006	0.066	0.055	0.051	-0.171	j
33_33_33_0	matching	0.012	0.011	0.020	n/a	0.018	0.011	0.034	n/a	0.012	0.004	-0.017	n/a	0.018	0.000	-0.019	n/a	k
25_25_25_25	40_20_20_20	0.121	0.055	0.035	0.031	0.062	0.075	0.074	0.088	-0.121	0.055	0.035	0.030	-0.062	0.075	0.074	-0.087	l
25_25_25_25	20_40_20_20	0.062	0.136	0.039	0.034	0.083	0.077	0.074	0.081	0.062	-0.136	0.039	0.034	0.083	-0.077	0.074	-0.080	m
25_25_25_25	20_20_40_20	0.054	0.052	0.153	0.046	0.083	0.076	0.091	0.068	0.054	0.052	-0.153	0.046	0.083	0.076	-0.091	-0.068	n
25_25_25_25	20_20_20_40	0.051	0.049	0.047	0.147	0.07	0.063	0.063	0.196	0.051	0.049	0.047	-0.147	0.070	0.063	0.063	-0.196	o
40_20_20_20	25_25_25_25	0.151	0.05	0.052	0.051	0.184	0.031	0.022	0.146	0.151	-0.049	-0.052	-0.051	0.184	-0.019	-0.020	-0.146	p
20_40_20_20	25_25_25_25	0.044	0.15	0.055	0.054	0.014	0.178	0.022	0.15	-0.042	0.150	-0.055	-0.054	-0.007	0.178	-0.020	-0.150	q
20_20_40_20	25_25_25_25	0.033	0.042	0.138	0.064	0.005	0.019	0.178	0.164	-0.033	-0.042	0.138	-0.063	-0.001	-0.013	0.178	-0.164	r
20_20_20_40	25_25_25_25	0.029	0.038	0.062	0.129	0.003	0.015	0.019	0.028	-0.029	-0.038	-0.062	0.129	0.001	-0.010	-0.019	0.028	s

B. Heatmap of AUC and MCC in simulations

Sample size ratios % (SCZ_BIP_MDD_Contr)	Prior % (SCZ_BIP_MDD_Contr)	DDx-PRS (concat.) SCZ vs. rest AUC	BIP vs. rest AUC	MDD vs. rest AUC	Control vs. rest AUC	Mean AUC	Multi-class MCC	Marginal-PRS (concat.) SCZ vs. rest AUC	BIP vs. rest AUC	MDD vs. rest AUC	Control vs. rest AUC	Mean AUC	Multi-class MCC	Figure Panel
25_25_25_25	matching	0.672	0.624	0.615	0.667	0.645	0.163	0.675	0.626	0.573	0.666	0.635	0.148	a
40_20_20_20	matching	0.673	0.611	0.633	0.680	0.649	0.168	0.674	0.606	0.568	0.679	0.632	0.171	b
20_40_20_20	matching	0.652	0.624	0.622	0.672	0.642	0.139	0.659	0.627	0.565	0.670	0.630	0.144	c
20_20_40_20	matching	0.676	0.629	0.614	0.644	0.641	0.123	0.678	0.632	0.583	0.644	0.634	0.094	d
20_20_20_40	matching	0.685	0.640	0.595	0.665	0.646	0.155	0.686	0.642	0.570	0.665	0.641	0.142	e
85_5_5_5	matching	0.675	0.632	0.686	0.718	0.678	0.071	0.660	0.548	0.517	0.712	0.609	0.092	f
5_85_5_5	matching	0.627	0.624	0.647	0.689	0.647	0.030	0.619	0.620	0.519	0.684	0.611	0.054	g
5_5_85_5	matching	0.692	0.648	0.614	0.606	0.640	0.005	0.689	0.646	0.608	0.606	0.637	0.000	h
5_5_5_85	matching	0.722	0.686	0.591	0.663	0.666	0.016	0.716	0.682	0.591	0.662	0.663	0.006	i
30_30_30_10	matching	0.658	0.610	0.645	0.667	0.645	0.163	0.663	0.611	0.582	0.666	0.631	0.133	j
33_33_33_0	matching	0.650	0.605	0.671	n/a	0.642	0.186	0.644	0.605	0.665	n/a	0.638	0.181	k
25_25_25_25	40_20_20_20	0.672	0.626	0.613	0.667	0.645	0.147	0.673	0.626	0.571	0.662	0.633	0.150	l
25_25_25_25	20_40_20_20	0.676	0.624	0.612	0.666	0.644	0.128	0.675	0.627	0.571	0.664	0.634	0.134	m
25_25_25_25	20_20_40_20	0.671	0.622	0.615	0.665	0.643	0.118	0.675	0.626	0.584	0.666	0.638	0.108	n
25_25_25_25	20_20_20_40	0.669	0.620	0.614	0.667	0.642	0.137	0.676	0.626	0.563	0.666	0.633	0.127	o
40_20_20_20	25_25_25_25	0.673	0.601	0.633	0.679	0.646	0.170	0.676	0.607	0.571	0.681	0.634	0.149	p
20_40_20_20	25_25_25_25	0.647	0.624	0.622	0.671	0.641	0.150	0.659	0.626	0.568	0.670	0.631	0.137	q
20_20_40_20	25_25_25_25	0.677	0.629	0.614	0.643	0.641	0.149	0.678	0.632	0.573	0.641	0.631	0.123	r
20_20_20_40	25_25_25_25	0.687	0.643	0.588	0.665	0.646	0.167	0.684	0.641	0.577	0.665	0.642	0.163	s

Supplementary Figure 7. Heatmap of results of DDx-PRS and Marginal-PRS for different sample size ratios in simulations. We report the results for DDx-PRS and Marginal PRS for different sets of sample size ratios and assumed priors for schizophrenia (SCZ), bipolar disorder (BIP), major depressive disorder (MDD) and controls. The listed Figure Panels refer to Supplementary Figure 6. Panel a reports the Integrated Calibration Index (ICI), the weighted average absolute difference between true and predicted disorder risk, based on the smoothened calibration curve. We also report the signed ICI, the weighted average signed difference between true and predicted disorder risk. The signed ICI is useful for assessing systematic overestimation/underestimation in predicted probabilities (but is not appropriate to assess overall calibration as overestimation and underestimation of risk can cancel each other). For example, the signed ICI shows that assuming a too large prior (for e.g. SCZ, in the row corresponding to Supplementary Figure 6 Panel l), results in a too larger posterior probability for the same diagnostic category (corresponding to a signed ICI < 0). Panel b reports the AUC (area under the ROC curve) and the multi-class Matthews Correlation Coefficient (MCC).

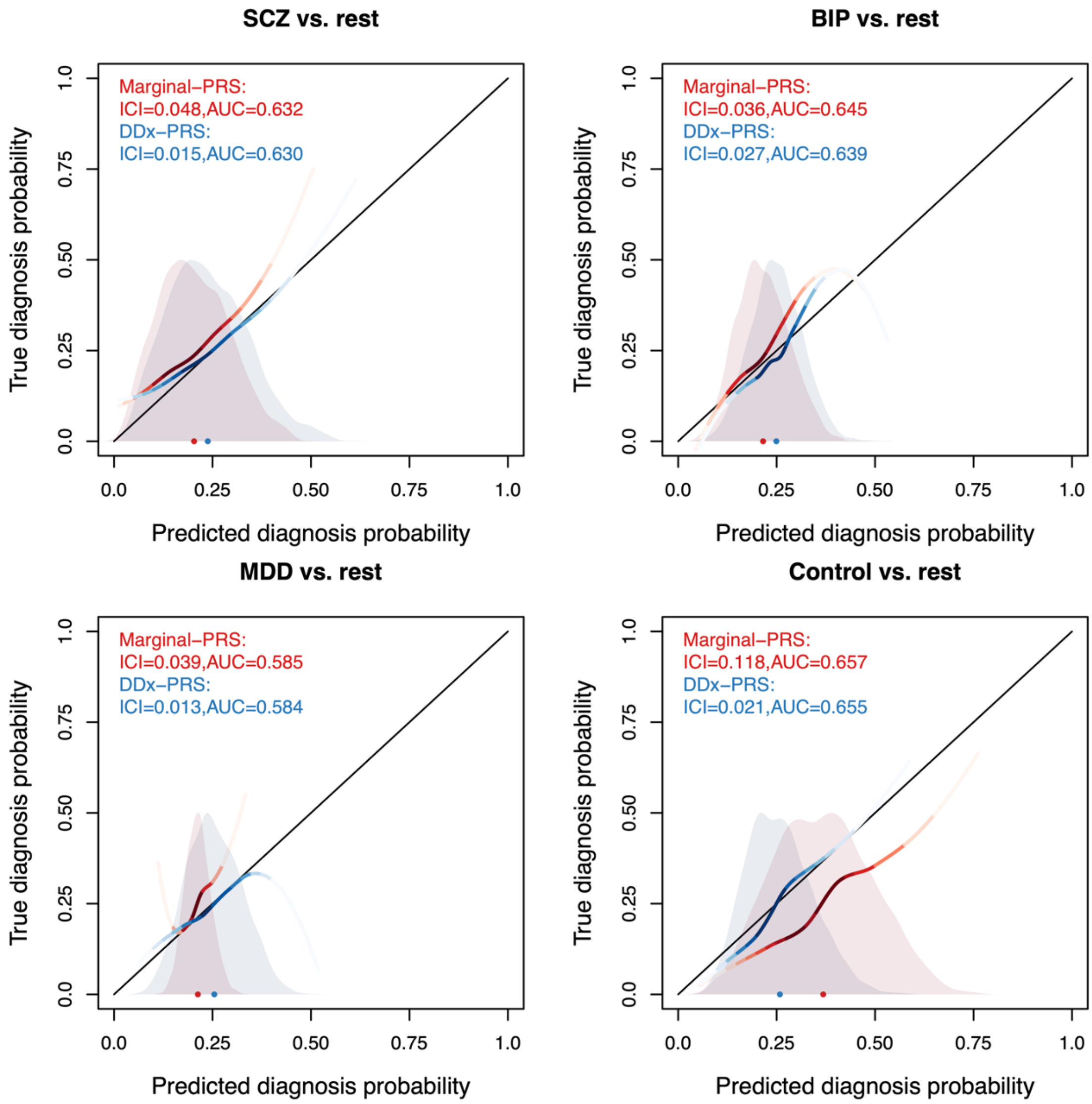

Supplementary Figure 8a. Calibration and accuracy of DDx-PRS and Marginal-PRS in individual cohorts in PGC data. Panel a – cohort GER. This Figure is analogue to main Figure 4 and reports the calibration curves for the four PGC cohorts. Every panel reports the true diagnosis probability vs. the predicted diagnosis probability for the comparisons of schizophrenia (SCZ) vs. rest, bipolar disorder (BIP) vs. rest, major depressive disorder (MDD) vs. rest, and control vs. rest, for two methods: Marginal-PRS (red) and DDx-PRS (blue). The calibration curves are plotted using a locally estimated scatterplot smoothing (loess)-based smoothing function. Results are based on the PGC test cohort GER (N=3,368, with 842 for each of SCZ, BIP, MDD and control). The Integrated Calibration Index (ICI) equals the average absolute difference between the calibration curve and the line y=x (plotted in black), weighted by the density of the predicted diagnosis probabilities (displayed in the shaded histograms and in the color-intensity of the calibration curves). AUC, area under the ROC curve. These secondary analyses show that similar results were observed for all four cohorts individually as for the mean results in Figure 3.

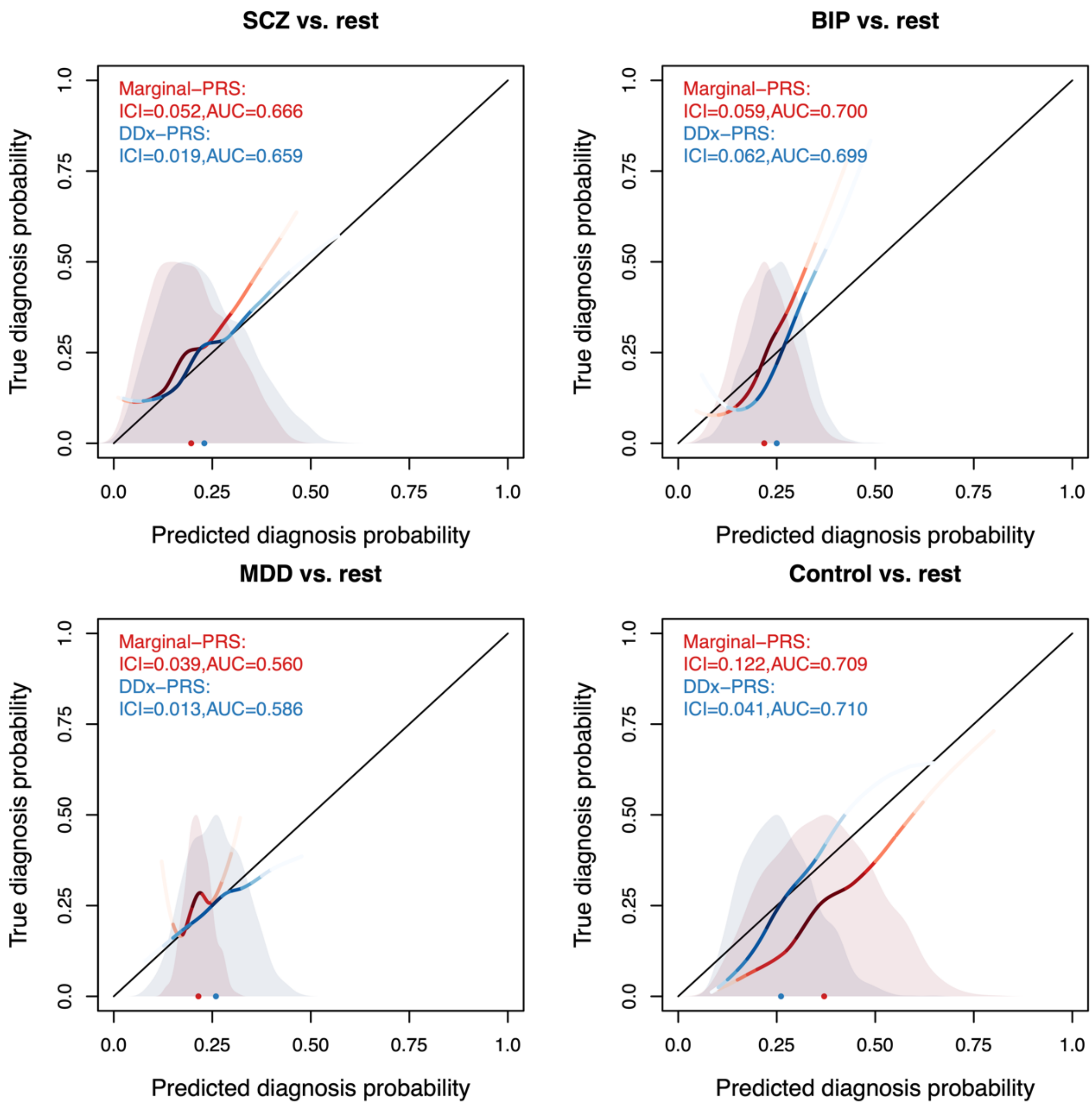

Supplementary Figure 8b. Calibration and accuracy of DDx-PRS and Marginal-PRS in individual cohorts in PGC data. Panel b – cohort UK1. This Figure is analogue to main Figure 4 and reports the calibration curves for the four PGC cohorts. Every panel reports the true diagnosis probability vs. the predicted diagnosis probability for the comparisons of schizophrenia (SCZ) vs. rest, bipolar disorder (BIP) vs. rest, major depressive disorder (MDD) vs. rest, and control vs. rest, for two methods: Marginal-PRS (red) and DDx-PRS (blue). The calibration curves are plotted using a locally estimated scatterplot smoothing (loess)-based smoothing function. Results are based on the PGC test cohort UK1 (N=1,136, with 284 for each of SCZ, BIP, MDD and control). The Integrated Calibration Index (ICI) equals the average absolute difference between the calibration curve and the line y=x (plotted in black), weighted by the density of the predicted diagnosis probabilities (displayed in the shaded histograms and in the color-intensity of the calibration curves). AUC, area under the ROC curve. These secondary analyses show that similar results were observed for all four cohorts individually as for the mean results in Figure 3.

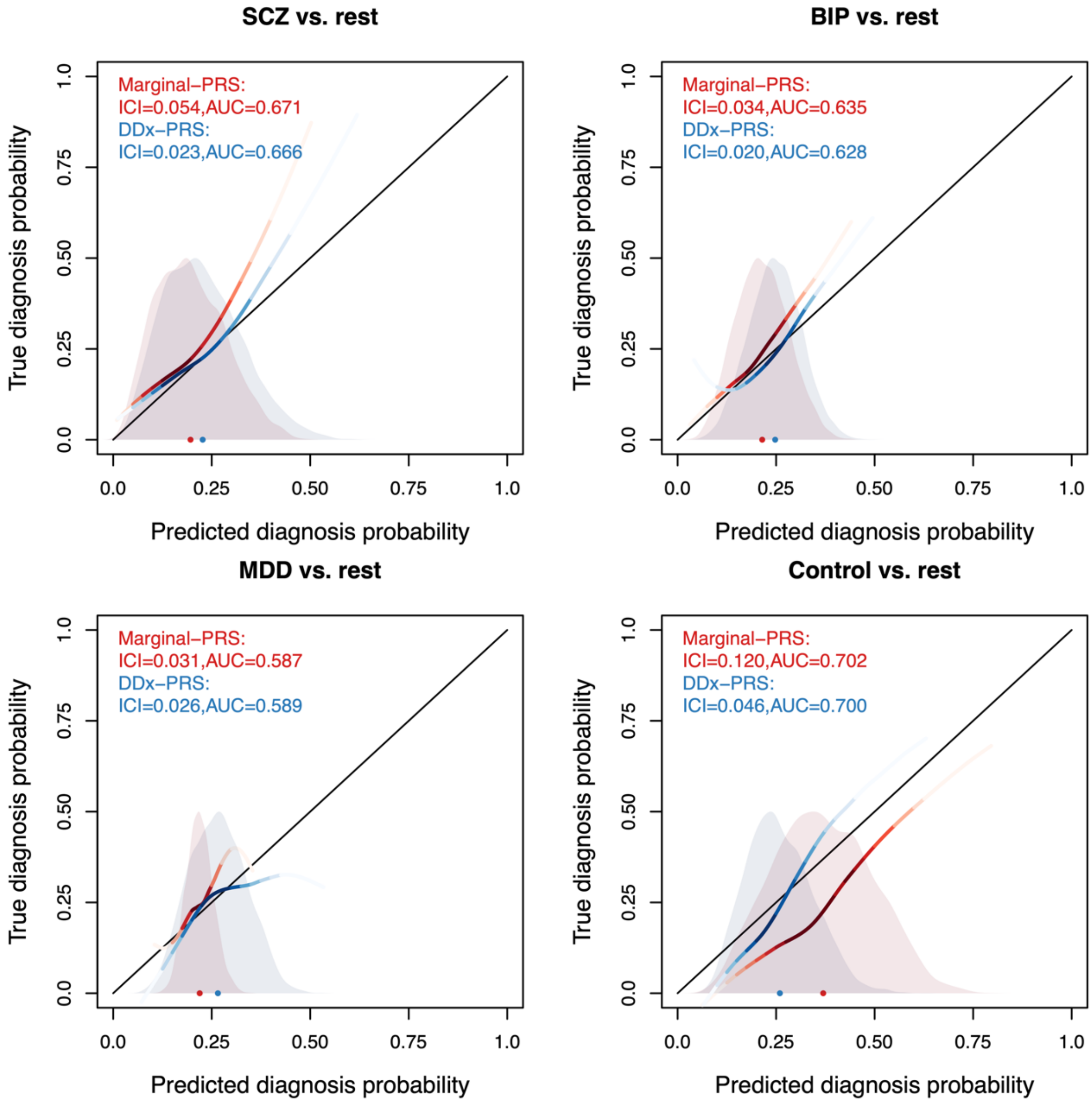

Supplementary Figure 8c. Calibration and accuracy of DDx-PRS and Marginal-PRS in individual cohorts in PGC data. Panel c – UK2. This Figure is analogue to main Figure 4 and reports the calibration curves for the four PGC cohorts. Every panel reports the true diagnosis probability vs. the predicted diagnosis probability for the comparisons of schizophrenia (SCZ) vs. rest, bipolar disorder (BIP) vs. rest, major depressive disorder (MDD) vs. rest, and control vs. rest, for two methods: Marginal-PRS (red) and DDx-PRS (blue). The calibration curves are plotted using a locally estimated scatterplot smoothing (loess)-based smoothing function. Results are based on the PGC test cohort UK2 (N=6,080, with 520 for each of SCZ, BIP, MDD and control). The Integrated Calibration Index (ICI) equals the average absolute difference between the calibration curve and the line y=x (plotted in black), weighted by the density of the predicted diagnosis probabilities (displayed in the shaded histograms and in the color-intensity of the calibration curves). AUC, area under the ROC curve. These secondary analyses show that similar results were observed for all four cohorts individually as for the mean results in Figure 3.

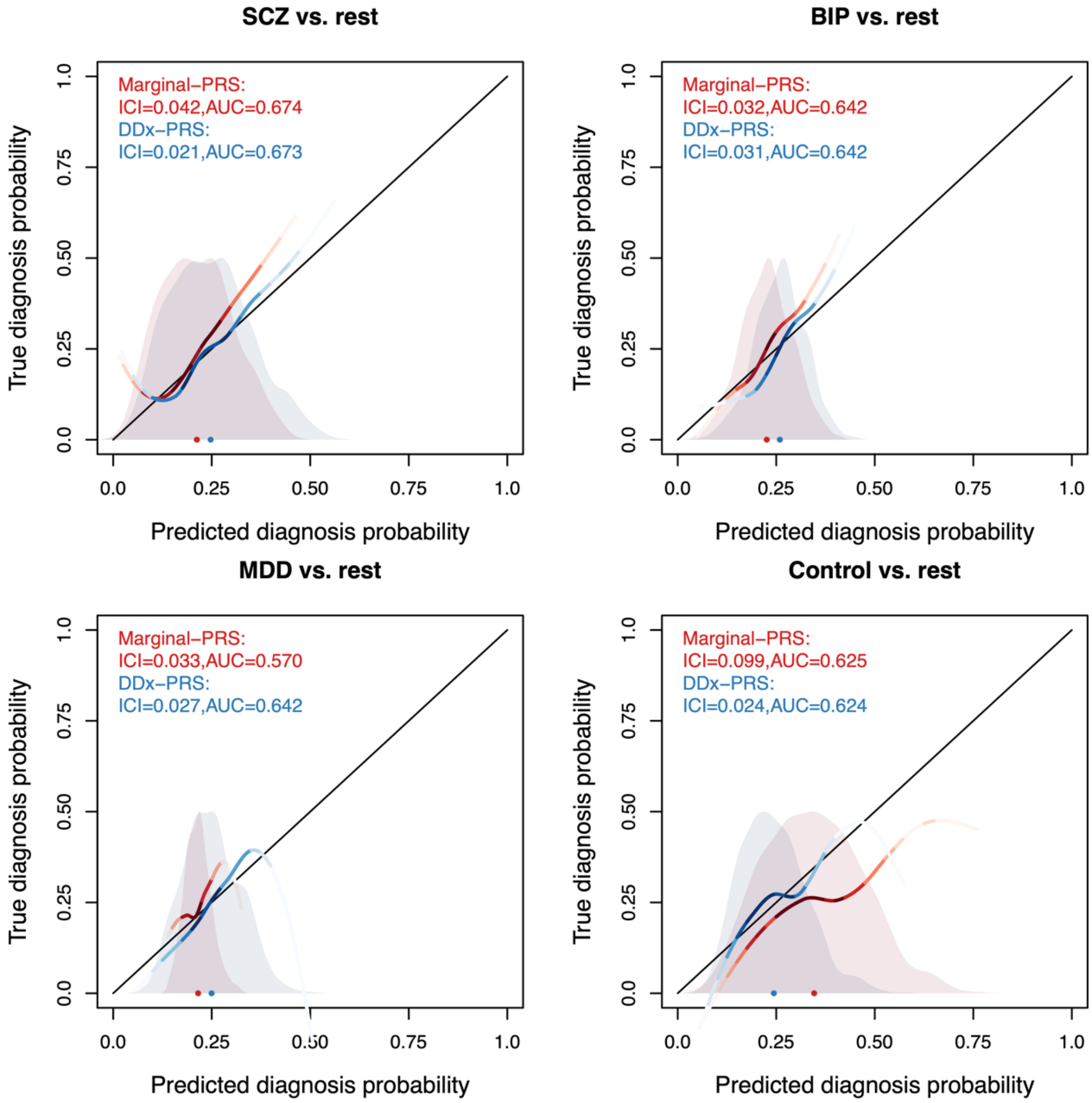

Supplementary Figure 8d. Calibration and accuracy of DDx-PRS and Marginal-PRS in individual cohorts in PGC data. Panel d – USA. This Figure is analogue to main Figure 4 and reports the calibration curves for the four PGC cohorts. Every panel reports the true diagnosis probability vs. the predicted diagnosis probability for the comparisons of schizophrenia (SCZ) vs. rest, bipolar disorder (BIP) vs. rest, major depressive disorder (MDD) vs. rest, and control vs. rest, for two methods: Marginal-PRS (red) and DDx-PRS (blue). The calibration curves are plotted using a locally estimated scatterplot smoothing (loess)-based smoothing function. Results are based on the PGC test cohort USA (N=876, with 219 for each of SCZ, BIP, MDD and control). The Integrated Calibration Index (ICI) equals the average absolute difference between the calibration curve and the line y=x (plotted in black), weighted by the density of the predicted diagnosis probabilities (displayed in the shaded histograms and in the color-intensity of the calibration curves). AUC, area under the ROC curve. These secondary analyses show that similar results were observed for all four cohorts individually as for the mean results in Figure 3.

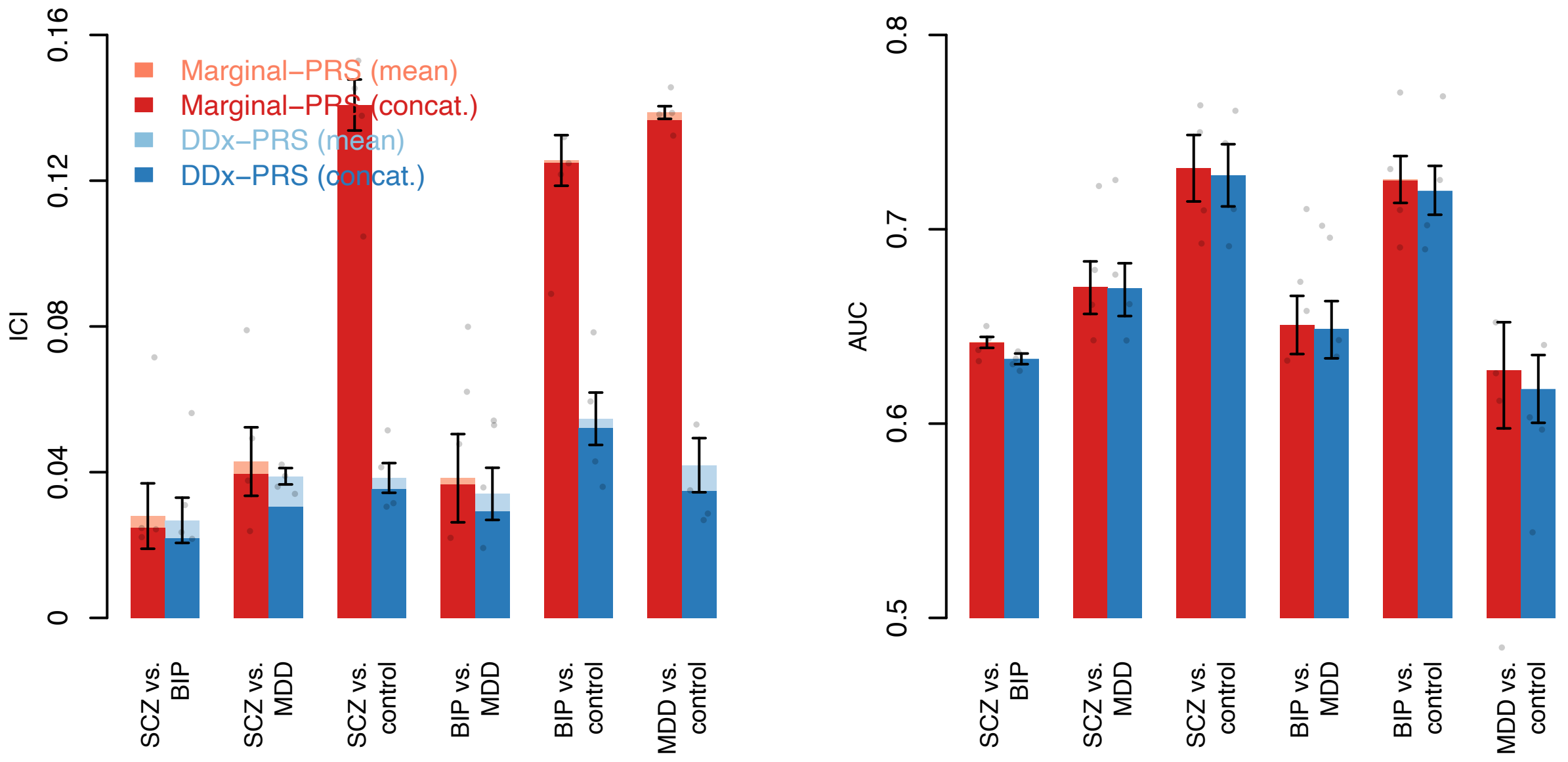

Supplementary Figure 9. Calibration and accuracy of DDx-PRS and Marginal-PRS for comparisons across all 6 pairs of diagnostic categories in PGC data. This Figure accompanies main Figure 3 and reports the calibration (Integrated Calibration Index; ICI) and accuracy (area under the ROC curve; AUC) for the comparisons of schizophrenia (SCZ) vs. bipolar disorder (BIP), SCZ vs. major depressive disorder (MDD), SCZ vs. control, BIP vs. MDD, BIP vs. control and MDD vs. control, for two methods: Marginal-PRS (red) and DDx-PRS (blue). The training data consisted of case-control GWAS summary statistics for SCZ, BIP and MDD; the test data consisted of independent PGC samples subdivided in four cohorts that were matched with respect to country and genotyping platform (total N=11,460, with 2,865 for each of SCZ, BIP, MDD and control). The mean values across these four test cohorts are displayed in light red and light blue, and results based on data concatenated across these four test cohorts are displayed in dark red and dark blue. Error bars denote standard errors. Numerical results are reported in Supplementary Table 12. These secondary analyses show DDx-PRS yielded better calibration than Marginal-PRS and similar prediction accuracy across all 6 pairs of diagnostic categories, in line with the 4 comparisons of one diagnostic category vs. rest displayed in main Figure 3.

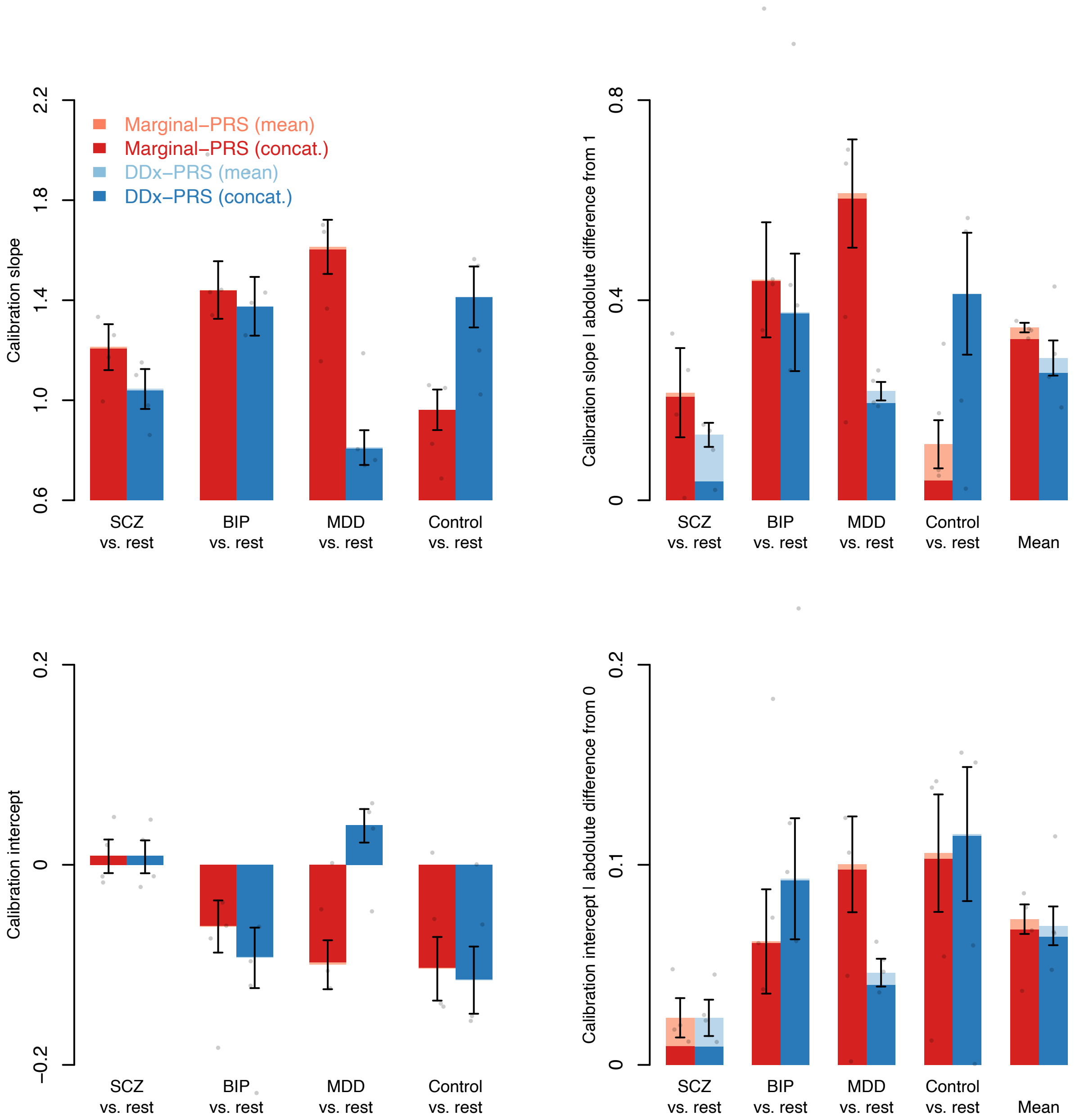

Supplementary Figure 10. Calibration slopes of DDx-PRS and Marginal-PRS in PGC data. This Figure accompanies main Figure 3 and reports the calibration slopes and intercepts for the comparisons of schizophrenia (SCZ) vs. rest, bipolar disorder (BIP) vs. rest, major depressive disorder (MDD) vs. rest, and control vs. rest, for two methods: Marginal-PRS (red) and DDx-PRS (blue). The training data consisted of case-control GWAS summary statistics for SCZ, BIP and MDD; the test data consisted of independent PGC samples subdivided in four cohorts that were matched with respect to country and genotyping platform (total N=11,460, with 2,865 for each of SCZ, BIP, MDD and control). The mean values across these four test cohorts are displayed in light red and light blue, and results based on data concatenated across these four test cohorts are displayed in dark red and dark blue. Error bars denote standard errors. Perfect calibration would attain a slope of 1 and intercept of 0. Numerical results are reported in Supplementary Table 13. These secondary analyses show that DDx-PRS attained slightly better calibration than Marginal-PRS when considering the calibration slope and intercept instead of the ICI (main Figure 3). We note that the calibration slope shows more variance between comparisons than the ICI, because the slope is impacted by sparse subregions with poor calibration while the ICI is much more robust to this (also see Austin et al. 2019 Statistics in Medicine).

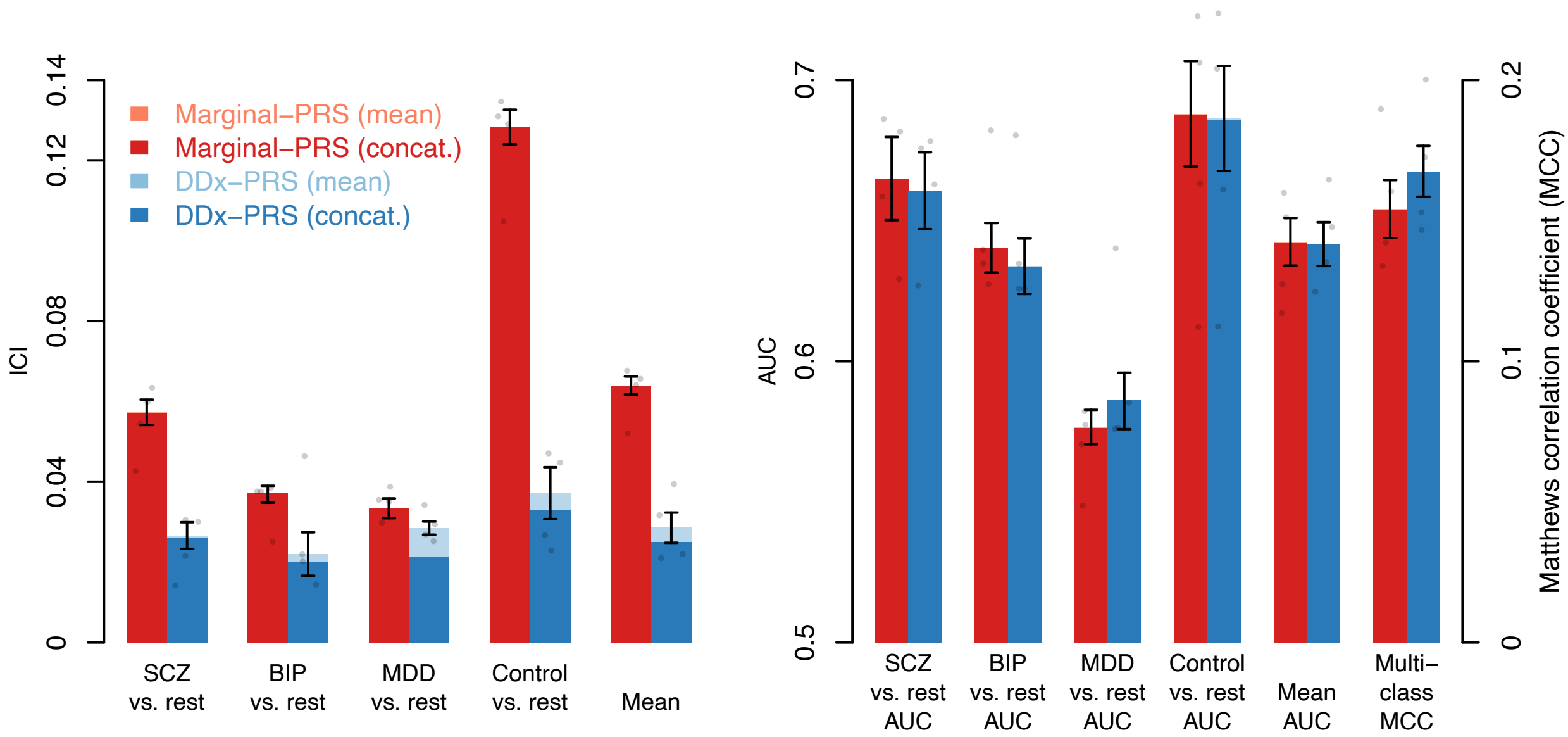

Supplementary Figure 11a. Calibration and accuracy of DDx-PRS and Marginal-PRS for different QC in PGC data. Panel a – looser SNP QC. This Figure is analogue to main Figure 3 with SNP QC of INFO>0.3 and MAF>0.01 (compared to INFO>0.9 and MAF>0.10 displayed in Figure 3). We report the calibration (Integrated Calibration Index; ICI) and accuracy (area under the ROC curve; AUC) for the comparisons of schizophrenia (SCZ) vs. rest, bipolar disorder (BIP) vs. rest, major depressive disorder (MDD) vs. rest, control vs. rest and the mean across these four comparisons, for two methods: Marginal-PRS (red) and DDx-PRS (blue). We also report the multi-class Matthews Correlation Coefficient (MCC). The training data consisted of case-control GWAS summary statistics for SCZ, BIP and MDD; the test data consisted of independent PGC samples subdivided in four cohorts that were matched with respect to country and genotyping platform (total N=11,460, with 2,865 for each of SCZ, BIP, MDD and control). The mean values across these four test cohorts are displayed in light red and light blue, and results based on data concatenated across these four test cohorts are displayed in dark red and dark blue. Error bars denote standard errors. Numerical results are reported in Supplementary Table 14. These secondary analyses show that arbitrary choices in QC had no impact on the presented results.

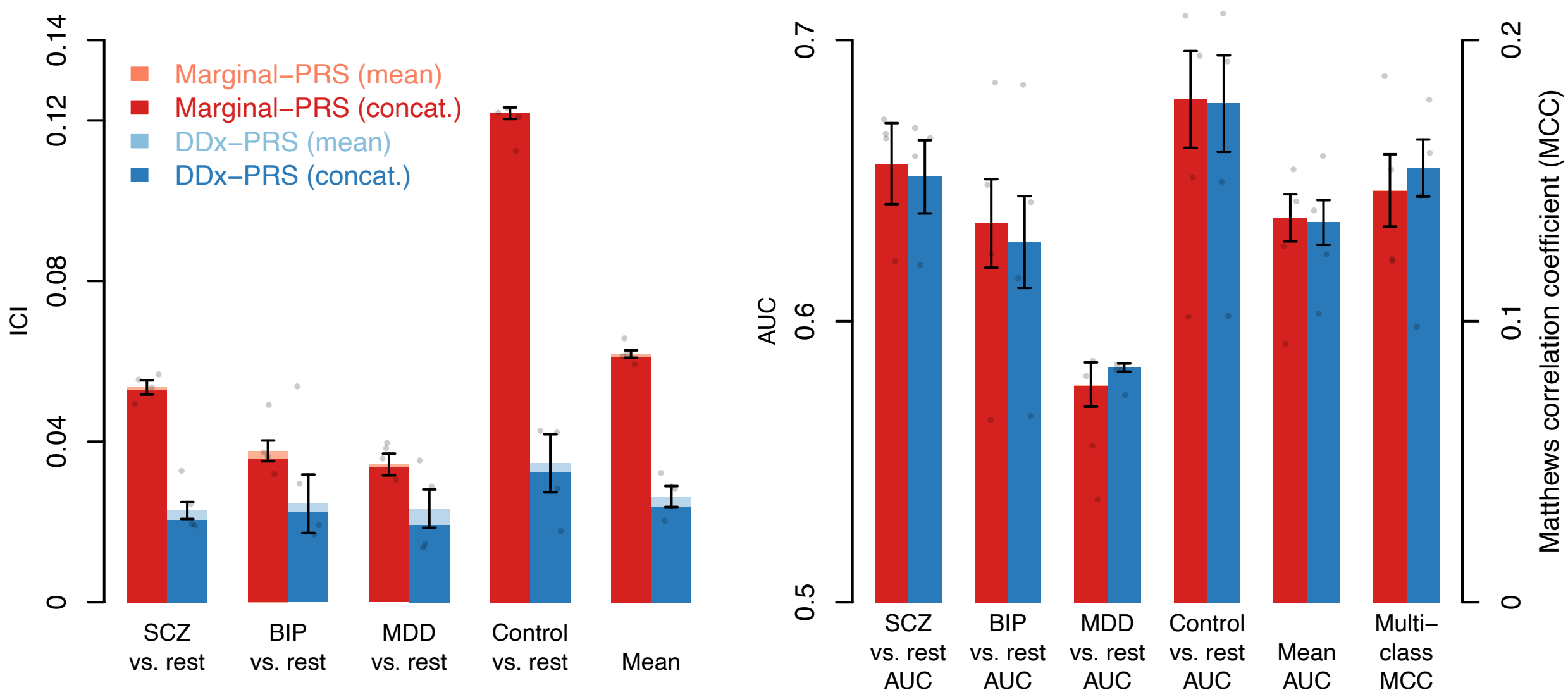

Supplementary Figure 11b. Calibration and accuracy of DDx-PRS and Marginal-PRS for different QC in PGC data. Panel b – stricter subject QC. This Figure is analogue to main Figure 3 with additional stricter subject QC (see Methods). We report the calibration (Integrated Calibration Index; ICI) and accuracy (area under the ROC curve; AUC) for the comparisons of schizophrenia (SCZ) vs. rest, bipolar disorder (BIP) vs. rest, major depressive disorder (MDD) vs. rest, control vs. rest and the mean across these four comparisons, for two methods: Marginal-PRS (red) and DDx-PRS (blue). We also report the multi-class Matthews Correlation Coefficient (MCC). The training data consisted of case-control GWAS summary statistics for SCZ, BIP and MDD; the test data consisted of independent PGC samples subdivided in four cohorts that were matched with respect to country and genotyping platform (total N=10,952, with 2,738 for each of SCZ, BIP, MDD and control; total N main analyses = 11,460). The mean values across these four test cohorts are displayed in light red and light blue, and results based on data concatenated across these four test cohorts are displayed in dark red and dark blue. Error bars denote standard errors. Numerical results are reported in Supplementary Table 14. These secondary analyses show that arbitrary choices in QC had no impact on the presented results.

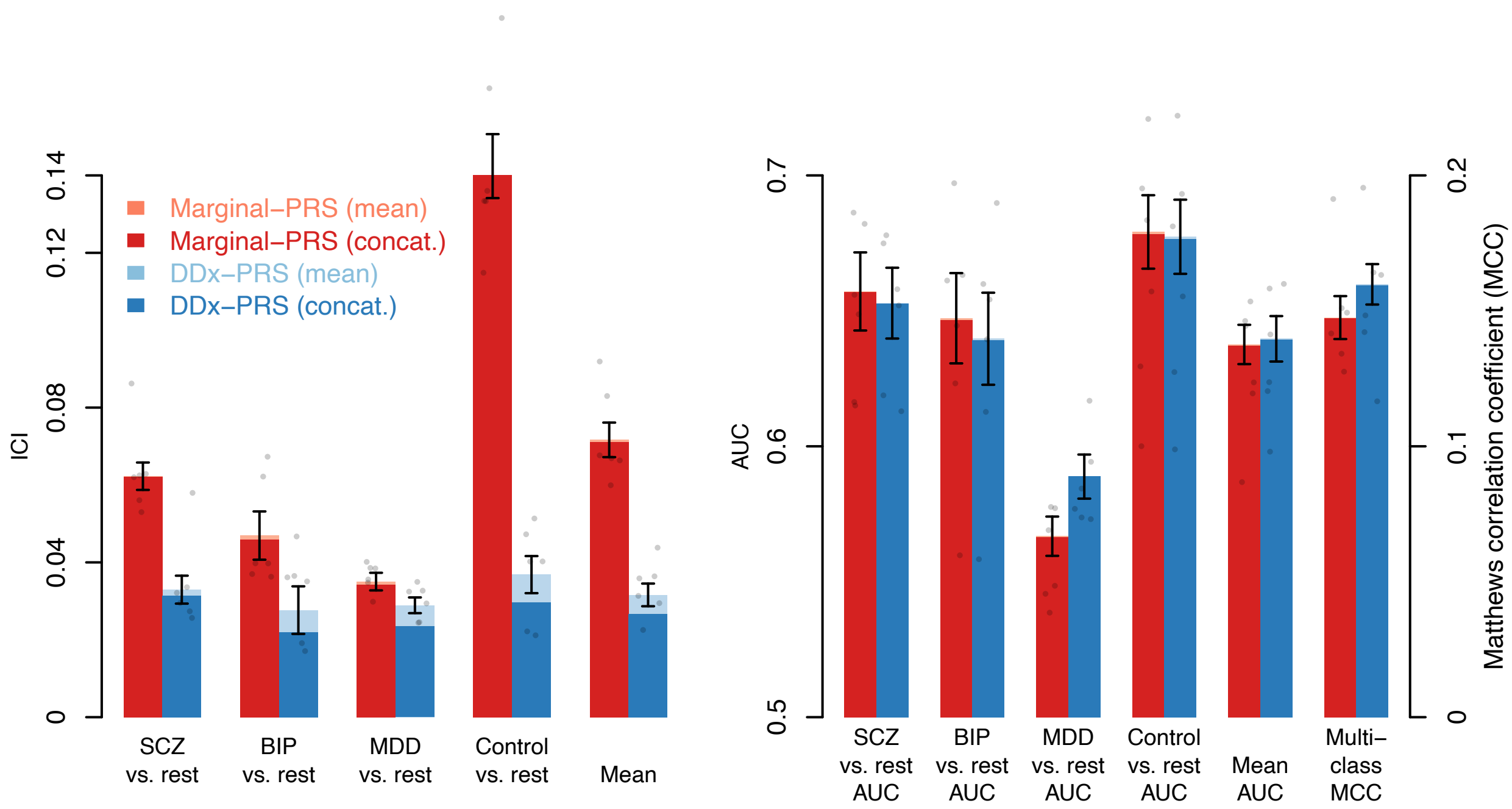

Supplementary Figure 11c. Calibration and accuracy of DDx-PRS and Marginal-PRS for different QC in PGC data. Panel c – looser SNP QC and stricter subject QC. This Figure is analogue to main Figure 3 with SNP QC of INFO>0.3 and MAF>0.01 (compared to INFO>0.9 and MAF>0.10 displayed in Figure 3) and additional stricter subject QC (see Methods). We report the calibration (Integrated Calibration Index; ICI) and accuracy (area under the ROC curve; AUC) for the comparisons of schizophrenia (SCZ) vs. rest, bipolar disorder (BIP) vs. rest, major depressive disorder (MDD) vs. rest, control vs. rest and the mean across these four comparisons, for two methods: Marginal-PRS (red) and DDx-PRS (blue). We also report the multi-class Matthews Correlation Coefficient (MCC). The training data consisted of case-control GWAS summary statistics for SCZ, BIP and MDD; the test data consisted of independent PGC samples subdivided in four cohorts that were matched with respect to country and genotyping platform (total N=10,952, with 2,738 for each of SCZ, BIP, MDD and control; total N main analyses = 11,460). The mean values across these four test cohorts are displayed in light red and light blue, and results based on data concatenated across these four test cohorts are displayed in dark red and dark blue. Error bars denote standard errors. Numerical results are reported in Supplementary Table 14. These secondary analyses show that arbitrary choices in QC had no impact on the presented results.

A. Heatmap of ICI in PGC data

Sample size ratios % (SCZ_BIP_MDD_Control)	DDx-PRS (concat.) SCZ vs. rest	BIP vs. rest	MDD vs. rest	Control vs. rest	Mean	Marginal-PRS (concat.) SCZ vs. rest	BIP vs. rest	MDD vs. rest	Control vs. rest	Mean
25_25_25_25	0.018	0.026	0.016	0.034	0.023	0.050	0.034	0.034	0.118	0.059
40_20_20_20	0.023	0.020	0.017	0.027	0.022	0.082	0.026	0.027	0.136	0.068
20_40_20_20	0.016	0.028	0.017	0.033	0.023	0.039	0.060	0.027	0.128	0.064
20_20_40_20	0.017	0.019	0.018	0.034	0.022	0.043	0.028	0.059	0.131	0.065
20_20_20_40	0.018	0.022	0.009	0.045	0.023	0.034	0.021	0.018	0.075	0.037
85_5_5_5	0.017	0.006	0.009	0.009	0.010	0.085	0.009	0.005	0.085	0.046
5_85_5_5	0.006	0.018	0.005	0.008	0.009	0.011	0.070	0.008	0.071	0.040
5_5_85_5	0.008	0.007	0.012	0.009	0.009	0.008	0.005	0.065	0.074	0.038
5_5_5_85	0.009	0.011	0.010	0.025	0.014	0.009	0.009	0.009	0.029	0.014
30_30_30_10	0.019	0.023	0.022	0.018	0.021	0.071	0.054	0.054	0.181	0.090
33_33_33_0	0.019	0.022	0.026	n/a	0.022	0.035	0.021	0.040	n/a	0.032
All available data	0.019	0.011	0.009	0.006	0.011	0.094	0.040	0.013	0.148	0.074

B. Heatmap of AUC & MCC in PGC data

Sample size ratios % (SCZ_BIP_MDD_Control)	DDx-PRS (concat.) SCZ vs. rest AUC	BIP vs. rest AUC	MDD vs. rest AUC	Control vs. rest AUC	Mean AUC	Multi-class MCC	Marginal-PRS (concat.) SCZ vs. rest AUC	BIP vs. rest AUC	MDD vs. rest AUC	Control vs. rest AUC	Mean AUC	Multi-class MCC
25_25_25_25	0.655	0.639	0.592	0.682	0.642	0.163	0.660	0.645	0.582	0.684	0.642	0.151
40_20_20_20	0.664	0.622	0.607	0.695	0.647	0.146	0.669	0.623	0.576	0.695	0.641	0.170
20_40_20_20	0.658	0.628	0.594	0.693	0.643	0.104	0.658	0.634	0.572	0.695	0.639	0.153
20_20_40_20	0.663	0.627	0.589	0.666	0.636	0.107	0.664	0.635	0.587	0.667	0.638	0.089
20_20_20_40	0.679	0.644	0.575	0.677	0.644	0.150	0.682	0.652	0.580	0.679	0.648	0.131
85_5_5_5	0.679	0.639	0.670	0.744	0.683	0.006	0.678	0.567	0.530	0.743	0.630	0.047
5_85_5_5	0.635	0.626	0.627	0.698	0.647	0.000	0.602	0.615	0.515	0.698	0.608	0.009
5_5_85_5	0.646	0.645	0.581	0.635	0.627	0.000	0.652	0.636	0.590	0.636	0.628	0.000
5_5_5_85	0.697	0.694	0.619	0.680	0.673	0.000	0.696	0.693	0.623	0.682	0.673	0.000
30_30_30_10	0.652	0.613	0.617	0.689	0.643	0.149	0.654	0.619	0.590	0.691	0.638	0.146
33_33_33_0	0.644	0.605	0.643	n/a	0.630	0.172	0.652	0.612	0.640	n/a	0.635	0.176
All available data	0.744	0.779	0.721	0.763	0.752	0.323	0.741	0.769	0.689	0.748	0.737	0.317

Supplementary Figure 12. Heatmap of results of DDx-PRS and Marginal-PRS for different sample size ratios in PGC data. We report the results for DDx-PRS and Marginal PRS for different sample size ratios with matching priors for schizophrenia (SCZ), bipolar disorder (BIP), major depressive disorder (MDD) and controls. The first row with sample size ratios 25/25/25/25 corresponds to the calibration curves plotted in main Figure 4. Panel a reports the Integrated Calibration Index (ICI), the weighted average absolute difference between true and predicted disorder risk, based on the smoothened calibration curve. Panel b reports the AUC (area under the ROC curve) and the multi-class Matthews Correlation Coefficient (MCC). These secondary analyses show that DDx-PRS performs well for the wide range of sample size ratios considered, specifically also for sample size ratios with less/no controls (with a mean AUC of 0.630 for 33/33/33/0, 0.643 for 30/30/30/10, compared to 0.642 for 25/25/25/25). Note that the multi-class Matthews Correlation Coefficient (MCC) of most likely diagnosis is hard to interpret for highly unbalanced sample size ratios (e.g., 85/5/5/5), because in these settings the most likely predicted diagnosis is equal to the diagnosis with the highest prior probability for almost all individuals (e.g. SCZ for 85/5/5/5); in fact, at places where MCC=0, all individuals had most likely diagnosis equal to the diagnosis with the highest prior probability.

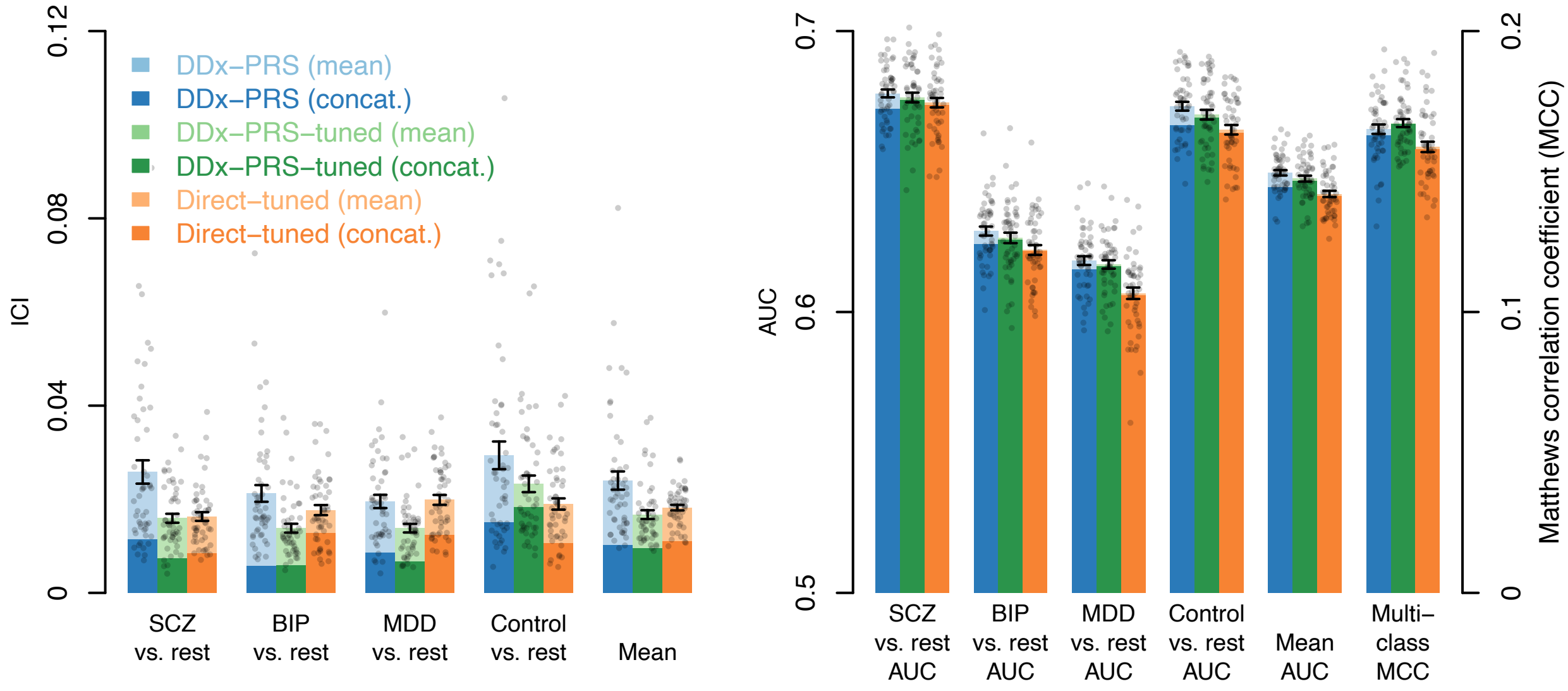

Supplementary Figure 13. Calibration and accuracy of DDx-PRS, DDx-PRS-tuned and Direct-tuned in simulations. We report the calibration (Integrated Calibration Index; ICI) and accuracy (area under the ROC curve; AUC) for the comparisons of schizophrenia (SCZ) vs. rest, bipolar disorder (BIP) vs. rest, major depressive disorder (MDD) vs. rest, control vs. rest and the mean across these four comparisons, for three methods: DDx-PRS (blue), DDx-PRS-tuned (green) and Direct-tuned (orange). We also report the multi-class Matthews Correlation Coefficient (MCC). Results are based on 50 simulation replicates; mean values are displayed in light red and light blue. Simulated tuning data sets comprised of N=200 of each of SCZ, BIP, MDD and control; other simulation parameters are the same as in the main simulations reported in Figure 1. Error bars denote standard errors. Numerical results are reported in Supplementary Table 16. These secondary analyses show that DDx-PRS, DD-PRS-tuned and Direct-tuned yielded similar performance in simulations, which is in line with the results based on PGC data presented in main Figure 5.

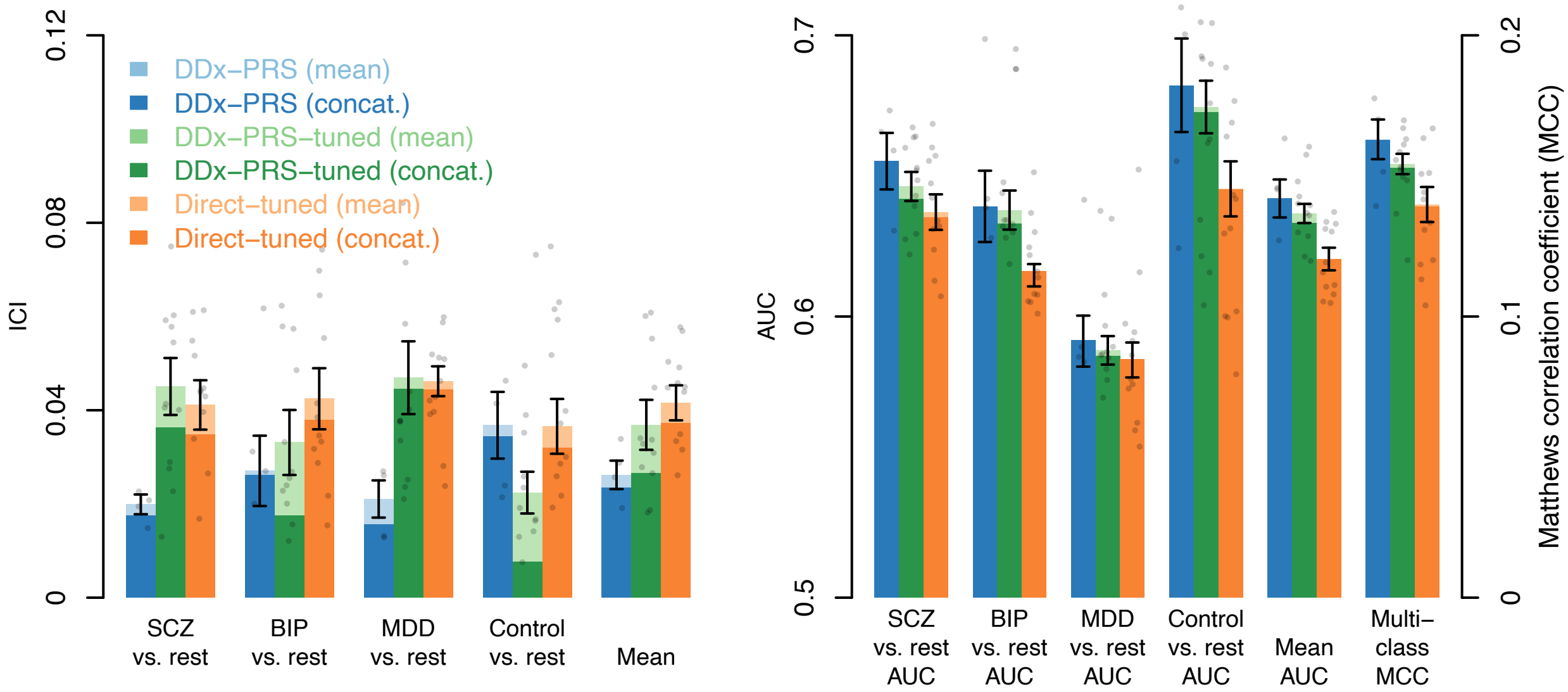

Supplementary Figure 14a. Calibration and accuracy of DDx-PRS-tuned and Direct-tuned at smaller tuning sample sizes in PGC data. Panel a – N tuning 50 for each of SCZ, BIP, MDD control. This Figure is analogue to main Figure 5 (which reports results for N tuning 200 for each of SCZ, BIP, MDD control) and reports the calibration (Integrated Calibration Index; ICI) and accuracy (area under the ROC curve; AUC) for the comparisons of schizophrenia (SCZ) vs. rest, bipolar disorder (BIP) vs. rest, major depressive disorder (MDD) vs. rest, control vs. rest and the mean across these four comparisons, for three methods: DDx-PRS (blue), DDx-PRS-tuned (green) and Direct-tuned (orange). We also report the multi-class Matthews Correlation Coefficient (MCC). The training data consisted of case-control GWAS summary statistics for SCZ, BIP and MDD; the test data consisted of independent PGC samples subdivided in four cohorts that were matched with respect to country and genotyping platform (total N=11,460, with 2,865 for each of SCZ, BIP, MDD and control). For tuning for DDx-PRS-tuned and Direct-tuned, 4x3=12 tuning/test cohort-pairs were analysed with tuning data restricted to 50 samples from each of SCZ, BIP, MDD and control (total N_{tuning}=200). The mean values across these four test cohorts (DDx-PRS) resp. 12 tuning/test cohort-pairs (DDx-PRS-tuned and Direct-tuned) are displayed in light blue, light green and light orange, and results based on data concatenated across these four test cohorts resp. 12 tuning/test cohort-pairs are displayed in dark blue, dark green and dark orange. Error bars denote standard errors. Numerical results are reported in Supplementary Table 17. These secondary analyses show that the results were only slightly impacted by smaller tuning sample size, compared to Figure 5.

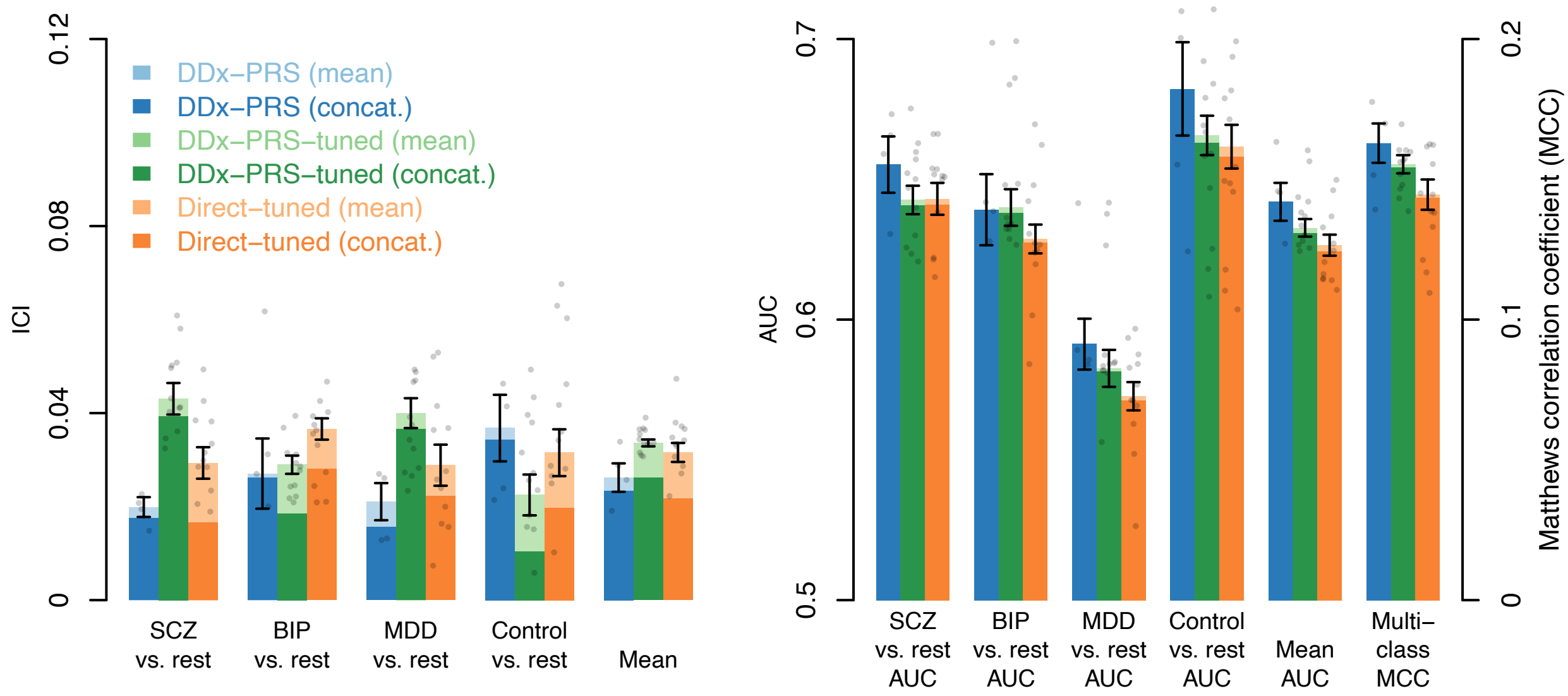

Supplementary Figure 14b. Calibration and accuracy of DDx-PRS-tuned and Direct-tuned at smaller tuning sample sizes in PGC data. Panel b – N tuning 100 for each of SCZ, BIP, MDD control. This Figure is analogue to main Figure 5 (which reports results for N tuning 200 for each of SCZ, BIP, MDD control) and reports the calibration (Integrated Calibration Index; ICI) and accuracy (area under the ROC curve; AUC) for the comparisons of schizophrenia (SCZ) vs. rest, bipolar disorder (BIP) vs. rest, major depressive disorder (MDD) vs. rest, control vs. rest and the mean across these four comparisons, for three methods: DDx-PRS (blue), DDx-PRS-tuned (green) and Direct-tuned (orange). We also report the multi-class Matthews Correlation Coefficient (MCC). We also report the multi-class Matthews Correlation Coefficient (MCC). The training data consisted of case-control GWAS summary statistics for SCZ, BIP and MDD; the test data consisted of independent PGC samples subdivided in four cohorts that were matched with respect to country and genotyping platform (total N=11,460, with 2,865 for each of SCZ, BIP, MDD and control). For tuning for DDx-PRS-tuned and Direct-tuned, 4x3=12 tuning/test cohort-pairs were analysed with tuning data restricted to 10 samples from each of SCZ, BIP, MDD and control (total N_{tuning}=400). The mean values across these four test cohorts (DDx-PRS) resp. 12 tuning/test cohort-pairs (DDx-PRS-tuned and Direct-tuned) are displayed in light blue, light green and light orange, and results based on data concatenated across these four test cohorts resp. 12 tuning/test cohort-pairs are displayed in dark blue, dark green and dark orange. Error bars denote standard errors. Numerical results are reported in Supplementary Table 17. These secondary analyses show that the results were only slightly impacted by smaller tuning sample size, compared to Figure 5.

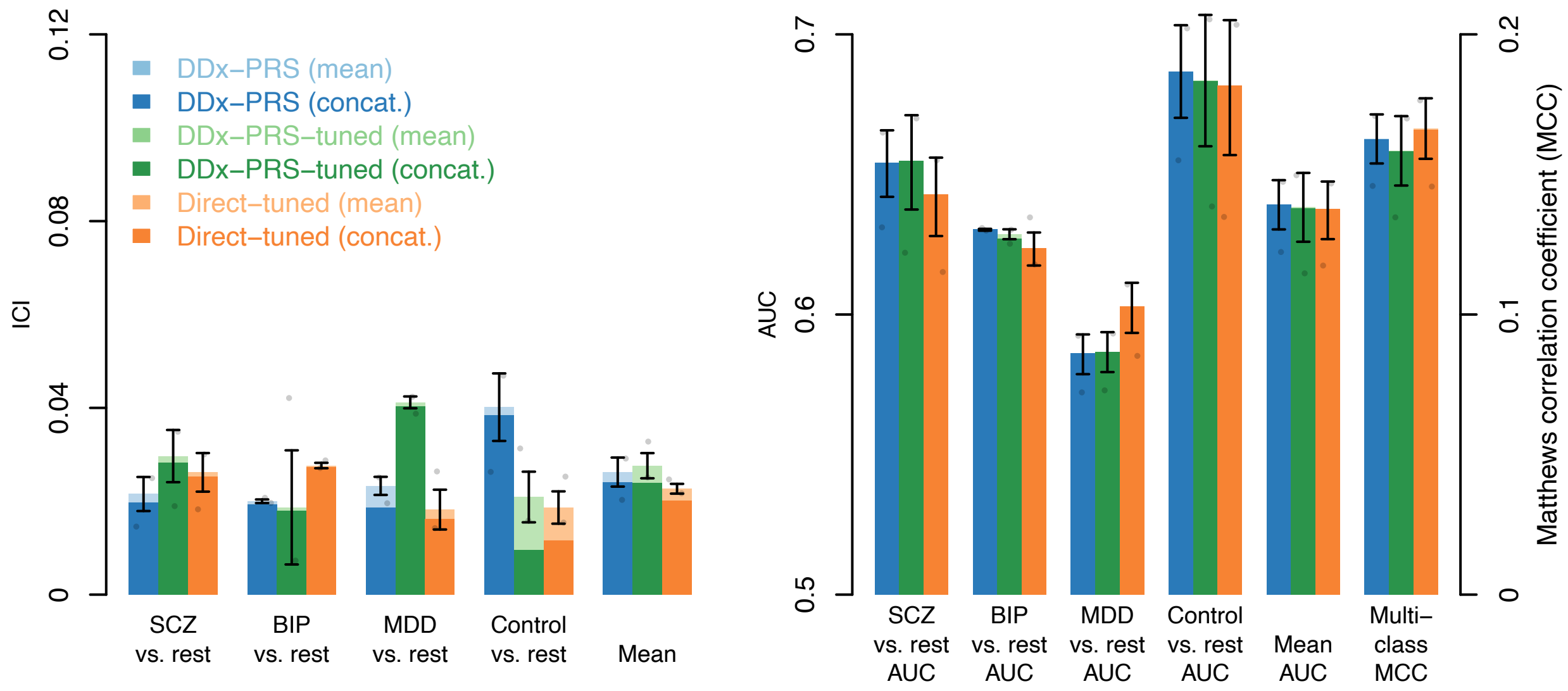

Supplementary Figure 15. Calibration and accuracy of DDx-PRS-tuned and Direct-tuned using within-cohort tuning data in PGC data. This Figure is analogue to main Figure 5 and based on within-cohort tuning (compared to cross-cohort tuning reported in main Figure 5). We report the calibration (Integrated Calibration Index; ICI) and accuracy (area under the ROC curve; AUC) for the comparisons of schizophrenia (SCZ) vs. rest, bipolar disorder (BIP) vs. rest, major depressive disorder (MDD) vs. rest, control vs. rest and the mean across these four comparisons, for three methods: DDx-PRS (blue), DDx-PRS-tuned (green) and Direct-tuned (orange). We also report the multi-class Matthews Correlation Coefficient (MCC). The training data consisted of case-control GWAS summary statistics for SCZ, BIP and MDD; the test data consisted of independent PGC samples subdivided in two cohorts that were matched with respect to country and genotyping platform (GER (N=3,368) and UK2 (N=6,080); sample-sizes of UK1 and USA were too small for within-cohort tuning). For tuning for DDx-PRS-tuned and Direct-tuned, tuning data were randomly selected at 200 samples from each of SCZ, BIP, MDD and control (total N_{tuning}=800); the test data comprised of the remaining samples (i.e. 2,568 for GER and 5,280 for UK2). The mean values across these two test cohorts are displayed in light blue, light green and light orange, and results based on data concatenated across these two test cohorts are displayed in dark blue, dark green and dark orange. Error bars denote standard errors. Numerical results are reported in Supplementary Table 18. These secondary analyses show that the performance of DDx-PRS-tuned and Direct-tuned improved only very slightly when using within-cohort tuning instead of cross-cohort tuning (main Figure 5), and that results for DDx-PRS, DDx-PRS-tuned and Direct-tuned remained comparable.

A. Heatmap of ICI in PGC data comparing DDx-PRS, DDx-PRS-tuned and Direct-tuned

	DDx-PRS (concat.)					DDx-PRS-tuned (concat.)					Direct-tuned (concat.)				
Sample size ratios % (SCZ_BIP_MDD_Control)	SCZ vs. rest	BIP vs. rest	MDD vs. rest	Control vs. rest	Mean	SCZ vs. rest	BIP vs. rest	MDD vs. rest	Control vs. rest	Mean	SCZ vs. rest	BIP vs. rest	MDD vs. rest	Control vs. rest	Mean
25_25_25_25	0.018	0.026	0.016	0.034	0.023	0.033	0.012	0.032	0.009	0.021	0.013	0.020	0.024	0.012	0.017
40_20_20_20	0.023	0.020	0.017	0.027	0.022	0.034	0.012	0.031	0.010	0.022	0.020	0.020	0.016	0.011	0.017
20_40_20_20	0.016	0.028	0.017	0.033	0.023	0.015	0.022	0.032	0.010	0.020	0.014	0.032	0.024	0.014	0.021
20_20_40_20	0.017	0.019	0.018	0.034	0.022	0.024	0.017	0.041	0.014	0.024	0.012	0.023	0.032	0.012	0.020
20_20_20_40	0.018	0.022	0.009	0.045	0.023	0.023	0.013	0.021	0.015	0.018	0.010	0.020	0.025	0.020	0.019
85_5_5_5	0.017	0.006	0.009	0.009	0.010	0.021	0.004	0.014	0.007	0.012	0.015	0.004	0.007	0.005	0.008
5_85_5_5	0.006	0.018	0.005	0.008	0.009	0.007	0.023	0.015	0.011	0.014	0.007	0.029	0.012	0.009	0.014
5_5_85_5	0.008	0.007	0.012	0.009	0.009	0.015	0.009	0.026	0.008	0.015	0.008	0.010	0.020	0.004	0.010
5_5_5_85	0.009	0.011	0.010	0.025	0.014	0.011	0.006	0.009	0.009	0.009	0.006	0.007	0.005	0.012	0.008
30_30_30_10	0.019	0.023	0.022	0.018	0.021	0.028	0.018	0.044	0.006	0.024	0.017	0.031	0.023	0.008	0.020
33_33_33_0	0.019	0.022	0.026	n/a	0.022	0.029	0.019	0.053	n/a	0.034	0.016	0.033	0.023	n/a	0.024
All available data	0.019	0.011	0.009	0.006	0.011	0.0218	0.0183	0.0155	0.0086	0.0161	0.0156	0.0138	0.0075	0.0055	0.0106

B. Heatmap of AUC and MCC in PGC data comparing DDx-PRS, DDx-PRS-tuned and Direct-tuned

	DDx-PRS (concat.)						DDx-PRS-tuned (concat.)						Direct-tuned (concat.)					
Sample size ratios % (SCZ_BIP_MDD_Control)	SCZ vs. rest AUC	BIP vs. rest AUC	MDD vs. rest AUC	Control vs. rest AUC	Mean AUC	Multi-class MCC	SCZ vs. rest AUC	BIP vs. rest AUC	MDD vs. rest AUC	Control vs. rest AUC	Mean AUC	Multi-class MCC	SCZ vs. rest AUC	BIP vs. rest AUC	MDD vs. rest AUC	Control vs. rest AUC	Mean AUC	Multi-class MCC
25_25_25_25	0.655	0.639	0.592	0.682	0.642	0.163	0.644	0.638	0.583	0.671	0.634	0.159	0.649	0.633	0.573	0.667	0.630	0.155
40_20_20_20	0.664	0.622	0.607	0.695	0.647	0.146	0.652	0.621	0.596	0.682	0.638	0.147	0.657	0.615	0.591	0.681	0.636	0.133
20_40_20_20	0.658	0.628	0.594	0.693	0.643	0.104	0.648	0.627	0.585	0.680	0.635	0.135	0.649	0.623	0.576	0.678	0.632	0.126
20_20_40_20	0.663	0.627	0.589	0.666	0.636	0.107	0.652	0.628	0.579	0.658	0.629	0.115	0.655	0.625	0.569	0.646	0.624	0.103
20_20_20_40	0.679	0.644	0.575	0.677	0.644	0.150	0.666	0.644	0.565	0.667	0.636	0.159	0.670	0.639	0.550	0.663	0.630	0.154
85_5_5_5	0.679	0.639	0.670	0.744	0.683	0.006	0.666	0.637	0.653	0.725	0.670	0.026	0.669	0.619	0.656	0.729	0.668	0.003
5_85_5_5	0.635	0.626	0.627	0.698	0.647	0.000	0.633	0.624	0.624	0.686	0.642	0.024	0.621	0.619	0.612	0.688	0.635	0.039
5_5_85_5	0.646	0.645	0.581	0.635	0.627	0.000	0.632	0.640	0.571	0.646	0.622	0.003	0.645	0.630	0.562	0.601	0.609	0.012
5_5_5_85	0.697	0.694	0.619	0.680	0.673	0.000	0.683	0.685	0.617	0.668	0.663	0.027	0.691	0.679	0.582	0.666	0.654	0.037
30_30_30_10	0.652	0.613	0.617	0.689	0.643	0.149	0.641	0.614	0.606	0.676	0.635	0.148	0.646	0.609	0.601	0.672	0.632	0.140
33_33_33_0	0.644	0.605	0.643	n/a	0.630	0.172	0.635	0.606	0.632	n/a	0.624	0.170	0.639	0.600	0.630	n/a	0.623	0.161
All available data	0.744	0.779	0.721	0.763	0.752	0.323	0.736	0.778	0.708	0.753	0.744	0.318	0.737	0.771	0.715	0.755	0.745	0.319

Supplementary Figure 16. Heatmaps of results of DDx-PRS, DDx-PRS-tuned and Direct-tuned for different sample size ratios in PGC data in PGC data. We report the results for DDx-PRS, DDx-PRS-tuned and Direct-tuned for different sample size ratios with matching priors for schizophrenia (SCZ), bipolar disorder (BIP), major depressive disorder (MDD) and controls. The first row with sample size ratio 25/25/25/25 corresponds to the calibration curves plotted in main Extended Data Figure 1. Panel a reports the Integrated Calibration Index (ICI), the weighted average absolute difference between true and predicted disorder risk, based on the smoothened calibration curve. Panel b reports the AUC (area under the ROC curve) and the multi-class Matthews Correlation Coefficient (MCC). These secondary analyses show that DDx-PRS performs well for the wide range of sample size ratios considered, specifically also for sample size ratios with less/no controls (with a mean AUC of 0.630 for 33/33/33/0, 0.643 for 30/30/30/10, compared to 0.642 for 25/25/25/25). Note that the multi-class Matthews Correlation Coefficient (MCC) of most likely diagnosis is hard to interpret for highly unbalanced sample size ratios (e.g., 85/5/5/5), because in these settings the most likely predicted diagnosis is equal to the diagnosis with the highest prior probability for almost all individuals (e.g. SCZ for 85/5/5/5); in fact, at places where MCC=0, all individuals had most likely diagnosis equal to the diagnosis with the highest prior probability. These secondary analyses show that DDx-PRS, DDx-PRS-tuned and Direct-tuned yield highly similar performance across a wide range of sample size ratios.

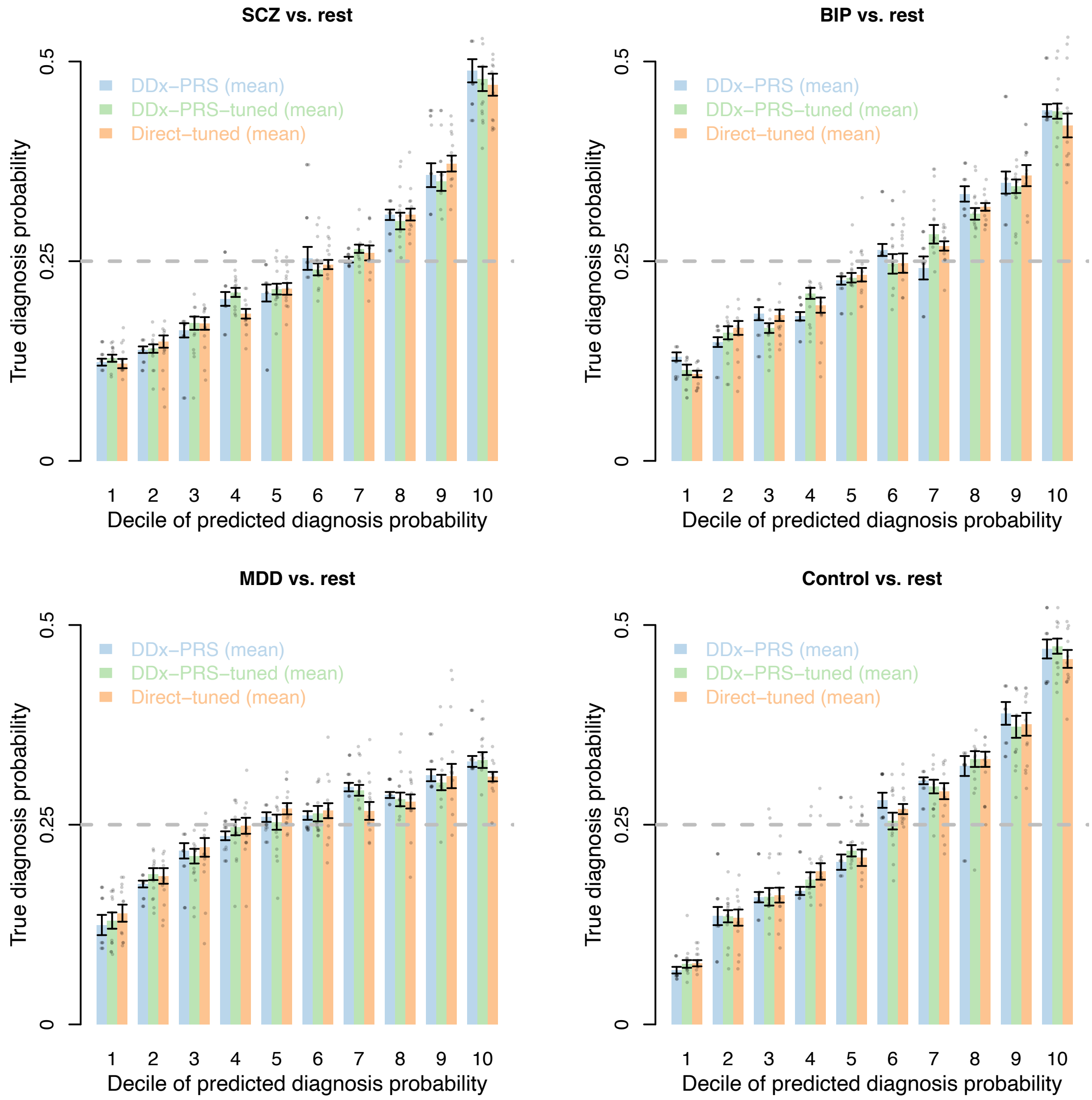

Supplementary Figure 17. True diagnosis probability per decile of predicted diagnosis probability of DDx-PRS, DDx-PRS-tuned and Direct-tuned in PGC data. This Figure accompanies main Figure 6 and reports the true diagnosis probability per decile of predicted diagnosis probability for the comparisons of schizophrenia (SCZ) vs. rest, bipolar disorder (BIP) vs. rest, major depressive disorder (MDD) vs. rest, and control vs. rest, for three methods: DDx-PRS (blue), DDx-PRS-tuned (green) and Direct-tuned (orange). The training data consisted of case-control GWAS summary statistics for SCZ, BIP and MDD; the test data consisted of independent PGC samples subdivided in four cohorts that were matched with respect to country and genotyping platform (total N=11,460, with 2,865 for each of SCZ, BIP, MDD and control). For tuning for DDx-PRS-tuned and Direct-tuned, 4x3=12 tuning/test cohort-pairs were analysed with tuning data restricted to 200 samples from each of SCZ, BIP, MDD and control (total N_{tuning}=800). The mean values across these four test cohorts (DDx-PRS) resp. 12 tuning/test cohort-pairs (DDx-PRS-tuned and Direct-tuned) are displayed in light blue, light green and light orange. Error bars denote standard errors. Numerical results are reported in Supplementary Table 21. These secondary analyses show that the results are similar for DDx-PRS, DDx-PRS-tuned and Direct-tuned.

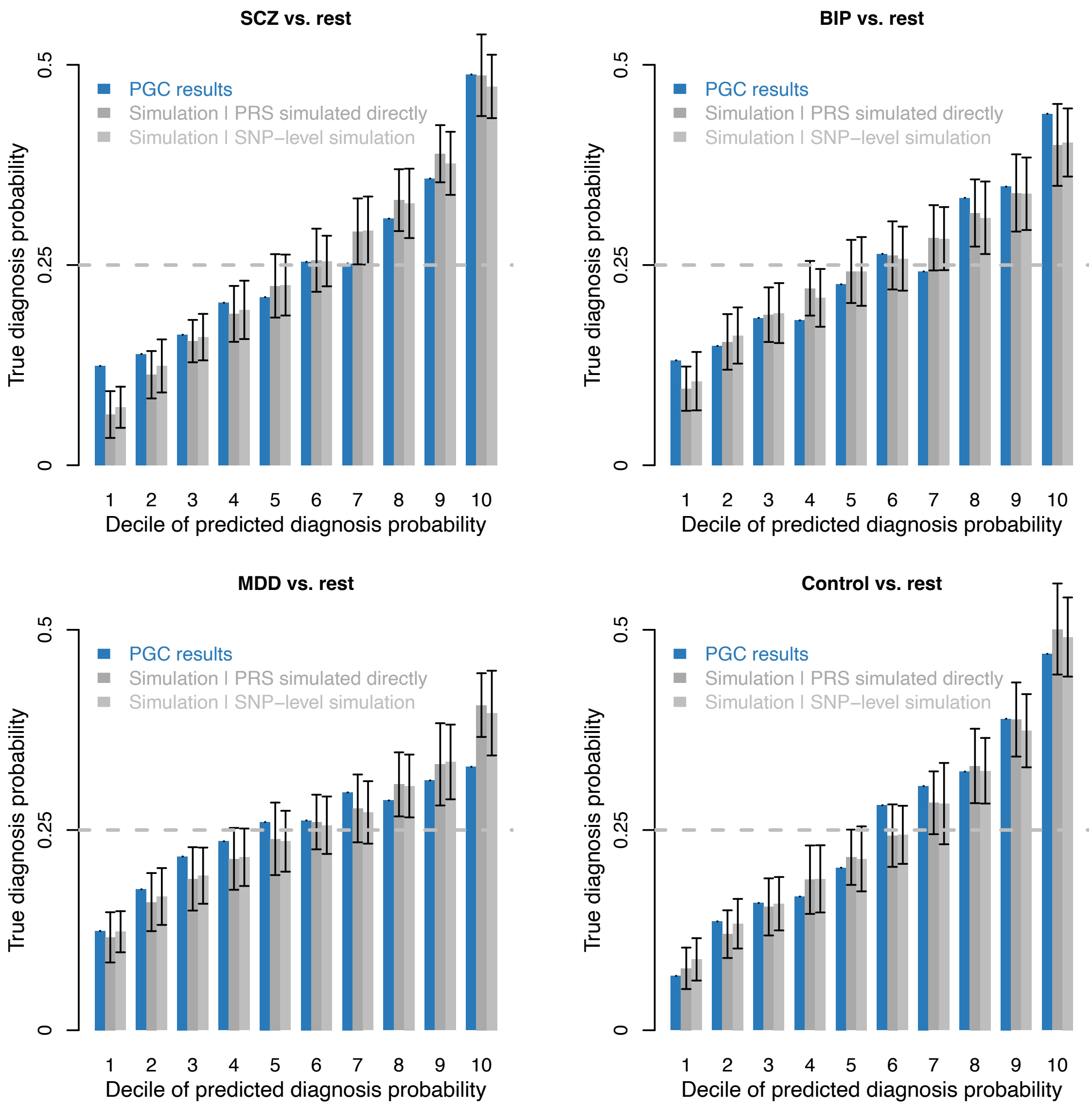

Supplementary Figure 18. True diagnosis probability per decile of predicted diagnosis probability of DDx-PRS in PGC data and in simulations at the exact empirical values. We report the true diagnosis probability per decile of predicted diagnosis probability for the comparisons of schizophrenia (SCZ) vs. rest, bipolar disorder (BIP) vs. rest, major depressive disorder (MDD) vs. rest, and control vs. rest, for one method: DDx-PRS. Empirical results in PGC data are reported in blue, and present the results based on concatenated data (see Supplementary Figure 14). Results based on simulating PRS directly are displayed in dark grey and results based on simulating SNP-level data are reported in light grey; error bars of simulation denote 1.96 times the SE around the mean across 50 simulation runs. Parameters of simulations are set to the exact empirical values of % of SNP-heritability captured by case-control PRS (i.e., 42% for SCZ, 47% for BIP, and 44% for MDD); all other parameters are the same as in the main simulations. Numerical results are reported in Supplementary Table 22. These secondary analyses show that simulation results were close to the empirical results based on PGC data, which suggests that the simulations can be used to approximate projections of clinical utility at larger training sample sizes.

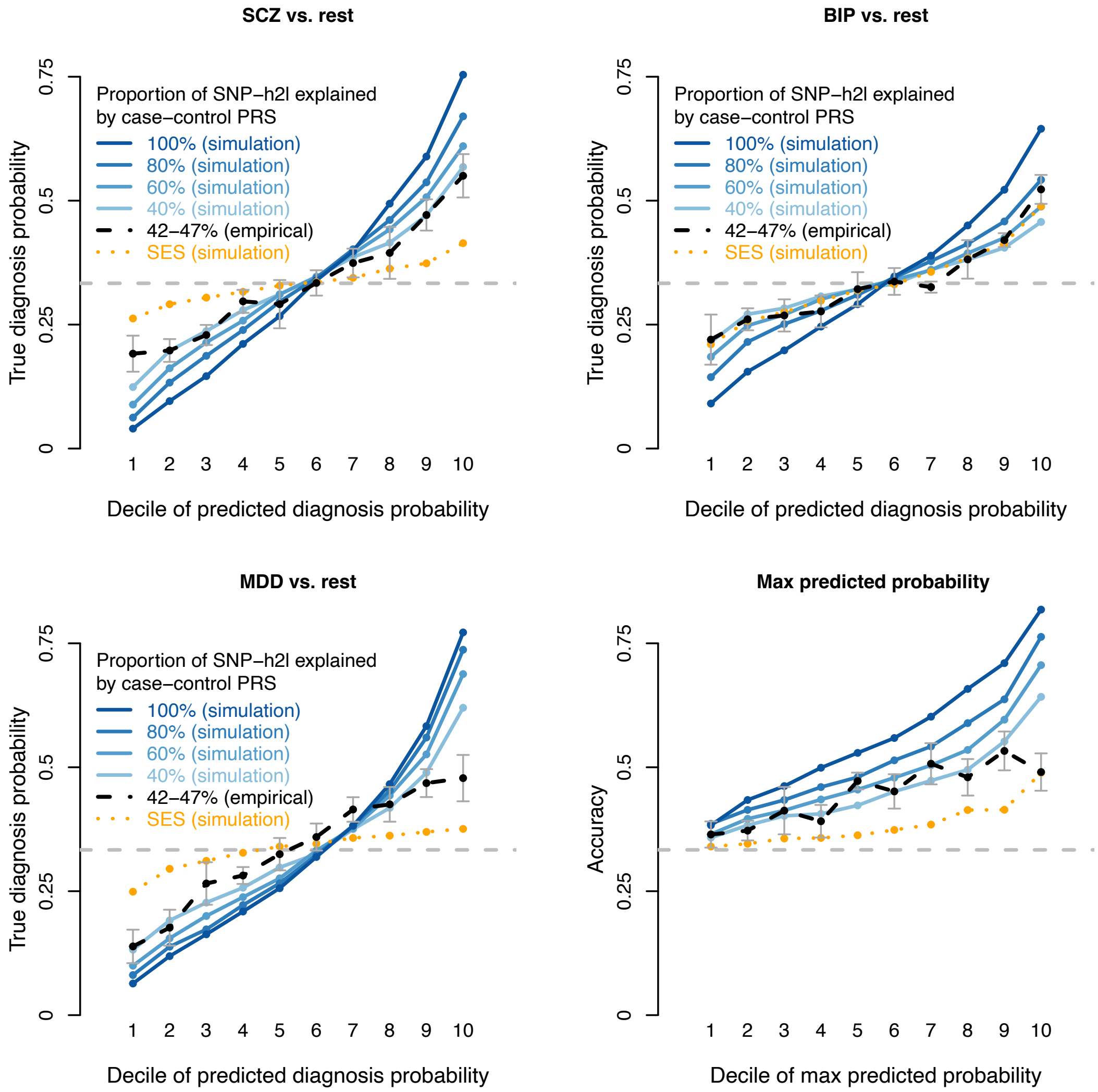

Supplementary Figure 19. Projections at larger training sample sizes for the prior 33/33/33/0 for SCZ/BIP/MDD/control. (This Figure is analogue to main Figure 6 (reporting results for the prior 25/25/25/25).) We report the true diagnosis probability per decile of predicted diagnosis probability for the comparisons of schizophrenia (SCZ) vs. rest, bipolar disorder (BIP) vs. rest, and major depressive disorder (MDD) vs. rest, and maximum (Max) predicted probability vs. rest, for DDx-PRS. Empirical results in PGC data are reported as black points (error bars in grey) connected with dashed black lines; error bars denote 1.96 times the SE around the mean across the four PGC test cohorts. Simulation results at various values of SNP-heritability captured by case-control PRS (40%, 60%, 80% and 100%) are reported as blue points connected with solid blue lines, with varying colour intensity; these results represent the mean across 50 simulations runs. We also show the expected potential to distinguish diagnostic categories using socio-economic status (SES), based on simulations); these results are based on a single simulation run in a very large population of N=5,000,000. The baseline diagnosis probability (i.e. prior clinical probability) equals 0.33 for each of SCZ, BIP and MDD (dashed grey horizontal lines). We note that the true diagnosis probability for Max predicted probability vs. rest corresponds to Accuracy of most likely diagnosis. These secondary analyses show that DDx-PRS has appreciable potential for clinical utility at larger training sample sizes, also when controls are omitted.

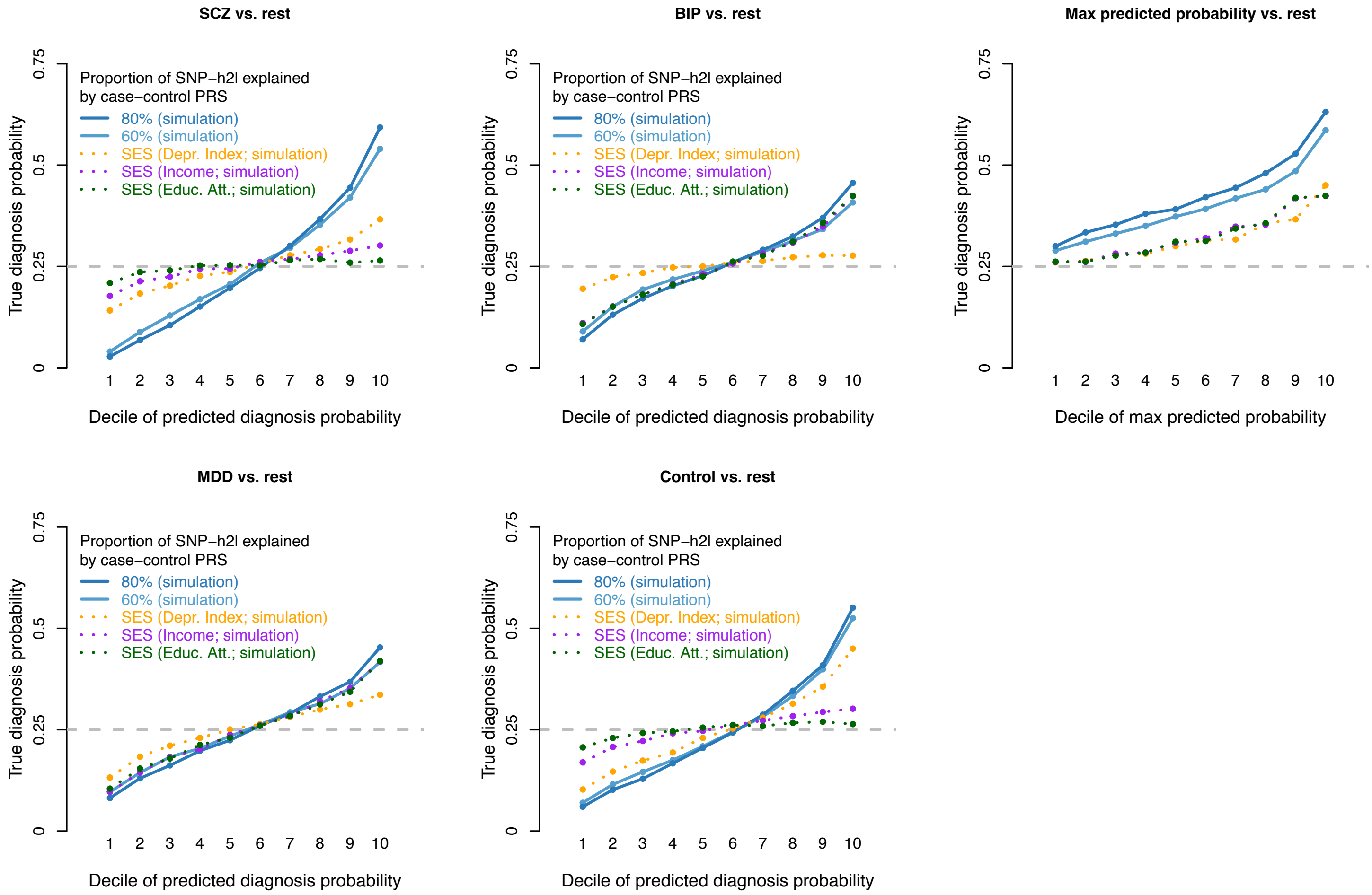

Supplementary Figure 20. Simulated expected predictive performance of multiple SES measurements. We report the true diagnosis probability per decile of predicted diagnosis probability for the comparisons of schizophrenia (SCZ) vs. rest, bipolar disorder (BIP) vs. rest, major depressive disorder (MDD) vs. rest, control vs. rest, and maximum (Max) predicted probability vs. rest, for DDx-PRS. Simulations results of DDx-PRS is reported at various values of SNP-heritability captured by case-control PRS (60%, 80%) are reported as blue points connected with solid blue lines, with varying colour intensity; these results represent the mean across 50 simulations runs. We also show the expected potential to distinguish diagnostic categories using socio-economic status (SES) based on simulations for three SES measurements: Townsend deprivation index with assumed correlations of 0.21, 0.07, 0.36 between SES and the liabilities of SCZ, BIP, MDD respectively; Income with assumed correlations of -0.09, 0.11, -0.29 respectively; Educational Attainment with assumed correlations of 0.06, 0.19, -0.17 respectively (all assumed correlations are based on the genetic correlation estimated in Wendt et al. 2021 Nat Hum Behav); these results are based on a single simulation run in a very large population of N=5,000,000. The baseline diagnosis probability (i.e. prior clinical probability) equals 0.25 for each of SCZ, BIP, MDD and control (dashed grey horizontal lines). We note that the true diagnosis probability for Max predicted probability vs. rest corresponds to Accuracy of most likely diagnosis. These secondary results show that different SES measurements yield comparable potential to distinguish SCZ, BIP, MDD and controls.

References

1. Vilhjálmsson, B. J. *et al.* Modeling Linkage Disequilibrium Increases Accuracy of Polygenic Risk Scores. *Am J Hum Genet* **97**, 576–592 (2015).
2. van der Gaag, M. *et al.* Preventing a first episode of psychosis: Meta-analysis of randomized controlled prevention trials of 12month and longer-term follow-ups. *Schizophrenia Research* **149**, 56–62 (2013).
3. Ising, H. K. *et al.* Four-Year Follow-up of Cognitive Behavioral Therapy in Persons at Ultra-High Risk for Developing Psychosis: The Dutch Early Detection Intervention Evaluation (EDIE-NL) Trial. *Schizophrenia Bulletin* **42**, 1243–1252 (2016).
4. Iversen, T. S. J. *et al.* Side effect burden of antipsychotic drugs in real life - Impact of gender and polypharmacy. *Prog Neuropsychopharmacol Biol Psychiatry* **82**, 263–271 (2018).
5. Solmi, M. *et al.* Safety of 80 antidepressants, antipsychotics, anti-attention-deficit/hyperactivity medications and mood stabilizers in children and adolescents with psychiatric disorders: a large scale systematic meta-review of 78 adverse effects. *World Psychiatry* **19**, 214–232 (2020).
6. Trubetskoy, V. *et al.* Mapping genomic loci implicates genes and synaptic biology in schizophrenia. *Nature* **604**, 502–508 (2022).
7. Ge, T., Chen, C.-Y., Ni, Y., Feng, Y.-C. A. & Smoller, J. W. Polygenic prediction via Bayesian regression and continuous shrinkage priors. *Nat Commun* **10**, 1776 (2019).
8. Murray, G. K. *et al.* Could Polygenic Risk Scores Be Useful in Psychiatry?: A Review. *JAMA Psychiatry* **78**, 210–219 (2021).
9. Yung, A. R. & McGorry, P. D. The Prodromal Phase of First-episode Psychosis: Past and Current Conceptualizations. *Schizophrenia Bulletin* **22**, 353–370 (1996).
10. McHugh, M. J. *et al.* The Ultra-High-Risk for psychosis groups: Evidence to maintain the status quo. *Schizophr Res* **195**, 543–548 (2018).
11. International Early Psychosis Association Writing Group. International clinical practice guidelines for early psychosis. *Br J Psychiatry Suppl* **48**, s120-124 (2005).
12. Nelson, B. *et al.* Long-term follow-up of a group at ultra high risk ('prodromal') for psychosis: the PACE 400 study. *JAMA Psychiatry* **70**, 793–802 (2013).
13. Hartmann, J. A. *et al.* Declining transition rates to psychotic disorder in "ultra-high risk" clients: Investigation of a dilution effect. *Schizophrenia Research* **170**, 130–136 (2016).
14. Salazar de Pablo, G. *et al.* Probability of Transition to Psychosis in Individuals at Clinical High Risk: An Updated Meta-analysis. *JAMA Psychiatry* **78**, 970–978 (2021).
15. Raballo, A., Poletti, M., Preti, A. & McGorry, P. Clinical high risk for psychosis in children and adolescents: A meta-analysis of transition prevalences. *Schizophrenia Research* **243**, 254–261 (2022).
16. Hartmann, S. *et al.* Development and temporal validation of a clinical prediction model of transition to psychosis in individuals at ultra-high risk in the UHR 1000+ cohort. *World Psychiatry* **23**, 400–410 (2024).
17. Bromet, E. J. *et al.* Diagnostic shifts during the decade following first admission for psychosis. *Am J Psychiatry* **168**, 1186–1194 (2011).
18. Clayton, E. W. Ethical, Legal, and Social Implications of Genomic Medicine. *New England Journal of Medicine* **349**, 562–569 (2003).
19. Henneman, L., van El, C. G. & Cornel, M. C. Genetic testing and implications for personalized medicine: changes in public and healthcare professional perspectives. *Per Med* **10**, 217–219 (2013).

20. Turley, P. *et al.* Problems with Using Polygenic Scores to Select Embryos. *N Engl J Med* **385**, 78–86 (2021).
21. Hyman, S. E. The diagnosis of mental disorders: the problem of reification. *Annu Rev Clin Psychol* **6**, 155–179 (2010).
22. Sullivan, P. F. *et al.* Psychiatric Genomics: An Update and an Agenda. *Am J Psychiatry* **175**, 15–27 (2018).
23. Hyman, S. E. Psychiatric Disorders: Grounded in Human Biology but Not Natural Kinds. *Perspect Biol Med* **64**, 6–28 (2021).
24. Hyman, S. E. The Biology of Mental Disorders: Progress at Last. *Daedalus* **152**, 186–211 (2023).
25. Knevel, R. *et al.* Using genetics to prioritize diagnoses for rheumatology outpatients with inflammatory arthritis. *Sci Transl Med* **12**, eaay1548 (2020).
26. O'Neal, K. S., Johnson, J. L. & Panak, R. L. Recognizing and Appropriately Treating Latent Autoimmune Diabetes in Adults. *Diabetes Spectr* **29**, 249–252 (2016).
27. Tho, N. V., Park, H. Y. & Nakano, Y. Asthma-COPD overlap syndrome (ACOS): A diagnostic challenge. *Respirology* **21**, 410–418 (2016).
28. Mullins, N. *et al.* Genome-wide association study of more than 40,000 bipolar disorder cases provides new insights into the underlying biology. *Nat Genet* **53**, 817–829 (2021).
29. Wray, N. R. *et al.* Genome-wide association analyses identify 44 risk variants and refine the genetic architecture of major depression. *Nat Genet* **50**, 668–681 (2018).
30. Howard, D. M. *et al.* Genome-wide meta-analysis of depression identifies 102 independent variants and highlights the importance of the prefrontal brain regions. *Nat Neurosci* **22**, 343–352 (2019).
31. Márquez-Luna, C., Loh, P.-R., South Asian Type 2 Diabetes (SAT2D) Consortium, SIGMA Type 2 Diabetes Consortium & Price, A. L. Multiethnic polygenic risk scores improve risk prediction in diverse populations. *Genet Epidemiol* **41**, 811–823 (2017).
32. Martin, A. R. *et al.* Clinical use of current polygenic risk scores may exacerbate health disparities. *Nat Genet* **51**, 584–591 (2019).
33. Ding, Y. *et al.* Polygenic scoring accuracy varies across the genetic ancestry continuum. *Nature* **618**, 774–781 (2023).
34. Patel, A. P. *et al.* A multi-ancestry polygenic risk score improves risk prediction for coronary artery disease. *Nat Med* **29**, 1793–1803 (2023).
35. Lam, M. *et al.* Comparative genetic architectures of schizophrenia in East Asian and European populations. *Nat Genet* **51**, 1670–1678 (2019).
36. Giannakopoulou, O. *et al.* The Genetic Architecture of Depression in Individuals of East Asian Ancestry: A Genome-Wide Association Study. *JAMA Psychiatry* **78**, 1258–1269 (2021).
37. Weissbrod, O. *et al.* Leveraging fine-mapping and multipopulation training data to improve cross-population polygenic risk scores. *Nat Genet* **54**, 450–458 (2022).
38. Ruan, Y. *et al.* Improving polygenic prediction in ancestrally diverse populations. *Nat Genet* **54**, 573–580 (2022).
39. Zhang, H. *et al.* A new method for multiancestry polygenic prediction improves performance across diverse populations. *Nat Genet* **55**, 1757–1768 (2023).
40. Kachuri, L. *et al.* Principles and methods for transferring polygenic risk scores across global populations. *Nat Rev Genet* **25**, 8–25 (2024).
41. Hou, K. *et al.* Calibrated prediction intervals for polygenic scores across diverse contexts. *Nat Genet* **56**, 1386–1396 (2024).

42. Karayiorgou, M. *et al.* Schizophrenia susceptibility associated with interstitial deletions of chromosome 22q11. *Proc Natl Acad Sci U S A* **92**, 7612–7616 (1995).
43. Marshall, C. R. *et al.* Contribution of copy number variants to schizophrenia from a genome-wide study of 41,321 subjects. *Nat Genet* **49**, 27–35 (2017).
44. Chen, T. & Guestrin, C. XGBoost: A Scalable Tree Boosting System. in *Proceedings of the 22nd ACM SIGKDD International Conference on Knowledge Discovery and Data Mining* 785–794 (Association for Computing Machinery, New York, NY, USA, 2016). doi:10.1145/2939672.2939785.
45. Cabrera-Mendoza, B., Wendt, F. R., Pathak, G. A., Yengo, L. & Polimanti, R. The impact of assortative mating, participation bias and socioeconomic status on the polygenic risk of behavioural and psychiatric traits. *Nat Hum Behav* **8**, 976–987 (2024).
46. Cai, N. *et al.* Assessment and ascertainment in psychiatric molecular genetics: challenges and opportunities for cross-disorder research. *Mol Psychiatry* https://doi.org/10.1038/s41380-024-02878-x (2024) doi:10.1038/s41380-024-02878-x.
47. So, H.-C., Kwan, J. S. H., Cherny, S. S. & Sham, P. C. Risk prediction of complex diseases from family history and known susceptibility loci, with applications for cancer screening. *Am J Hum Genet* **88**, 548–565 (2011).
48. Do, C. B., Hinds, D. A., Francke, U. & Eriksson, N. Comparison of family history and SNPs for predicting risk of complex disease. *PLoS Genet* **8**, e1002973 (2012).
49. Hujoel, M. L. A., Loh, P.-R., Neale, B. M. & Price, A. L. Incorporating family history of disease improves polygenic risk scores in diverse populations. *Cell Genom* **2**, 100152 (2022).
50. Falconer, D. S. The inheritance of liability to diseases with variable age of onset, with particular reference to diabetes mellitus. *Ann Hum Genet* **31**, 1–20 (1967).
51. Bulik-Sullivan, B. *et al.* An atlas of genetic correlations across human diseases and traits. *Nat Genet* **47**, 1236–1241 (2015).
52. Loh, P.-R. *et al.* Contrasting genetic architectures of schizophrenia and other complex diseases using fast variance-components analysis. *Nat Genet* **47**, 1385–1392 (2015).
53. Sodini, S. M., Kemper, K. E., Wray, N. R. & Trzaskowski, M. Comparison of Genotypic and Phenotypic Correlations: Cheverud's Conjecture in Humans. *Genetics* **209**, 941–948 (2018).
54. Sullivan, P. F. & Geschwind, D. H. Defining the Genetic, Genomic, Cellular, and Diagnostic Architectures of Psychiatric Disorders. *Cell* **177**, 162–183 (2019).
55. Auton, A. *et al.* A global reference for human genetic variation. *Nature* **526**, 68–74 (2015).
56. Lee, S. H., Wray, N. R., Goddard, M. E. & Visscher, P. M. Estimating missing heritability for disease from genome-wide association studies. *Am J Hum Genet* **88**, 294–305 (2011).
57. Peyrot, W. J. & Price, A. L. Identifying loci with different allele frequencies among cases of eight psychiatric disorders using CC-GWAS. *Nat Genet* **53**, 445–454 (2021).
58. Uffelmann, E. *et al.* Estimating Disorder Probability Based on Polygenic Prediction Using the BPC Approach. *MedRxiv [revision at Nature Communications]* 2024.01.12.24301157 (2024) doi:10.1101/2024.01.12.24301157.
59. Lloyd-Jones, L. R. *et al.* Improved polygenic prediction by Bayesian multiple regression on summary statistics. *Nat Commun* **10**, 5086 (2019).
60. Finucane, H. K. *et al.* Partitioning heritability by functional annotation using genome-wide association summary statistics. *Nat Genet* **47**, 1228–1235 (2015).
61. Gazal, S. *et al.* Linkage disequilibrium-dependent architecture of human complex traits shows action of negative selection. *Nat Genet* **49**, 1421–1427 (2017).

62. Gazal, S., Marquez-Luna, C., Finucane, H. K. & Price, A. L. Reconciling S-LDSC and LDAK functional enrichment estimates. *Nat Genet* **51**, 1202–1204 (2019).
63. Tallis, G. M. Ancestral covariance and the Bulmer effect. *Theor Appl Genet* **73**, 815–820 (1987).
64. Peyrot, W. J., Boomsma, D. I., Penninx, B. W. J. H. & Wray, N. R. Disease and Polygenic Architecture: Avoid Trio Design and Appropriately Account for Unscreened Control Subjects for Common Disease. *Am J Hum Genet* **98**, 382–391 (2016).
65. Lee, S. H., Goddard, M. E., Wray, N. R. & Visscher, P. M. A better coefficient of determination for genetic profile analysis. *Genet Epidemiol* **36**, 214–224 (2012).
66. Golan, D., Lander, E. S. & Rosset, S. Measuring missing heritability: inferring the contribution of common variants. *Proc Natl Acad Sci U S A* **111**, E5272-5281 (2014).
67. Dudbridge, F. Power and predictive accuracy of polygenic risk scores. *PLoS Genet* **9**, e1003348 (2013).
68. Sullivan, P. F. The psychiatric GWAS consortium: big science comes to psychiatry. *Neuron* **68**, 182–186 (2010).
69. Wray, N. R. *et al.* Pitfalls of predicting complex traits from SNPs. *Nat Rev Genet* **14**, 507–515 (2013).
